# Supplementary material for: HD 66051, an eclipsing binary hosting a highly peculiar, HgMn-related star
Source: Sci Rep. 2017 Jul 19;7:5906. doi: 10.1038/s41598-017-05987-6 (PMC5517476; doi:10.1038/s41598-017-05987-6)
Supplement: Supplementary file 1 — Supplementary Dataset 1 [file 41598_2017_5987_MOESM1_ESM.doc]

HD66051, an eclipsing binary hosting a highly peculiar, HgMn-related star

Ewa Niemczura

Stefan Hümmerich

Fiorella Castelli

Ernst Paunzen

Klaus Bernhard

Franz-Josef Hambsch

Krzysztof Hełminiak

JD mag(B) mag_err

2457669.75419 8.834 0.025

2457669.75675 8.808 0.023

2457669.75929 8.848 0.022

2457669.76185 8.859 0.020

2457669.76442 8.836 0.019

2457669.76698 8.836 0.018

2457669.76953 8.834 0.017

2457669.77211 8.826 0.017

2457669.77468 8.860 0.016

2457669.77723 8.861 0.015

2457669.77980 8.868 0.015

2457669.78236 8.826 0.015

2457669.78491 8.861 0.014

2457669.78748 8.869 0.013

2457669.79012 8.835 0.013

2457669.79266 8.851 0.013

2457669.79522 8.859 0.013

2457669.79779 8.830 0.013

2457669.80035 8.842 0.012

2457669.80291 8.856 0.012

2457669.80546 8.859 0.012

2457669.80802 8.851 0.012

2457669.81057 8.846 0.011

2457669.81313 8.851 0.012

2457669.81567 8.854 0.011

2457669.81823 8.863 0.011

2457669.82082 8.857 0.011

2457669.82339 8.867 0.011

2457669.82850 8.860 0.011

2457669.83105 8.864 0.011

2457669.83362 8.858 0.010

2457669.83620 8.858 0.011

2457669.83875 8.859 0.010

2457669.84131 8.867 0.010

2457669.84388 8.867 0.010

2457669.84644 8.846 0.010

2457669.84900 8.860 0.010

2457669.85157 8.856 0.010

2457669.85414 8.854 0.010

2457669.85671 8.860 0.010

2457669.85927 8.860 0.010

2457669.86182 8.861 0.010

2457669.86440 8.840 0.010

2457669.86714 8.858 0.010

2457669.86994 8.844 0.010

2457669.87271 8.843 0.009

2457669.87546 8.834 0.010

2457670.75265 8.767 0.025

2457670.75630 8.747 0.022

2457670.75997 8.718 0.020

2457670.76362 8.764 0.019

2457670.76727 8.780 0.017

2457670.77095 8.759 0.016

2457670.77461 8.727 0.016

2457670.77826 8.711 0.015

2457670.78191 8.749 0.014

2457670.78558 8.731 0.013

2457670.78924 8.757 0.013

2457670.79289 8.757 0.013

2457670.79654 8.750 0.012

2457670.80020 8.733 0.012

2457670.80385 8.775 0.012

2457670.80752 8.760 0.011

2457670.81117 8.751 0.012

2457670.81483 8.740 0.011

2457670.81850 8.766 0.011

2457670.82215 8.747 0.011

2457670.82582 8.785 0.011

2457670.82949 8.742 0.011

2457670.83317 8.740 0.014

2457670.83694 8.737 0.010

2457670.84073 8.737 0.010

2457670.84451 8.726 0.010

2457670.84832 8.747 0.010

2457670.85212 8.736 0.010

2457670.85590 8.735 0.011

2457670.85968 8.752 0.010

2457670.86344 8.734 0.010

2457670.86721 8.747 0.009

2457670.87097 8.751 0.009

2457670.87476 8.749 0.010

2457671.74877 8.732 0.025

2457671.75425 8.761 0.021

2457671.75697 8.779 0.020

2457671.75970 8.725 0.019

2457671.76244 8.751 0.017

2457671.76515 8.753 0.017

2457671.77063 8.721 0.016

2457671.77336 8.719 0.015

2457671.77609 8.752 0.015

2457671.77882 8.750 0.014

2457671.78156 8.761 0.013

2457671.78428 8.759 0.013

2457671.78703 8.734 0.013

2457671.78976 8.749 0.012

2457671.79249 8.758 0.012

2457671.79523 8.730 0.012

2457671.79797 8.745 0.012

2457671.80072 8.744 0.011

2457671.80345 8.764 0.011

2457671.80618 8.743 0.011

2457671.80891 8.725 0.011

2457671.81164 8.747 0.011

2457671.81439 8.742 0.011

2457671.81713 8.726 0.011

2457671.81987 8.741 0.011

2457671.82262 8.746 0.011

2457671.82536 8.747 0.011

2457671.82810 8.739 0.010

2457671.83084 8.721 0.010

2457671.83358 8.744 0.010

2457671.83632 8.737 0.010

2457671.83905 8.750 0.010

2457671.84178 8.746 0.010

2457671.84454 8.746 0.010

2457671.84728 8.747 0.010

2457671.85003 8.734 0.010

2457671.85277 8.763 0.010

2457671.85551 8.741 0.010

2457671.85825 8.740 0.010

2457671.86119 8.734 0.010

2457671.86412 8.747 0.010

2457671.86706 8.762 0.009

2457671.87000 8.723 0.010

2457671.87294 8.739 0.009

2457672.74601 8.737 0.025

2457672.74874 8.745 0.024

2457672.75146 8.775 0.021

2457672.75419 8.805 0.020

2457672.75693 8.756 0.019

2457672.75966 8.776 0.018

2457672.76241 8.769 0.017

2457672.76513 8.767 0.016

2457672.76786 8.798 0.016

2457672.77060 8.719 0.015

2457672.77334 8.774 0.014

2457672.77606 8.795 0.014

2457672.77880 8.767 0.014

2457672.78153 8.771 0.013

2457672.78426 8.795 0.013

2457672.78699 8.788 0.013

2457672.78973 8.773 0.012

2457672.79248 8.778 0.012

2457672.79521 8.778 0.012

2457672.79794 8.753 0.011

2457672.80067 8.777 0.011

2457672.80341 8.790 0.011

2457672.80615 8.766 0.011

2457672.80888 8.771 0.011

2457672.81162 8.771 0.011

2457672.81434 8.749 0.011

2457672.81707 8.771 0.011

2457672.81983 8.754 0.011

2457672.82256 8.755 0.010

2457672.82530 8.747 0.010

2457672.82804 8.766 0.010

2457672.83080 8.760 0.010

2457672.83355 8.757 0.010

2457672.83627 8.779 0.010

2457672.83900 8.766 0.010

2457672.84176 8.756 0.010

2457672.84449 8.771 0.010

2457672.84722 8.771 0.010

2457672.84995 8.773 0.010

2457672.85271 8.773 0.010

2457672.85544 8.764 0.010

2457672.85837 8.756 0.010

2457672.86133 8.761 0.009

2457672.86427 8.746 0.010

2457672.86721 8.743 0.010

2457672.87015 8.761 0.010

2457672.87311 8.770 0.009

2457673.74318 8.826 0.025

2457673.74865 8.829 0.022

2457673.75138 8.819 0.020

2457673.75411 8.707 0.020

2457673.75686 8.807 0.018

2457673.75961 8.813 0.018

2457673.76234 8.789 0.017

2457673.76508 8.775 0.016

2457673.76780 8.783 0.015

2457673.77054 8.771 0.015

2457673.77328 8.776 0.015

2457673.77602 8.817 0.014

2457673.77876 8.790 0.014

2457673.78150 8.774 0.013

2457673.78425 8.785 0.013

2457673.78698 8.779 0.013

2457673.78971 8.784 0.013

2457673.79245 8.799 0.012

2457673.79519 8.797 0.012

2457673.79791 8.793 0.012

2457673.80065 8.772 0.012

2457673.80340 8.777 0.011

2457673.80613 8.776 0.011

2457673.80885 8.729 0.012

2457673.81162 8.763 0.011

2457673.81434 8.758 0.012

2457673.81708 8.803 0.011

2457673.81984 8.782 0.011

2457673.82531 8.751 0.012

2457673.82804 8.774 0.049

2457673.83078 8.767 0.011

2457673.83354 8.725 0.035

2457673.83628 8.810 0.022

2457673.83900 8.751 0.013

2457673.84172 8.730 0.018

2457673.84447 8.771 0.010

2457673.84722 8.764 0.011

2457673.84995 8.791 0.010

2457673.85269 8.773 0.011

2457673.85564 8.749 0.028

2457673.85859 8.772 0.010

2457673.86150 8.796 0.015

2457673.86447 8.771 0.010

2457673.86741 8.750 0.010

2457673.87035 8.775 0.010

2457673.87332 8.798 0.010

2457674.73910 8.790 0.027

2457674.74184 8.759 0.024

2457674.74457 8.746 0.023

2457674.74731 8.753 0.022

2457674.75278 8.811 0.019

2457674.75550 8.727 0.018

2457674.75824 8.736 0.018

2457674.76096 8.767 0.017

2457674.76369 8.773 0.016

2457674.76642 8.767 0.015

2457674.76914 8.744 0.015

2457674.77189 8.793 0.014

2457674.77461 8.764 0.014

2457674.77735 8.786 0.014

2457674.78008 8.740 0.013

2457674.78282 8.761 0.013

2457674.78557 8.794 0.012

2457674.78831 8.767 0.013

2457674.79104 8.746 0.012

2457674.79376 8.755 0.012

2457674.79649 8.758 0.012

2457674.79921 8.766 0.012

2457674.80194 8.778 0.012

2457674.80469 8.767 0.011

2457674.80743 8.753 0.011

2457674.81017 8.763 0.011

2457674.81292 8.760 0.011

2457674.81565 8.734 0.011

2457674.81838 8.761 0.011

2457674.82111 8.763 0.010

2457674.82384 8.765 0.011

2457674.82655 8.756 0.010

2457674.82927 8.752 0.010

2457674.83201 8.755 0.010

2457674.83475 8.727 0.010

2457674.83749 8.762 0.010

2457674.84022 8.728 0.010

2457674.84295 8.758 0.010

2457674.84569 8.751 0.010

2457674.84846 8.741 0.010

2457674.85119 8.758 0.009

2457674.85412 8.741 0.010

2457674.85709 8.777 0.009

2457674.86002 8.757 0.010

2457674.86297 8.747 0.010

2457674.86589 8.759 0.010

2457674.86884 8.749 0.009

2457674.87177 8.748 0.009

2457675.73568 8.808 0.028

2457675.73778 8.745 0.027

2457675.73990 8.766 0.025

2457675.74201 8.796 0.023

2457675.74410 8.766 0.023

2457675.74619 8.735 0.021

2457675.74830 8.744 0.020

2457675.75041 8.758 0.019

2457675.75250 8.772 0.018

2457675.75459 8.773 0.018

2457675.75669 8.710 0.018

2457675.75878 8.725 0.018

2457675.76089 8.762 0.016

2457675.76301 8.728 0.016

2457675.76512 8.774 0.015

2457675.76721 8.750 0.015

2457675.76931 8.760 0.014

2457675.77140 8.775 0.014

2457675.77352 8.718 0.014

2457675.77564 8.768 0.013

2457675.77777 8.748 0.014

2457675.77988 8.743 0.013

2457675.78199 8.753 0.013

2457675.78410 8.767 0.012

2457675.78619 8.724 0.013

2457675.78829 8.758 0.013

2457675.79038 8.754 0.012

2457675.79249 8.733 0.012

2457675.79458 8.753 0.012

2457675.79668 8.716 0.012

2457675.79880 8.756 0.012

2457675.80091 8.747 0.012

2457675.80303 8.737 0.012

2457675.80512 8.753 0.012

2457675.80721 8.758 0.012

2457675.80931 8.737 0.012

2457675.81140 8.728 0.011

2457675.81350 8.727 0.011

2457675.81559 8.752 0.011

2457675.81770 8.722 0.011

2457675.81980 8.766 0.011

2457675.82192 8.737 0.011

2457675.82404 8.718 0.010

2457675.82616 8.743 0.010

2457675.82829 8.727 0.011

2457675.83043 8.737 0.010

2457675.83255 8.714 0.010

2457675.83468 8.737 0.010

2457675.83678 8.753 0.010

2457675.83888 8.741 0.010

2457675.84097 8.729 0.010

2457675.84307 8.735 0.010

2457675.84516 8.721 0.010

2457675.84726 8.727 0.010

2457675.84955 8.738 0.010

2457675.85181 8.747 0.010

2457675.85406 8.717 0.010

2457675.85631 8.735 0.010

2457675.85856 8.735 0.010

2457675.86083 8.737 0.013

2457675.86308 8.732 0.010

2457675.86537 8.732 0.013

2457675.86762 8.718 0.009

2457675.86990 8.742 0.013

2457675.87214 8.725 0.010

2457676.73495 8.777 0.027

2457676.73706 8.747 0.025

2457676.73917 8.789 0.023

2457676.74126 8.797 0.022

2457676.74546 8.781 0.021

2457676.74758 8.808 0.046

2457676.74969 8.805 0.018

2457676.75179 8.731 0.018

2457676.75390 8.786 0.017

2457676.75601 8.776 0.018

2457676.75810 8.765 0.016

2457676.76022 8.728 0.017

2457676.76233 8.768 0.015

2457676.76444 8.746 0.015

2457676.76654 8.789 0.015

2457676.76865 8.759 0.016

2457676.77076 8.772 0.014

2457676.77288 8.771 0.015

2457676.77497 8.767 0.016

2457676.77707 8.788 0.018

2457676.77918 8.742 0.014

2457676.78128 8.750 0.013

2457676.78339 8.748 0.013

2457676.78549 8.741 0.013

2457676.78759 8.735 0.013

2457676.78971 8.753 0.012

2457676.79182 8.748 0.012

2457676.79390 8.727 0.012

2457676.79602 8.765 0.012

2457676.79813 8.744 0.012

2457676.80022 8.748 0.012

2457676.80230 8.782 0.012

2457676.80442 8.762 0.012

2457676.80653 8.764 0.011

2457676.80863 8.732 0.012

2457676.81072 8.757 0.013

2457676.81281 8.744 0.011

2457676.81493 8.749 0.012

2457676.81705 8.765 0.012

2457676.81916 8.780 0.015

2457676.82126 8.814 0.011

2457676.82337 8.732 0.011

2457676.82546 8.761 0.012

2457676.82755 8.753 0.011

2457676.82964 8.756 0.013

2457676.83174 8.787 0.013

2457676.83384 8.784 0.011

2457676.83595 8.781 0.011

2457676.83806 8.799 0.012

2457676.84017 8.776 0.017

2457676.84228 8.787 0.010

2457676.84440 8.793 0.010

2457676.84668 8.785 0.010

2457676.84892 8.794 0.011

2457676.85118 8.828 0.013

2457676.85344 8.827 0.010

2457676.85568 8.819 0.014

2457676.85794 8.818 0.013

2457676.86020 8.839 0.012

2457676.86247 8.831 0.012

2457676.86471 8.823 0.022

2457676.86924 8.844 0.014

2457676.87149 8.854 0.011

2457677.78097 8.759 0.013

2457677.78307 8.761 0.013

2457677.78517 8.774 0.013

2457677.78729 8.767 0.013

2457677.78940 8.750 0.013

2457677.79150 8.773 0.012

2457677.79360 8.761 0.012

2457677.79569 8.752 0.012

2457677.79780 8.803 0.012

2457677.79991 8.790 0.012

2457677.80203 8.735 0.012

2457677.80414 8.763 0.012

2457677.80625 8.783 0.011

2457677.80837 8.780 0.012

2457677.81046 8.755 0.012

2457677.81256 8.765 0.011

2457677.81465 8.765 0.011

2457677.81675 8.770 0.011

2457677.81884 8.771 0.011

2457677.82094 8.779 0.011

2457677.82304 8.756 0.011

2457677.82516 8.754 0.011

2457677.82729 8.774 0.010

2457677.82940 8.783 0.011

2457677.83150 8.783 0.011

2457677.83361 8.766 0.011

2457677.83573 8.769 0.011

2457677.83785 8.789 0.011

2457677.83995 8.770 0.011

2457677.84206 8.776 0.010

2457677.84433 8.758 0.011

2457677.84657 8.777 0.011

2457677.84884 8.779 0.010

2457677.85110 8.754 0.010

2457677.85334 8.760 0.010

2457677.85561 8.771 0.010

2457677.85786 8.771 0.010

2457677.86014 8.756 0.010

2457677.86240 8.755 0.010

2457677.86466 8.774 0.010

2457677.86692 8.761 0.010

2457677.86917 8.769 0.010

2457677.87142 8.759 0.010

2457678.77825 8.805 0.012

2457678.78035 8.773 0.013

2457678.78247 8.755 0.013

2457678.78458 8.769 0.012

2457678.78669 8.789 0.012

2457678.78881 8.808 0.012

2457678.79089 8.790 0.012

2457678.79300 8.781 0.012

2457678.79509 8.786 0.012

2457678.79719 8.777 0.012

2457678.79928 8.770 0.011

2457678.80139 8.786 0.011

2457678.80351 8.784 0.011

2457678.80560 8.769 0.011

2457678.80771 8.782 0.011

2457678.80981 8.783 0.011

2457678.81194 8.780 0.011

2457678.81406 8.756 0.011

2457678.81618 8.767 0.011

2457678.81829 8.760 0.011

2457678.82041 8.760 0.011

2457678.82253 8.766 0.010

2457678.82464 8.755 0.010

2457678.82676 8.786 0.010

2457678.82888 8.777 0.010

2457678.83097 8.792 0.010

2457678.83307 8.779 0.010

2457678.83516 8.773 0.010

2457678.83726 8.796 0.010

2457678.83935 8.766 0.010

2457678.84160 8.765 0.010

2457678.84388 8.761 0.010

2457678.84612 8.760 0.010

2457678.84840 8.772 0.010

2457678.85065 8.762 0.010

2457678.85289 8.763 0.012

2457678.85515 8.757 0.010

2457678.85741 8.764 0.010

2457678.85965 8.755 0.010

2457678.86191 8.740 0.010

2457678.86417 8.763 0.010

2457678.86641 8.756 0.010

2457678.86866 8.790 0.010

2457680.77257 8.742 0.013

2457680.77466 8.730 0.012

2457680.77676 8.778 0.012

2457680.77887 8.740 0.012

2457680.78097 8.755 0.012

2457680.78307 8.769 0.012

2457680.78517 8.742 0.012

2457680.78727 8.756 0.012

2457680.78938 8.742 0.011

2457680.79146 8.739 0.011

2457680.79355 8.721 0.011

2457680.79565 8.734 0.011

2457680.79777 8.749 0.011

2457680.79987 8.735 0.011

2457680.80197 8.717 0.011

2457680.80405 8.737 0.011

2457680.80615 8.742 0.011

2457680.80824 8.751 0.011

2457680.81034 8.726 0.010

2457680.81243 8.733 0.011

2457680.81453 8.736 0.010

2457680.81663 8.741 0.010

2457680.81872 8.720 0.010

2457680.82081 8.737 0.010

2457680.82289 8.732 0.010

2457680.82499 8.719 0.010

2457680.82708 8.765 0.010

2457680.82919 8.744 0.010

2457680.83130 8.740 0.010

2457680.83339 8.741 0.011

2457680.83563 8.734 0.010

2457680.83787 8.723 0.010

2457680.84010 8.715 0.011

2457680.84235 8.763 0.010

2457680.84459 8.750 0.010

2457680.84685 8.742 0.010

2457680.84910 8.723 0.010

2457680.85134 8.727 0.010

2457680.85360 8.728 0.010

2457680.85586 8.758 0.010

2457680.85809 8.744 0.010

2457680.86034 8.729 0.010

2457680.86258 8.730 0.010

2457680.86481 8.735 0.010

2457680.86707 8.744 0.010

2457680.86939 8.729 0.009

2457681.76976 8.956 0.013

2457681.77186 8.974 0.012

2457681.77396 8.977 0.012

2457681.77605 8.954 0.012

2457681.77815 8.923 0.012

2457681.78024 8.947 0.012

2457681.78235 8.946 0.011

2457681.78444 8.909 0.012

2457681.78656 8.926 0.011

2457681.78867 8.889 0.012

2457681.79079 8.909 0.011

2457681.79289 8.896 0.011

2457681.79501 8.900 0.011

2457681.79713 8.894 0.011

2457681.79925 8.866 0.011

2457681.80135 8.863 0.011

2457681.80347 8.869 0.011

2457681.80558 8.858 0.011

2457681.80769 8.843 0.011

2457681.80978 8.845 0.011

2457681.81188 8.848 0.010

2457681.81397 8.810 0.010

2457681.81606 8.832 0.010

2457681.81818 8.832 0.010

2457681.82030 8.823 0.010

2457681.82241 8.824 0.010

2457681.82451 8.809 0.010

2457681.82661 8.770 0.010

2457681.82872 8.818 0.010

2457681.83082 8.783 0.010

2457681.83307 8.798 0.010

2457681.83534 8.799 0.010

2457681.83759 8.776 0.010

2457681.83985 8.764 0.010

2457681.84211 8.771 0.010

2457681.84436 8.757 0.010

2457681.84663 8.771 0.009

2457681.84888 8.786 0.010

2457681.85113 8.753 0.010

2457681.85338 8.752 0.010

2457681.85564 8.756 0.010

2457681.85788 8.768 0.009

2457681.86014 8.755 0.009

2457681.86240 8.781 0.009

2457681.86464 8.745 0.010

2457681.86697 8.749 0.010

2457681.86929 8.750 0.010

2457682.76703 8.769 0.013

2457682.76913 8.795 0.012

2457682.77126 8.760 0.013

2457682.77339 8.766 0.014

2457682.77551 8.767 0.012

2457682.77762 8.752 0.012

2457682.77975 8.772 0.012

2457682.78186 8.786 0.012

2457682.78399 8.772 0.012

2457682.78610 8.785 0.012

2457682.78824 8.749 0.012

2457682.79035 8.770 0.013

2457682.79248 8.768 0.018

2457682.79461 8.770 0.014

2457682.79674 8.775 0.013

2457682.79887 8.780 0.012

2457682.80096 8.801 0.012

2457682.80311 8.784 0.014

2457682.80521 8.781 0.012

2457682.80733 8.753 0.011

2457682.80943 8.756 0.011

2457682.81155 8.745 0.012

2457682.81368 8.743 0.011

2457682.81580 8.767 0.012

2457682.81794 8.786 0.012

2457682.82007 8.760 0.013

2457682.82220 8.808 0.011

2457682.82433 8.778 0.011

2457682.82645 8.761 0.011

2457682.82855 8.779 0.012

2457682.83082 8.763 0.012

2457682.83310 8.778 0.010

2457682.83538 8.772 0.011

2457682.83769 8.758 0.011

2457682.83994 8.764 0.010

2457682.84221 8.755 0.012

2457682.84446 8.775 0.011

2457682.84670 8.749 0.010

2457682.84897 8.759 0.010

2457682.85125 8.777 0.009

2457682.85351 8.760 0.010

2457682.85580 8.770 0.010

2457682.85806 8.751 0.010

2457682.86031 8.757 0.010

2457682.86258 8.762 0.010

2457682.86493 8.754 0.010

2457682.86727 8.767 0.010

2457683.76425 8.775 0.012

2457683.76699 8.781 0.012

2457683.76975 8.766 0.012

2457683.77251 8.771 0.012

2457683.77524 8.747 0.011

2457683.77803 8.773 0.012

2457683.78076 8.775 0.011

2457683.78355 8.772 0.011

2457683.78630 8.777 0.011

2457683.78905 8.754 0.011

2457683.79181 8.765 0.011

2457683.79458 8.754 0.011

2457683.79735 8.774 0.011

2457683.80010 8.767 0.011

2457683.80287 8.762 0.011

2457683.80563 8.765 0.011

2457683.80837 8.783 0.010

2457683.81113 8.759 0.011

2457683.81388 8.759 0.010

2457683.81662 8.760 0.010

2457683.81939 8.745 0.010

2457683.82216 8.758 0.010

2457683.82494 8.755 0.010

2457683.82789 8.764 0.010

2457683.83080 8.766 0.010

2457683.83370 8.749 0.010

2457683.83661 8.767 0.010

2457683.83951 8.755 0.010

2457683.84245 8.757 0.010

2457683.84536 8.744 0.010

2457683.84826 8.778 0.010

2457683.85116 8.770 0.010

2457683.85407 8.753 0.010

2457683.85699 8.770 0.010

2457683.85990 8.769 0.009

2457683.86286 8.761 0.010

2457683.86583 8.766 0.009

2457684.76160 8.763 0.012

2457684.76433 8.740 0.012

2457684.76707 8.724 0.012

2457684.76981 8.764 0.012

2457684.77257 8.766 0.012

2457684.77530 8.718 0.012

2457684.77804 8.744 0.011

2457684.78081 8.747 0.011

2457684.78356 8.736 0.011

2457684.78631 8.745 0.011

2457684.78905 8.763 0.011

2457684.79182 8.723 0.010

2457684.79458 8.744 0.011

2457684.79731 8.753 0.011

2457684.80006 8.737 0.010

2457684.80281 8.730 0.010

2457684.80556 8.738 0.010

2457684.80831 8.738 0.010

2457684.81105 8.750 0.010

2457684.81380 8.733 0.010

2457684.81654 8.741 0.010

2457684.81929 8.742 0.010

2457684.82204 8.763 0.010

2457684.82497 8.760 0.010

2457684.82788 8.730 0.010

2457684.83080 8.760 0.010

2457684.83374 8.762 0.010

2457684.83664 8.762 0.010

2457684.83957 8.741 0.010

2457684.84248 8.745 0.010

2457684.84541 8.728 0.010

2457684.84832 8.754 0.010

2457684.85126 8.746 0.010

2457684.85418 8.757 0.010

2457684.85712 8.745 0.010

2457684.86010 8.745 0.009

2457684.86310 8.742 0.010

2457684.86610 8.738 0.010

2457685.75965 8.737 0.012

2457685.76269 8.748 0.012

2457685.76572 8.729 0.012

2457685.76876 8.714 0.011

2457685.77178 8.730 0.011

2457685.77484 8.741 0.011

2457685.77791 8.709 0.011

2457685.78095 8.747 0.011

2457685.78398 8.740 0.011

2457685.78704 8.738 0.011

2457685.79007 8.750 0.010

2457685.79318 8.737 0.010

2457685.79630 8.736 0.010

2457685.79938 8.731 0.010

2457685.80245 8.712 0.010

2457685.80553 8.752 0.010

2457685.80863 8.720 0.010

2457685.81175 8.727 0.010

2457685.81484 8.726 0.010

2457685.81794 8.735 0.010

2457685.82105 8.717 0.010

2457685.82416 8.729 0.010

2457685.82723 8.719 0.010

2457685.83032 8.716 0.010

2457685.83343 8.735 0.009

2457685.83653 8.743 0.010

2457685.83962 8.752 0.009

2457685.84272 8.714 0.009

2457685.84582 8.724 0.010

2457685.84889 8.740 0.009

2457685.85200 8.719 0.009

2457685.85512 8.740 0.010

2457685.85829 8.750 0.009

2457685.86145 8.741 0.009

2457685.86459 8.736 0.009

2457686.75669 8.764 0.012

2457686.75948 8.757 0.012

2457686.76229 8.750 0.011

2457686.76508 8.748 0.017

2457686.76788 8.769 0.011

2457686.77068 8.753 0.011

2457686.77347 8.787 0.011

2457686.77625 8.757 0.011

2457686.77928 8.760 0.011

2457686.78234 8.762 0.011

2457686.78537 8.742 0.010

2457686.78839 8.756 0.010

2457686.79141 8.758 0.010

2457686.79443 8.762 0.010

2457686.79745 8.761 0.010

2457686.80047 8.754 0.010

2457686.80351 8.763 0.010

2457686.80655 8.766 0.010

2457686.80958 8.751 0.010

2457686.81262 8.752 0.010

2457686.81564 8.757 0.010

2457686.81867 8.743 0.010

2457686.82168 8.770 0.009

2457686.82469 8.762 0.010

2457686.82770 8.759 0.010

2457686.83072 8.751 0.010

2457686.83375 8.752 0.010

2457686.83676 8.769 0.010

2457686.83976 8.766 0.009

2457686.84278 8.758 0.010

2457686.84578 8.753 0.010

2457686.84881 8.759 0.009

2457686.85182 8.745 0.009

2457686.85484 8.754 0.009

2457686.85789 8.742 0.009

2457686.86094 8.757 0.010

2457686.86395 8.748 0.009

2457687.75383 8.800 0.012

2457687.75662 8.782 0.011

2457687.75942 8.773 0.012

2457687.76221 8.752 0.012

2457687.76501 8.748 0.012

2457687.76780 8.759 0.011

2457687.77059 8.800 0.011

2457687.77341 8.772 0.011

2457687.77644 8.764 0.011

2457687.77947 8.782 0.011

2457687.78248 8.776 0.011

2457687.78552 8.778 0.010

2457687.78854 8.788 0.011

2457687.79159 8.768 0.010

2457687.79462 8.763 0.010

2457687.79764 8.777 0.010

2457687.80068 8.772 0.010

2457687.80372 8.762 0.010

2457687.80675 8.761 0.010

2457687.80977 8.776 0.010

2457687.81280 8.742 0.010

2457687.81583 8.776 0.010

2457687.81884 8.767 0.010

2457687.82186 8.762 0.010

2457687.82488 8.778 0.010

2457687.82788 8.756 0.010

2457687.83090 8.777 0.009

2457687.83391 8.771 0.009

2457687.83693 8.768 0.010

2457687.83995 8.773 0.009

2457687.84297 8.781 0.009

2457687.84600 8.780 0.009

2457687.84902 8.784 0.009

2457687.85200 8.770 0.009

2457687.85500 8.757 0.009

2457687.85801 8.780 0.009

2457687.86101 8.776 0.009

2457687.86399 8.764 0.009

2457688.75115 8.808 0.012

2457688.75395 8.808 0.012

2457688.75674 8.822 0.012

2457688.75951 8.842 0.011

2457688.76231 8.857 0.011

2457688.76509 8.845 0.011

2457688.76789 8.814 0.011

2457688.77069 8.836 0.011

2457688.77374 8.831 0.011

2457688.77677 8.837 0.011

2457688.77983 8.824 0.011

2457688.78286 8.866 0.010

2457688.78588 8.848 0.011

2457688.78890 8.840 0.011

2457688.79193 8.847 0.010

2457688.79494 8.859 0.010

2457688.79800 8.855 0.010

2457688.80102 8.857 0.010

2457688.80404 8.842 0.010

2457688.80707 8.841 0.010

2457688.81012 8.857 0.010

2457688.81314 8.848 0.010

2457688.81615 8.848 0.010

2457688.81917 8.862 0.010

2457688.82219 8.845 0.010

2457688.82519 8.857 0.010

2457688.82819 8.858 0.010

2457688.83122 8.840 0.010

2457688.83422 8.854 0.010

2457688.83722 8.860 0.009

2457688.84025 8.839 0.010

2457688.84328 8.865 0.009

2457688.84631 8.867 0.010

2457688.84933 8.857 0.010

2457688.85234 8.858 0.009

2457688.85536 8.857 0.009

2457688.85837 8.848 0.010

2457688.86138 8.852 0.009

2457688.86440 8.866 0.009

2457689.74948 8.783 0.012

2457689.75343 8.764 0.012

2457689.75738 8.748 0.012

2457689.76134 8.744 0.012

2457689.76530 8.742 0.011

2457689.76924 8.751 0.011

2457689.77344 8.757 0.011

2457689.77763 8.754 0.011

2457689.78184 8.742 0.011

2457689.78586 8.721 0.011

2457689.78986 8.728 0.011

2457689.79388 8.752 0.010

2457689.79788 8.751 0.010

2457689.80192 8.722 0.010

2457689.80595 8.746 0.010

2457689.80998 8.753 0.010

2457689.81398 8.716 0.010

2457689.81800 8.722 0.010

2457689.82200 8.735 0.010

2457689.82602 8.758 0.010

2457689.83003 8.733 0.010

2457689.83407 8.741 0.010

2457689.83810 8.734 0.010

2457689.84212 8.748 0.010

2457689.84616 8.751 0.010

2457689.85019 8.750 0.010

2457689.85424 8.729 0.010

2457689.85826 8.758 0.009

2457689.86228 8.733 0.009

2457690.74604 8.749 0.012

2457690.74916 8.737 0.012

2457690.75229 8.742 0.012

2457690.75543 8.704 0.012

2457690.75858 8.738 0.011

2457690.76171 8.728 0.012

2457690.76484 8.744 0.011

2457690.76818 8.730 0.011

2457690.77154 8.757 0.011

2457690.77491 8.756 0.011

2457690.77826 8.728 0.011

2457690.78149 8.734 0.011

2457690.78466 8.728 0.011

2457690.78785 8.731 0.010

2457690.79106 8.754 0.011

2457690.79424 8.728 0.010

2457690.79743 8.754 0.010

2457690.80065 8.732 0.010

2457690.80381 8.755 0.010

2457690.80703 8.732 0.010

2457690.81020 8.735 0.010

2457690.81340 8.740 0.010

2457690.81657 8.719 0.010

2457690.81977 8.734 0.010

2457690.82294 8.747 0.010

2457690.82617 8.746 0.010

2457690.82933 8.739 0.010

2457690.83256 8.746 0.010

2457690.83575 8.718 0.010

2457690.83891 8.737 0.010

2457690.84212 8.750 0.009

2457690.84534 8.723 0.010

2457690.84850 8.748 0.010

2457690.85171 8.734 0.010

2457690.85493 8.735 0.010

2457690.85815 8.745 0.009

2457690.86139 8.724 0.010

2457691.74328 8.781 0.013

2457691.74642 8.783 0.012

2457691.74955 8.769 0.012

2457691.75267 8.768 0.012

2457691.75581 8.763 0.011

2457691.75895 8.783 0.011

2457691.76208 8.757 0.011

2457691.76544 8.764 0.011

2457691.76880 8.752 0.011

2457691.77214 8.759 0.010

2457691.77551 8.776 0.010

2457691.77869 8.744 0.011

2457691.78191 8.784 0.010

2457691.78513 8.737 0.011

2457691.78830 8.792 0.010

2457691.79148 8.779 0.010

2457691.79469 8.763 0.010

2457691.79786 8.768 0.010

2457691.80106 8.752 0.010

2457691.80426 8.755 0.010

2457691.80744 8.756 0.010

2457691.81061 8.766 0.010

2457691.81382 8.765 0.010

2457691.81700 8.759 0.010

2457691.82017 8.788 0.010

2457691.82338 8.761 0.010

2457691.82657 8.749 0.010

2457691.82978 8.751 0.010

2457691.83295 8.758 0.010

2457691.83615 8.774 0.010

2457691.83936 8.740 0.010

2457691.84255 8.760 0.009

2457691.84572 8.756 0.009

2457691.84892 8.754 0.010

2457691.85213 8.749 0.010

2457691.85532 8.762 0.009

2457691.85852 8.759 0.010

2457691.86169 8.753 0.009

2457692.74046 8.774 0.012

2457692.74361 8.762 0.012

2457692.74671 8.769 0.012

2457692.74983 8.768 0.012

2457692.75296 8.769 0.011

2457692.75606 8.788 0.011

2457692.75919 8.786 0.011

2457692.76256 8.762 0.011

2457692.76594 8.794 0.011

2457692.76928 8.750 0.011

2457692.77265 8.746 0.011

2457692.77582 8.762 0.011

2457692.77905 8.772 0.010

2457692.78222 8.752 0.011

2457692.78542 8.775 0.010

2457692.78859 8.758 0.010

2457692.79177 8.754 0.010

2457692.79498 8.762 0.010

2457692.79815 8.780 0.010

2457692.80134 8.778 0.010

2457692.80454 8.778 0.010

2457692.80772 8.773 0.010

2457692.81093 8.775 0.010

2457692.81409 8.792 0.010

2457692.81727 8.767 0.010

2457692.82047 8.768 0.010

2457692.82367 8.765 0.010

2457692.82685 8.780 0.009

2457692.83006 8.777 0.009

2457692.83325 8.771 0.010

2457692.83645 8.768 0.010

2457692.83965 8.776 0.009

2457692.84284 8.780 0.009

2457692.84601 8.777 0.009

2457692.84921 8.763 0.009

2457692.85242 8.760 0.009

2457692.85561 8.767 0.009

2457692.85877 8.761 0.009

2457692.86197 8.766 0.009

2457693.73771 8.777 0.012

2457693.74086 8.746 0.012

2457693.74402 8.752 0.011

2457693.74715 8.748 0.011

2457693.75029 8.743 0.011

2457693.75343 8.750 0.011

2457693.75657 8.742 0.011

2457693.75998 8.757 0.011

2457693.76334 8.759 0.011

2457693.76674 8.755 0.010

2457693.77013 8.748 0.010

2457693.77334 8.764 0.010

2457693.77655 8.743 0.010

2457693.77977 8.752 0.010

2457693.78297 8.753 0.011

2457693.78619 8.748 0.010

2457693.78941 8.766 0.010

2457693.79262 8.769 0.010

2457693.79583 8.748 0.010

2457693.79903 8.760 0.010

2457693.80225 8.752 0.010

2457693.80547 8.765 0.010

2457693.80872 8.751 0.010

2457693.81192 8.754 0.010

2457693.81513 8.748 0.010

2457693.81836 8.750 0.010

2457693.82156 8.760 0.010

2457693.82478 8.768 0.009

2457693.82800 8.757 0.010

2457693.83123 8.762 0.010

2457693.83444 8.748 0.010

2457693.83767 8.739 0.010

2457693.84089 8.738 0.010

2457693.84410 8.737 0.010

2457693.84730 8.749 0.010

2457693.85053 8.753 0.009

2457693.85375 8.756 0.010

2457693.85697 8.755 0.009

2457693.86019 8.756 0.009

2457694.73495 8.742 0.012

2457694.73810 8.753 0.012

2457694.74126 8.780 0.012

2457694.74442 8.763 0.012

2457694.74757 8.739 0.012

2457694.75071 8.752 0.011

2457694.75385 8.739 0.011

2457694.75722 8.737 0.011

2457694.76060 8.748 0.011

2457694.76402 8.745 0.011

2457694.76741 8.718 0.011

2457694.77063 8.750 0.011

2457694.77384 8.747 0.010

2457694.77704 8.732 0.011

2457694.78023 8.717 0.010

2457694.78345 8.730 0.011

2457694.78667 8.739 0.010

2457694.78987 8.747 0.010

2457694.79310 8.753 0.010

2457694.79631 8.729 0.010

2457694.79953 8.730 0.010

2457694.80274 8.744 0.010

2457694.80595 8.739 0.010

2457694.80917 8.749 0.010

2457694.81240 8.715 0.010

2457694.81561 8.739 0.010

2457694.81884 8.749 0.009

2457694.82205 8.735 0.010

2457694.82525 8.742 0.010

2457694.82847 8.728 0.010

2457694.83169 8.736 0.010

2457694.83492 8.716 0.010

2457694.83814 8.733 0.010

2457694.84134 8.742 0.009

2457694.84457 8.718 0.010

2457694.84779 8.730 0.009

2457694.85101 8.750 0.010

2457694.85424 8.733 0.009

2457694.85745 8.733 0.009

2457694.86069 8.752 0.009

2457695.73205 8.714 0.012

2457695.73521 8.762 0.012

2457695.73837 8.735 0.012

2457695.74148 8.759 0.011

2457695.74464 8.734 0.011

2457695.74777 8.735 0.011

2457695.75088 8.753 0.011

2457695.75424 8.740 0.011

2457695.75762 8.748 0.011

2457695.76096 8.737 0.011

2457695.76435 8.747 0.011

2457695.76753 8.729 0.010

2457695.77072 8.721 0.011

2457695.77390 8.741 0.010

2457695.77707 8.730 0.010

2457695.78025 8.756 0.010

2457695.78344 8.742 0.010

2457695.78662 8.751 0.010

2457695.78981 8.744 0.010

2457695.79303 8.755 0.010

2457695.79619 8.733 0.010

2457695.79938 8.757 0.010

2457695.80258 8.751 0.010

2457695.80576 8.755 0.010

2457695.80898 8.746 0.010

2457695.81215 8.742 0.010

2457695.81534 8.737 0.010

2457695.81855 8.749 0.010

2457695.82172 8.763 0.010

2457695.82491 8.745 0.010

2457695.82808 8.766 0.010

2457695.83128 8.772 0.010

2457695.83450 8.770 0.010

2457695.83772 8.781 0.010

2457695.84088 8.780 0.010

2457695.84409 8.787 0.009

2457695.84727 8.791 0.010

2457695.85046 8.809 0.009

2457695.85368 8.797 0.009

2457695.85690 8.819 0.009

2457695.86010 8.824 0.010

2457696.72926 8.766 0.013

2457696.73245 8.757 0.013

2457696.73563 8.739 0.012

2457696.73878 8.783 0.012

2457696.74192 8.761 0.012

2457696.74505 8.757 0.012

2457696.74821 8.786 0.012

2457696.75157 8.754 0.011

2457696.75493 8.792 0.011

2457696.75829 8.793 0.011

2457696.76164 8.781 0.011

2457696.76486 8.776 0.011

2457696.76808 8.746 0.010

2457696.77126 8.780 0.010

2457696.77443 8.737 0.011

2457696.77760 8.780 0.010

2457696.78078 8.763 0.010

2457696.78395 8.767 0.010

2457696.78712 8.763 0.010

2457696.79030 8.764 0.010

2457696.79352 8.758 0.011

2457696.79669 8.763 0.010

2457696.79987 8.788 0.010

2457696.80309 8.783 0.011

2457696.80627 8.752 0.010

2457696.80946 8.767 0.010

2457696.81265 8.748 0.010

2457696.81583 8.762 0.010

2457696.81902 8.755 0.010

2457696.82223 8.751 0.010

2457696.82541 8.741 0.010

2457696.82860 8.749 0.010

2457696.83177 8.777 0.010

2457696.83499 8.765 0.010

2457696.83817 8.756 0.010

2457696.84138 8.768 0.010

2457696.84458 8.774 0.009

2457696.84778 8.763 0.009

2457696.85096 8.773 0.010

2457696.85417 8.759 0.010

2457696.85737 8.753 0.010

2457700.71917 9.040 0.013

2457700.72262 9.062 0.013

2457700.72608 9.086 0.012

2457700.72951 9.043 0.012

2457700.73300 9.042 0.011

2457700.73646 9.025 0.011

2457700.73988 9.039 0.011

2457700.74354 8.996 0.011

2457700.74720 9.020 0.011

2457700.75090 9.002 0.011

2457700.75459 8.997 0.010

2457700.75825 8.973 0.011

2457700.76196 8.957 0.010

2457700.76561 8.953 0.010

2457700.76931 8.952 0.011

2457700.77300 8.928 0.010

2457700.77669 8.916 0.011

2457700.78036 8.920 0.010

2457700.78402 8.913 0.010

2457700.78771 8.903 0.010

2457700.79137 8.903 0.010

2457700.79506 8.886 0.010

2457700.79872 8.857 0.010

2457700.80240 8.835 0.010

2457700.80606 8.853 0.010

2457700.80975 8.810 0.010

2457700.81341 8.817 0.009

2457700.81707 8.818 0.010

2457700.82073 8.797 0.010

2457700.82438 8.794 0.010

2457700.82804 8.792 0.010

2457700.83172 8.787 0.010

2457700.83538 8.770 0.009

2457700.83902 8.782 0.010

2457700.84269 8.759 0.010

2457700.84635 8.732 0.010

2457700.85003 8.753 0.010

2457700.85372 8.748 0.009

2457700.85740 8.742 0.010

2457701.71635 8.744 0.013

2457701.71983 8.756 0.012

2457701.72325 8.781 0.012

2457701.72670 8.812 0.012

2457701.73016 8.788 0.012

2457701.73363 8.791 0.011

2457701.73711 8.779 0.011

2457701.74078 8.747 0.011

2457701.74447 8.762 0.011

2457701.74814 8.777 0.010

2457701.75183 8.760 0.011

2457701.75551 8.767 0.011

2457701.75918 8.761 0.011

2457701.76286 8.779 0.010

2457701.76655 8.781 0.010

2457701.77023 8.766 0.010

2457701.77392 8.777 0.010

2457701.77762 8.748 0.010

2457701.78127 8.758 0.010

2457701.78495 8.763 0.010

2457701.78862 8.780 0.010

2457701.79227 8.769 0.010

2457701.79594 8.764 0.010

2457701.79963 8.768 0.010

2457701.80330 8.790 0.010

2457701.80696 8.776 0.010

2457701.81064 8.768 0.010

2457701.81431 8.754 0.010

2457701.81799 8.781 0.010

2457701.82164 8.769 0.010

2457701.82530 8.748 0.010

2457701.82895 8.768 0.010

2457701.83265 8.769 0.010

2457701.83630 8.745 0.010

2457701.83995 8.758 0.010

2457701.84365 8.779 0.010

2457701.84731 8.764 0.010

2457701.85097 8.780 0.010

2457702.71280 8.788 0.013

2457702.71558 8.792 0.013

2457702.71834 8.755 0.013

2457702.72113 8.764 0.012

2457702.72394 8.760 0.012

2457702.72670 8.784 0.012

2457702.72950 8.770 0.012

2457702.73227 8.752 0.012

2457702.73505 8.765 0.012

2457702.73781 8.775 0.011

2457702.74059 8.793 0.011

2457702.74336 8.779 0.011

2457702.74613 8.765 0.011

2457702.74891 8.761 0.011

2457702.75168 8.784 0.011

2457702.75447 8.763 0.011

2457702.75723 8.782 0.011

2457702.76000 8.749 0.011

2457702.76278 8.748 0.011

2457702.76557 8.747 0.011

2457702.76836 8.764 0.010

2457702.77112 8.755 0.010

2457702.77391 8.757 0.010

2457702.77685 8.771 0.010

2457702.77979 8.750 0.010

2457702.78271 8.753 0.010

2457702.78565 8.759 0.010

2457702.78860 8.760 0.010

2457702.79152 8.783 0.010

2457702.79446 8.741 0.010

2457702.79738 8.753 0.010

2457702.80030 8.756 0.010

2457702.80325 8.739 0.010

2457702.80617 8.739 0.010

2457702.80909 8.722 0.010

2457702.81206 8.777 0.010

2457702.81502 8.763 0.010

2457702.81800 8.758 0.010

2457702.82102 8.749 0.010

2457702.82402 8.759 0.010

2457702.82700 8.757 0.010

2457702.83001 8.751 0.010

2457702.83301 8.770 0.010

2457702.83600 8.776 0.010

2457702.83897 8.743 0.010

2457702.84193 8.738 0.010

2457702.84494 8.774 0.010

2457702.84793 8.753 0.010

2457702.85093 8.746 0.010

2457703.71008 8.742 0.028

2457703.71285 8.764 0.030

2457703.71561 8.787 0.076

2457703.71840 8.736 0.014

2457703.72119 8.796 0.023

2457703.72397 8.761 0.035

2457703.72672 8.764 0.030

2457703.73230 8.768 0.019

2457703.73508 8.741 0.024

2457703.73787 8.775 0.029

2457703.74064 8.779 0.024

2457703.74344 8.749 0.025

2457703.74623 8.716 0.024

2457703.74900 8.765 0.024

2457703.75181 8.722 0.016

2457703.75458 8.736 0.017

2457703.75736 8.710 0.022

2457703.76015 8.733 0.019

2457703.76292 8.717 0.014

2457703.76567 8.758 0.013

2457703.76844 8.747 0.013

2457703.77122 8.726 0.012

2457703.77416 8.754 0.014

2457703.77707 8.713 0.017

2457703.78000 8.764 0.017

2457703.78294 8.733 0.025

2457703.78586 8.721 0.024

2457703.78878 8.743 0.012

2457703.79174 8.720 0.011

2457703.79468 8.722 0.011

2457703.79759 8.745 0.011

2457703.80051 8.743 0.010

2457703.80345 8.732 0.010

2457703.80637 8.723 0.016

2457703.80934 8.732 0.017

2457703.81231 8.720 0.014

2457703.81531 8.739 0.012

2457703.81830 8.743 0.011

2457703.82131 8.752 0.013

2457703.82431 8.735 0.014

2457703.82728 8.730 0.012

2457703.83028 8.748 0.011

2457703.83326 8.724 0.012

2457703.83627 8.734 0.012

2457703.83922 8.732 0.011

2457703.84219 8.755 0.011

2457703.84519 8.727 0.011

2457703.84819 8.745 0.011

2457704.70817 8.753 0.013

2457704.71185 8.749 0.013

2457704.71554 8.745 0.013

2457704.71925 8.761 0.012

2457704.72293 8.757 0.012

2457704.72664 8.746 0.012

2457704.73032 8.762 0.012

2457704.73403 8.734 0.012

2457704.73772 8.730 0.012

2457704.74141 8.737 0.011

2457704.74519 8.764 0.011

2457704.74895 8.720 0.011

2457704.75270 8.754 0.011

2457704.75646 8.747 0.011

2457704.76020 8.740 0.011

2457704.76394 8.744 0.011

2457704.76771 8.754 0.010

2457704.77147 8.739 0.011

2457704.77524 8.727 0.010

2457704.77899 8.742 0.010

2457704.78275 8.734 0.010

2457704.78652 8.763 0.010

2457704.79029 8.733 0.010

2457704.79405 8.743 0.010

2457704.79782 8.744 0.010

2457704.80156 8.752 0.010

2457704.80539 8.751 0.010

2457704.80920 8.751 0.010

2457704.81300 8.734 0.010

2457704.81683 8.742 0.010

2457704.82066 8.729 0.010

2457704.82450 8.735 0.010

2457704.82832 8.740 0.010

2457704.83216 8.728 0.010

2457704.83596 8.735 0.010

2457704.83978 8.725 0.009

2457704.84361 8.722 0.010

2457704.84740 8.734 0.010

2457705.70450 8.783 0.014

2457705.70728 8.754 0.014

2457705.71005 8.761 0.013

2457705.71280 8.772 0.013

2457705.71557 8.783 0.013

2457705.71836 8.735 0.013

2457705.72115 8.762 0.013

2457705.72392 8.746 0.012

2457705.72670 8.745 0.012

2457705.72947 8.760 0.012

2457705.73223 8.756 0.012

2457705.73502 8.756 0.011

2457705.73780 8.773 0.012

2457705.74058 8.763 0.011

2457705.74334 8.727 0.012

2457705.74612 8.757 0.012

2457705.74890 8.768 0.011

2457705.75169 8.759 0.011

2457705.75446 8.757 0.011

2457705.75725 8.739 0.011

2457705.76001 8.761 0.011

2457705.76279 8.766 0.011

2457705.76554 8.758 0.011

2457705.76848 8.759 0.011

2457705.77142 8.763 0.011

2457705.77434 8.765 0.011

2457705.77729 8.753 0.011

2457705.78021 8.733 0.010

2457705.78313 8.764 0.010

2457705.78605 8.768 0.010

2457705.78897 8.750 0.011

2457705.79190 8.758 0.010

2457705.79483 8.780 0.010

2457705.79774 8.749 0.010

2457705.80066 8.760 0.010

2457705.80365 8.736 0.010

2457705.80663 8.770 0.010

2457705.80962 8.742 0.010

2457705.81262 8.764 0.010

2457705.81557 8.767 0.010

2457705.81856 8.761 0.010

2457705.82154 8.749 0.010

2457705.82453 8.747 0.010

2457705.82752 8.761 0.010

2457705.83051 8.757 0.010

2457705.83348 8.751 0.010

2457705.83646 8.741 0.010

2457705.83944 8.755 0.010

2457705.84243 8.779 0.010

2457705.84544 8.758 0.010

2457705.84847 8.738 0.010

2457707.69881 8.804 0.014

2457707.70137 8.777 0.014

2457707.70392 8.785 0.014

2457707.70648 8.804 0.013

2457707.70906 8.781 0.013

2457707.71161 8.795 0.013

2457707.71417 8.793 0.012

2457707.71672 8.812 0.012

2457707.71928 8.771 0.012

2457707.72184 8.775 0.013

2457707.72439 8.788 0.012

2457707.72694 8.792 0.013

2457707.72951 8.802 0.012

2457707.73207 8.802 0.012

2457707.73464 8.808 0.012

2457707.73720 8.801 0.012

2457707.73973 8.808 0.012

2457707.74231 8.821 0.012

2457707.74486 8.828 0.011

2457707.74741 8.796 0.011

2457707.74995 8.840 0.011

2457707.75250 8.824 0.011

2457707.75508 8.821 0.011

2457707.75764 8.847 0.011

2457707.76019 8.821 0.011

2457707.76287 8.803 0.012

2457707.76558 8.821 0.011

2457707.76830 8.840 0.011

2457707.77101 8.830 0.011

2457707.77372 8.830 0.011

2457707.77642 8.837 0.011

2457707.77913 8.837 0.010

2457707.78183 8.838 0.011

2457707.78454 8.848 0.011

2457707.78725 8.851 0.010

2457707.78994 8.848 0.011

2457707.79264 8.817 0.011

2457707.79536 8.879 0.010

2457707.79807 8.832 0.011

2457707.80078 8.856 0.010

2457707.80348 8.873 0.010

2457707.80620 8.889 0.011

2457707.80891 8.865 0.011

2457707.81163 8.878 0.011

2457707.81435 8.864 0.010

2457707.81707 8.853 0.010

2457707.81978 8.864 0.010

2457707.82249 8.868 0.010

2457707.82521 8.843 0.011

2457707.82792 8.852 0.011

2457707.83063 8.871 0.010

2457707.83333 8.844 0.010

2457707.83603 8.876 0.010

2457707.83873 8.862 0.010

2457707.84150 8.868 0.010

2457707.84428 8.858 0.010

2457707.84706 8.852 0.010

2457708.69603 8.754 0.014

2457708.69856 8.777 0.014

2457708.70113 8.752 0.014

2457708.70368 8.744 0.014

2457708.70620 8.740 0.014

2457708.70876 8.776 0.013

2457708.71130 8.768 0.013

2457708.71382 8.785 0.013

2457708.71640 8.768 0.013

2457708.71898 8.751 0.013

2457708.72153 8.728 0.012

2457708.72409 8.745 0.012

2457708.72664 8.739 0.012

2457708.72921 8.764 0.012

2457708.73177 8.771 0.011

2457708.73432 8.751 0.012

2457708.73689 8.731 0.012

2457708.73947 8.752 0.011

2457708.74203 8.765 0.012

2457708.74455 8.742 0.012

2457708.74711 8.738 0.012

2457708.74963 8.757 0.011

2457708.75219 8.749 0.011

2457708.75472 8.743 0.011

2457708.75726 8.745 0.011

2457708.75995 8.731 0.012

2457708.76265 8.737 0.011

2457708.76536 8.740 0.011

2457708.76807 8.739 0.011

2457708.77078 8.711 0.011

2457708.77348 8.734 0.011

2457708.77617 8.731 0.011

2457708.77889 8.735 0.011

2457708.78161 8.743 0.011

2457708.78433 8.745 0.010

2457708.78703 8.746 0.010

2457708.78971 8.742 0.010

2457708.79240 8.755 0.011

2457708.79510 8.745 0.011

2457708.79782 8.739 0.011

2457708.80054 8.746 0.011

2457708.80326 8.740 0.011

2457708.80598 8.760 0.011

2457708.80870 8.756 0.012

2457708.81142 8.756 0.012

2457708.81413 8.738 0.012

2457708.81684 8.746 0.011

2457708.81955 8.755 0.011

2457708.82226 8.749 0.011

2457708.82497 8.740 0.011

2457708.82767 8.753 0.011

2457708.83039 8.748 0.011

2457708.83308 8.744 0.010

2457708.83579 8.737 0.011

2457708.83853 8.723 0.010

2457708.84130 8.735 0.011

2457708.84407 8.748 0.010

2457708.84684 8.722 0.010

2457709.69328 8.728 0.014

2457709.69583 8.759 0.013

2457709.69837 8.747 0.013

2457709.70091 8.760 0.013

2457709.70346 8.749 0.013

2457709.70601 8.772 0.013

2457709.70855 8.761 0.012

2457709.71112 8.738 0.012

2457709.71367 8.787 0.012

2457709.71622 8.733 0.012

2457709.71877 8.755 0.012

2457709.72133 8.743 0.011

2457709.72389 8.764 0.012

2457709.72645 8.758 0.011

2457709.72899 8.762 0.011

2457709.73157 8.759 0.012

2457709.73414 8.763 0.011

2457709.73670 8.749 0.011

2457709.73924 8.744 0.011

2457709.74179 8.724 0.011

2457709.74432 8.735 0.011

2457709.74688 8.744 0.011

2457709.74941 8.723 0.011

2457709.75197 8.746 0.011

2457709.75450 8.749 0.011

2457709.75722 8.760 0.010

2457709.75993 8.749 0.011

2457709.76260 8.729 0.011

2457709.76532 8.725 0.011

2457709.76802 8.759 0.011

2457709.77074 8.704 0.011

2457709.77345 8.718 0.011

2457709.77616 8.747 0.010

2457709.77888 8.753 0.010

2457709.78159 8.747 0.010

2457709.78429 8.744 0.010

2457709.78700 8.759 0.010

2457709.78971 8.726 0.010

2457709.79242 8.709 0.010

2457709.79513 8.733 0.010

2457709.79780 8.742 0.010

2457709.80049 8.734 0.011

2457709.80317 8.731 0.010

2457709.80587 8.739 0.010

2457709.80858 8.734 0.010

2457709.81128 8.748 0.010

2457709.81399 8.742 0.010

2457709.81668 8.714 0.010

2457709.81938 8.722 0.011

2457709.82208 8.736 0.010

2457709.82479 8.734 0.010

2457709.82751 8.729 0.011

2457709.83020 8.750 0.011

2457709.83289 8.751 0.010

2457709.83567 8.734 0.010

2457709.83844 8.729 0.010

2457709.84122 8.732 0.010

2457709.84399 8.720 0.010

2457709.84678 8.734 0.010

2457710.69073 8.751 0.015

2457710.69340 8.774 0.015

2457710.69608 8.794 0.019

2457710.69876 8.760 0.021

2457710.70142 8.763 0.018

2457710.70407 8.760 0.024

2457710.70941 8.791 0.019

2457710.71205 8.789 0.026

2457710.71470 8.792 0.020

2457710.71737 8.756 0.017

2457710.72005 8.752 0.019

2457710.72271 8.769 0.016

2457710.72536 8.766 0.015

2457710.72802 8.766 0.014

2457710.73069 8.761 0.016

2457710.73337 8.709 0.014

2457710.73602 8.764 0.016

2457710.73869 8.768 0.015

2457710.74135 8.734 0.016

2457710.74399 8.752 0.016

2457710.74664 8.774 0.014

2457710.74929 8.776 0.013

2457710.75194 8.764 0.014

2457710.75461 8.791 0.016

2457710.75726 8.759 0.015

2457710.75991 8.750 0.016

2457710.76256 8.761 0.014

2457710.76520 8.767 0.014

2457710.76785 8.752 0.014

2457710.77050 8.761 0.016

2457710.77314 8.763 0.016

2457710.77579 8.741 0.018

2457710.77841 8.782 0.013

2457710.78106 8.749 0.014

2457710.78370 8.763 0.013

2457710.78637 8.781 0.013

2457710.78900 8.757 0.013

2457710.79164 8.756 0.013

2457710.79429 8.737 0.012

2457710.79696 8.740 0.011

2457710.79961 8.747 0.011

2457710.80225 8.769 0.011

2457710.80491 8.731 0.011

2457710.80756 8.766 0.011

2457710.81021 8.759 0.010

2457710.81286 8.758 0.010

2457710.81550 8.766 0.010

2457710.81841 8.758 0.010

2457710.82135 8.737 0.011

2457710.82429 8.748 0.011

2457710.82723 8.771 0.010

2457710.83017 8.775 0.010

2457710.83311 8.760 0.010

2457710.83605 8.775 0.010

2457710.83900 8.745 0.010

2457710.84198 8.749 0.010

2457710.84478 8.771 0.010

2457710.84744 8.760 0.010

2457711.68719 8.788 0.014

2457711.68940 8.771 0.014

2457711.69160 8.789 0.014

2457711.69380 8.794 0.014

2457711.69598 8.801 0.014

2457711.69817 8.769 0.014

2457711.70037 8.769 0.014

2457711.70259 8.758 0.013

2457711.70480 8.765 0.013

2457711.70699 8.798 0.013

2457711.70922 8.773 0.013

2457711.71142 8.779 0.012

2457711.71362 8.751 0.012

2457711.71583 8.793 0.012

2457711.71804 8.767 0.012

2457711.72025 8.774 0.012

2457711.72249 8.768 0.012

2457711.72468 8.771 0.012

2457711.72688 8.758 0.012

2457711.72907 8.788 0.012

2457711.73128 8.766 0.011

2457711.73348 8.770 0.012

2457711.73569 8.757 0.012

2457711.73789 8.770 0.012

2457711.74009 8.768 0.012

2457711.74228 8.779 0.011

2457711.74448 8.774 0.011

2457711.74668 8.768 0.011

2457711.74890 8.788 0.011

2457711.75110 8.755 0.012

2457711.75331 8.804 0.011

2457711.75552 8.772 0.011

2457711.75773 8.762 0.011

2457711.75994 8.790 0.011

2457711.76215 8.772 0.011

2457711.76435 8.763 0.011

2457711.76657 8.758 0.011

2457711.76877 8.775 0.011

2457711.77098 8.774 0.011

2457711.77319 8.763 0.011

2457711.77541 8.757 0.011

2457711.77759 8.788 0.011

2457711.77980 8.766 0.011

2457711.78201 8.751 0.012

2457711.78420 8.782 0.016

2457711.78642 8.802 0.020

2457711.78861 8.769 0.021

2457711.79083 8.729 0.041

2457711.79302 8.780 0.021

2457711.79522 8.792 0.035

2457711.79742 8.783 0.037

2457711.80182 8.781 0.027

2457711.80402 8.797 0.026

2457711.80622 8.791 0.036

2457711.80841 8.776 0.018

2457711.81061 8.809 0.020

2457711.81280 8.756 0.020

2457711.81499 8.753 0.021

2457711.81719 8.782 0.014

2457711.81939 8.760 0.012

2457711.82159 8.778 0.011

2457711.82380 8.769 0.011

2457711.82598 8.769 0.011

2457711.82816 8.766 0.011

2457711.83035 8.767 0.011

2457711.83253 8.763 0.011

2457711.83473 8.807 0.011

2457711.83694 8.778 0.011

2457711.83926 8.767 0.011

2457711.84157 8.750 0.013

2457711.84373 8.769 0.014

2457711.84582 8.736 0.015

2457713.68162 8.752 0.013

2457713.68383 8.741 0.012

2457713.68604 8.742 0.013

2457713.68825 8.757 0.012

2457713.69043 8.732 0.012

2457713.69263 8.761 0.012

2457713.69484 8.781 0.012

2457713.69704 8.707 0.014

2457713.69925 8.763 0.012

2457713.70146 8.740 0.012

2457713.70366 8.757 0.012

2457713.70588 8.732 0.012

2457713.70808 8.733 0.011

2457713.71029 8.756 0.011

2457713.71472 8.746 0.011

2457713.71693 8.739 0.012

2457713.71913 8.744 0.016

2457713.72133 8.756 0.012

2457713.72355 8.763 0.012

2457713.72579 8.733 0.011

2457713.72801 8.723 0.023

2457713.73022 8.756 0.063

2457713.75671 8.748 0.014

2457713.76112 8.750 0.026

2457713.76334 8.706 0.013

2457713.76556 8.776 0.027

2457713.78541 8.703 0.026

2457713.78984 8.764 0.026

2457713.79206 8.787 0.101

2457713.79426 8.734 0.042

2457713.79868 8.767 0.045

2457713.80089 8.717 0.027

2457713.80311 8.734 0.019

2457713.80534 8.763 0.014

2457713.80755 8.748 0.035

2457713.80976 8.735 0.038

2457713.81199 8.734 0.048

2457713.81638 8.738 0.015

2457713.81859 8.744 0.065

2457713.82080 8.766 0.014

2457713.82301 8.801 0.020

2457713.82522 8.727 0.013

2457713.82743 8.733 0.011

2457713.82963 8.722 0.015

2457713.83183 8.724 0.028

2457713.83416 8.743 0.015

2457713.83648 8.732 0.046

2457713.84072 8.704 0.013

2457713.84497 8.702 0.019

2457713.84707 8.735 0.012

2457714.74303 8.741 0.011

2457714.74522 8.766 0.010

2457714.74741 8.754 0.010

2457714.74959 8.745 0.010

2457714.75178 8.745 0.010

2457714.75397 8.743 0.010

2457714.75613 8.742 0.010

2457714.75832 8.759 0.010

2457714.76051 8.721 0.011

2457714.76270 8.744 0.010

2457714.76487 8.723 0.010

2457714.76705 8.740 0.010

2457714.76922 8.725 0.010

2457714.77140 8.755 0.010

2457714.77359 8.742 0.010

2457714.77575 8.736 0.010

2457714.77793 8.745 0.010

2457714.78012 8.733 0.010

2457714.78229 8.729 0.010

2457714.78447 8.733 0.010

2457714.78664 8.747 0.010

2457714.78882 8.726 0.010

2457714.79102 8.752 0.010

2457714.79321 8.742 0.010

2457714.79538 8.746 0.010

2457714.79756 8.740 0.010

2457714.79973 8.729 0.010

2457714.80192 8.737 0.010

2457714.80411 8.738 0.010

2457714.80630 8.737 0.010

2457714.80847 8.728 0.010

2457714.81065 8.743 0.010

2457714.81285 8.750 0.010

2457714.81503 8.731 0.010

2457714.81723 8.752 0.010

2457714.81942 8.755 0.010

2457714.82160 8.779 0.010

2457714.82377 8.737 0.010

2457714.82595 8.767 0.010

2457714.82814 8.761 0.010

2457714.83032 8.756 0.010

2457714.83263 8.787 0.010

2457714.83477 8.781 0.010

2457714.83685 8.784 0.010

2457714.83894 8.799 0.010

2457714.84103 8.788 0.010

2457714.84313 8.800 0.010

2457714.84521 8.778 0.010

2457715.67539 8.779 0.012

2457715.67770 8.778 0.012

2457715.67948 8.785 0.012

2457715.68124 8.758 0.012

2457715.68303 8.749 0.012

2457715.68480 8.774 0.011

2457715.68657 8.767 0.012

2457715.68837 8.742 0.012

2457715.69019 8.777 0.011

2457715.69240 8.775 0.011

2457715.69459 8.778 0.011

2457715.69679 8.795 0.011

2457715.69898 8.773 0.011

2457715.70118 8.783 0.011

2457715.70340 8.764 0.011

2457715.70560 8.781 0.011

2457715.70780 8.799 0.011

2457715.71002 8.776 0.011

2457715.71223 8.789 0.011

2457715.71443 8.776 0.011

2457715.71662 8.759 0.011

2457715.71883 8.775 0.010

2457715.72105 8.776 0.010

2457715.72323 8.792 0.010

2457715.72544 8.770 0.011

2457715.72764 8.774 0.010

2457715.72983 8.778 0.010

2457715.73205 8.755 0.011

2457715.73426 8.769 0.010

2457715.73646 8.780 0.010

2457715.73869 8.760 0.010

2457715.74088 8.769 0.010

2457715.74308 8.765 0.010

2457715.74529 8.775 0.010

2457715.74749 8.772 0.010

2457715.74969 8.770 0.010

2457715.75190 8.758 0.010

2457715.75410 8.770 0.010

2457715.75630 8.761 0.010

2457715.75850 8.771 0.010

2457715.76069 8.765 0.010

2457715.76288 8.750 0.010

2457715.76508 8.751 0.010

2457715.76728 8.780 0.010

2457715.76949 8.754 0.010

2457715.77169 8.757 0.010

2457715.77389 8.750 0.010

2457715.77609 8.765 0.010

2457715.77829 8.748 0.010

2457715.78049 8.778 0.010

2457715.78269 8.790 0.010

2457715.78488 8.752 0.010

2457715.78708 8.781 0.009

2457715.78928 8.760 0.010

2457715.79148 8.758 0.010

2457715.79368 8.770 0.009

2457715.79588 8.770 0.010

2457715.79808 8.780 0.010

2457715.80028 8.761 0.010

2457715.80248 8.764 0.010

2457715.80468 8.771 0.009

2457715.80688 8.769 0.009

2457715.80907 8.764 0.009

2457715.81128 8.753 0.010

2457715.81348 8.783 0.010

2457715.81568 8.762 0.009

2457715.81788 8.773 0.010

2457715.82008 8.777 0.010

2457715.82227 8.769 0.010

2457715.82448 8.760 0.010

2457715.82668 8.762 0.010

2457715.82888 8.764 0.010

2457715.83108 8.758 0.010

2457715.83323 8.767 0.010

2457715.83551 8.768 0.010

2457715.83780 8.791 0.010

2457715.84008 8.766 0.010

2457715.84235 8.774 0.010

2457716.67416 8.756 0.013

2457716.67590 8.774 0.012

2457716.67767 8.775 0.012

2457716.67944 8.783 0.012

2457716.68122 8.799 0.012

2457716.68300 8.780 0.012

2457716.68479 8.759 0.012

2457716.68656 8.778 0.012

2457716.68892 8.795 0.011

2457716.69131 8.770 0.011

2457716.69369 8.748 0.011

2457716.69608 8.801 0.011

2457716.69846 8.754 0.011

2457716.70086 8.760 0.011

2457716.70324 8.784 0.011

2457716.70563 8.773 0.011

2457716.70802 8.756 0.011

2457716.71042 8.786 0.011

2457716.71280 8.752 0.011

2457716.71517 8.796 0.010

2457716.71755 8.770 0.011

2457716.71992 8.771 0.011

2457716.72230 8.779 0.011

2457716.72468 8.780 0.011

2457716.72707 8.766 0.011

2457716.72946 8.764 0.010

2457716.73184 8.779 0.010

2457716.73422 8.749 0.010

2457716.73660 8.780 0.010

2457716.73897 8.788 0.010

2457716.74135 8.775 0.010

2457716.74375 8.759 0.010

2457716.74612 8.787 0.010

2457716.74850 8.763 0.010

2457716.75089 8.771 0.010

2457716.75325 8.762 0.010

2457716.75563 8.776 0.010

2457716.75802 8.771 0.010

2457716.76039 8.768 0.010

2457716.76279 8.780 0.010

2457716.76519 8.738 0.010

2457716.76756 8.776 0.010

2457716.76995 8.746 0.010

2457716.77236 8.775 0.010

2457716.77475 8.768 0.010

2457716.77713 8.762 0.010

2457716.77950 8.751 0.010

2457716.78189 8.751 0.010

2457716.78426 8.767 0.010

2457716.78663 8.761 0.010

2457716.78902 8.767 0.010

2457716.79140 8.769 0.010

2457716.79378 8.769 0.010

2457716.79617 8.756 0.010

2457716.79856 8.775 0.010

2457716.80094 8.748 0.009

2457716.80332 8.747 0.010

2457716.80569 8.754 0.010

2457716.80807 8.761 0.009

2457716.81047 8.749 0.010

2457716.81285 8.754 0.009

2457716.81521 8.762 0.009

2457716.81758 8.755 0.010

2457716.81995 8.774 0.009

2457716.82233 8.758 0.010

2457716.82470 8.773 0.009

2457716.82707 8.766 0.010

2457716.82940 8.747 0.010

2457716.83185 8.754 0.010

2457716.83431 8.758 0.010

2457716.83676 8.754 0.010

2457716.83921 8.769 0.010

2457716.84167 8.750 0.010

2457717.67132 8.723 0.013

2457717.67288 8.733 0.013

2457717.67442 8.764 0.012

2457717.67598 8.744 0.012

2457717.67755 8.744 0.012

2457717.67911 8.746 0.012

2457717.68066 8.749 0.012

2457717.68221 8.747 0.012

2457717.68378 8.730 0.012

2457717.68532 8.758 0.012

2457717.68689 8.754 0.012

2457717.68846 8.753 0.012

2457717.69003 8.753 0.012

2457717.69159 8.762 0.011

2457717.69315 8.740 0.011

2457717.69470 8.755 0.011

2457717.69624 8.758 0.011

2457717.69780 8.759 0.011

2457717.69936 8.762 0.011

2457717.70091 8.725 0.011

2457717.70247 8.745 0.011

2457717.70403 8.750 0.011

2457717.70559 8.759 0.010

2457717.70715 8.738 0.011

2457717.70872 8.747 0.011

2457717.71028 8.764 0.011

2457717.71184 8.765 0.011

2457717.71340 8.755 0.011

2457717.71495 8.758 0.011

2457717.71650 8.791 0.010

2457717.71807 8.768 0.011

2457717.71963 8.770 0.011

2457717.72118 8.740 0.011

2457717.72274 8.745 0.011

2457717.72429 8.736 0.010

2457717.72586 8.741 0.010

2457717.72743 8.739 0.010

2457717.72899 8.729 0.010

2457717.73054 8.730 0.010

2457717.73209 8.718 0.010

2457717.73366 8.737 0.010

2457717.73521 8.739 0.010

2457717.73676 8.735 0.010

2457717.73831 8.749 0.010

2457717.73986 8.765 0.010

2457717.74139 8.743 0.010

2457717.74293 8.761 0.010

2457717.74448 8.759 0.010

2457717.74602 8.766 0.010

2457717.74756 8.729 0.011

2457717.74910 8.734 0.010

2457717.75064 8.755 0.010

2457717.75219 8.761 0.010

2457717.75374 8.752 0.010

2457717.75529 8.734 0.010

2457717.75685 8.745 0.010

2457717.75840 8.738 0.010

2457717.75995 8.743 0.010

2457717.76150 8.749 0.010

2457717.76304 8.754 0.010

2457717.76459 8.730 0.010

2457717.76615 8.734 0.010

2457717.76770 8.751 0.010

2457717.76924 8.743 0.010

2457717.77078 8.728 0.010

2457717.77233 8.737 0.010

2457717.77388 8.761 0.010

2457717.77543 8.765 0.010

2457717.77698 8.742 0.010

2457717.77853 8.750 0.010

2457717.78007 8.740 0.010

2457717.78161 8.764 0.010

2457717.78316 8.755 0.010

2457717.78471 8.730 0.010

2457717.78627 8.773 0.010

2457717.78782 8.740 0.010

2457717.78938 8.729 0.010

2457717.79093 8.750 0.010

2457717.79248 8.752 0.009

2457717.79403 8.757 0.010

2457717.79557 8.765 0.010

2457717.79712 8.732 0.010

2457717.79867 8.754 0.010

2457717.80022 8.723 0.010

2457717.80176 8.728 0.010

2457717.80330 8.738 0.010

2457717.80484 8.766 0.010

2457717.80639 8.724 0.010

2457717.80794 8.735 0.010

2457717.80949 8.754 0.010

2457717.81104 8.754 0.010

2457717.81259 8.756 0.010

2457717.81414 8.740 0.010

2457717.81569 8.732 0.010

2457717.81725 8.750 0.010

2457717.81881 8.748 0.010

2457717.82036 8.740 0.010

2457717.82190 8.737 0.010

2457717.82345 8.746 0.010

2457717.82500 8.758 0.009

2457717.82650 8.737 0.010

2457717.82797 8.711 0.010

2457717.82944 8.743 0.010

2457717.83091 8.739 0.010

2457717.83237 8.750 0.010

2457717.83384 8.728 0.010

2457717.83530 8.735 0.010

2457717.83676 8.737 0.010

2457717.83822 8.725 0.010

2457717.83968 8.733 0.010

2457717.84115 8.740 0.010

2457717.84260 8.767 0.010

2457718.67006 8.735 0.013

2457718.67309 8.745 0.013

2457718.67612 8.743 0.012

2457718.67914 8.740 0.014

2457718.68215 8.760 0.013

2457718.68519 8.747 0.012

2457718.68822 8.749 0.012

2457718.69125 8.725 0.012

2457718.69428 8.742 0.012

2457718.69730 8.750 0.012

2457718.70032 8.759 0.011

2457718.70337 8.758 0.011

2457718.70639 8.731 0.011

2457718.70942 8.731 0.011

2457718.71244 8.741 0.012

2457718.71546 8.743 0.012

2457718.71848 8.759 0.011

2457718.72153 8.730 0.012

2457718.72456 8.739 0.012

2457718.72760 8.715 0.011

2457718.73066 8.748 0.011

2457718.73368 8.727 0.012

2457718.73671 8.731 0.011

2457718.73975 8.712 0.012

2457718.74278 8.722 0.012

2457718.74581 8.748 0.012

2457718.74885 8.732 0.012

2457718.75189 8.741 0.013

2457718.75492 8.758 0.012

2457718.75797 8.751 0.012

2457718.76098 8.753 0.012

2457718.76403 8.728 0.012

2457718.76708 8.735 0.012

2457718.77014 8.746 0.011

2457718.77317 8.728 0.011

2457718.77623 8.745 0.012

2457718.77927 8.720 0.012

2457718.78233 8.745 0.012

2457718.78538 8.746 0.012

2457718.78838 8.748 0.013

2457718.79140 8.744 0.012

2457718.79443 8.724 0.012

2457718.79749 8.708 0.011

2457718.80052 8.734 0.014

2457718.80355 8.729 0.011

2457718.80657 8.729 0.011

2457718.80959 8.718 0.014

2457718.81263 8.713 0.012

2457718.81565 8.750 0.011

2457718.81867 8.740 0.011

2457718.82168 8.748 0.011

2457718.82463 8.718 0.011

2457718.82757 8.740 0.010

2457718.83051 8.736 0.012

2457718.83346 8.732 0.014

2457718.83642 8.733 0.017

2457718.83935 8.738 0.015

2457718.84228 8.749 0.015

2457719.66664 9.039 0.013

2457719.66904 9.046 0.013

2457719.67146 9.021 0.013

2457719.67387 9.055 0.013

2457719.67626 9.036 0.012

2457719.67866 9.037 0.013

2457719.68106 9.050 0.012

2457719.68347 9.091 0.012

2457719.68586 9.043 0.012

2457719.68824 9.036 0.012

2457719.69065 9.087 0.011

2457719.69304 9.073 0.011

2457719.69546 9.057 0.011

2457719.69786 9.069 0.012

2457719.70025 9.057 0.011

2457719.70267 9.034 0.011

2457719.70506 9.050 0.011

2457719.70744 9.086 0.011

2457719.70984 9.044 0.011

2457719.71225 9.029 0.011

2457719.71463 9.052 0.011

2457719.71704 9.065 0.011

2457719.71944 9.050 0.011

2457719.72185 9.032 0.011

2457719.72422 9.045 0.011

2457719.72662 9.039 0.011

2457719.72903 9.027 0.011

2457719.73144 9.030 0.010

2457719.73387 9.023 0.010

2457719.73627 9.003 0.010

2457719.73869 8.997 0.010

2457719.74111 8.999 0.011

2457719.74353 9.002 0.010

2457719.74594 8.988 0.010

2457719.74833 8.973 0.010

2457719.75074 8.984 0.010

2457719.75314 8.978 0.010

2457719.75552 8.990 0.010

2457719.75795 8.971 0.011

2457719.76034 8.970 0.010

2457719.76273 8.951 0.010

2457719.76514 8.947 0.010

2457719.76757 8.938 0.010

2457719.76998 8.926 0.010

2457719.77237 8.905 0.010

2457719.77477 8.912 0.010

2457719.77716 8.904 0.010

2457719.77957 8.890 0.010

2457719.78199 8.893 0.010

2457719.78439 8.896 0.010

2457719.78679 8.862 0.010

2457719.78920 8.868 0.010

2457719.79160 8.868 0.010

2457719.79399 8.856 0.010

2457719.79639 8.856 0.010

2457719.79878 8.841 0.010

2457719.80118 8.844 0.010

2457719.80358 8.839 0.010

2457719.80597 8.833 0.009

2457719.80836 8.803 0.010

2457719.81075 8.825 0.010

2457719.81315 8.797 0.010

2457719.81556 8.796 0.010

2457719.81795 8.793 0.010

2457719.82028 8.779 0.010

2457719.82256 8.796 0.010

2457719.82481 8.764 0.010

2457719.82708 8.781 0.010

2457719.82935 8.774 0.010

2457719.83163 8.769 0.010

2457719.83391 8.757 0.010

2457719.83618 8.751 0.010

2457719.83845 8.750 0.010

2457719.84072 8.750 0.010

2457719.84299 8.755 0.010

2457720.66525 8.786 0.015

2457720.66830 8.758 0.014

2457720.67139 8.749 0.014

2457720.67447 8.789 0.014

2457720.67753 8.772 0.013

2457720.68059 8.776 0.013

2457720.68366 8.757 0.013

2457720.68671 8.764 0.013

2457720.68976 8.773 0.013

2457720.69285 8.771 0.013

2457720.69591 8.764 0.013

2457720.69895 8.757 0.013

2457720.70199 8.769 0.013

2457720.70507 8.768 0.012

2457720.70813 8.779 0.012

2457720.71116 8.747 0.012

2457720.71422 8.756 0.012

2457720.71728 8.765 0.012

2457720.72030 8.766 0.011

2457720.72337 8.761 0.012

2457720.72644 8.777 0.011

2457720.72947 8.776 0.011

2457720.73252 8.750 0.012

2457720.73557 8.757 0.012

2457720.73865 8.762 0.013

2457720.74171 8.743 0.011

2457720.74476 8.748 0.011

2457720.74780 8.776 0.012

2457720.75088 8.745 0.012

2457720.75395 8.774 0.012

2457720.75703 8.771 0.011

2457720.76008 8.767 0.011

2457720.76317 8.756 0.011

2457720.76623 8.752 0.011

2457720.76929 8.753 0.011

2457720.77234 8.753 0.011

2457720.77539 8.757 0.012

2457720.77844 8.773 0.011

2457720.78149 8.758 0.010

2457720.78459 8.750 0.010

2457720.78771 8.759 0.010

2457720.79083 8.767 0.010

2457720.79396 8.756 0.010

2457720.79707 8.758 0.010

2457720.80020 8.760 0.010

2457720.80333 8.775 0.010

2457720.80644 8.750 0.010

2457720.80954 8.753 0.011

2457720.81264 8.765 0.010

2457720.81574 8.772 0.010

2457720.81870 8.751 0.010

2457720.82164 8.754 0.010

2457720.82457 8.767 0.010

2457720.82749 8.759 0.010

2457720.83043 8.778 0.010

2457720.83334 8.770 0.010

2457720.83627 8.776 0.010

2457720.83919 8.761 0.010

2457720.84212 8.783 0.010

2457721.66243 8.781 0.016

2457721.66547 8.766 0.013

2457721.66853 8.784 0.013

2457721.67161 8.776 0.012

2457721.67468 8.778 0.012

2457721.67773 8.753 0.013

2457721.68079 8.783 0.012

2457721.68384 8.778 0.012

2457721.68691 8.775 0.012

2457721.68995 8.737 0.012

2457721.69301 8.770 0.012

2457721.69606 8.761 0.012

2457721.69910 8.775 0.012

2457721.70215 8.741 0.012

2457721.70520 8.772 0.011

2457721.70825 8.773 0.012

2457721.71131 8.744 0.012

2457721.71436 8.740 0.012

2457721.71743 8.769 0.011

2457721.72049 8.764 0.011

2457721.72355 8.754 0.011

2457721.72663 8.761 0.011

2457721.72969 8.751 0.011

2457721.73275 8.783 0.012

2457721.73582 8.754 0.011

2457721.73887 8.766 0.012

2457721.74193 8.758 0.013

2457721.74500 8.761 0.011

2457721.74807 8.744 0.011

2457721.75113 8.748 0.010

2457721.75417 8.766 0.010

2457721.75723 8.767 0.010

2457721.76030 8.758 0.011

2457721.76334 8.768 0.011

2457721.76638 8.754 0.010

2457721.76943 8.757 0.010

2457721.77248 8.755 0.010

2457721.77552 8.756 0.010

2457721.77855 8.719 0.010

2457721.78168 8.757 0.010

2457721.78477 8.758 0.011

2457721.78787 8.768 0.010

2457721.79097 8.746 0.010

2457721.79407 8.754 0.010

2457721.79716 8.762 0.010

2457721.80027 8.748 0.010

2457721.80337 8.744 0.010

2457721.80647 8.739 0.010

2457721.80957 8.747 0.010

2457721.81269 8.741 0.010

2457721.81566 8.762 0.010

2457721.81859 8.757 0.010

2457721.82153 8.767 0.010

2457721.82444 8.748 0.010

2457721.82738 8.754 0.010

2457721.83032 8.756 0.010

2457721.83322 8.758 0.010

2457721.83616 8.745 0.010

2457721.83909 8.762 0.010

2457721.84200 8.761 0.010

2457722.65965 8.784 0.046

2457722.66273 8.748 0.037

2457722.66576 8.737 0.024

2457722.66882 8.752 0.025

2457722.67186 8.749 0.024

2457722.67493 8.711 0.040

2457722.67800 8.751 0.043

2457722.68105 8.789 0.027

2457722.68412 8.764 0.019

2457722.68720 8.729 0.019

2457722.69689 8.786 0.025

2457722.69994 8.751 0.023

2457722.70300 8.749 0.018

2457722.70606 8.741 0.015

2457722.70912 8.745 0.017

2457722.71218 8.750 0.022

2457722.71523 8.739 0.019

2457722.71826 8.749 0.019

2457722.72133 8.768 0.027

2457722.72744 8.767 0.062

2457722.73051 8.772 0.031

2457722.73358 8.731 0.028

2457722.73667 8.749 0.026

2457722.73972 8.738 0.032

2457722.74279 8.785 0.036

2457722.74581 8.778 0.061

2457724.65332 8.761 0.014

2457724.65552 8.755 0.022

2457724.65773 8.809 0.019

2457724.65997 8.768 0.016

2457724.66220 8.756 0.017

2457724.66442 8.749 0.050

2457724.66666 8.797 0.019

2457724.66888 8.728 0.027

2457724.67111 8.776 0.039

2457724.67332 8.770 0.017

2457724.67554 8.719 0.069

2457724.67774 8.774 0.037

2457724.68220 8.809 0.065

2457724.68444 8.737 0.051

2457724.68889 8.754 0.029

2457724.69111 8.738 0.021

2457724.69333 8.757 0.018

2457724.69557 8.768 0.018

2457724.69781 8.765 0.024

2457724.70002 8.743 0.029

2457724.70225 8.735 0.024

2457724.70446 8.690 0.050

2457724.70667 8.744 0.055

2457724.70889 8.801 0.035

2457724.71110 8.738 0.029

2457724.72222 8.715 0.047

2457724.72446 8.718 0.052

2457724.72668 8.793 0.042

2457724.72890 8.748 0.017

2457724.73112 8.753 0.015

2457724.73332 8.791 0.056

2457724.73554 8.764 0.017

2457724.73778 8.771 0.018

2457724.74000 8.790 0.036

2457724.74223 8.745 0.016

2457724.74444 8.765 0.013

2457724.74668 8.760 0.014

2457724.74891 8.706 0.019

2457724.75112 8.726 0.022

2457724.75334 8.726 0.021

2457724.75557 8.748 0.012

2457724.75779 8.754 0.011

2457724.76001 8.775 0.010

2457724.76223 8.757 0.011

2457724.76446 8.749 0.010

2457724.76668 8.773 0.010

2457724.76890 8.748 0.010

2457724.77112 8.732 0.011

2457724.77341 8.798 0.010

2457724.77571 8.743 0.011

2457724.77799 8.740 0.012

2457724.78028 8.767 0.010

2457724.78258 8.733 0.010

2457724.78488 8.755 0.010

2457724.78718 8.770 0.010

2457724.78947 8.771 0.010

2457724.79176 8.756 0.010

2457724.79406 8.776 0.010

2457724.79634 8.746 0.010

2457724.79863 8.731 0.011

2457724.80093 8.781 0.010

2457724.80322 8.771 0.010

2457724.80551 8.735 0.010

2457724.80767 8.766 0.010

2457724.80979 8.759 0.010

2457724.81189 8.753 0.010

2457724.81399 8.751 0.010

2457724.81611 8.760 0.010

2457724.81822 8.756 0.010

2457724.82032 8.746 0.013

2457724.82242 8.801 0.011

2457724.82455 8.772 0.010

2457724.82667 8.758 0.010

2457724.82878 8.748 0.010

2457724.83091 8.779 0.010

2457724.83303 8.799 0.010

2457724.83516 8.741 0.010

2457724.83728 8.735 0.010

2457724.83940 8.738 0.010

2457724.84153 8.744 0.010

2457725.65088 8.814 0.013

2457725.65356 8.746 0.013

2457725.65627 8.770 0.013

2457725.65898 8.783 0.013

2457725.66168 8.776 0.012

2457725.66438 8.769 0.013

2457725.66708 8.798 0.012

2457725.66979 8.763 0.012

2457725.67247 8.771 0.011

2457725.67517 8.757 0.012

2457725.67787 8.747 0.012

2457725.68059 8.756 0.012

2457725.68329 8.803 0.011

2457725.68600 8.754 0.011

2457725.68869 8.769 0.011

2457725.69139 8.774 0.011

2457725.69407 8.785 0.012

2457725.69678 8.759 0.011

2457725.69948 8.756 0.011

2457725.70219 8.765 0.011

2457725.70490 8.755 0.011

2457725.70759 8.794 0.011

2457725.71030 8.770 0.011

2457725.71300 8.761 0.011

2457725.71572 8.762 0.010

2457725.71840 8.751 0.011

2457725.72111 8.744 0.011

2457725.72384 8.789 0.010

2457725.72655 8.748 0.011

2457725.72924 8.748 0.011

2457725.73191 8.772 0.010

2457725.73461 8.773 0.011

2457725.73730 8.756 0.011

2457725.74000 8.781 0.010

2457725.74269 8.763 0.011

2457725.74538 8.759 0.010

2457725.74810 8.779 0.010

2457725.75081 8.769 0.010

2457725.75361 8.775 0.010

2457725.75641 8.781 0.010

2457725.75921 8.772 0.010

2457725.76203 8.769 0.010

2457725.76483 8.766 0.010

2457725.76764 8.773 0.010

2457725.77043 8.776 0.010

2457725.77317 8.774 0.010

2457725.77595 8.772 0.010

2457725.77872 8.760 0.010

2457725.78150 8.750 0.010

2457725.78428 8.794 0.010

2457725.78707 8.774 0.010

2457725.78986 8.768 0.010

2457725.79264 8.766 0.010

2457725.79541 8.763 0.010

2457725.79817 8.763 0.010

2457725.80095 8.777 0.010

2457725.80374 8.771 0.010

2457725.80639 8.797 0.010

2457725.80896 8.773 0.010

2457725.81155 8.763 0.010

2457725.81413 8.771 0.010

2457725.81674 8.769 0.010

2457725.81929 8.785 0.010

2457725.82185 8.765 0.010

2457725.82443 8.771 0.010

2457725.82703 8.775 0.010

2457725.82962 8.757 0.010

2457725.83220 8.776 0.010

2457725.83477 8.763 0.010

2457725.83736 8.765 0.010

2457726.64834 8.727 0.023

2457726.65204 8.752 0.022

2457726.65573 8.712 0.022

2457726.65944 8.749 0.022

2457726.66313 8.737 0.022

2457726.66682 8.725 0.022

2457726.67052 8.750 0.021

2457726.67421 8.768 0.020

2457726.67790 8.732 0.020

2457726.68159 8.783 0.020

2457726.68528 8.798 0.019

2457726.68899 8.753 0.020

2457726.69268 8.730 0.019

2457726.69638 8.745 0.020

2457726.70011 8.739 0.019

2457726.70381 8.784 0.018

2457726.70747 8.774 0.018

2457726.71117 8.772 0.019

2457726.71484 8.835 0.024

2457726.71854 8.797 0.022

2457726.72224 8.805 0.018

2457726.72594 8.750 0.021

2457726.72966 8.801 0.018

2457726.73334 8.814 0.018

2457726.73705 8.784 0.018

2457726.74076 8.798 0.017

2457726.74444 8.807 0.018

2457726.74815 8.830 0.018

2457726.75190 8.785 0.018

2457726.75562 8.831 0.017

2457726.75933 8.816 0.018

2457726.76305 8.845 0.018

2457726.76677 8.857 0.018

2457726.77046 8.815 0.018

2457726.77416 8.820 0.017

2457726.77785 8.829 0.018

2457726.78156 8.826 0.017

2457726.78526 8.859 0.017

2457726.78896 8.866 0.017

2457726.79267 8.849 0.018

2457726.79638 8.854 0.017

2457726.80010 8.854 0.017

2457726.80370 8.839 0.017

2457726.80747 8.863 0.017

2457726.81123 8.836 0.017

2457726.81499 8.840 0.017

2457726.81876 8.849 0.017

2457726.82252 8.856 0.017

2457726.82986 8.859 0.018

2457726.83343 8.863 0.018

2457727.64410 8.737 0.023

2457727.64633 8.746 0.023

2457727.64854 8.761 0.023

2457727.65076 8.775 0.022

2457727.65296 8.703 0.023

2457727.65517 8.744 0.022

2457727.65737 8.804 0.027

2457727.65958 8.761 0.023

2457727.66180 8.745 0.022

2457727.66401 8.743 0.023

2457727.66621 8.760 0.020

2457727.66842 8.715 0.022

2457727.67511 8.727 0.020

2457727.67730 8.700 0.021

2457727.67953 8.748 0.020

2457727.68177 8.750 0.021

2457727.68398 8.767 0.019

2457727.68620 8.764 0.019

2457727.68842 8.770 0.021

2457727.69064 8.734 0.020

2457727.69285 8.730 0.020

2457727.69508 8.766 0.019

2457727.69727 8.754 0.020

2457727.69948 8.751 0.019

2457727.70168 8.729 0.019

2457727.70387 8.700 0.019

2457727.70608 8.778 0.019

2457727.70829 8.763 0.018

2457727.71052 8.753 0.019

2457727.71275 8.717 0.019

2457727.71497 8.737 0.019

2457727.71720 8.738 0.018

2457727.71943 8.713 0.020

2457727.72164 8.754 0.018

2457727.72384 8.732 0.018

2457727.72604 8.725 0.019

2457727.72822 8.744 0.019

2457727.73042 8.752 0.017

2457727.73266 8.748 0.018

2457727.73488 8.725 0.018

2457727.73711 8.742 0.019

2457727.73930 8.725 0.017

2457727.74151 8.783 0.017

2457727.74372 8.715 0.018

2457727.74595 8.752 0.019

2457727.74815 8.739 0.018

2457727.75034 8.764 0.017

2457727.75255 8.717 0.018

2457727.75474 8.743 0.018

2457727.75695 8.757 0.017

2457727.75944 8.749 0.017

2457727.76194 8.742 0.018

2457727.76442 8.757 0.018

2457727.76692 8.724 0.019

2457727.76940 8.730 0.018

2457727.77188 8.781 0.016

2457727.77436 8.757 0.018

2457727.77678 8.701 0.018

2457727.77921 8.767 0.017

2457727.78163 8.709 0.022

2457727.78406 8.698 0.093

2457727.78891 8.711 0.020

2457727.79131 8.753 0.018

2457727.79373 8.747 0.017

2457727.79617 8.757 0.017

2457727.79848 8.741 0.018

2457727.80099 8.724 0.019

2457727.80597 8.723 0.018

2457727.80844 8.718 0.018

2457727.81094 8.726 0.017

2457727.81344 8.745 0.018

2457727.81594 8.725 0.018

2457727.81842 8.761 0.017

2457727.82312 8.735 0.017

2457727.82535 8.739 0.017

2457727.82755 8.714 0.017

2457727.82976 8.736 0.018

2457727.83416 8.741 0.017

2457728.64136 8.733 0.024

2457728.64361 8.818 0.022

2457728.64580 8.759 0.023

2457728.64803 8.707 0.023

2457728.65024 8.753 0.022

2457728.65244 8.772 0.022

2457728.65466 8.718 0.023

2457728.65687 8.753 0.022

2457728.65908 8.727 0.022

2457728.66130 8.706 0.022

2457728.66352 8.715 0.023

2457728.66573 8.739 0.021

2457728.66793 8.740 0.021

2457728.67016 8.718 0.020

2457728.67237 8.740 0.020

2457728.67458 8.755 0.020

2457728.67678 8.747 0.021

2457728.67899 8.751 0.020

2457728.68119 8.753 0.020

2457728.68343 8.755 0.021

2457728.68563 8.720 0.021

2457728.68785 8.730 0.019

2457728.69005 8.739 0.019

2457728.69227 8.755 0.019

2457728.69447 8.729 0.019

2457728.69668 8.736 0.020

2457728.69888 8.761 0.019

2457728.70111 8.771 0.019

2457728.70335 8.710 0.019

2457728.70556 8.716 0.019

2457728.70778 8.752 0.018

2457728.71001 8.741 0.019

2457728.71222 8.709 0.018

2457728.71443 8.723 0.019

2457728.71663 8.727 0.019

2457728.71883 8.730 0.019

2457728.72104 8.741 0.019

2457728.72327 8.739 0.019

2457728.72548 8.719 0.019

2457728.72768 8.727 0.018

2457728.72991 8.751 0.018

2457728.73212 8.729 0.019

2457728.73436 8.724 0.018

2457728.73657 8.726 0.018

2457728.73879 8.693 0.018

2457728.74100 8.705 0.019

2457728.74321 8.738 0.019

2457728.74542 8.732 0.018

2457728.74764 8.714 0.018

2457728.74984 8.768 0.017

2457728.75205 8.772 0.018

2457728.75425 8.726 0.018

2457728.75674 8.722 0.018

2457728.75922 8.700 0.018

2457728.76171 8.724 0.017

2457728.76421 8.733 0.017

2457728.76670 8.771 0.018

2457728.76918 8.752 0.018

2457728.77167 8.726 0.018

2457728.77410 8.772 0.018

2457728.77652 8.714 0.017

2457728.77894 8.759 0.017

2457728.78136 8.706 0.018

2457728.78379 8.713 0.018

2457728.78623 8.763 0.017

2457728.78865 8.719 0.018

2457728.79107 8.729 0.017

2457728.79351 8.734 0.017

2457728.79585 8.750 0.018

2457728.79832 8.728 0.018

2457728.80082 8.708 0.019

2457728.80329 8.761 0.018

2457728.80577 8.728 0.017

2457728.80826 8.750 0.018

2457728.81076 8.772 0.018

2457728.81323 8.719 0.018

2457728.81570 8.722 0.017

2457728.81818 8.729 0.017

2457728.82038 8.735 0.018

2457728.82258 8.738 0.018

2457728.82477 8.751 0.017

2457728.82700 8.715 0.018

2457728.82921 8.766 0.017

2457728.83142 8.754 0.017

2457728.83363 8.731 0.018

2457729.63984 8.755 0.024

2457729.64205 8.723 0.024

2457729.64425 8.774 0.022

2457729.64646 8.797 0.022

2457729.64869 8.739 0.023

2457729.65089 8.730 0.022

2457729.65310 8.741 0.023

2457729.65532 8.766 0.021

2457729.65751 8.721 0.021

2457729.65969 8.749 0.022

2457729.66192 8.803 0.021

2457729.66413 8.735 0.021

2457729.66633 8.814 0.020

2457729.66851 8.787 0.021

2457729.67073 8.766 0.021

2457729.67293 8.773 0.020

2457729.67514 8.800 0.019

2457729.67734 8.761 0.020

2457729.67955 8.763 0.020

2457729.68177 8.767 0.019

2457729.68399 8.763 0.020

2457729.68617 8.714 0.021

2457729.68840 8.758 0.019

2457729.69062 8.734 0.020

2457729.69283 8.766 0.019

2457729.69504 8.764 0.020

2457729.69726 8.728 0.020

2457729.69947 8.783 0.019

2457729.70170 8.805 0.019

2457729.70392 8.760 0.019

2457729.70615 8.749 0.019

2457729.70837 8.761 0.020

2457729.71058 8.755 0.019

2457729.71281 8.770 0.019

2457729.71504 8.747 0.019

2457729.71726 8.773 0.018

2457729.71946 8.776 0.019

2457729.72166 8.728 0.019

2457729.72387 8.712 0.019

2457729.72608 8.774 0.018

2457729.72830 8.761 0.018

2457729.73053 8.773 0.018

2457729.73275 8.767 0.018

2457729.73497 8.749 0.019

2457729.73719 8.752 0.018

2457729.73943 8.739 0.019

2457729.74165 8.744 0.018

2457729.74386 8.764 0.018

2457729.74607 8.747 0.018

2457729.74827 8.748 0.018

2457729.75049 8.749 0.018

2457729.75297 8.790 0.018

2457729.75543 8.749 0.017

2457729.75792 8.774 0.018

2457729.76040 8.762 0.018

2457729.76289 8.766 0.018

2457729.76536 8.761 0.017

2457729.76785 8.764 0.018

2457729.77035 8.783 0.018

2457729.77276 8.775 0.017

2457729.77518 8.741 0.018

2457729.77760 8.780 0.017

2457729.78002 8.772 0.017

2457729.78244 8.753 0.018

2457729.78486 8.744 0.017

2457729.78729 8.740 0.018

2457729.78970 8.769 0.018

2457729.79211 8.772 0.017

2457729.79442 8.722 0.018

2457729.79689 8.704 0.018

2457729.79940 8.764 0.018

2457729.80188 8.734 0.018

2457729.80439 8.754 0.018

2457729.80687 8.727 0.018

2457729.80935 8.708 0.018

2457729.81183 8.749 0.018

2457729.81429 8.758 0.017

2457729.81674 8.770 0.017

2457729.81896 8.761 0.018

2457729.82117 8.789 0.017

2457729.82339 8.794 0.018

2457729.82558 8.724 0.018

2457729.82778 8.744 0.019

2457729.82998 8.750 0.018

2457729.83218 8.753 0.018

2457729.83438 8.768 0.017
