# Supplementary material for: HD 66051, an eclipsing binary hosting a highly peculiar, HgMn-related star
Source: Sci Rep. 2017 Jul 19;7:5906. doi: 10.1038/s41598-017-05987-6 (PMC5517476; doi:10.1038/s41598-017-05987-6)
Supplement: Supplementary file 2 — Supplementary Dataset 2 [file 41598_2017_5987_MOESM2_ESM.doc]

HD66051, an eclipsing binary hosting a highly peculiar, HgMn-related star

Ewa Niemczura

Stefan Hümmerich

Fiorella Castelli

Ernst Paunzen

Klaus Bernhard

Franz-Josef Hambsch

Krzysztof Hełminiak

JD mag(Ic) mag_err

2457795.50376 8.776 0.017

2457795.50648 8.830 0.016

2457795.50922 8.812 0.016

2457795.51195 8.759 0.016

2457795.51468 8.790 0.015

2457795.51741 8.806 0.015

2457795.52012 8.822 0.015

2457795.52557 8.767 0.015

2457795.52832 8.772 0.015

2457795.53101 8.782 0.015

2457795.53376 8.813 0.016

2457795.53648 8.773 0.015

2457795.53921 8.811 0.014

2457795.54195 8.793 0.015

2457795.54468 8.766 0.015

2457795.54741 8.779 0.015

2457795.55016 8.818 0.015

2457795.55285 8.787 0.015

2457795.55560 8.822 0.014

2457795.55832 8.858 0.015

2457795.56104 8.834 0.015

2457795.56377 8.825 0.015

2457795.56652 8.851 0.015

2457795.56924 8.851 0.015

2457795.57198 8.838 0.014

2457795.57470 8.845 0.015

2457795.57744 8.868 0.014

2457795.58017 8.854 0.015

2457795.58290 8.847 0.015

2457795.58563 8.876 0.015

2457795.58834 8.916 0.015

2457795.59107 8.876 0.015

2457795.59381 8.892 0.015

2457795.59655 8.926 0.014

2457795.59929 8.919 0.014

2457795.60201 8.921 0.015

2457795.60474 8.945 0.014

2457795.60745 8.954 0.015

2457795.61018 8.980 0.014

2457795.61292 8.941 0.015

2457795.65975 9.021 0.015

2457795.66466 9.067 0.015

2457795.66960 9.086 0.014

2457795.67454 9.057 0.015

2457795.68076 9.094 0.016

2457795.68569 9.078 0.015

2457795.69063 9.088 0.015

2457795.69557 9.091 0.015

2457795.70050 9.089 0.015

2457795.70545 9.045 0.015

2457795.71040 9.080 0.015

2457795.71534 9.059 0.016

2457795.72028 9.038 0.016

2457795.72524 9.068 0.015

2457795.73018 9.006 0.016

2457795.73512 9.002 0.016

2457795.74006 8.990 0.015

2457795.74501 9.016 0.016

2457795.74994 8.925 0.017

2457795.75487 8.962 0.016

2457795.75980 8.937 0.017

2457795.76475 8.939 0.017

2457795.76970 8.918 0.016

2457795.77466 8.924 0.017

2457795.77957 8.917 0.017

2457795.78453 8.900 0.017

2457795.78947 8.883 0.018

2457795.79443 8.877 0.018

2457795.79935 8.880 0.019

2457795.80417 8.822 0.019

2457795.80898 8.877 0.050

2457795.81379 8.818 0.020

2457795.81861 8.819 0.021

2457795.82342 8.797 0.020

2457796.51469 8.799 0.018

2457796.52078 8.815 0.027

2457796.52887 8.817 0.015

2457796.53090 8.826 0.015

2457796.53291 8.837 0.014

2457796.53494 8.809 0.015

2457796.53695 8.815 0.015

2457796.53899 8.774 0.015

2457796.54102 8.840 0.015

2457796.54304 8.816 0.015

2457796.54506 8.830 0.014

2457796.54709 8.820 0.015

2457796.54910 8.843 0.015

2457796.55113 8.787 0.018

2457796.55315 8.855 0.017

2457796.55519 8.762 0.019

2457796.55722 8.812 0.023

2457796.55924 8.845 0.015

2457796.56127 8.806 0.014

2457796.56329 8.851 0.014

2457796.56532 8.794 0.015

2457796.56734 8.787 0.015

2457796.56938 8.805 0.014

2457796.57141 8.815 0.015

2457796.57343 8.809 0.015

2457796.57547 8.823 0.014

2457796.57749 8.784 0.014

2457796.57952 8.865 0.015

2457796.58156 8.819 0.015

2457796.58359 8.767 0.014

2457796.58766 8.841 0.014

2457796.58968 8.842 0.014

2457796.59171 8.820 0.014

2457796.59373 8.787 0.015

2457796.59576 8.820 0.014

2457796.59778 8.796 0.014

2457796.59981 8.837 0.014

2457796.60183 8.812 0.014

2457796.60386 8.819 0.014

2457796.60589 8.793 0.014

2457796.60791 8.824 0.014

2457796.60994 8.808 0.014

2457796.61189 8.800 0.014

2457796.61400 8.824 0.014

2457796.61609 8.780 0.014

2457796.61820 8.813 0.014

2457796.62032 8.817 0.014

2457796.62242 8.821 0.015

2457796.62453 8.828 0.014

2457796.62665 8.832 0.014

2457796.62877 8.813 0.015

2457796.63087 8.807 0.015

2457796.65700 8.774 0.014

2457796.66141 8.814 0.014

2457796.66582 8.808 0.014

2457796.67023 8.817 0.015

2457796.67465 8.817 0.014

2457796.67798 8.810 0.014

2457796.68241 8.800 0.014

2457796.68682 8.816 0.015

2457796.69127 8.791 0.015

2457796.69567 8.781 0.015

2457796.70009 8.804 0.015

2457796.70450 8.809 0.014

2457796.70892 8.793 0.015

2457796.71334 8.817 0.015

2457796.71776 8.780 0.015

2457796.72220 8.790 0.015

2457796.72663 8.813 0.015

2457796.73104 8.787 0.015

2457796.73546 8.810 0.015

2457796.73990 8.802 0.015

2457796.74433 8.804 0.016

2457796.74873 8.802 0.016

2457796.75318 8.846 0.015

2457796.75759 8.790 0.016

2457796.76201 8.816 0.015

2457796.76644 8.824 0.015

2457796.77084 8.783 0.016

2457796.77527 8.770 0.016

2457796.77970 8.810 0.017

2457796.78411 8.801 0.017

2457796.78856 8.807 0.017

2457796.79298 8.817 0.018

2457796.79740 8.850 0.017

2457796.80170 8.834 0.017

2457796.80599 8.829 0.018

2457796.81031 8.793 0.019

2457796.81459 8.813 0.019

2457796.81891 8.810 0.020

2457796.82319 8.842 0.021

2457797.50487 8.835 0.014

2457797.50806 8.792 0.015

2457797.51127 8.812 0.014

2457797.51446 8.790 0.014

2457797.51768 8.814 0.014

2457797.52087 8.808 0.014

2457797.52407 8.822 0.014

2457797.52726 8.833 0.014

2457797.53046 8.820 0.014

2457797.53366 8.808 0.014

2457797.53686 8.801 0.014

2457797.54005 8.814 0.014

2457797.54323 8.798 0.014

2457797.54642 8.792 0.014

2457797.54962 8.793 0.014

2457797.55282 8.796 0.014

2457797.55600 8.835 0.014

2457797.55920 8.800 0.015

2457797.56239 8.793 0.014

2457797.56558 8.789 0.014

2457797.56878 8.813 0.014

2457797.57198 8.807 0.014

2457797.57520 8.799 0.014

2457797.57842 8.837 0.014

2457797.58160 8.851 0.014

2457797.58480 8.811 0.014

2457797.58802 8.843 0.014

2457797.59122 8.807 0.014

2457797.59439 8.808 0.014

2457797.59760 8.833 0.013

2457797.60089 8.783 0.014

2457797.60420 8.815 0.014

2457797.60732 8.789 0.014

2457797.61062 8.825 0.014

2457797.61391 8.818 0.014

2457797.61717 8.838 0.014

2457797.62365 8.844 0.014

2457797.62688 8.816 0.014

2457797.65424 8.776 0.014

2457797.65925 8.831 0.014

2457797.66428 8.797 0.014

2457797.66931 8.801 0.014

2457797.67527 8.817 0.014

2457797.68032 8.827 0.014

2457797.68536 8.813 0.014

2457797.69041 8.800 0.014

2457797.69546 8.820 0.015

2457797.70053 8.813 0.014

2457797.70557 8.813 0.015

2457797.71063 8.806 0.014

2457797.71568 8.804 0.014

2457797.72071 8.828 0.015

2457797.72575 8.784 0.015

2457797.73079 8.785 0.015

2457797.73587 8.814 0.015

2457797.74090 8.786 0.015

2457797.74590 8.828 0.015

2457797.75091 8.817 0.015

2457797.75595 8.845 0.015

2457798.50290 8.767 0.015

2457798.50497 8.774 0.015

2457798.50704 8.749 0.015

2457798.50911 8.757 0.015

2457798.51117 8.786 0.015

2457798.51323 8.774 0.015

2457798.51530 8.807 0.014

2457798.51737 8.748 0.014

2457798.51945 8.792 0.015

2457798.52152 8.791 0.015

2457798.52359 8.793 0.014

2457798.52565 8.802 0.014

2457798.52775 8.757 0.014

2457798.52982 8.812 0.014

2457798.53187 8.807 0.014

2457798.53396 8.768 0.014

2457798.53604 8.765 0.014

2457798.53811 8.795 0.014

2457798.54018 8.779 0.014

2457798.54227 8.766 0.014

2457798.54432 8.804 0.014

2457798.54641 8.798 0.014

2457798.54848 8.809 0.014

2457798.55058 8.791 0.014

2457798.55264 8.796 0.014

2457798.55473 8.803 0.014

2457798.55681 8.789 0.014

2457798.55888 8.798 0.014

2457798.56095 8.804 0.014

2457798.56303 8.803 0.014

2457798.56510 8.789 0.014

2457798.56717 8.780 0.014

2457798.56925 8.771 0.014

2457798.57134 8.817 0.014

2457798.57341 8.802 0.014

2457798.57550 8.775 0.014

2457798.57759 8.804 0.014

2457798.57966 8.762 0.014

2457798.58173 8.784 0.014

2457798.58381 8.794 0.014

2457798.58589 8.742 0.014

2457798.58796 8.783 0.014

2457798.59003 8.796 0.014

2457798.59211 8.782 0.014

2457798.59420 8.796 0.014

2457798.59628 8.801 0.014

2457798.59836 8.794 0.014

2457798.60046 8.801 0.014

2457798.60256 8.816 0.013

2457798.60466 8.795 0.014

2457798.60675 8.774 0.014

2457798.60884 8.785 0.014

2457798.61092 8.800 0.014

2457798.61303 8.776 0.014

2457798.61510 8.763 0.014

2457798.61717 8.782 0.014

2457798.61924 8.767 0.014

2457798.62134 8.761 0.014

2457798.62344 8.776 0.014

2457798.62565 8.795 0.013

2457798.65155 8.769 0.014

2457798.65600 8.779 0.015

2457798.66043 8.773 0.014

2457798.66484 8.782 0.014

2457798.66929 8.761 0.015

2457798.67239 8.778 0.014

2457798.67685 8.786 0.014

2457798.68128 8.792 0.014

2457798.68572 8.794 0.014

2457798.69016 8.791 0.014

2457798.69461 8.793 0.014

2457798.69906 8.775 0.014

2457798.70352 8.784 0.014

2457798.70796 8.788 0.014

2457798.71241 8.759 0.014

2457798.71686 8.758 0.015

2457798.72131 8.822 0.014

2457798.72575 8.767 0.015

2457798.73018 8.789 0.014

2457798.73459 8.759 0.015

2457798.73906 8.763 0.015

2457798.74348 8.763 0.014

2457798.74789 8.789 0.015

2457798.75230 8.778 0.015

2457799.50597 8.808 0.017

2457799.51510 8.772 0.016

2457799.51738 8.809 0.018

2457799.51965 8.759 0.016

2457799.52192 8.781 0.016

2457799.52422 8.747 0.016

2457799.52650 8.793 0.016

2457799.52880 8.785 0.015

2457799.53107 8.777 0.016

2457799.53335 8.774 0.015

2457799.53564 8.775 0.015

2457799.53791 8.794 0.015

2457799.54020 8.762 0.014

2457799.54249 8.776 0.015

2457799.54481 8.780 0.015

2457799.54710 8.778 0.015

2457799.54942 8.789 0.015

2457799.55172 8.780 0.015

2457799.55399 8.769 0.015

2457799.55627 8.778 0.015

2457799.55857 8.798 0.014

2457799.56086 8.785 0.015

2457799.56317 8.758 0.015

2457799.56546 8.773 0.015

2457799.56775 8.766 0.015

2457799.57003 8.763 0.014

2457799.57233 8.774 0.015

2457799.57466 8.778 0.014

2457799.57693 8.745 0.014

2457799.57922 8.764 0.015

2457799.58152 8.761 0.014

2457799.58384 8.765 0.015

2457799.58614 8.766 0.015

2457799.58843 8.804 0.014

2457799.59073 8.790 0.015

2457799.59304 8.794 0.014

2457799.59532 8.785 0.014

2457799.59761 8.803 0.015

2457799.59989 8.788 0.015

2457799.60218 8.791 0.015

2457799.60448 8.787 0.014

2457799.60677 8.797 0.014

2457799.60908 8.768 0.014

2457799.61138 8.760 0.014

2457799.61366 8.775 0.015

2457799.61598 8.759 0.015

2457799.61825 8.783 0.014

2457799.62056 8.805 0.014

2457799.64970 8.780 0.014

2457799.65262 8.799 0.014

2457799.65557 8.781 0.015

2457799.65850 8.773 0.015

2457799.66145 8.767 0.014

2457799.66439 8.790 0.014

2457799.66731 8.777 0.015

2457799.66966 8.786 0.015

2457799.67408 8.780 0.014

2457799.67848 8.809 0.014

2457799.68285 8.768 0.014

2457799.68727 8.771 0.014

2457799.69171 8.795 0.014

2457799.69608 8.800 0.014

2457799.70049 8.782 0.014

2457799.70492 8.779 0.015

2457799.70933 8.801 0.015

2457799.71373 8.786 0.015

2457799.71811 8.762 0.015

2457799.72251 8.778 0.015

2457799.72692 8.779 0.015

2457799.73134 8.772 0.015

2457799.73576 8.803 0.015

2457799.74017 8.791 0.016

2457799.74457 8.786 0.015

2457799.74895 8.793 0.015

2457800.50202 8.991 0.015

2457800.50341 8.973 0.016

2457800.50479 8.968 0.015

2457800.50616 8.957 0.016

2457800.50755 8.956 0.015

2457800.50892 8.975 0.015

2457800.51032 8.982 0.015

2457800.51171 8.969 0.015

2457800.51308 8.944 0.015

2457800.51448 8.946 0.015

2457800.51585 8.930 0.015

2457800.51723 8.917 0.015

2457800.51861 8.916 0.016

2457800.51998 8.908 0.015

2457800.52137 8.929 0.015

2457800.52276 8.900 0.015

2457800.52415 8.885 0.015

2457800.52553 8.903 0.015

2457800.52690 8.900 0.015

2457800.52828 8.887 0.015

2457800.52966 8.888 0.016

2457800.53104 8.910 0.015

2457800.53242 8.867 0.015

2457800.53383 8.899 0.015

2457800.53520 8.889 0.015

2457800.53659 8.881 0.014

2457800.53799 8.863 0.015

2457800.53936 8.909 0.015

2457800.54075 8.883 0.015

2457800.54214 8.839 0.016

2457800.54350 8.891 0.015

2457800.54488 8.843 0.015

2457800.54627 8.849 0.015

2457800.54764 8.864 0.015

2457800.54905 8.866 0.015

2457800.55041 8.864 0.014

2457800.55178 8.843 0.015

2457800.55317 8.869 0.014

2457800.55455 8.824 0.015

2457800.55593 8.839 0.014

2457800.55732 8.853 0.014

2457800.55871 8.812 0.015

2457800.56008 8.847 0.015

2457800.56148 8.847 0.015

2457800.56288 8.823 0.015

2457800.56424 8.823 0.015

2457800.56562 8.816 0.015

2457800.56702 8.818 0.014

2457800.56840 8.798 0.014

2457800.56980 8.830 0.015

2457800.57119 8.827 0.014

2457800.57258 8.795 0.014

2457800.57396 8.813 0.014

2457800.57535 8.816 0.014

2457800.57674 8.795 0.014

2457800.57812 8.822 0.014

2457800.57950 8.795 0.014

2457800.58090 8.776 0.014

2457800.58227 8.810 0.014

2457800.58365 8.786 0.014

2457800.58503 8.818 0.014

2457800.58641 8.818 0.014

2457800.58780 8.800 0.014

2457800.58920 8.789 0.015

2457800.59058 8.806 0.015

2457800.59198 8.787 0.014

2457800.59337 8.821 0.014

2457800.59475 8.797 0.015

2457800.59613 8.826 0.014

2457800.59749 8.826 0.015

2457800.59889 8.776 0.014

2457800.60030 8.761 0.015

2457800.60168 8.797 0.015

2457800.60306 8.789 0.014

2457800.60444 8.765 0.014

2457800.60582 8.790 0.015

2457800.60722 8.782 0.015

2457800.60862 8.800 0.014

2457800.61001 8.801 0.015

2457800.61139 8.803 0.014

2457800.61278 8.809 0.014

2457800.61416 8.797 0.015

2457800.61556 8.788 0.015

2457800.61694 8.807 0.014

2457800.61832 8.797 0.015

2457800.61984 8.805 0.015

2457800.64583 8.789 0.015

2457800.64928 8.804 0.015

2457800.65277 8.772 0.014

2457800.65622 8.751 0.015

2457800.65969 8.817 0.014

2457800.66313 8.783 0.013

2457800.66683 8.812 0.014

2457800.67126 8.800 0.015

2457800.67568 8.772 0.014

2457800.68011 8.820 0.014

2457800.68454 8.786 0.015

2457800.68898 8.812 0.014

2457800.69343 8.784 0.015

2457800.69788 8.801 0.014

2457800.70232 8.778 0.014

2457800.70680 8.762 0.015

2457800.71124 8.768 0.015

2457800.71570 8.805 0.014

2457800.72012 8.794 0.015

2457800.72457 8.758 0.015

2457800.72902 8.811 0.015

2457800.73348 8.812 0.015

2457800.73795 8.744 0.015

2457800.74241 8.816 0.017

2457800.74686 8.811 0.019

2457801.50182 8.789 0.015

2457801.50401 8.822 0.015

2457801.50619 8.809 0.015

2457801.50835 8.789 0.014

2457801.51053 8.762 0.014

2457801.51270 8.813 0.014

2457801.51487 8.792 0.014

2457801.51707 8.824 0.014

2457801.51922 8.823 0.014

2457801.52142 8.801 0.014

2457801.52362 8.797 0.014

2457801.52579 8.812 0.014

2457801.52797 8.802 0.014

2457801.53016 8.821 0.014

2457801.53233 8.804 0.014

2457801.53451 8.781 0.015

2457801.53670 8.816 0.014

2457801.53888 8.817 0.014

2457801.54106 8.797 0.014

2457801.54326 8.801 0.013

2457801.54542 8.812 0.014

2457801.54761 8.833 0.014

2457801.54976 8.831 0.014

2457801.55197 8.815 0.014

2457801.55415 8.806 0.014

2457801.55634 8.791 0.014

2457801.55851 8.846 0.014

2457801.56071 8.831 0.014

2457801.56290 8.817 0.014

2457801.56509 8.801 0.014

2457801.56730 8.790 0.014

2457801.56946 8.800 0.014

2457801.57165 8.809 0.014

2457801.57381 8.831 0.014

2457801.57598 8.814 0.014

2457801.57817 8.828 0.013

2457801.58036 8.788 0.014

2457801.58255 8.798 0.014

2457801.58475 8.813 0.014

2457801.58695 8.810 0.014

2457801.58913 8.806 0.014

2457801.59130 8.809 0.014

2457801.59349 8.804 0.014

2457801.59569 8.849 0.013

2457801.59789 8.818 0.014

2457801.60009 8.804 0.014

2457801.60226 8.807 0.014

2457801.60447 8.805 0.014

2457801.60667 8.807 0.014

2457801.60886 8.835 0.013

2457801.61107 8.791 0.014

2457801.61327 8.786 0.014

2457801.61548 8.808 0.014

2457801.64495 8.836 0.014

2457801.64935 8.805 0.014

2457801.65377 8.823 0.014

2457801.65820 8.797 0.014

2457801.66261 8.826 0.014

2457801.66483 8.847 0.014

2457801.66992 8.822 0.014

2457801.67499 8.803 0.014

2457801.68005 8.840 0.014

2457801.68512 8.816 0.014

2457801.69021 8.803 0.014

2457801.69527 8.821 0.014

2457801.70038 8.800 0.015

2457801.70546 8.785 0.015

2457801.71054 8.792 0.015

2457801.71561 8.815 0.015

2457801.72070 8.803 0.015

2457801.72582 8.826 0.014

2457801.73093 8.814 0.015

2457801.73598 8.835 0.015

2457801.74104 8.774 0.016

2457801.74607 8.769 0.016

2457804.74731 8.751 0.017

2457804.75134 8.771 0.016

2457804.75538 8.828 0.016

2457804.75939 8.820 0.017

2457804.76343 8.790 0.016

2457804.76747 8.790 0.017

2457805.73098 8.783 0.015

2457805.73438 8.801 0.016

2457805.73777 8.798 0.016

2457805.74116 8.778 0.016

2457805.74455 8.838 0.015

2457805.74793 8.784 0.018

2457817.49112 8.804 0.014

2457817.49124 8.772 0.015

2457817.49138 8.782 0.015

2457817.49151 8.793 0.014

2457817.49165 8.791 0.015

2457817.49177 8.791 0.014

2457817.49192 8.786 0.014

2457817.49204 8.799 0.014

2457817.49218 8.787 0.014

2457817.49231 8.816 0.014

2457817.49244 8.768 0.015

2457817.49258 8.777 0.014

2457817.49270 8.803 0.014

2457817.49283 8.768 0.015

2457817.49297 8.797 0.014

2457817.49310 8.799 0.014

2457817.49323 8.773 0.014

2457817.49336 8.780 0.015

2457817.49350 8.775 0.014

2457817.49364 8.780 0.014

2457817.49378 8.767 0.014

2457817.49391 8.751 0.014

2457817.49405 8.790 0.014

2457817.49418 8.774 0.014

2457817.49431 8.787 0.014

2457817.49445 8.798 0.014

2457817.49459 8.764 0.014

2457817.49472 8.783 0.014

2457817.49486 8.790 0.014

2457817.49498 8.784 0.014

2457817.49512 8.790 0.014

2457817.49525 8.765 0.014

2457817.49538 8.780 0.014

2457817.49552 8.778 0.014

2457817.49564 8.775 0.014

2457817.49578 8.773 0.014

2457817.49592 8.797 0.014

2457817.49604 8.788 0.014

2457817.49616 8.781 0.014

2457817.49630 8.787 0.014

2457817.49643 8.771 0.013

2457817.49657 8.771 0.014

2457817.49671 8.760 0.014

2457817.49683 8.775 0.014

2457817.49695 8.784 0.014

2457817.49708 8.787 0.014

2457817.49720 8.766 0.014

2457817.49734 8.783 0.014

2457817.49748 8.783 0.014

2457817.49760 8.764 0.014

2457817.49774 8.783 0.014

2457817.49788 8.811 0.013

2457817.49799 8.766 0.014

2457817.49813 8.743 0.014

2457817.49827 8.792 0.014

2457817.49839 8.781 0.014

2457817.49852 8.740 0.014

2457817.49866 8.775 0.014

2457817.49878 8.783 0.014

2457817.49892 8.765 0.014

2457817.49906 8.774 0.014

2457817.49918 8.786 0.014

2457817.49932 8.776 0.014

2457817.49946 8.778 0.014

2457817.49960 8.769 0.014

2457817.49972 8.791 0.014

2457817.49986 8.780 0.014

2457817.49999 8.755 0.014

2457817.50011 8.788 0.014

2457817.50025 8.782 0.014

2457817.50038 8.783 0.014

2457817.50050 8.763 0.014

2457817.50064 8.792 0.013

2457817.50078 8.758 0.014

2457817.50092 8.769 0.013

2457817.50104 8.762 0.014

2457817.50119 8.768 0.014

2457817.50130 8.772 0.014

2457817.50144 8.763 0.014

2457817.50158 8.761 0.014

2457817.50171 8.779 0.014

2457817.50183 8.751 0.014

2457817.50197 8.807 0.014

2457817.50211 8.794 0.014

2457817.50224 8.781 0.014

2457817.50237 8.778 0.014

2457817.50251 8.792 0.014

2457817.50263 8.802 0.014

2457817.50277 8.758 0.014

2457817.50291 8.776 0.014

2457817.50305 8.775 0.014

2457817.50318 8.768 0.014

2457817.50330 8.751 0.013

2457817.50344 8.792 0.014

2457817.50357 8.750 0.014

2457817.50371 8.777 0.014

2457817.50384 8.791 0.014

2457817.50396 8.784 0.014

2457817.50409 8.760 0.014

2457817.50423 8.790 0.014

2457817.50436 8.786 0.014

2457817.50448 8.789 0.015

2457817.50461 8.764 0.015

2457817.50475 8.760 0.014

2457817.50488 8.772 0.014

2457817.50502 8.762 0.014

2457817.50514 8.792 0.014

2457817.50530 8.766 0.014

2457817.50543 8.803 0.013

2457817.50557 8.779 0.014

2457817.50571 8.787 0.014

2457817.50584 8.784 0.014

2457817.50597 8.794 0.014

2457817.50611 8.766 0.014

2457817.50623 8.736 0.014

2457817.50637 8.792 0.014

2457817.50673 8.768 0.014

2457817.50686 8.793 0.014

2457817.50698 8.810 0.014

2457817.50712 8.757 0.014

2457817.50725 8.800 0.013

2457817.50738 8.760 0.014

2457817.50751 8.794 0.014

2457817.50764 8.770 0.014

2457817.50777 8.779 0.013

2457817.50791 8.800 0.013

2457817.50804 8.744 0.014

2457817.50817 8.766 0.014

2457817.50829 8.791 0.013

2457817.50843 8.790 0.014

2457817.50857 8.759 0.015

2457817.50870 8.808 0.013

2457817.50883 8.773 0.013

2457817.50896 8.787 0.013

2457817.50909 8.786 0.014

2457817.50923 8.786 0.014

2457817.50936 8.759 0.014

2457817.50950 8.764 0.013

2457817.50962 8.778 0.013

2457817.50976 8.773 0.014

2457817.50988 8.762 0.014

2457817.51002 8.754 0.014

2457817.51016 8.754 0.014

2457817.51027 8.778 0.014

2457817.51041 8.789 0.014

2457817.51055 8.778 0.014

2457817.51069 8.760 0.014

2457817.51083 8.788 0.013

2457817.51097 8.775 0.014

2457817.51111 8.776 0.014

2457817.51123 8.775 0.014

2457817.51137 8.803 0.014

2457817.51151 8.797 0.013

2457817.51165 8.790 0.014

2457817.51179 8.760 0.014

2457817.51192 8.772 0.014

2457817.51204 8.782 0.014

2457817.51218 8.778 0.014

2457817.51232 8.770 0.014

2457817.51244 8.770 0.014

2457817.51258 8.779 0.014

2457817.51270 8.776 0.014

2457817.51283 8.758 0.014

2457817.51297 8.779 0.014

2457817.51311 8.784 0.014

2457817.51322 8.805 0.014

2457817.51336 8.785 0.014

2457817.51350 8.767 0.014

2457817.51363 8.793 0.013

2457817.51376 8.769 0.014

2457817.51389 8.776 0.013

2457817.51402 8.777 0.014

2457817.51416 8.766 0.014

2457817.51429 8.770 0.014

2457817.51442 8.812 0.014

2457817.51455 8.770 0.013

2457817.51468 8.784 0.014

2457817.51481 8.795 0.014

2457817.51494 8.789 0.014

2457817.51508 8.798 0.014

2457817.51519 8.785 0.013

2457817.51533 8.773 0.013

2457817.51547 8.770 0.014

2457817.51560 8.790 0.014

2457817.51573 8.751 0.014

2457817.51586 8.765 0.014

2457817.51600 8.756 0.014

2457817.51614 8.776 0.014

2457817.51628 8.770 0.013

2457817.51641 8.769 0.013

2457817.51655 8.763 0.014

2457817.51667 8.767 0.014

2457817.51680 8.787 0.013

2457817.51693 8.778 0.014

2457817.51707 8.768 0.014

2457817.51719 8.755 0.014

2457817.51733 8.741 0.014

2457817.51747 8.790 0.014

2457817.51761 8.784 0.013

2457817.51774 8.781 0.013

2457817.51788 8.802 0.014

2457817.51802 8.797 0.014

2457817.51814 8.775 0.014

2457817.51828 8.787 0.013

2457817.51842 8.769 0.014

2457817.51855 8.795 0.014

2457817.51869 8.758 0.014

2457817.51883 8.763 0.014

2457817.51896 8.792 0.014

2457817.51910 8.783 0.014

2457817.51924 8.781 0.014

2457817.51937 8.777 0.014

2457817.51950 8.794 0.014

2457817.51964 8.788 0.013

2457817.51976 8.778 0.014

2457817.51989 8.760 0.014

2457817.52003 8.791 0.014

2457817.52030 8.790 0.013

2457817.52042 8.800 0.014

2457817.52055 8.761 0.014

2457817.52068 8.783 0.013

2457817.52082 8.774 0.014

2457817.52095 8.765 0.014

2457817.52108 8.771 0.014

2457817.52121 8.806 0.014

2457817.52135 8.773 0.014

2457817.52148 8.784 0.013

2457817.52160 8.786 0.014

2457817.52174 8.782 0.014

2457817.52187 8.791 0.014

2457817.52202 8.796 0.014

2457817.52216 8.762 0.014

2457817.52230 8.792 0.014

2457817.52244 8.789 0.014

2457817.52258 8.788 0.014

2457817.52271 8.765 0.014

2457817.52285 8.797 0.014

2457817.52297 8.765 0.014

2457817.52311 8.780 0.014

2457817.52323 8.790 0.013

2457817.52337 8.770 0.014

2457817.52351 8.751 0.014

2457817.52363 8.774 0.014

2457817.52377 8.774 0.014

2457817.52391 8.778 0.014

2457817.52405 8.759 0.014

2457817.52417 8.826 0.014

2457817.52431 8.786 0.014

2457817.52445 8.769 0.014

2457817.52459 8.766 0.014

2457817.52473 8.786 0.014

2457817.52484 8.785 0.014

2457817.52498 8.795 0.014

2457817.52512 8.782 0.014

2457817.52526 8.777 0.014

2457817.52540 8.803 0.013

2457817.52553 8.758 0.014

2457817.52565 8.801 0.013

2457817.52579 8.789 0.014

2457817.52593 8.816 0.013

2457817.52607 8.767 0.014

2457817.52621 8.802 0.013

2457817.52635 8.769 0.013

2457817.52649 8.800 0.013

2457817.52661 8.788 0.013

2457817.52674 8.762 0.014

2457817.52688 8.790 0.014

2457817.52702 8.785 0.013

2457817.52716 8.786 0.013

2457817.52730 8.789 0.014

2457817.52744 8.792 0.013

2457817.52758 8.793 0.013

2457817.52773 8.786 0.014

2457817.52785 8.803 0.014

2457817.52799 8.794 0.014

2457817.52812 8.787 0.014

2457817.52825 8.801 0.014

2457817.52837 8.803 0.013

2457817.52852 8.786 0.013

2457817.52865 8.759 0.014

2457817.52880 8.782 0.014

2457817.52893 8.784 0.014

2457817.52907 8.776 0.014

2457817.52920 8.782 0.014

2457817.52933 8.759 0.014

2457817.52947 8.766 0.014

2457817.52960 8.776 0.013

2457817.52974 8.778 0.014

2457817.52987 8.793 0.013

2457817.52999 8.795 0.014

2457817.53013 8.784 0.013

2457817.53026 8.790 0.014

2457817.53039 8.767 0.014

2457817.53053 8.792 0.014

2457817.53067 8.797 0.013

2457817.53080 8.769 0.014

2457817.53093 8.791 0.014

2457817.53106 8.770 0.013

2457817.53119 8.741 0.014

2457817.53133 8.749 0.013

2457817.53146 8.750 0.014

2457817.53160 8.782 0.014

2457817.53173 8.776 0.014

2457817.53187 8.795 0.014

2457817.53201 8.755 0.014

2457817.53215 8.780 0.013

2457817.53229 8.791 0.013

2457817.53241 8.782 0.014

2457817.53255 8.759 0.014

2457817.53269 8.783 0.014

2457817.53282 8.764 0.014

2457817.53297 8.787 0.014

2457817.53311 8.799 0.014

2457817.53325 8.746 0.014

2457817.53337 8.792 0.014

2457817.53351 8.772 0.014

2457817.53364 8.771 0.014

2457817.53378 8.802 0.014

2457817.53391 8.768 0.014

2457817.53403 8.783 0.014

2457817.53417 8.796 0.014

2457817.53431 8.782 0.014

2457817.53445 8.772 0.014

2457817.53459 8.801 0.014

2457817.53473 8.761 0.014

2457817.53487 8.779 0.014

2457817.53499 8.798 0.013

2457817.53513 8.767 0.014

2457817.53527 8.787 0.014

2457817.53541 8.766 0.013

2457817.53555 8.805 0.014

2457817.53570 8.784 0.013

2457817.53583 8.785 0.014

2457817.53597 8.796 0.014

2457817.53611 8.768 0.014

2457817.53623 8.799 0.014

2457817.53636 8.775 0.014

2457817.53649 8.775 0.014

2457817.53677 8.812 0.013

2457817.53689 8.781 0.013

2457817.53702 8.755 0.014

2457817.53715 8.785 0.014

2457817.53727 8.788 0.014

2457817.53742 8.800 0.014

2457817.53755 8.768 0.014

2457817.53769 8.802 0.014

2457817.53783 8.777 0.014

2457817.53796 8.797 0.013

2457817.53808 8.787 0.014

2457817.53821 8.792 0.013

2457817.53835 8.755 0.014

2457817.53849 8.760 0.014

2457817.53863 8.747 0.015

2457817.53877 8.782 0.014

2457817.53892 8.782 0.014

2457817.53905 8.783 0.013

2457817.53920 8.778 0.014

2457817.53933 8.762 0.014

2457817.53947 8.761 0.014

2457817.53961 8.740 0.015

2457817.53974 8.798 0.013

2457817.53989 8.776 0.014

2457817.54002 8.759 0.014

2457817.54016 8.754 0.014

2457817.54030 8.785 0.014

2457817.54042 8.768 0.013

2457817.54056 8.783 0.014

2457817.54070 8.771 0.014

2457817.54084 8.780 0.014

2457817.54097 8.751 0.014

2457817.54111 8.788 0.014

2457817.54124 8.807 0.014

2457817.54137 8.745 0.014

2457817.54150 8.764 0.014

2457817.54163 8.761 0.014

2457817.54175 8.752 0.014

2457817.54189 8.785 0.014

2457817.54202 8.783 0.013

2457817.54216 8.797 0.014

2457817.54230 8.770 0.014

2457817.54242 8.779 0.014

2457817.54256 8.779 0.013

2457817.54270 8.787 0.014

2457817.54284 8.771 0.014

2457817.54298 8.796 0.013

2457817.54311 8.796 0.013

2457817.54325 8.756 0.014

2457817.54339 8.760 0.014

2457817.54351 8.753 0.014

2457817.54365 8.787 0.014

2457817.54379 8.776 0.014

2457817.54393 8.735 0.014

2457817.54407 8.773 0.014

2457817.54421 8.780 0.014

2457817.54435 8.761 0.014

2457817.54448 8.766 0.014

2457817.54461 8.766 0.015

2457817.54475 8.749 0.014

2457817.54488 8.769 0.014

2457817.54501 8.770 0.015

2457817.54513 8.796 0.014

2457817.54527 8.786 0.014

2457817.54542 8.755 0.014

2457817.54556 8.762 0.014

2457817.54570 8.758 0.014

2457817.54583 8.779 0.014

2457817.54598 8.790 0.014

2457817.54612 8.763 0.014

2457817.54624 8.801 0.014

2457817.54639 8.817 0.014

2457817.54652 8.782 0.014

2457817.54667 8.778 0.014

2457817.54680 8.770 0.013

2457817.54694 8.779 0.014

2457817.54708 8.760 0.014

2457817.54722 8.770 0.013

2457817.54748 8.767 0.014

2457817.54761 8.763 0.014

2457817.54775 8.801 0.014

2457817.54789 8.770 0.014

2457817.54804 8.779 0.014

2457817.54818 8.762 0.015

2457817.54830 8.751 0.014

2457817.54845 8.769 0.014

2457817.54859 8.787 0.014

2457817.54873 8.786 0.013

2457817.54886 8.768 0.013

2457817.54900 8.764 0.014

2457817.54914 8.751 0.014

2457817.54928 8.767 0.014

2457817.54942 8.796 0.014

2457817.54955 8.784 0.014

2457817.54968 8.775 0.014

2457817.54982 8.774 0.014

2457817.54995 8.804 0.014

2457817.55009 8.770 0.014

2457817.55024 8.785 0.013

2457817.55038 8.761 0.014

2457817.55050 8.738 0.014

2457817.55064 8.784 0.013

2457817.55078 8.768 0.014

2457817.55091 8.781 0.014

2457817.55105 8.776 0.014

2457817.55119 8.779 0.014

2457817.55131 8.796 0.014

2457817.55144 8.757 0.014

2457817.55158 8.797 0.014

2457817.55173 8.789 0.014

2457817.55187 8.765 0.014

2457817.55201 8.780 0.014

2457817.55215 8.775 0.014

2457817.55227 8.773 0.014

2457817.55240 8.793 0.014

2457817.55253 8.781 0.013

2457817.55267 8.794 0.014

2457817.55281 8.782 0.014

2457817.55295 8.774 0.014

2457817.55308 8.752 0.014

2457817.55322 8.792 0.014

2457817.55336 8.776 0.014

2457817.55349 8.772 0.014

2457817.55364 8.769 0.014

2457817.55378 8.786 0.014

2457817.55392 8.763 0.014

2457817.55405 8.795 0.013

2457817.55418 8.753 0.014

2457817.55431 8.779 0.013

2457817.55444 8.782 0.014

2457817.55457 8.768 0.014

2457817.55470 8.776 0.013

2457817.55484 8.782 0.014

2457817.55499 8.769 0.013

2457817.55511 8.784 0.014

2457817.55524 8.775 0.014

2457817.55539 8.777 0.014

2457817.55553 8.780 0.014

2457817.55567 8.772 0.014

2457817.55580 8.785 0.014

2457817.55593 8.768 0.014

2457817.55606 8.787 0.014

2457817.55620 8.790 0.014

2457817.55634 8.811 0.013

2457817.55648 8.782 0.013

2457817.55660 8.774 0.014

2457817.55674 8.796 0.014

2457817.55688 8.782 0.014

2457817.55702 8.767 0.013

2457817.55716 8.773 0.014

2457817.55731 8.761 0.013

2457817.55745 8.773 0.013

2457817.55759 8.766 0.014

2457817.55773 8.768 0.014

2457817.55786 8.791 0.014

2457817.55802 8.745 0.014

2457817.55815 8.769 0.014

2457817.55829 8.810 0.014

2457817.55843 8.763 0.014

2457817.55857 8.821 0.013

2457817.55871 8.792 0.013

2457817.55884 8.772 0.014

2457817.55895 8.781 0.014

2457817.55908 8.761 0.014

2457817.55921 8.805 0.014

2457817.55933 8.785 0.014

2457817.55947 8.783 0.014

2457817.55962 8.765 0.014

2457817.55975 8.788 0.014

2457817.55988 8.762 0.014

2457817.56003 8.775 0.014

2457817.56017 8.804 0.014

2457817.56031 8.780 0.014

2457817.56045 8.808 0.014

2457817.56058 8.793 0.014

2457817.56072 8.790 0.013

2457817.56087 8.766 0.013

2457817.56101 8.788 0.014

2457817.56115 8.744 0.014

2457817.56129 8.785 0.014

2457817.56143 8.784 0.014

2457817.56157 8.780 0.014

2457817.56171 8.780 0.013

2457817.56183 8.803 0.014

2457817.56197 8.778 0.014

2457817.56211 8.765 0.013

2457817.56225 8.808 0.014

2457817.56239 8.814 0.014

2457817.56253 8.793 0.014

2457817.56268 8.767 0.014

2457817.56282 8.814 0.014

2457817.56296 8.784 0.014

2457817.56310 8.777 0.013

2457817.56323 8.788 0.013

2457817.56337 8.792 0.013

2457817.56350 8.824 0.014

2457817.56364 8.802 0.013

2457817.56378 8.772 0.014

2457817.56392 8.802 0.013

2457817.56405 8.791 0.014

2457817.56417 8.785 0.014

2457817.56430 8.764 0.014

2457817.56443 8.801 0.013

2457817.56455 8.777 0.013

2457817.56470 8.770 0.014

2457817.56484 8.781 0.014

2457817.56498 8.783 0.013

2457817.56512 8.766 0.014

2457817.56526 8.753 0.014

2457817.56540 8.790 0.014

2457817.56553 8.767 0.014

2457817.56567 8.769 0.014

2457817.56579 8.801 0.013

2457817.56591 8.765 0.014

2457817.56605 8.806 0.013

2457817.56619 8.777 0.014

2457817.56633 8.794 0.014

2457817.56648 8.772 0.014

2457817.56661 8.785 0.013

2457817.56675 8.801 0.013

2457817.56689 8.791 0.014

2457817.56703 8.782 0.014

2457817.56718 8.796 0.014

2457817.56730 8.784 0.014

2457817.56742 8.736 0.014

2457817.56756 8.787 0.014

2457817.56769 8.771 0.014

2457817.56783 8.776 0.014

2457817.56797 8.779 0.013

2457817.56811 8.784 0.013

2457817.56825 8.784 0.013

2457817.56839 8.780 0.013

2457817.56852 8.780 0.013

2457817.56866 8.777 0.013

2457817.56880 8.790 0.013

2457817.56895 8.765 0.014

2457817.56909 8.780 0.014

2457817.56922 8.816 0.013

2457817.56936 8.783 0.014

2457817.56950 8.752 0.014

2457817.56964 8.779 0.013

2457817.56977 8.762 0.014

2457817.56991 8.787 0.013

2457817.57004 8.806 0.013

2457817.57018 8.818 0.014

2457817.57033 8.816 0.014

2457817.57047 8.779 0.014

2457817.57061 8.797 0.013

2457817.57075 8.777 0.014

2457817.57089 8.767 0.014

2457817.57102 8.775 0.014

2457817.57116 8.787 0.013

2457817.57130 8.792 0.014

2457817.57145 8.809 0.014

2457817.57159 8.801 0.014

2457817.57173 8.788 0.013

2457817.57187 8.809 0.013

2457817.57201 8.788 0.014

2457817.57215 8.774 0.014

2457817.57227 8.786 0.013

2457817.57242 8.743 0.014

2457817.57256 8.802 0.014

2457817.57270 8.798 0.014

2457817.57284 8.792 0.013

2457817.57298 8.762 0.014

2457817.57312 8.815 0.014

2457817.57326 8.785 0.014

2457817.57340 8.799 0.014

2457817.57354 8.743 0.014

2457817.60498 8.771 0.014

2457817.60798 8.778 0.014

2457817.61095 8.784 0.014

2457817.61394 8.753 0.014

2457817.61693 8.788 0.014

2457817.62028 8.754 0.014

2457817.62327 8.759 0.014

2457817.62627 8.789 0.014

2457817.62927 8.776 0.014

2457817.63225 8.799 0.014

2457817.63526 8.801 0.014

2457817.63826 8.780 0.014

2457817.64123 8.768 0.015

2457817.64423 8.774 0.015

2457817.64722 8.786 0.014

2457817.65020 8.773 0.014

2457817.65320 8.779 0.014

2457817.65619 8.771 0.014

2457817.65918 8.781 0.014

2457817.66217 8.776 0.015

2457817.66517 8.769 0.015

2457817.66817 8.788 0.014

2457817.67115 8.772 0.015

2457817.67417 8.767 0.015

2457817.67717 8.755 0.015

2457817.68019 8.786 0.014

2457817.68319 8.779 0.015

2457817.68620 8.775 0.015

2457817.68920 8.760 0.015

2457817.69219 8.786 0.015

2457817.69519 8.769 0.015

2457817.69819 8.782 0.015

2457817.70119 8.769 0.016

2457817.70420 8.772 0.015

2457817.70722 8.787 0.015

2457817.71021 8.789 0.015

2457817.71322 8.787 0.015

2457817.71623 8.782 0.015

2457817.71923 8.789 0.016

2457817.72226 8.802 0.016

2457817.72526 8.774 0.016

2457817.72825 8.770 0.016

2457817.73126 8.747 0.016

2457817.73428 8.794 0.016

2457818.49135 8.785 0.015

2457818.49293 8.809 0.014

2457818.49454 8.796 0.014

2457818.49614 8.775 0.014

2457818.49774 8.780 0.015

2457818.49933 8.740 0.015

2457818.50094 8.729 0.015

2457818.50258 8.780 0.014

2457818.50418 8.771 0.014

2457818.50578 8.763 0.014

2457818.50741 8.787 0.014

2457818.50905 8.778 0.014

2457818.51069 8.790 0.014

2457818.51227 8.763 0.014

2457818.51388 8.773 0.014

2457818.51547 8.740 0.015

2457818.51707 8.771 0.014

2457818.51866 8.777 0.014

2457818.52027 8.805 0.014

2457818.52186 8.776 0.015

2457818.52347 8.760 0.014

2457818.52504 8.791 0.014

2457818.52665 8.805 0.014

2457818.52825 8.763 0.015

2457818.52984 8.808 0.014

2457818.53142 8.763 0.014

2457818.53300 8.772 0.014

2457818.53461 8.762 0.014

2457818.53620 8.758 0.014

2457818.53778 8.790 0.014

2457818.53939 8.785 0.014

2457818.54098 8.816 0.014

2457818.54258 8.785 0.015

2457818.54417 8.794 0.014

2457818.54577 8.808 0.014

2457818.54737 8.777 0.014

2457818.54895 8.802 0.014

2457818.55054 8.762 0.014

2457818.55214 8.797 0.014

2457818.55371 8.775 0.014

2457818.55530 8.752 0.014

2457818.55688 8.770 0.014

2457818.55848 8.789 0.014

2457818.56008 8.769 0.014

2457818.56166 8.781 0.014

2457818.56326 8.763 0.014

2457818.56486 8.765 0.014

2457818.56645 8.770 0.015

2457818.56804 8.755 0.025

2457818.56962 8.757 0.014

2457818.60290 8.768 0.014

2457818.60535 8.817 0.014

2457818.60782 8.814 0.014

2457818.61028 8.768 0.014

2457818.61275 8.788 0.015

2457818.61521 8.809 0.014

2457818.61768 8.773 0.014

2457818.62013 8.785 0.014

2457818.62260 8.771 0.014

2457818.62509 8.785 0.014

2457818.62754 8.772 0.015

2457818.63001 8.780 0.015

2457818.63246 8.757 0.015

2457818.63494 8.769 0.015

2457818.63740 8.766 0.015

2457818.63986 8.780 0.015

2457818.64231 8.785 0.014

2457818.64497 8.770 0.015

2457818.64764 8.776 0.015

2457818.65032 8.791 0.014

2457818.65298 8.779 0.015

2457818.65564 8.778 0.015

2457818.65830 8.802 0.015

2457818.66099 8.779 0.016

2457818.66365 8.794 0.016

2457818.66634 8.777 0.015

2457818.66901 8.772 0.016

2457818.67167 8.749 0.016

2457818.67435 8.816 0.015

2457818.67701 8.760 0.016

2457818.67968 8.758 0.016

2457818.68239 8.774 0.016

2457818.68506 8.749 0.017

2457818.68776 8.816 0.015

2457818.69041 8.779 0.016

2457818.69308 8.782 0.016

2457818.69576 8.825 0.016

2457818.69847 8.817 0.017

2457818.70113 8.819 0.017

2457818.70379 8.785 0.017

2457818.70646 8.775 0.019

2457818.70914 8.773 0.019

2457818.71180 8.770 0.019

2457818.71450 8.818 0.019

2457818.71718 8.748 0.020

2457818.71987 8.800 0.017

2457818.72254 8.779 0.017

2457818.72523 8.733 0.018

2457818.72791 8.793 0.019

2457818.73058 8.752 0.019

2457818.73328 8.792 0.020

2457819.48966 9.010 0.015

2457819.49055 9.026 0.015

2457819.49146 8.987 0.015

2457819.49237 8.998 0.015

2457819.49327 9.016 0.016

2457819.49417 8.989 0.016

2457819.49506 8.962 0.016

2457819.49597 8.988 0.017

2457819.49686 9.002 0.017

2457819.49776 8.962 0.021

2457819.49866 8.933 0.021

2457819.49957 8.970 0.020

2457819.50047 9.011 0.021

2457819.50136 8.998 0.038

2457819.50226 9.016 0.025

2457819.50317 8.899 0.070

2457819.52755 8.972 0.072

2457819.52844 9.024 0.047

2457819.52936 8.922 0.039

2457819.53025 8.961 0.042

2457819.53115 8.922 0.035

2457819.53205 8.903 0.031

2457819.53296 8.870 0.032

2457819.53385 8.846 0.029

2457819.53475 8.839 0.025

2457819.53564 8.911 0.025

2457819.53655 8.873 0.026

2457819.53745 8.921 0.029

2457819.53836 8.883 0.026

2457819.53927 8.902 0.023

2457819.54016 8.870 0.022

2457819.54107 8.812 0.025

2457819.54196 8.872 0.022

2457819.54286 8.873 0.021

2457819.54377 8.879 0.021

2457819.54467 8.884 0.025

2457819.54556 8.837 0.028

2457819.54646 8.855 0.024

2457819.54737 8.874 0.023

2457819.54827 8.843 0.022

2457819.54917 8.819 0.022

2457819.55008 8.807 0.024

2457819.55097 8.801 0.024

2457819.55189 8.857 0.027

2457819.55280 8.848 0.044

2457819.56273 8.917 0.030

2457819.56363 8.870 0.024

2457819.56453 8.841 0.022

2457819.56542 8.848 0.021

2457819.59932 8.802 0.018

2457819.60230 8.797 0.018

2457819.60528 8.796 0.017

2457819.60826 8.780 0.017

2457819.61123 8.836 0.016

2457819.61420 8.790 0.018

2457819.61717 8.809 0.020

2457819.62013 8.814 0.020

2457819.62310 8.757 0.022

2457819.62608 8.804 0.020

2457819.62907 8.788 0.020

2457819.63203 8.765 0.020

2457819.63502 8.809 0.019

2457819.63799 8.777 0.022

2457819.64101 8.780 0.021

2457819.64401 8.838 0.020

2457819.64697 8.750 0.025

2457819.64996 8.764 0.027

2457819.65296 8.823 0.031

2457819.65595 8.775 0.026

2457819.65894 8.783 0.021

2457819.66192 8.779 0.021

2457819.66490 8.794 0.021

2457819.66789 8.751 0.023

2457819.67085 8.815 0.022

2457819.67384 8.758 0.029

2457819.67683 8.848 0.027

2457819.67986 8.787 0.028

2457819.68282 8.848 0.027

2457819.68580 8.810 0.024

2457819.68878 8.859 0.024

2457819.69175 8.818 0.030

2457819.69474 8.790 0.033

2457819.69773 8.812 0.036

2457819.70071 8.833 0.031

2457819.70367 8.760 0.034

2457819.70667 8.751 0.030

2457819.70966 8.801 0.028

2457819.71263 8.761 0.029

2457819.71560 8.830 0.032

2457819.71858 8.803 0.035

2457819.72156 8.773 0.030

2457819.72455 8.771 0.032

2457819.72754 8.759 0.028

2457819.73053 8.799 0.025

2457820.48908 8.822 0.028

2457820.48998 8.864 0.028

2457820.49087 8.809 0.024

2457820.49179 8.784 0.026

2457820.49268 8.831 0.026

2457820.49357 8.860 0.027

2457820.49447 8.820 0.029

2457820.49536 8.813 0.028

2457820.49627 8.822 0.026

2457820.49717 8.803 0.022

2457820.49807 8.830 0.024

2457820.49898 8.805 0.024

2457820.49987 8.799 0.024

2457820.50077 8.794 0.022

2457820.50167 8.820 0.026

2457820.50256 8.866 0.024

2457820.50347 8.833 0.026

2457820.50436 8.828 0.027

2457820.50526 8.798 0.026

2457820.50616 8.853 0.028

2457820.50796 8.830 0.032

2457820.50886 8.858 0.030

2457820.50975 8.781 0.030

2457820.51065 8.849 0.031

2457820.51155 8.837 0.031

2457820.51245 8.780 0.032

2457820.51335 8.782 0.030

2457820.51424 8.775 0.030

2457820.51514 8.815 0.030

2457820.51605 8.850 0.028

2457820.51695 8.837 0.026

2457820.51785 8.800 0.026

2457820.51877 8.847 0.027

2457820.51968 8.819 0.026

2457820.52058 8.844 0.027

2457820.52149 8.763 0.029

2457820.52239 8.801 0.028

2457820.52329 8.815 0.028

2457820.52418 8.838 0.029

2457820.52509 8.867 0.027

2457820.52599 8.799 0.028

2457820.52688 8.769 0.026

2457820.52778 8.787 0.027

2457820.52870 8.805 0.025

2457820.52960 8.853 0.020

2457820.53050 8.827 0.019

2457820.53141 8.794 0.018

2457820.53232 8.808 0.017

2457820.53321 8.788 0.017

2457820.53413 8.834 0.017

2457820.53504 8.839 0.017

2457820.53593 8.800 0.021

2457820.53685 8.805 0.019

2457820.53774 8.786 0.018

2457820.53865 8.814 0.017

2457820.53955 8.832 0.016

2457820.54046 8.813 0.016

2457820.54136 8.787 0.016

2457820.54226 8.846 0.016

2457820.54318 8.809 0.017

2457820.54408 8.808 0.016

2457820.54498 8.821 0.016

2457820.54589 8.797 0.017

2457820.54680 8.806 0.018

2457820.54770 8.830 0.018

2457820.54862 8.833 0.018

2457820.54951 8.814 0.017

2457820.55042 8.813 0.018

2457820.55134 8.800 0.018

2457820.55225 8.794 0.018

2457820.55315 8.822 0.017

2457820.55406 8.794 0.018

2457820.55496 8.808 0.017

2457820.55586 8.810 0.019

2457820.55677 8.810 0.019

2457820.55767 8.787 0.021

2457820.55858 8.810 0.020

2457820.55950 8.808 0.019

2457820.56040 8.807 0.018

2457820.56130 8.851 0.017

2457820.56220 8.831 0.017

2457820.56311 8.839 0.017

2457820.56400 8.810 0.017

2457820.56491 8.836 0.018

2457820.59635 8.797 0.018

2457820.59933 8.813 0.016

2457820.60231 8.836 0.015

2457820.60528 8.796 0.015

2457820.60825 8.800 0.015

2457820.61122 8.812 0.015

2457820.61420 8.800 0.015

2457820.61718 8.840 0.014

2457820.62017 8.813 0.015

2457820.62312 8.808 0.015

2457820.62607 8.794 0.015

2457820.62905 8.808 0.015

2457820.63201 8.804 0.014

2457820.63499 8.813 0.015

2457820.63799 8.780 0.015

2457820.64095 8.793 0.015

2457820.64392 8.813 0.015

2457820.64688 8.777 0.015

2457820.64987 8.791 0.015

2457820.65284 8.814 0.014

2457820.65583 8.792 0.015

2457820.65880 8.794 0.015

2457820.66178 8.785 0.015

2457820.66475 8.804 0.015

2457820.66773 8.803 0.015

2457820.67070 8.812 0.015

2457820.67369 8.789 0.017

2457820.67667 8.798 0.017

2457820.68262 8.848 0.021

2457820.68560 8.820 0.025

2457820.68857 8.790 0.021

2457820.69157 8.800 0.020

2457820.69454 8.840 0.026

2457820.69753 8.815 0.077

2457820.70050 8.771 0.076

2457820.70644 8.826 0.039

2457820.71238 8.818 0.028

2457820.71535 8.792 0.024

2457820.71835 8.808 0.022

2457820.72134 8.780 0.018

2457820.72436 8.785 0.017

2457820.72734 8.797 0.017

2457821.48854 8.805 0.015

2457821.49023 8.863 0.015

2457821.49192 8.818 0.015

2457821.49363 8.796 0.015

2457821.49533 8.803 0.015

2457821.49704 8.809 0.015

2457821.49873 8.826 0.015

2457821.50045 8.824 0.014

2457821.50214 8.811 0.014

2457821.50381 8.812 0.015

2457821.50550 8.807 0.015

2457821.50722 8.790 0.015

2457821.50892 8.810 0.015

2457821.51061 8.781 0.015

2457821.51232 8.818 0.015

2457821.51401 8.835 0.015

2457821.51572 8.815 0.014

2457821.51744 8.780 0.015

2457821.51914 8.781 0.015

2457821.52086 8.797 0.015

2457821.52259 8.788 0.015

2457821.52429 8.797 0.015

2457821.52599 8.821 0.015

2457821.52769 8.822 0.015

2457821.52939 8.810 0.015

2457821.53109 8.791 0.015

2457821.53280 8.815 0.015

2457821.53448 8.815 0.015

2457821.53619 8.812 0.014

2457821.53790 8.813 0.014

2457821.53961 8.805 0.015

2457821.54131 8.819 0.015

2457821.54302 8.831 0.015

2457821.54472 8.791 0.016

2457821.54643 8.815 0.014

2457821.54813 8.808 0.015

2457821.54984 8.815 0.015

2457821.55155 8.790 0.015

2457821.55325 8.810 0.015

2457821.55496 8.817 0.015

2457821.55668 8.808 0.015

2457821.55839 8.802 0.015

2457821.56009 8.786 0.016

2457821.56180 8.808 0.015

2457821.59371 8.795 0.015

2457821.59615 8.779 0.015

2457821.59861 8.793 0.015

2457821.60107 8.800 0.015

2457821.60354 8.823 0.015

2457821.60569 8.783 0.016

2457821.60784 8.789 0.015

2457821.60998 8.795 0.015

2457821.61215 8.793 0.015

2457821.61429 8.785 0.016

2457821.61645 8.783 0.015

2457821.61862 8.816 0.015

2457821.62078 8.813 0.015

2457821.62292 8.818 0.015

2457821.62506 8.805 0.015

2457821.62723 8.770 0.015

2457821.62940 8.844 0.015

2457821.63158 8.775 0.016

2457821.63373 8.799 0.016

2457821.63590 8.792 0.015

2457821.63806 8.791 0.016

2457821.64020 8.809 0.016

2457821.64237 8.807 0.015

2457821.64453 8.787 0.015

2457821.64667 8.830 0.014

2457821.64881 8.778 0.016

2457821.65097 8.789 0.016

2457821.65310 8.788 0.016

2457821.65525 8.802 0.015

2457821.65740 8.820 0.016

2457821.65955 8.777 0.016

2457821.66170 8.791 0.016

2457821.66386 8.818 0.016

2457821.66600 8.818 0.015

2457821.66817 8.817 0.015

2457821.67034 8.782 0.016

2457821.67251 8.805 0.016

2457821.67464 8.807 0.016

2457821.67681 8.806 0.015

2457821.67894 8.826 0.016

2457821.68108 8.801 0.017

2457821.68323 8.826 0.017

2457821.68539 8.782 0.017

2457821.68755 8.829 0.016

2457821.68970 8.816 0.016

2457821.69187 8.854 0.016

2457821.69402 8.826 0.016

2457821.69619 8.815 0.017

2457821.69834 8.794 0.017

2457821.70048 8.829 0.016

2457821.70264 8.814 0.017

2457821.70482 8.804 0.018

2457821.70696 8.872 0.017

2457821.71127 8.851 0.016

2457821.71342 8.816 0.017

2457821.71773 8.834 0.018

2457821.71988 8.853 0.017

2457821.72205 8.864 0.018

2457821.72421 8.864 0.018

2457823.63146 8.777 0.018

2457823.63445 8.837 0.018

2457823.63742 8.805 0.016

2457823.64038 8.762 0.016

2457823.64334 8.763 0.016

2457823.64609 8.803 0.016

2457823.64885 8.772 0.017

2457823.65158 8.782 0.017

2457823.65433 8.821 0.017

2457823.65710 8.822 0.017

2457823.65988 8.787 0.016

2457823.66264 8.768 0.017

2457823.66540 8.765 0.017

2457823.66815 8.811 0.016

2457824.59351 8.800 0.016

2457824.59758 8.798 0.016

2457824.60137 8.774 0.015

2457824.60516 8.811 0.015

2457824.60895 8.800 0.015

2457824.61275 8.774 0.015

2457824.61652 8.782 0.017

2457824.62032 8.808 0.016

2457824.62410 8.788 0.016

2457824.62789 8.776 0.017

2457824.63168 8.781 0.017

2457824.63547 8.828 0.016

2457824.63925 8.767 0.020

2457824.64305 8.775 0.038

2457824.64683 8.806 0.029

2457824.65443 8.769 0.025

2457824.65821 8.782 0.019

2457824.66200 8.821 0.019

2457824.66579 8.821 0.016

2457824.66959 8.792 0.016

2457824.67337 8.810 0.016

2457824.67717 8.780 0.016

2457824.68095 8.814 0.015

2457824.68476 8.803 0.016

2457824.68855 8.806 0.016

2457824.69233 8.836 0.016

2457824.69613 8.787 0.017

2457824.69992 8.792 0.017

2457824.70373 8.770 0.017

2457824.70753 8.813 0.017

2457824.71134 8.786 0.018

2457824.71513 8.810 0.017

2457825.48620 8.808 0.015

2457825.48825 8.801 0.016

2457825.49030 8.816 0.015

2457825.49234 8.783 0.015

2457825.49440 8.789 0.015

2457825.49644 8.821 0.015

2457825.49850 8.807 0.015

2457825.50056 8.844 0.014

2457825.50262 8.822 0.015

2457825.50466 8.800 0.015

2457825.50672 8.802 0.014

2457825.50877 8.822 0.014

2457825.51082 8.825 0.015

2457825.51286 8.831 0.015

2457825.51491 8.826 0.014

2457825.51697 8.835 0.014

2457825.51902 8.838 0.014

2457825.52108 8.794 0.014

2457825.52313 8.824 0.014

2457825.52519 8.805 0.014

2457825.52724 8.822 0.014

2457825.52929 8.806 0.014

2457825.53135 8.824 0.014

2457825.53340 8.837 0.014

2457825.53545 8.830 0.014

2457825.53748 8.824 0.014

2457825.53954 8.811 0.015

2457825.54158 8.784 0.014

2457825.54363 8.805 0.014

2457825.54568 8.799 0.015

2457825.54776 8.807 0.014

2457825.54981 8.843 0.014

2457825.58249 8.802 0.014

2457825.58527 8.822 0.014

2457825.58806 8.791 0.015

2457825.59085 8.790 0.015

2457825.59363 8.833 0.014

2457825.59614 8.830 0.014

2457825.59864 8.828 0.015

2457825.60116 8.828 0.015

2457825.60365 8.832 0.014

2457825.60616 8.852 0.015

2457825.60867 8.832 0.014

2457825.61120 8.820 0.015

2457825.61370 8.828 0.015

2457825.61621 8.793 0.015

2457825.61871 8.818 0.014

2457825.62121 8.816 0.015

2457825.62372 8.796 0.015

2457825.62623 8.844 0.015

2457825.62873 8.819 0.014

2457825.63123 8.813 0.015

2457825.63373 8.834 0.015

2457825.63623 8.841 0.015

2457825.63874 8.788 0.016

2457825.64126 8.816 0.015

2457825.64377 8.806 0.015

2457825.64627 8.800 0.016

2457825.64877 8.812 0.015

2457825.65127 8.818 0.016

2457825.65377 8.806 0.015

2457825.65627 8.815 0.015

2457825.65876 8.803 0.015

2457825.66127 8.779 0.016

2457825.66376 8.795 0.016

2457825.66627 8.804 0.016

2457825.66876 8.774 0.016

2457825.67128 8.823 0.016

2457825.67378 8.804 0.016

2457825.67629 8.824 0.016

2457825.67879 8.802 0.016

2457825.68131 8.773 0.016

2457825.68381 8.822 0.016

2457825.68631 8.814 0.016

2457825.68881 8.845 0.016

2457825.69131 8.759 0.017

2457825.69381 8.835 0.017

2457825.69633 8.838 0.016

2457825.69885 8.787 0.017

2457825.70136 8.777 0.017

2457825.70389 8.836 0.017

2457825.70639 8.800 0.017

2457825.70892 8.834 0.017

2457825.71143 8.838 0.017

2457826.48504 8.896 0.015

2457826.48710 8.890 0.015

2457826.48915 8.886 0.014

2457826.49121 8.855 0.015

2457826.49326 8.864 0.015

2457826.49531 8.915 0.014

2457826.49737 8.888 0.015

2457826.49943 8.904 0.015

2457826.50148 8.887 0.015

2457826.50352 8.884 0.015

2457826.50557 8.910 0.014

2457826.50761 8.923 0.015

2457826.50966 8.892 0.015

2457826.51172 8.920 0.015

2457826.51377 8.899 0.015

2457826.51585 8.935 0.015

2457826.51791 8.896 0.015

2457826.51996 8.898 0.015

2457826.52201 8.934 0.014

2457826.52408 8.889 0.015

2457826.52614 8.931 0.015

2457826.52818 8.930 0.015

2457826.53024 8.919 0.015

2457826.53230 8.926 0.015

2457826.53436 8.948 0.015

2457826.53641 8.917 0.015

2457826.53847 8.916 0.015

2457826.54054 8.887 0.016

2457826.54260 8.908 0.015

2457826.54465 8.923 0.015

2457826.54670 8.960 0.015

2457826.57983 8.912 0.015

2457826.58263 8.899 0.015

2457826.58540 8.895 0.015

2457826.58817 8.917 0.015

2457826.59095 8.945 0.015

2457826.59344 8.924 0.016

2457826.59594 8.950 0.015

2457826.59845 8.921 0.015

2457826.60094 8.931 0.015

2457826.60344 8.930 0.015

2457826.60593 8.890 0.015

2457826.60842 8.925 0.015

2457826.61094 8.897 0.016

2457826.61347 8.888 0.016

2457826.61599 8.893 0.015

2457826.61850 8.922 0.016

2457826.62100 8.883 0.015

2457826.62352 8.863 0.015

2457826.62602 8.885 0.015

2457826.62852 8.880 0.015

2457826.63104 8.870 0.016

2457826.63355 8.868 0.015

2457826.63604 8.852 0.016

2457826.63856 8.853 0.015

2457826.64106 8.899 0.015

2457826.64355 8.882 0.015

2457826.64605 8.850 0.016

2457826.64856 8.838 0.016

2457826.65106 8.848 0.016

2457826.65358 8.843 0.016

2457826.65608 8.839 0.016

2457826.65861 8.811 0.016

2457826.66109 8.839 0.016

2457826.66361 8.811 0.017

2457826.66609 8.822 0.017

2457826.66862 8.837 0.016

2457826.67112 8.843 0.017

2457826.67363 8.804 0.016

2457826.67615 8.799 0.017

2457826.67865 8.813 0.016

2457826.68116 8.813 0.017

2457826.68366 8.875 0.016

2457826.68617 8.805 0.017

2457826.68870 8.785 0.016

2457826.69121 8.794 0.017

2457826.69372 8.785 0.017

2457826.69623 8.847 0.017

2457826.69872 8.769 0.017

2457826.70122 8.799 0.017

2457826.70374 8.793 0.016

2457826.70627 8.814 0.017

2457826.70877 8.807 0.018

2457827.48439 8.779 0.015

2457827.48585 8.765 0.014

2457827.48732 8.784 0.014

2457827.48879 8.794 0.015

2457827.49024 8.774 0.014

2457827.49171 8.783 0.015

2457827.49318 8.783 0.014

2457827.49464 8.775 0.015

2457827.49609 8.773 0.015

2457827.49756 8.791 0.015

2457827.49903 8.779 0.015

2457827.50050 8.804 0.014

2457827.50344 8.776 0.015

2457827.50490 8.801 0.014

2457827.50637 8.749 0.015

2457827.50784 8.794 0.014

2457827.50931 8.782 0.015

2457827.51079 8.771 0.015

2457827.51225 8.788 0.016

2457827.51372 8.732 0.016

2457827.51518 8.762 0.014

2457827.51664 8.780 0.015

2457827.51810 8.782 0.015

2457827.51954 8.770 0.015

2457827.52100 8.762 0.015

2457827.52248 8.776 0.014

2457827.52395 8.793 0.014

2457827.52542 8.768 0.015

2457827.52689 8.788 0.015

2457827.52836 8.797 0.014

2457827.52982 8.777 0.014

2457827.53129 8.758 0.015

2457827.53276 8.779 0.014

2457827.53422 8.763 0.015

2457827.53568 8.779 0.014

2457827.53715 8.789 0.014

2457827.53862 8.730 0.015

2457827.54009 8.769 0.014

2457827.54156 8.778 0.015

2457827.54303 8.780 0.015

2457827.54450 8.777 0.015

2457827.54595 8.778 0.015

2457827.57778 8.773 0.014

2457827.58056 8.775 0.014

2457827.58337 8.750 0.016

2457827.58614 8.786 0.015

2457827.58891 8.801 0.015

2457827.59142 8.763 0.014

2457827.59392 8.762 0.016

2457827.59641 8.760 0.015

2457827.59889 8.798 0.015

2457827.60138 8.776 0.015

2457827.60388 8.759 0.015

2457827.60638 8.768 0.015

2457827.60887 8.780 0.015

2457827.61137 8.737 0.015

2457827.61388 8.760 0.015

2457827.61642 8.747 0.015

2457827.61892 8.776 0.016

2457827.62143 8.789 0.015

2457827.62394 8.802 0.015

2457827.62645 8.784 0.015

2457827.62895 8.778 0.015

2457827.63145 8.785 0.015

2457827.63396 8.809 0.015

2457827.63646 8.801 0.015

2457827.63898 8.774 0.014

2457827.64148 8.786 0.015

2457827.64398 8.775 0.016

2457827.64646 8.760 0.016

2457827.64898 8.784 0.016

2457827.65149 8.790 0.015

2457827.65401 8.790 0.015

2457827.65651 8.791 0.016

2457827.65902 8.780 0.016

2457827.66153 8.803 0.015

2457827.66405 8.779 0.016

2457827.66657 8.779 0.017

2457827.66908 8.761 0.016

2457827.67159 8.807 0.016

2457827.67408 8.743 0.017

2457827.67658 8.770 0.016

2457827.67910 8.814 0.016

2457827.68160 8.781 0.017

2457827.68409 8.757 0.016

2457827.68661 8.774 0.016

2457827.68913 8.782 0.017

2457827.69163 8.779 0.017

2457827.69414 8.764 0.017

2457827.69666 8.784 0.016

2457827.69916 8.787 0.017

2457827.70167 8.741 0.017

2457827.70418 8.747 0.018

2457827.70668 8.782 0.018

2457830.48238 8.812 0.014

2457830.48355 8.841 0.014

2457830.48473 8.810 0.014

2457830.48591 8.795 0.014

2457830.48708 8.802 0.014

2457830.48825 8.797 0.014

2457830.48942 8.793 0.014

2457830.49057 8.780 0.015

2457830.49172 8.830 0.014

2457830.49290 8.824 0.014

2457830.49407 8.813 0.014

2457830.49524 8.833 0.014

2457830.49641 8.793 0.015

2457830.49758 8.797 0.014

2457830.49876 8.794 0.014

2457830.49992 8.826 0.015

2457830.50111 8.813 0.014

2457830.50227 8.830 0.014

2457830.50344 8.815 0.014

2457830.50462 8.807 0.014

2457830.50579 8.835 0.014

2457830.50696 8.811 0.014

2457830.50814 8.831 0.014

2457830.50932 8.797 0.015

2457830.51048 8.812 0.014

2457830.51165 8.816 0.014

2457830.51282 8.816 0.014

2457830.51399 8.806 0.014

2457830.51517 8.797 0.014

2457830.51634 8.820 0.014

2457830.51753 8.817 0.014

2457830.51870 8.826 0.014

2457830.51987 8.814 0.014

2457830.52104 8.797 0.014

2457830.52220 8.831 0.014

2457830.52337 8.797 0.014

2457830.52455 8.847 0.014

2457830.52572 8.838 0.014

2457830.52689 8.820 0.014

2457830.52807 8.809 0.014

2457830.52924 8.826 0.014

2457830.53040 8.809 0.014

2457830.53157 8.803 0.014

2457830.53275 8.837 0.014

2457830.53393 8.825 0.014

2457830.53511 8.849 0.014

2457830.53628 8.809 0.015

2457830.53745 8.823 0.014

2457830.57381 8.833 0.020

2457830.57822 8.795 0.015

2457830.58042 8.788 0.015

2457830.58263 8.819 0.015

2457830.58483 8.782 0.015

2457830.58703 8.806 0.015

2457830.58924 8.821 0.015

2457830.59143 8.806 0.015

2457830.59363 8.808 0.015

2457830.59582 8.813 0.015

2457830.59804 8.808 0.016

2457830.60023 8.810 0.015

2457830.60241 8.802 0.015

2457830.60462 8.817 0.015

2457830.60681 8.810 0.014

2457830.60902 8.809 0.015

2457830.61122 8.835 0.015

2457830.61343 8.800 0.015

2457830.61563 8.805 0.015

2457830.61786 8.792 0.015

2457830.62226 8.801 0.016

2457830.62447 8.791 0.015

2457830.62668 8.815 0.016

2457830.62888 8.805 0.016

2457830.63108 8.821 0.015

2457830.63329 8.815 0.014

2457830.63549 8.825 0.015

2457830.63769 8.815 0.016

2457830.63990 8.786 0.016

2457830.64211 8.832 0.015

2457830.64431 8.833 0.016

2457830.64649 8.840 0.015

2457830.64869 8.822 0.016

2457830.65091 8.790 0.016

2457830.65312 8.782 0.016

2457830.65530 8.809 0.016

2457830.65751 8.788 0.016

2457830.65968 8.812 0.016

2457830.66188 8.822 0.016

2457830.66409 8.816 0.016

2457830.66629 8.820 0.016

2457830.67075 8.837 0.016

2457830.67295 8.811 0.015

2457830.67514 8.816 0.016

2457830.67736 8.796 0.016

2457830.67954 8.851 0.016

2457830.68175 8.790 0.017

2457830.68398 8.774 0.017

2457830.68619 8.776 0.018

2457830.69058 8.851 0.023

2457831.48177 8.776 0.015

2457831.48293 8.785 0.015

2457831.48408 8.780 0.014

2457831.48525 8.769 0.014

2457831.48642 8.789 0.014

2457831.48759 8.782 0.014

2457831.48874 8.777 0.014

2457831.48990 8.789 0.015

2457831.49107 8.778 0.014

2457831.49223 8.773 0.014

2457831.49340 8.747 0.014

2457831.49455 8.760 0.014

2457831.49572 8.775 0.014

2457831.49690 8.776 0.014

2457831.49808 8.799 0.014

2457831.49924 8.792 0.014

2457831.50041 8.773 0.014

2457831.50159 8.781 0.014

2457831.50278 8.795 0.014

2457831.50395 8.780 0.014

2457831.50513 8.821 0.014

2457831.50631 8.792 0.014

2457831.50748 8.791 0.014

2457831.50866 8.788 0.014

2457831.50983 8.761 0.014

2457831.51101 8.783 0.014

2457831.51217 8.783 0.014

2457831.51335 8.800 0.014

2457831.51452 8.805 0.014

2457831.51569 8.790 0.014

2457831.51683 8.807 0.014

2457831.51802 8.748 0.015

2457831.51918 8.821 0.014

2457831.52034 8.827 0.014

2457831.52152 8.789 0.014

2457831.52267 8.807 0.014

2457831.52385 8.767 0.015

2457831.52502 8.809 0.015

2457831.52620 8.795 0.014

2457831.52737 8.764 0.014

2457831.52855 8.779 0.014

2457831.52973 8.786 0.014

2457831.53090 8.776 0.014

2457831.53208 8.777 0.015

2457831.53325 8.786 0.014

2457831.53442 8.789 0.014

2457831.56616 8.799 0.013

2457831.56836 8.785 0.014

2457831.57055 8.774 0.015

2457831.57274 8.781 0.014

2457831.57491 8.813 0.014

2457831.57711 8.776 0.014

2457831.57931 8.787 0.014

2457831.58150 8.815 0.014

2457831.58370 8.758 0.015

2457831.58591 8.820 0.014

2457831.58808 8.802 0.014

2457831.59030 8.785 0.015

2457831.59247 8.765 0.015

2457831.59467 8.788 0.015

2457831.59688 8.812 0.014

2457831.59910 8.753 0.015

2457831.60129 8.773 0.015

2457831.60349 8.782 0.014

2457831.60570 8.794 0.014

2457831.60789 8.784 0.014

2457831.61008 8.783 0.014

2457831.61226 8.749 0.015

2457831.61446 8.769 0.015

2457831.61666 8.801 0.015

2457831.61886 8.748 0.015

2457831.62104 8.772 0.015

2457831.62322 8.747 0.015

2457831.62543 8.778 0.015

2457831.62762 8.781 0.015

2457831.62982 8.808 0.015

2457831.63203 8.773 0.015

2457831.63423 8.752 0.015

2457831.63644 8.781 0.015

2457831.63865 8.767 0.015

2457831.64085 8.772 0.016

2457831.64306 8.785 0.015

2457831.64526 8.791 0.016

2457831.64745 8.767 0.016

2457831.64965 8.767 0.016

2457831.65188 8.800 0.015

2457831.65409 8.790 0.015

2457831.65630 8.799 0.015

2457831.65851 8.760 0.016

2457831.66071 8.775 0.015

2457831.66291 8.766 0.016

2457831.66513 8.772 0.016

2457831.66737 8.768 0.016

2457831.66954 8.781 0.016

2457831.67175 8.759 0.016

2457831.67395 8.798 0.016

2457831.67615 8.791 0.017

2457831.67836 8.805 0.016

2457831.68060 8.773 0.017

2457831.68280 8.768 0.017

2457831.68498 8.742 0.017

2457831.68718 8.770 0.017

2457831.68938 8.760 0.017

2457831.69158 8.758 0.018

2457831.69380 8.786 0.017

2457831.69600 8.765 0.017

2457832.48089 8.783 0.014

2457832.48207 8.795 0.014

2457832.48325 8.779 0.014

2457832.48443 8.784 0.015

2457832.48560 8.798 0.014

2457832.48677 8.758 0.014

2457832.48793 8.758 0.014

2457832.48910 8.762 0.014

2457832.49028 8.795 0.014

2457832.49143 8.799 0.014

2457832.49261 8.805 0.014

2457832.49378 8.765 0.014

2457832.49497 8.795 0.014

2457832.49614 8.770 0.014

2457832.49732 8.797 0.014

2457832.49848 8.802 0.014

2457832.49965 8.753 0.014

2457832.50079 8.788 0.014

2457832.50197 8.807 0.014

2457832.50314 8.770 0.014

2457832.50432 8.799 0.014

2457832.50547 8.774 0.014

2457832.50663 8.774 0.014

2457832.50780 8.792 0.014

2457832.50896 8.786 0.014

2457832.51012 8.779 0.014

2457832.51129 8.812 0.014

2457832.51246 8.779 0.014

2457832.51364 8.804 0.014

2457832.51482 8.792 0.014

2457832.51598 8.763 0.014

2457832.51717 8.796 0.014

2457832.51832 8.780 0.014

2457832.51948 8.763 0.014

2457832.52068 8.793 0.014

2457832.52186 8.782 0.013

2457832.52305 8.785 0.014

2457832.52423 8.790 0.014

2457832.52541 8.766 0.014

2457832.52658 8.743 0.014

2457832.52776 8.759 0.014

2457832.52893 8.773 0.014

2457832.53012 8.785 0.014

2457832.53128 8.771 0.014

2457832.53246 8.784 0.014

2457832.56335 8.772 0.014

2457832.56555 8.770 0.014

2457832.56774 8.807 0.014

2457832.56991 8.775 0.014

2457832.57214 8.783 0.014

2457832.57436 8.781 0.015

2457832.57656 8.763 0.014

2457832.57876 8.779 0.014

2457832.58097 8.778 0.014

2457832.58317 8.777 0.015

2457832.58536 8.774 0.015

2457832.58755 8.759 0.015

2457832.58975 8.760 0.014

2457832.59195 8.764 0.014

2457832.59420 8.758 0.015

2457832.59638 8.793 0.015

2457832.59858 8.795 0.014

2457832.60078 8.776 0.014

2457832.60299 8.750 0.014

2457832.60519 8.765 0.015

2457832.60741 8.787 0.014

2457832.60962 8.768 0.014

2457832.61182 8.762 0.015

2457832.61401 8.759 0.015

2457832.61620 8.763 0.015

2457832.61837 8.772 0.015

2457832.62060 8.761 0.015

2457832.62282 8.788 0.014

2457832.62501 8.793 0.015

2457832.62720 8.781 0.015

2457832.62943 8.792 0.015

2457832.63165 8.800 0.015

2457832.63387 8.769 0.015

2457832.63608 8.766 0.015

2457832.63826 8.804 0.015

2457832.64047 8.750 0.016

2457832.64268 8.787 0.015

2457832.64490 8.756 0.015

2457832.64711 8.792 0.015

2457832.64931 8.751 0.016

2457832.65151 8.735 0.016

2457832.65370 8.751 0.015

2457832.65592 8.762 0.016

2457832.65811 8.782 0.015

2457832.66031 8.745 0.016

2457832.66251 8.779 0.016

2457832.66473 8.801 0.016

2457832.66692 8.793 0.016

2457832.66913 8.801 0.016

2457832.67131 8.824 0.015

2457832.67352 8.781 0.017

2457832.67572 8.768 0.017

2457832.67792 8.763 0.016

2457832.68014 8.783 0.016

2457832.68236 8.824 0.016

2457832.68455 8.782 0.017

2457832.68678 8.772 0.016

2457832.68898 8.798 0.016

2457832.69117 8.787 0.016

2457832.69339 8.774 0.017
