# Supplementary material for: HD 66051, an eclipsing binary hosting a highly peculiar, HgMn-related star
Source: Sci Rep. 2017 Jul 19;7:5906. doi: 10.1038/s41598-017-05987-6 (PMC5517476; doi:10.1038/s41598-017-05987-6)
Supplement: Supplementary file 3 — Supplementary Dataset 3 [file 41598_2017_5987_MOESM3_ESM.doc]

HD66051, an eclipsing binary hosting a highly peculiar, HgMn-related star

Ewa Niemczura

Stefan Hümmerich

Fiorella Castelli

Ernst Paunzen

Klaus Bernhard

Franz-Josef Hambsch

Krzysztof Hełminiak

JD mag(V) mag_err

2457669.75438 8.886 0.021

2457669.75693 8.862 0.019

2457669.75948 8.840 0.018

2457669.76204 8.810 0.016

2457669.76461 8.826 0.015

2457669.76716 8.858 0.014

2457669.76971 8.835 0.014

2457669.77229 8.842 0.013

2457669.77486 8.830 0.013

2457669.77742 8.833 0.012

2457669.77999 8.855 0.011

2457669.78255 8.838 0.011

2457669.78509 8.859 0.010

2457669.78766 8.856 0.010

2457669.79030 8.873 0.010

2457669.79285 8.867 0.010

2457669.79541 8.858 0.009

2457669.79797 8.866 0.009

2457669.80053 8.879 0.009

2457669.80309 8.871 0.009

2457669.80565 8.863 0.009

2457669.80821 8.861 0.008

2457669.81075 8.884 0.008

2457669.81331 8.877 0.008

2457669.81586 8.878 0.008

2457669.81841 8.892 0.008

2457669.82100 8.889 0.008

2457669.82358 8.872 0.007

2457669.82612 8.878 0.008

2457669.82868 8.881 0.009

2457669.83124 8.893 0.007

2457669.83381 8.887 0.007

2457669.83639 8.885 0.007

2457669.83894 8.887 0.007

2457669.84149 8.890 0.007

2457669.84406 8.873 0.007

2457669.84662 8.874 0.007

2457669.84919 8.865 0.007

2457669.85176 8.877 0.007

2457669.85433 8.886 0.007

2457669.85690 8.876 0.007

2457669.85946 8.881 0.007

2457669.86200 8.881 0.007

2457669.86458 8.881 0.007

2457669.86733 8.878 0.007

2457669.87013 8.881 0.007

2457669.87289 8.862 0.007

2457669.87565 8.871 0.007

2457670.75285 8.755 0.020

2457670.75648 8.751 0.018

2457670.76015 8.736 0.016

2457670.76381 8.740 0.015

2457670.76745 8.744 0.014

2457670.77479 8.768 0.012

2457670.77845 8.718 0.011

2457670.78209 8.751 0.010

2457670.78576 8.717 0.010

2457670.78942 8.752 0.010

2457670.79307 8.775 0.010

2457670.79672 8.739 0.009

2457670.80038 8.755 0.008

2457670.80404 8.782 0.008

2457670.80771 8.751 0.008

2457670.81135 8.730 0.008

2457670.81501 8.749 0.008

2457670.81868 8.738 0.008

2457670.82234 8.731 0.008

2457670.82601 8.741 0.007

2457670.82968 8.753 0.007

2457670.83334 8.723 0.009

2457670.83713 8.737 0.007

2457670.84091 8.747 0.007

2457670.84471 8.749 0.007

2457670.84851 8.753 0.007

2457670.85230 8.756 0.007

2457670.85609 8.757 0.007

2457670.85986 8.746 0.007

2457670.86362 8.749 0.007

2457670.86740 8.762 0.006

2457670.87116 8.755 0.007

2457670.87494 8.750 0.006

2457671.74896 8.751 0.021

2457671.75170 8.754 0.019

2457671.75443 8.807 0.017

2457671.75715 8.758 0.016

2457671.75988 8.793 0.015

2457671.76263 8.758 0.014

2457671.76534 8.740 0.013

2457671.76808 8.762 0.013

2457671.77081 8.751 0.012

2457671.77354 8.752 0.011

2457671.77627 8.759 0.011

2457671.77900 8.766 0.010

2457671.78175 8.773 0.010

2457671.78447 8.761 0.010

2457671.78721 8.762 0.009

2457671.78993 8.751 0.009

2457671.79267 8.755 0.009

2457671.79542 8.763 0.009

2457671.79816 8.732 0.009

2457671.80090 8.750 0.008

2457671.80363 8.775 0.008

2457671.80637 8.737 0.008

2457671.80910 8.744 0.008

2457671.81183 8.751 0.008

2457671.81457 8.746 0.008

2457671.81731 8.741 0.007

2457671.82006 8.743 0.007

2457671.82280 8.748 0.007

2457671.82554 8.739 0.007

2457671.82829 8.748 0.007

2457671.83103 8.750 0.007

2457671.83376 8.738 0.007

2457671.83650 8.742 0.007

2457671.83924 8.743 0.007

2457671.84197 8.755 0.007

2457671.84472 8.732 0.007

2457671.84747 8.750 0.007

2457671.85022 8.768 0.007

2457671.85295 8.757 0.007

2457671.85569 8.743 0.007

2457671.85844 8.757 0.007

2457671.86138 8.747 0.007

2457671.86431 8.751 0.007

2457671.86725 8.757 0.006

2457671.87019 8.748 0.006

2457671.87313 8.767 0.006

2457672.74892 8.810 0.019

2457672.75164 8.792 0.017

2457672.75438 8.750 0.016

2457672.75712 8.781 0.015

2457672.76259 8.796 0.013

2457672.76531 8.765 0.013

2457672.76804 8.761 0.012

2457672.77079 8.760 0.011

2457672.77353 8.766 0.011

2457672.77625 8.735 0.010

2457672.77898 8.775 0.010

2457672.78171 8.774 0.010

2457672.78444 8.769 0.009

2457672.78718 8.755 0.009

2457672.78992 8.743 0.009

2457672.79266 8.752 0.009

2457672.79539 8.768 0.008

2457672.79813 8.763 0.008

2457672.80086 8.773 0.008

2457672.80360 8.788 0.008

2457672.80633 8.756 0.008

2457672.80906 8.768 0.008

2457672.81181 8.787 0.008

2457672.81453 8.763 0.008

2457672.81726 8.772 0.007

2457672.82001 8.743 0.007

2457672.82274 8.768 0.007

2457672.82549 8.752 0.007

2457672.82823 8.773 0.007

2457672.83098 8.767 0.007

2457672.83374 8.766 0.007

2457672.83646 8.782 0.007

2457672.83919 8.777 0.007

2457672.84194 8.790 0.007

2457672.84468 8.765 0.007

2457672.84741 8.773 0.007

2457672.85014 8.791 0.007

2457672.85289 8.770 0.007

2457672.85563 8.776 0.007

2457672.85855 8.765 0.007

2457672.86152 8.758 0.007

2457672.86446 8.774 0.007

2457672.86740 8.778 0.007

2457672.87034 8.780 0.007

2457672.87330 8.778 0.006

2457673.74337 8.760 0.021

2457673.74611 8.751 0.019

2457673.74883 8.766 0.018

2457673.75156 8.777 0.016

2457673.75429 8.765 0.015

2457673.75705 8.761 0.015

2457673.75979 8.768 0.014

2457673.76252 8.796 0.013

2457673.76799 8.790 0.012

2457673.77073 8.759 0.011

2457673.77620 8.766 0.010

2457673.77895 8.784 0.010

2457673.78169 8.766 0.010

2457673.78443 8.788 0.009

2457673.78716 8.782 0.009

2457673.78990 8.805 0.009

2457673.79264 8.778 0.009

2457673.79537 8.784 0.008

2457673.79809 8.762 0.008

2457673.80083 8.784 0.008

2457673.80359 8.783 0.008

2457673.80632 8.773 0.008

2457673.80904 8.777 0.008

2457673.81181 8.780 0.008

2457673.81453 8.779 0.008

2457673.81727 8.777 0.007

2457673.82002 8.756 0.008

2457673.82278 8.746 0.028

2457673.82550 8.788 0.008

2457673.82823 8.778 0.008

2457673.83096 8.769 0.007

2457673.83647 8.776 0.009

2457673.84191 8.782 0.012

2457673.84465 8.777 0.007

2457673.84741 8.774 0.008

2457673.85014 8.797 0.007

2457673.85287 8.786 0.007

2457673.85582 8.776 0.008

2457673.85877 8.786 0.007

2457673.86169 8.752 0.008

2457673.86465 8.777 0.007

2457673.86759 8.795 0.007

2457673.87053 8.773 0.007

2457673.87351 8.797 0.007

2457674.73928 8.727 0.022

2457674.74203 8.747 0.020

2457674.74476 8.786 0.018

2457674.74750 8.790 0.017

2457674.75023 8.779 0.016

2457674.75295 8.790 0.015

2457674.75568 8.750 0.014

2457674.75843 8.757 0.013

2457674.76115 8.758 0.013

2457674.76388 8.783 0.012

2457674.76661 8.752 0.011

2457674.76933 8.763 0.011

2457674.77207 8.766 0.010

2457674.77479 8.746 0.010

2457674.77752 8.757 0.010

2457674.78027 8.807 0.010

2457674.78301 8.728 0.010

2457674.78575 8.782 0.009

2457674.78850 8.764 0.009

2457674.79123 8.772 0.009

2457674.79394 8.769 0.008

2457674.79668 8.762 0.008

2457674.79940 8.760 0.008

2457674.80213 8.780 0.008

2457674.80487 8.795 0.008

2457674.80762 8.775 0.008

2457674.81036 8.769 0.008

2457674.81310 8.754 0.008

2457674.81583 8.754 0.008

2457674.81856 8.745 0.007

2457674.82130 8.770 0.007

2457674.82402 8.764 0.007

2457674.82674 8.776 0.007

2457674.82946 8.764 0.007

2457674.83220 8.751 0.007

2457674.83493 8.764 0.007

2457674.83767 8.765 0.007

2457674.84041 8.740 0.007

2457674.84314 8.732 0.007

2457674.84588 8.752 0.007

2457674.84865 8.754 0.007

2457674.85138 8.762 0.007

2457674.85431 8.743 0.007

2457674.85728 8.757 0.007

2457674.86021 8.750 0.007

2457674.86316 8.761 0.007

2457674.86608 8.759 0.006

2457674.86903 8.751 0.007

2457674.87196 8.748 0.007

2457675.73796 8.760 0.021

2457675.74219 8.782 0.019

2457675.74428 8.714 0.017

2457675.74638 8.803 0.016

2457675.74848 8.754 0.016

2457675.75058 8.765 0.015

2457675.75269 8.748 0.014

2457675.75478 8.780 0.013

2457675.75688 8.721 0.013

2457675.75897 8.720 0.013

2457675.76108 8.740 0.012

2457675.76319 8.757 0.012

2457675.76529 8.741 0.011

2457675.76740 8.742 0.011

2457675.76949 8.756 0.011

2457675.77159 8.777 0.010

2457675.77370 8.738 0.010

2457675.77581 8.758 0.010

2457675.77795 8.758 0.010

2457675.78006 8.753 0.010

2457675.78218 8.772 0.009

2457675.78428 8.741 0.009

2457675.78638 8.774 0.009

2457675.78847 8.726 0.009

2457675.79057 8.731 0.009

2457675.79267 8.733 0.009

2457675.79477 8.748 0.008

2457675.79686 8.756 0.008

2457675.79898 8.768 0.008

2457675.80110 8.749 0.008

2457675.80322 8.754 0.008

2457675.80530 8.779 0.008

2457675.80740 8.757 0.008

2457675.80949 8.730 0.008

2457675.81159 8.753 0.008

2457675.81368 8.738 0.008

2457675.81578 8.758 0.008

2457675.81788 8.744 0.007

2457675.81999 8.749 0.007

2457675.82211 8.744 0.007

2457675.82422 8.748 0.007

2457675.82634 8.741 0.007

2457675.82847 8.729 0.007

2457675.83060 8.751 0.007

2457675.83273 8.749 0.007

2457675.83486 8.749 0.007

2457675.83697 8.736 0.007

2457675.83906 8.749 0.007

2457675.84116 8.732 0.007

2457675.84325 8.746 0.007

2457675.84535 8.751 0.007

2457675.84744 8.760 0.007

2457675.84973 8.741 0.007

2457675.85199 8.762 0.007

2457675.85425 8.740 0.007

2457675.85649 8.763 0.007

2457675.85875 8.742 0.007

2457675.86102 8.751 0.008

2457675.86326 8.753 0.007

2457675.86556 8.747 0.008

2457675.86780 8.722 0.008

2457675.87008 8.729 0.008

2457675.87233 8.741 0.007

2457676.73303 8.803 0.023

2457676.73514 8.800 0.021

2457676.73725 8.754 0.020

2457676.73935 8.726 0.019

2457676.74145 8.752 0.018

2457676.74356 8.774 0.016

2457676.74565 8.731 0.016

2457676.74777 8.784 0.025

2457676.74987 8.797 0.014

2457676.75197 8.761 0.013

2457676.75409 8.743 0.013

2457676.75619 8.775 0.013

2457676.75829 8.769 0.012

2457676.76041 8.736 0.012

2457676.76251 8.737 0.011

2457676.76463 8.755 0.011

2457676.76672 8.766 0.011

2457676.76883 8.776 0.011

2457676.77095 8.771 0.010

2457676.77307 8.765 0.010

2457676.77515 8.778 0.011

2457676.77726 8.765 0.012

2457676.77936 8.770 0.009

2457676.78147 8.759 0.009

2457676.78358 8.736 0.009

2457676.78567 8.753 0.009

2457676.78778 8.774 0.009

2457676.78990 8.753 0.009

2457676.79199 8.751 0.009

2457676.79409 8.752 0.008

2457676.79620 8.761 0.008

2457676.79831 8.748 0.008

2457676.80039 8.780 0.008

2457676.80249 8.750 0.008

2457676.80461 8.751 0.008

2457676.80671 8.757 0.008

2457676.80881 8.758 0.008

2457676.81090 8.771 0.008

2457676.81300 8.782 0.008

2457676.81512 8.740 0.009

2457676.81723 8.780 0.008

2457676.81934 8.762 0.010

2457676.82145 8.765 0.007

2457676.82355 8.752 0.008

2457676.82564 8.772 0.008

2457676.82773 8.743 0.008

2457676.82983 8.790 0.008

2457676.83192 8.798 0.008

2457676.83403 8.780 0.007

2457676.83613 8.771 0.008

2457676.83824 8.797 0.008

2457676.84036 8.794 0.012

2457676.84247 8.824 0.007

2457676.84458 8.801 0.007

2457676.84686 8.791 0.007

2457676.84911 8.812 0.008

2457676.85137 8.814 0.009

2457676.85362 8.814 0.007

2457676.85587 8.833 0.009

2457676.85811 8.841 0.009

2457676.86038 8.841 0.008

2457676.86265 8.832 0.009

2457676.86490 8.883 0.016

2457676.86715 8.863 0.007

2457676.86942 8.867 0.008

2457676.87168 8.874 0.009

2457677.78116 8.769 0.009

2457677.78325 8.791 0.009

2457677.78536 8.782 0.009

2457677.78748 8.781 0.009

2457677.78958 8.780 0.009

2457677.79169 8.772 0.009

2457677.79378 8.758 0.009

2457677.79588 8.785 0.008

2457677.79797 8.767 0.008

2457677.80009 8.785 0.008

2457677.80221 8.769 0.008

2457677.80433 8.800 0.008

2457677.80644 8.809 0.008

2457677.80855 8.776 0.008

2457677.81065 8.795 0.008

2457677.81274 8.778 0.008

2457677.81484 8.793 0.008

2457677.81693 8.792 0.008

2457677.81903 8.780 0.007

2457677.82112 8.787 0.007

2457677.82323 8.780 0.007

2457677.82535 8.782 0.007

2457677.82747 8.785 0.007

2457677.82958 8.791 0.007

2457677.83168 8.788 0.007

2457677.83380 8.788 0.007

2457677.83591 8.784 0.007

2457677.83803 8.763 0.007

2457677.84014 8.781 0.007

2457677.84225 8.783 0.007

2457677.84451 8.784 0.007

2457677.84676 8.770 0.007

2457677.84903 8.778 0.007

2457677.85128 8.759 0.007

2457677.85353 8.768 0.007

2457677.85580 8.772 0.007

2457677.85804 8.774 0.007

2457677.86034 8.785 0.007

2457677.86258 8.771 0.007

2457677.86485 8.789 0.006

2457677.86711 8.778 0.007

2457677.86935 8.769 0.007

2457678.77844 8.788 0.009

2457678.78053 8.801 0.009

2457678.78265 8.803 0.009

2457678.78477 8.786 0.009

2457678.78688 8.784 0.009

2457678.78899 8.792 0.009

2457678.79108 8.786 0.008

2457678.79318 8.777 0.008

2457678.79528 8.805 0.008

2457678.79737 8.782 0.008

2457678.79947 8.774 0.008

2457678.80157 8.789 0.008

2457678.80369 8.784 0.008

2457678.80579 8.784 0.008

2457678.80789 8.786 0.008

2457678.81000 8.787 0.007

2457678.81213 8.798 0.007

2457678.81425 8.768 0.008

2457678.81637 8.791 0.007

2457678.81847 8.778 0.007

2457678.82059 8.777 0.007

2457678.82272 8.787 0.007

2457678.82483 8.775 0.007

2457678.82694 8.777 0.007

2457678.82906 8.774 0.007

2457678.83116 8.793 0.007

2457678.83325 8.786 0.007

2457678.83535 8.769 0.007

2457678.83744 8.776 0.007

2457678.83954 8.776 0.007

2457678.84178 8.783 0.007

2457678.84406 8.772 0.007

2457678.84631 8.787 0.007

2457678.84859 8.784 0.007

2457678.85083 8.772 0.007

2457678.85308 8.761 0.007

2457678.85534 8.768 0.007

2457678.85759 8.799 0.007

2457678.85984 8.783 0.007

2457678.86209 8.785 0.007

2457678.86435 8.783 0.007

2457678.86660 8.787 0.007

2457678.86884 8.776 0.007

2457680.77275 8.758 0.009

2457680.77485 8.745 0.009

2457680.77694 8.757 0.009

2457680.77905 8.757 0.008

2457680.78115 8.754 0.008

2457680.78324 8.762 0.008

2457680.78535 8.757 0.008

2457680.78744 8.748 0.008

2457680.78955 8.745 0.008

2457680.79164 8.755 0.008

2457680.79374 8.767 0.008

2457680.79583 8.751 0.008

2457680.79794 8.748 0.008

2457680.80005 8.740 0.008

2457680.80215 8.743 0.007

2457680.80424 8.774 0.007

2457680.80633 8.745 0.007

2457680.80843 8.772 0.007

2457680.81052 8.767 0.007

2457680.81262 8.743 0.007

2457680.81471 8.763 0.007

2457680.81681 8.760 0.007

2457680.81889 8.767 0.007

2457680.82098 8.745 0.007

2457680.82308 8.747 0.007

2457680.82517 8.749 0.007

2457680.82727 8.731 0.007

2457680.82938 8.751 0.007

2457680.83148 8.756 0.007

2457680.83356 8.751 0.007

2457680.83581 8.741 0.007

2457680.83806 8.755 0.007

2457680.84029 8.744 0.007

2457680.84253 8.755 0.007

2457680.84478 8.759 0.007

2457680.84704 8.748 0.007

2457680.84928 8.740 0.007

2457680.85153 8.754 0.007

2457680.85378 8.746 0.007

2457680.85603 8.753 0.007

2457680.85828 8.752 0.007

2457680.86052 8.753 0.007

2457680.86277 8.765 0.007

2457680.86500 8.747 0.007

2457680.86726 8.763 0.007

2457680.86957 8.754 0.007

2457681.76994 8.978 0.009

2457681.77205 8.959 0.009

2457681.77414 8.975 0.009

2457681.77624 8.950 0.009

2457681.77833 8.961 0.008

2457681.78043 8.943 0.008

2457681.78253 8.928 0.008

2457681.78463 8.926 0.008

2457681.78675 8.926 0.008

2457681.78885 8.939 0.008

2457681.79097 8.892 0.008

2457681.79308 8.913 0.008

2457681.79520 8.897 0.008

2457681.79731 8.889 0.008

2457681.79943 8.891 0.008

2457681.80154 8.865 0.007

2457681.80366 8.869 0.007

2457681.80576 8.860 0.007

2457681.80786 8.862 0.007

2457681.80997 8.841 0.007

2457681.81206 8.846 0.007

2457681.81416 8.847 0.007

2457681.81625 8.822 0.007

2457681.81837 8.823 0.007

2457681.82049 8.832 0.007

2457681.82259 8.817 0.007

2457681.82470 8.833 0.007

2457681.82679 8.821 0.007

2457681.82890 8.797 0.007

2457681.83101 8.804 0.007

2457681.83325 8.808 0.007

2457681.83552 8.798 0.007

2457681.83778 8.800 0.007

2457681.84003 8.793 0.007

2457681.84229 8.785 0.007

2457681.84455 8.780 0.007

2457681.84682 8.775 0.007

2457681.84906 8.786 0.007

2457681.85132 8.785 0.007

2457681.85356 8.764 0.006

2457681.85582 8.776 0.007

2457681.85807 8.770 0.007

2457681.86032 8.760 0.007

2457681.86258 8.761 0.007

2457681.86483 8.767 0.007

2457681.86715 8.760 0.007

2457682.76722 8.760 0.009

2457682.76933 8.785 0.009

2457682.77146 8.777 0.010

2457682.77358 8.776 0.009

2457682.77571 8.793 0.008

2457682.77781 8.783 0.008

2457682.77993 8.784 0.008

2457682.78205 8.786 0.008

2457682.78418 8.791 0.008

2457682.78630 8.780 0.008

2457682.78844 8.784 0.008

2457682.79053 8.790 0.009

2457682.79266 8.755 0.013

2457682.79480 8.772 0.009

2457682.79693 8.768 0.008

2457682.79905 8.778 0.008

2457682.80116 8.769 0.008

2457682.80330 8.793 0.009

2457682.80541 8.774 0.008

2457682.80752 8.777 0.008

2457682.80963 8.782 0.008

2457682.81175 8.779 0.008

2457682.81387 8.792 0.007

2457682.81600 8.781 0.008

2457682.81813 8.769 0.008

2457682.82025 8.787 0.008

2457682.82238 8.789 0.008

2457682.82451 8.765 0.007

2457682.82664 8.774 0.008

2457682.82875 8.785 0.008

2457682.83102 8.765 0.008

2457682.83330 8.774 0.007

2457682.83557 8.777 0.007

2457682.83788 8.778 0.008

2457682.84014 8.786 0.007

2457682.84238 8.784 0.008

2457682.84464 8.772 0.007

2457682.84690 8.789 0.007

2457682.84917 8.781 0.007

2457682.85144 8.785 0.007

2457682.85369 8.776 0.007

2457682.85598 8.783 0.007

2457682.85825 8.786 0.007

2457682.86051 8.799 0.007

2457682.86278 8.784 0.007

2457682.86512 8.791 0.007

2457682.86745 8.798 0.007

2457683.76443 8.791 0.009

2457683.76718 8.773 0.009

2457683.76993 8.779 0.008

2457683.77270 8.774 0.008

2457683.77544 8.785 0.008

2457683.77822 8.783 0.008

2457683.78096 8.772 0.008

2457683.78374 8.776 0.008

2457683.78648 8.768 0.008

2457683.78924 8.769 0.008

2457683.79199 8.757 0.008

2457683.79478 8.798 0.007

2457683.79753 8.786 0.007

2457683.80307 8.774 0.007

2457683.80581 8.787 0.007

2457683.80856 8.778 0.007

2457683.81132 8.779 0.007

2457683.81406 8.770 0.007

2457683.81681 8.789 0.007

2457683.81958 8.782 0.007

2457683.82236 8.763 0.007

2457683.82514 8.773 0.007

2457683.82808 8.758 0.007

2457683.83098 8.780 0.007

2457683.83389 8.779 0.007

2457683.83679 8.778 0.007

2457683.83970 8.779 0.007

2457683.84263 8.772 0.007

2457683.84554 8.773 0.006

2457683.84845 8.794 0.007

2457683.85134 8.765 0.006

2457683.85426 8.772 0.007

2457683.85716 8.768 0.007

2457683.86008 8.775 0.007

2457683.86303 8.782 0.006

2457683.86602 8.769 0.007

2457684.76178 8.739 0.009

2457684.76451 8.724 0.009

2457684.76726 8.758 0.008

2457684.77000 8.766 0.008

2457684.77274 8.756 0.008

2457684.77549 8.763 0.008

2457684.77823 8.769 0.008

2457684.78100 8.754 0.008

2457684.78375 8.758 0.008

2457684.78649 8.753 0.008

2457684.78924 8.744 0.008

2457684.79200 8.758 0.007

2457684.79476 8.761 0.007

2457684.79750 8.758 0.007

2457684.80024 8.748 0.007

2457684.80300 8.746 0.007

2457684.80574 8.751 0.007

2457684.80850 8.757 0.007

2457684.81124 8.752 0.007

2457684.81398 8.734 0.007

2457684.81672 8.744 0.007

2457684.81948 8.747 0.007

2457684.82222 8.753 0.007

2457684.82514 8.741 0.007

2457684.82807 8.754 0.007

2457684.83098 8.752 0.007

2457684.83392 8.751 0.007

2457684.83683 8.760 0.007

2457684.83976 8.753 0.007

2457684.84266 8.747 0.006

2457684.84559 8.762 0.006

2457684.84851 8.770 0.006

2457684.85145 8.761 0.006

2457684.85436 8.772 0.007

2457684.85730 8.751 0.007

2457684.86029 8.751 0.006

2457684.86329 8.751 0.006

2457684.86628 8.744 0.006

2457685.75984 8.748 0.009

2457685.76288 8.749 0.008

2457685.76590 8.745 0.008

2457685.76895 8.767 0.008

2457685.77197 8.748 0.008

2457685.77502 8.752 0.008

2457685.77809 8.750 0.008

2457685.78113 8.757 0.008

2457685.78418 8.740 0.008

2457685.78722 8.761 0.007

2457685.79027 8.754 0.007

2457685.79337 8.739 0.007

2457685.79648 8.754 0.007

2457685.79956 8.753 0.007

2457685.80264 8.749 0.007

2457685.80572 8.750 0.007

2457685.80883 8.761 0.007

2457685.81193 8.749 0.007

2457685.81502 8.750 0.007

2457685.81814 8.747 0.007

2457685.82125 8.749 0.007

2457685.82434 8.746 0.007

2457685.82742 8.741 0.007

2457685.83051 8.748 0.007

2457685.83361 8.751 0.007

2457685.83671 8.745 0.006

2457685.83981 8.738 0.007

2457685.84291 8.753 0.007

2457685.84601 8.763 0.007

2457685.84909 8.765 0.006

2457685.85220 8.758 0.006

2457685.85530 8.752 0.006

2457685.85847 8.753 0.006

2457685.86164 8.751 0.006

2457685.86479 8.758 0.006

2457686.75688 8.770 0.008

2457686.75968 8.769 0.008

2457686.76248 8.752 0.008

2457686.76528 8.760 0.008

2457686.76807 8.781 0.008

2457686.77087 8.777 0.008

2457686.77366 8.772 0.008

2457686.77644 8.767 0.007

2457686.77948 8.776 0.008

2457686.78252 8.773 0.007

2457686.78556 8.778 0.007

2457686.78858 8.764 0.007

2457686.79160 8.756 0.007

2457686.79462 8.760 0.007

2457686.79764 8.764 0.007

2457686.80067 8.774 0.007

2457686.80370 8.767 0.007

2457686.80674 8.775 0.007

2457686.80978 8.783 0.007

2457686.81280 8.784 0.007

2457686.81582 8.753 0.007

2457686.81887 8.760 0.007

2457686.82186 8.782 0.007

2457686.82487 8.780 0.007

2457686.82789 8.781 0.007

2457686.83091 8.775 0.007

2457686.83394 8.776 0.007

2457686.83696 8.765 0.007

2457686.83994 8.761 0.007

2457686.84296 8.763 0.007

2457686.84597 8.770 0.007

2457686.84899 8.771 0.007

2457686.85200 8.763 0.007

2457686.85503 8.769 0.006

2457686.85808 8.774 0.006

2457686.86112 8.773 0.006

2457686.86413 8.777 0.006

2457687.75403 8.793 0.008

2457687.75681 8.789 0.008

2457687.75961 8.774 0.008

2457687.76241 8.787 0.008

2457687.76520 8.798 0.008

2457687.76799 8.773 0.008

2457687.77079 8.767 0.008

2457687.77360 8.799 0.008

2457687.77662 8.803 0.007

2457687.77965 8.798 0.007

2457687.78266 8.793 0.007

2457687.78571 8.785 0.007

2457687.78873 8.797 0.007

2457687.79177 8.772 0.007

2457687.79480 8.783 0.007

2457687.79784 8.775 0.007

2457687.80087 8.790 0.007

2457687.80390 8.779 0.007

2457687.80694 8.770 0.007

2457687.80997 8.795 0.007

2457687.81299 8.797 0.007

2457687.81602 8.778 0.007

2457687.81904 8.800 0.007

2457687.82205 8.780 0.007

2457687.82507 8.784 0.007

2457687.82808 8.791 0.007

2457687.83109 8.786 0.007

2457687.83410 8.776 0.006

2457687.83712 8.785 0.006

2457687.84015 8.786 0.007

2457687.84316 8.794 0.007

2457687.84618 8.770 0.007

2457687.84920 8.794 0.007

2457687.85219 8.790 0.006

2457687.85519 8.777 0.006

2457687.85819 8.782 0.006

2457687.86119 8.785 0.006

2457687.86418 8.798 0.006

2457688.75133 8.823 0.009

2457688.75413 8.830 0.009

2457688.75692 8.862 0.008

2457688.75970 8.831 0.008

2457688.76250 8.824 0.008

2457688.76529 8.827 0.008

2457688.76808 8.862 0.008

2457688.77088 8.851 0.008

2457688.77392 8.851 0.008

2457688.77697 8.870 0.008

2457688.78001 8.870 0.007

2457688.78304 8.865 0.007

2457688.78606 8.875 0.007

2457688.78909 8.868 0.007

2457688.79212 8.865 0.007

2457688.79514 8.872 0.007

2457688.79818 8.875 0.007

2457688.80120 8.873 0.007

2457688.80422 8.863 0.007

2457688.80727 8.873 0.007

2457688.81030 8.865 0.007

2457688.81332 8.895 0.007

2457688.81634 8.878 0.007

2457688.81936 8.881 0.007

2457688.82237 8.881 0.007

2457688.82538 8.888 0.007

2457688.82839 8.882 0.007

2457688.83140 8.875 0.007

2457688.83441 8.898 0.007

2457688.83741 8.886 0.007

2457688.84044 8.884 0.006

2457688.84347 8.872 0.007

2457688.84649 8.902 0.007

2457688.84951 8.871 0.007

2457688.85253 8.886 0.006

2457688.85554 8.894 0.006

2457688.85855 8.883 0.006

2457688.86157 8.880 0.007

2457688.86458 8.871 0.006

2457689.74966 8.766 0.009

2457689.75362 8.725 0.008

2457689.75757 8.766 0.008

2457689.76153 8.732 0.008

2457689.76549 8.728 0.008

2457689.76943 8.742 0.008

2457689.77362 8.733 0.008

2457689.77781 8.766 0.007

2457689.78203 8.764 0.007

2457689.78605 8.752 0.007

2457689.79005 8.742 0.007

2457689.79406 8.747 0.007

2457689.79807 8.745 0.007

2457689.80211 8.772 0.007

2457689.80613 8.741 0.007

2457689.81016 8.746 0.007

2457689.81418 8.745 0.007

2457689.81819 8.753 0.007

2457689.82220 8.740 0.007

2457689.82622 8.752 0.007

2457689.83022 8.746 0.007

2457689.83427 8.749 0.007

2457689.83829 8.755 0.007

2457689.84230 8.752 0.007

2457689.84634 8.757 0.006

2457689.85038 8.749 0.006

2457689.85443 8.749 0.007

2457689.85845 8.748 0.006

2457689.86247 8.753 0.007

2457690.74623 8.740 0.009

2457690.74934 8.786 0.008

2457690.75248 8.781 0.008

2457690.75561 8.735 0.008

2457690.75875 8.732 0.008

2457690.76190 8.749 0.008

2457690.76502 8.761 0.008

2457690.76837 8.751 0.008

2457690.77172 8.769 0.007

2457690.77509 8.747 0.008

2457690.77845 8.770 0.007

2457690.78168 8.769 0.007

2457690.78485 8.755 0.007

2457690.78803 8.778 0.007

2457690.79125 8.753 0.007

2457690.79442 8.768 0.007

2457690.79762 8.761 0.007

2457690.80083 8.738 0.007

2457690.80399 8.756 0.007

2457690.80720 8.748 0.007

2457690.81038 8.754 0.007

2457690.81358 8.757 0.007

2457690.81676 8.754 0.007

2457690.81995 8.767 0.007

2457690.82313 8.740 0.007

2457690.82635 8.755 0.007

2457690.82951 8.747 0.007

2457690.83274 8.744 0.007

2457690.83594 8.753 0.007

2457690.83910 8.748 0.007

2457690.84230 8.749 0.007

2457690.84552 8.751 0.007

2457690.84869 8.744 0.007

2457690.85190 8.742 0.007

2457690.85512 8.754 0.007

2457690.85834 8.747 0.007

2457690.86157 8.748 0.007

2457691.74347 8.786 0.009

2457691.74660 8.758 0.009

2457691.74973 8.778 0.008

2457691.75286 8.774 0.008

2457691.75600 8.756 0.008

2457691.75913 8.758 0.008

2457691.76227 8.759 0.008

2457691.76561 8.779 0.007

2457691.76898 8.773 0.008

2457691.77233 8.778 0.007

2457691.77569 8.772 0.007

2457691.77888 8.770 0.007

2457691.78209 8.763 0.007

2457691.78530 8.781 0.007

2457691.78847 8.777 0.007

2457691.79167 8.781 0.007

2457691.79487 8.756 0.007

2457691.79804 8.758 0.007

2457691.80125 8.772 0.007

2457691.80444 8.764 0.007

2457691.80763 8.766 0.007

2457691.81080 8.775 0.007

2457691.81400 8.763 0.007

2457691.81719 8.768 0.007

2457691.82036 8.772 0.007

2457691.82356 8.773 0.007

2457691.82675 8.779 0.007

2457691.82997 8.774 0.006

2457691.83314 8.768 0.007

2457691.83633 8.771 0.006

2457691.83955 8.764 0.007

2457691.84273 8.765 0.007

2457691.84590 8.775 0.007

2457691.84911 8.773 0.007

2457691.85231 8.780 0.006

2457691.85551 8.777 0.006

2457691.85870 8.762 0.006

2457691.86188 8.770 0.006

2457692.74065 8.780 0.009

2457692.74380 8.765 0.008

2457692.74690 8.776 0.008

2457692.75001 8.788 0.008

2457692.75315 8.782 0.008

2457692.75625 8.790 0.008

2457692.75938 8.785 0.008

2457692.76274 8.785 0.008

2457692.76612 8.808 0.007

2457692.76947 8.804 0.007

2457692.77284 8.790 0.007

2457692.77601 8.793 0.007

2457692.77924 8.798 0.007

2457692.78241 8.794 0.007

2457692.78560 8.778 0.007

2457692.78877 8.783 0.007

2457692.79196 8.788 0.007

2457692.79516 8.772 0.007

2457692.79833 8.795 0.007

2457692.80153 8.805 0.007

2457692.80472 8.807 0.007

2457692.80791 8.781 0.007

2457692.81111 8.775 0.007

2457692.81427 8.780 0.007

2457692.81745 8.795 0.007

2457692.82066 8.787 0.007

2457692.82384 8.785 0.007

2457692.82704 8.782 0.007

2457692.83024 8.786 0.007

2457692.83344 8.787 0.006

2457692.83663 8.776 0.007

2457692.83984 8.781 0.007

2457692.84302 8.789 0.007

2457692.84619 8.782 0.006

2457692.84940 8.789 0.006

2457692.85260 8.786 0.006

2457692.85580 8.776 0.006

2457692.85896 8.795 0.006

2457692.86215 8.790 0.006

2457693.73789 8.754 0.009

2457693.74104 8.769 0.008

2457693.74420 8.753 0.008

2457693.74734 8.772 0.008

2457693.75047 8.738 0.008

2457693.75362 8.759 0.008

2457693.75676 8.784 0.008

2457693.76016 8.747 0.008

2457693.76354 8.786 0.007

2457693.76693 8.777 0.007

2457693.77031 8.779 0.007

2457693.77354 8.769 0.007

2457693.77674 8.770 0.007

2457693.77997 8.773 0.007

2457693.78316 8.770 0.007

2457693.78639 8.766 0.007

2457693.78959 8.750 0.007

2457693.79280 8.735 0.007

2457693.79602 8.757 0.007

2457693.79922 8.785 0.007

2457693.80244 8.740 0.007

2457693.80566 8.759 0.007

2457693.80890 8.785 0.007

2457693.81212 8.746 0.007

2457693.81531 8.763 0.007

2457693.81854 8.756 0.007

2457693.82176 8.769 0.006

2457693.82497 8.763 0.007

2457693.82818 8.760 0.007

2457693.83141 8.759 0.007

2457693.83463 8.754 0.007

2457693.83786 8.766 0.006

2457693.84108 8.770 0.006

2457693.84428 8.751 0.006

2457693.84749 8.763 0.007

2457693.85072 8.758 0.006

2457693.85394 8.765 0.006

2457693.85715 8.747 0.007

2457693.86037 8.768 0.006

2457694.73515 8.746 0.009

2457694.73830 8.763 0.008

2457694.74145 8.745 0.008

2457694.74461 8.727 0.008

2457694.74775 8.775 0.008

2457694.75089 8.759 0.008

2457694.75404 8.738 0.008

2457694.75741 8.756 0.007

2457694.76079 8.747 0.008

2457694.76421 8.748 0.008

2457694.76759 8.758 0.007

2457694.77082 8.770 0.007

2457694.77403 8.764 0.007

2457694.77723 8.757 0.007

2457694.78042 8.739 0.007

2457694.78363 8.757 0.007

2457694.78685 8.745 0.007

2457694.79006 8.758 0.007

2457694.79329 8.766 0.007

2457694.79650 8.744 0.007

2457694.79972 8.746 0.007

2457694.80293 8.753 0.007

2457694.80613 8.750 0.007

2457694.80936 8.737 0.007

2457694.81258 8.750 0.007

2457694.81580 8.753 0.007

2457694.81903 8.743 0.007

2457694.82223 8.771 0.007

2457694.82545 8.749 0.007

2457694.83188 8.755 0.006

2457694.83510 8.741 0.007

2457694.83832 8.747 0.006

2457694.84153 8.741 0.006

2457694.84476 8.728 0.007

2457694.84797 8.761 0.007

2457694.85119 8.765 0.006

2457694.85442 8.728 0.007

2457694.85765 8.740 0.006

2457694.86088 8.735 0.006

2457695.73223 8.749 0.009

2457695.73541 8.756 0.009

2457695.73855 8.751 0.008

2457695.74168 8.770 0.008

2457695.74483 8.770 0.008

2457695.74795 8.757 0.008

2457695.75106 8.762 0.008

2457695.75442 8.766 0.008

2457695.75780 8.774 0.007

2457695.76115 8.777 0.007

2457695.76453 8.774 0.007

2457695.76771 8.785 0.007

2457695.77090 8.763 0.007

2457695.77409 8.763 0.007

2457695.77726 8.779 0.007

2457695.78044 8.756 0.007

2457695.78362 8.762 0.007

2457695.78681 8.750 0.007

2457695.79000 8.756 0.007

2457695.79322 8.754 0.007

2457695.79638 8.748 0.007

2457695.79956 8.755 0.007

2457695.80277 8.752 0.007

2457695.80595 8.780 0.007

2457695.80917 8.761 0.007

2457695.81234 8.770 0.007

2457695.81552 8.750 0.007

2457695.81873 8.766 0.007

2457695.82191 8.775 0.006

2457695.82510 8.772 0.006

2457695.82826 8.784 0.006

2457695.83147 8.795 0.007

2457695.83469 8.791 0.007

2457695.83789 8.806 0.006

2457695.84106 8.805 0.006

2457695.84427 8.811 0.006

2457695.84745 8.820 0.006

2457695.85065 8.822 0.006

2457695.85387 8.832 0.007

2457695.85708 8.836 0.006

2457695.86029 8.855 0.007

2457696.72946 8.755 0.009

2457696.73265 8.795 0.009

2457696.73581 8.757 0.008

2457696.73897 8.795 0.008

2457696.74211 8.806 0.008

2457696.74523 8.789 0.008

2457696.74839 8.767 0.008

2457696.75176 8.786 0.008

2457696.75512 8.803 0.008

2457696.75847 8.753 0.008

2457696.76183 8.763 0.008

2457696.76505 8.789 0.007

2457696.76826 8.778 0.007

2457696.77144 8.783 0.007

2457696.77462 8.788 0.007

2457696.77779 8.777 0.007

2457696.78096 8.765 0.007

2457696.78413 8.778 0.007

2457696.78730 8.767 0.007

2457696.79049 8.777 0.007

2457696.79370 8.774 0.007

2457696.79688 8.772 0.007

2457696.80006 8.781 0.007

2457696.80328 8.786 0.007

2457696.80645 8.785 0.007

2457696.80964 8.772 0.007

2457696.81284 8.775 0.007

2457696.81602 8.774 0.007

2457696.81920 8.783 0.007

2457696.82242 8.771 0.007

2457696.82559 8.760 0.007

2457696.82878 8.773 0.007

2457696.83196 8.782 0.007

2457696.83517 8.781 0.007

2457696.83836 8.781 0.007

2457696.84156 8.773 0.007

2457696.84477 8.772 0.007

2457696.84796 8.767 0.007

2457696.85115 8.753 0.007

2457696.85435 8.761 0.007

2457696.85756 8.778 0.007

2457700.71935 9.035 0.009

2457700.72280 9.068 0.008

2457700.72626 9.050 0.008

2457700.72971 9.014 0.008

2457700.73318 9.028 0.008

2457700.73663 9.044 0.008

2457700.74007 9.014 0.008

2457700.74373 9.031 0.008

2457700.74738 9.014 0.007

2457700.75110 8.990 0.008

2457700.75478 8.981 0.007

2457700.75845 8.983 0.007

2457700.76214 8.960 0.007

2457700.76581 8.977 0.007

2457700.76950 8.950 0.007

2457700.77318 8.945 0.007

2457700.77688 8.934 0.007

2457700.78054 8.926 0.007

2457700.78421 8.911 0.007

2457700.78789 8.900 0.007

2457700.79155 8.877 0.007

2457700.79524 8.873 0.007

2457700.79891 8.872 0.007

2457700.80258 8.853 0.007

2457700.80626 8.840 0.007

2457700.80993 8.846 0.007

2457700.81360 8.823 0.007

2457700.81727 8.824 0.006

2457700.82093 8.815 0.007

2457700.82457 8.802 0.007

2457700.82823 8.798 0.007

2457700.83191 8.787 0.006

2457700.83557 8.791 0.007

2457700.83921 8.775 0.006

2457700.84288 8.775 0.007

2457700.84655 8.774 0.006

2457700.85022 8.752 0.007

2457700.85390 8.765 0.006

2457700.85758 8.752 0.006

2457701.71654 8.775 0.009

2457701.72001 8.780 0.009

2457701.72344 8.774 0.008

2457701.72690 8.783 0.008

2457701.73035 8.775 0.008

2457701.73382 8.785 0.008

2457701.73729 8.773 0.008

2457701.74096 8.784 0.008

2457701.74466 8.795 0.008

2457701.74832 8.774 0.007

2457701.75201 8.788 0.007

2457701.75569 8.777 0.007

2457701.75936 8.764 0.007

2457701.76304 8.782 0.007

2457701.76674 8.766 0.007

2457701.77043 8.783 0.007

2457701.77412 8.783 0.007

2457701.77780 8.783 0.007

2457701.78147 8.782 0.007

2457701.78514 8.784 0.007

2457701.78881 8.766 0.007

2457701.79245 8.767 0.007

2457701.79615 8.773 0.007

2457701.79981 8.779 0.007

2457701.80350 8.778 0.007

2457701.80715 8.769 0.007

2457701.81082 8.766 0.007

2457701.81450 8.781 0.007

2457701.81817 8.767 0.007

2457701.82183 8.783 0.007

2457701.82549 8.778 0.007

2457701.82916 8.780 0.007

2457701.83284 8.787 0.007

2457701.83648 8.775 0.007

2457701.84015 8.781 0.007

2457701.84383 8.767 0.007

2457701.84750 8.768 0.007

2457701.85117 8.769 0.007

2457702.71299 8.799 0.009

2457702.71576 8.780 0.009

2457702.71853 8.782 0.009

2457702.72133 8.804 0.009

2457702.72412 8.794 0.008

2457702.72690 8.786 0.008

2457702.72969 8.782 0.008

2457702.73245 8.751 0.008

2457702.73523 8.770 0.008

2457702.73800 8.778 0.008

2457702.74078 8.793 0.008

2457702.74355 8.761 0.008

2457702.74632 8.779 0.008

2457702.74910 8.778 0.008

2457702.75186 8.774 0.007

2457702.75742 8.791 0.007

2457702.76019 8.775 0.007

2457702.76296 8.768 0.007

2457702.76576 8.763 0.007

2457702.76854 8.764 0.007

2457702.77131 8.766 0.007

2457702.77412 8.764 0.007

2457702.77705 8.760 0.007

2457702.77998 8.781 0.007

2457702.78291 8.771 0.007

2457702.78584 8.779 0.007

2457702.78878 8.790 0.007

2457702.79170 8.771 0.007

2457702.79465 8.772 0.007

2457702.79757 8.779 0.007

2457702.80050 8.759 0.007

2457702.80344 8.765 0.007

2457702.80637 8.766 0.007

2457702.80928 8.779 0.007

2457702.81225 8.781 0.007

2457702.81521 8.776 0.007

2457702.81818 8.779 0.007

2457702.82120 8.780 0.007

2457702.82421 8.773 0.007

2457702.82720 8.759 0.007

2457702.83021 8.773 0.007

2457702.83321 8.769 0.007

2457702.83618 8.778 0.006

2457702.83916 8.768 0.007

2457702.84212 8.784 0.007

2457702.84514 8.768 0.006

2457702.84813 8.774 0.006

2457702.85112 8.767 0.007

2457703.71028 8.786 0.015

2457703.71304 8.796 0.016

2457703.71580 8.737 0.033

2457703.71859 8.759 0.009

2457703.72138 8.786 0.014

2457703.72416 8.745 0.023

2457703.72691 8.759 0.021

2457703.72970 8.752 0.027

2457703.73250 8.761 0.012

2457703.73527 8.765 0.014

2457703.73806 8.776 0.016

2457703.74083 8.775 0.014

2457703.74362 8.758 0.016

2457703.74641 8.756 0.013

2457703.74919 8.776 0.015

2457703.75199 8.755 0.011

2457703.75477 8.750 0.011

2457703.75756 8.756 0.014

2457703.76035 8.762 0.011

2457703.76310 8.735 0.009

2457703.76587 8.743 0.008

2457703.76862 8.755 0.007

2457703.77140 8.758 0.008

2457703.77434 8.751 0.008

2457703.77726 8.753 0.010

2457703.78019 8.750 0.011

2457703.78313 8.782 0.016

2457703.78605 8.763 0.014

2457703.78898 8.739 0.008

2457703.79192 8.744 0.007

2457703.79486 8.740 0.008

2457703.79778 8.750 0.007

2457703.80069 8.748 0.007

2457703.80363 8.758 0.007

2457703.80655 8.734 0.010

2457703.80953 8.753 0.008

2457703.81250 8.737 0.011

2457703.81550 8.758 0.008

2457703.81850 8.754 0.007

2457703.82149 8.747 0.007

2457703.82449 8.745 0.008

2457703.82748 8.748 0.007

2457703.83046 8.754 0.007

2457703.83345 8.761 0.008

2457703.83646 8.747 0.008

2457703.83941 8.758 0.007

2457703.84237 8.746 0.007

2457703.84538 8.733 0.008

2457703.84838 8.756 0.007

2457704.70836 8.762 0.009

2457704.71204 8.746 0.009

2457704.71573 8.735 0.009

2457704.71942 8.753 0.009

2457704.72311 8.749 0.008

2457704.72683 8.767 0.008

2457704.73051 8.763 0.008

2457704.73421 8.768 0.008

2457704.73792 8.764 0.008

2457704.74161 8.734 0.008

2457704.74537 8.781 0.007

2457704.74914 8.739 0.008

2457704.75288 8.747 0.008

2457704.75663 8.750 0.007

2457704.76039 8.761 0.007

2457704.76413 8.754 0.007

2457704.76791 8.754 0.007

2457704.77166 8.745 0.007

2457704.77543 8.745 0.007

2457704.77918 8.743 0.007

2457704.78294 8.760 0.007

2457704.78670 8.763 0.007

2457704.79049 8.754 0.007

2457704.79425 8.764 0.007

2457704.79801 8.756 0.007

2457704.80176 8.748 0.007

2457704.80558 8.750 0.007

2457704.80940 8.756 0.007

2457704.81319 8.759 0.007

2457704.81701 8.760 0.007

2457704.82086 8.740 0.007

2457704.82470 8.732 0.007

2457704.82852 8.740 0.007

2457704.83234 8.749 0.007

2457704.83616 8.743 0.007

2457704.83997 8.757 0.007

2457704.84380 8.751 0.006

2457704.84758 8.763 0.006

2457705.70470 8.779 0.009

2457705.70747 8.790 0.009

2457705.71023 8.769 0.009

2457705.71297 8.767 0.009

2457705.71576 8.794 0.009

2457705.71855 8.746 0.009

2457705.72134 8.763 0.009

2457705.72411 8.776 0.008

2457705.72689 8.781 0.008

2457705.72965 8.773 0.008

2457705.73243 8.774 0.008

2457705.73521 8.768 0.008

2457705.73799 8.798 0.008

2457705.74076 8.771 0.008

2457705.74353 8.758 0.008

2457705.74631 8.789 0.007

2457705.74909 8.768 0.008

2457705.75188 8.774 0.008

2457705.75464 8.747 0.008

2457705.75743 8.751 0.007

2457705.76020 8.751 0.008

2457705.76296 8.787 0.007

2457705.76573 8.773 0.007

2457705.76867 8.765 0.007

2457705.77161 8.761 0.007

2457705.77454 8.773 0.007

2457705.77748 8.770 0.007

2457705.78039 8.764 0.007

2457705.78331 8.778 0.007

2457705.78623 8.773 0.007

2457705.78917 8.777 0.007

2457705.79209 8.772 0.007

2457705.79501 8.761 0.007

2457705.79794 8.775 0.007

2457705.80086 8.768 0.007

2457705.80384 8.784 0.007

2457705.80682 8.764 0.007

2457705.80981 8.776 0.007

2457705.81280 8.771 0.007

2457705.81576 8.785 0.007

2457705.81876 8.761 0.007

2457705.82174 8.775 0.007

2457705.82471 8.769 0.007

2457705.82772 8.760 0.007

2457705.83069 8.765 0.007

2457705.83367 8.765 0.007

2457705.83666 8.773 0.007

2457705.83963 8.778 0.007

2457705.84263 8.756 0.007

2457705.84563 8.759 0.007

2457705.84867 8.768 0.007

2457707.69900 8.784 0.010

2457707.70155 8.747 0.009

2457707.70411 8.774 0.009

2457707.70668 8.807 0.009

2457707.70925 8.797 0.009

2457707.71179 8.803 0.009

2457707.71435 8.814 0.008

2457707.71690 8.778 0.009

2457707.71947 8.779 0.009

2457707.72203 8.803 0.008

2457707.72458 8.806 0.009

2457707.72713 8.820 0.008

2457707.72969 8.802 0.008

2457707.73227 8.830 0.008

2457707.73484 8.825 0.008

2457707.73738 8.820 0.008

2457707.73993 8.815 0.008

2457707.74250 8.839 0.008

2457707.74505 8.839 0.008

2457707.74759 8.833 0.008

2457707.75014 8.823 0.008

2457707.75270 8.840 0.008

2457707.75527 8.838 0.007

2457707.75784 8.847 0.008

2457707.76037 8.840 0.008

2457707.76306 8.860 0.007

2457707.76576 8.859 0.007

2457707.76848 8.855 0.007

2457707.77119 8.850 0.007

2457707.77390 8.874 0.007

2457707.77661 8.865 0.007

2457707.77931 8.859 0.007

2457707.78201 8.872 0.007

2457707.78472 8.871 0.007

2457707.78743 8.849 0.007

2457707.79013 8.884 0.007

2457707.79282 8.875 0.007

2457707.79554 8.890 0.007

2457707.79825 8.881 0.007

2457707.80096 8.872 0.007

2457707.80367 8.896 0.007

2457707.80639 8.876 0.007

2457707.80910 8.880 0.007

2457707.81182 8.889 0.007

2457707.81454 8.884 0.007

2457707.81726 8.883 0.007

2457707.81997 8.884 0.007

2457707.82267 8.878 0.007

2457707.82539 8.891 0.007

2457707.82810 8.885 0.007

2457707.83081 8.891 0.007

2457707.83352 8.885 0.007

2457707.83622 8.884 0.007

2457707.83891 8.875 0.007

2457707.84169 8.887 0.007

2457707.84447 8.874 0.007

2457707.84725 8.871 0.007

2457708.69622 8.753 0.010

2457708.69876 8.755 0.009

2457708.70132 8.765 0.009

2457708.70387 8.768 0.009

2457708.70639 8.749 0.009

2457708.70896 8.765 0.009

2457708.71148 8.769 0.009

2457708.71402 8.762 0.009

2457708.71659 8.770 0.008

2457708.71916 8.762 0.008

2457708.72171 8.774 0.008

2457708.72428 8.771 0.008

2457708.72683 8.768 0.008

2457708.72940 8.757 0.008

2457708.73196 8.762 0.008

2457708.73451 8.747 0.008

2457708.73708 8.768 0.008

2457708.73966 8.752 0.008

2457708.74221 8.762 0.008

2457708.74473 8.766 0.007

2457708.74729 8.773 0.008

2457708.74981 8.768 0.008

2457708.75237 8.756 0.008

2457708.75491 8.746 0.007

2457708.75744 8.751 0.007

2457708.76014 8.764 0.008

2457708.76284 8.760 0.007

2457708.76554 8.751 0.007

2457708.76825 8.760 0.007

2457708.77096 8.753 0.007

2457708.77366 8.735 0.007

2457708.77635 8.744 0.007

2457708.77907 8.754 0.007

2457708.78179 8.739 0.007

2457708.78451 8.761 0.007

2457708.78721 8.745 0.007

2457708.78990 8.743 0.007

2457708.79258 8.759 0.007

2457708.79529 8.753 0.008

2457708.79801 8.756 0.007

2457708.80073 8.772 0.008

2457708.80345 8.762 0.008

2457708.80617 8.753 0.008

2457708.80889 8.749 0.008

2457708.81161 8.752 0.008

2457708.81432 8.762 0.008

2457708.81703 8.739 0.007

2457708.81973 8.742 0.007

2457708.82244 8.755 0.008

2457708.82515 8.755 0.008

2457708.82786 8.740 0.007

2457708.83058 8.753 0.007

2457708.83326 8.742 0.008

2457708.83597 8.744 0.007

2457708.83872 8.755 0.007

2457708.84148 8.759 0.007

2457708.84426 8.752 0.007

2457708.84703 8.750 0.007

2457709.69346 8.746 0.009

2457709.69602 8.757 0.009

2457709.69856 8.753 0.009

2457709.70111 8.750 0.009

2457709.70366 8.762 0.009

2457709.70619 8.759 0.009

2457709.70874 8.765 0.009

2457709.71131 8.754 0.008

2457709.71384 8.756 0.008

2457709.71641 8.759 0.008

2457709.71897 8.760 0.008

2457709.72153 8.750 0.008

2457709.72409 8.766 0.008

2457709.72663 8.767 0.008

2457709.72918 8.745 0.008

2457709.73176 8.755 0.008

2457709.73433 8.760 0.008

2457709.73689 8.777 0.008

2457709.73943 8.755 0.007

2457709.74198 8.751 0.007

2457709.74450 8.756 0.008

2457709.74706 8.770 0.008

2457709.74959 8.755 0.007

2457709.75214 8.767 0.008

2457709.75469 8.765 0.007

2457709.75741 8.746 0.007

2457709.76010 8.749 0.007

2457709.76279 8.743 0.007

2457709.76550 8.752 0.007

2457709.76821 8.756 0.007

2457709.77093 8.747 0.007

2457709.77363 8.755 0.007

2457709.77634 8.754 0.007

2457709.77906 8.739 0.007

2457709.78177 8.742 0.007

2457709.78448 8.737 0.007

2457709.78719 8.745 0.007

2457709.78990 8.750 0.007

2457709.79260 8.748 0.007

2457709.79530 8.744 0.007

2457709.79799 8.755 0.007

2457709.80067 8.760 0.007

2457709.80336 8.746 0.007

2457709.80605 8.758 0.007

2457709.80876 8.742 0.007

2457709.81147 8.745 0.007

2457709.81418 8.755 0.007

2457709.81686 8.749 0.007

2457709.81956 8.740 0.007

2457709.82227 8.750 0.007

2457709.82498 8.752 0.007

2457709.82770 8.745 0.007

2457709.83038 8.735 0.007

2457709.83308 8.743 0.007

2457709.83586 8.741 0.007

2457709.83862 8.745 0.007

2457709.84140 8.760 0.007

2457709.84418 8.761 0.007

2457709.84697 8.755 0.007

2457710.69091 8.785 0.010

2457710.69359 8.770 0.010

2457710.69626 8.760 0.012

2457710.69895 8.792 0.014

2457710.70160 8.766 0.012

2457710.70426 8.778 0.014

2457710.70693 8.773 0.016

2457710.70959 8.741 0.012

2457710.71223 8.783 0.016

2457710.71488 8.797 0.013

2457710.71756 8.775 0.011

2457710.72023 8.772 0.011

2457710.72289 8.760 0.011

2457710.72554 8.809 0.009

2457710.72822 8.770 0.009

2457710.73088 8.777 0.010

2457710.73355 8.772 0.009

2457710.73620 8.799 0.011

2457710.73889 8.796 0.010

2457710.74154 8.754 0.010

2457710.74418 8.778 0.011

2457710.74683 8.774 0.010

2457710.74948 8.779 0.009

2457710.75213 8.774 0.009

2457710.75479 8.787 0.010

2457710.75744 8.778 0.010

2457710.76009 8.766 0.010

2457710.76274 8.757 0.011

2457710.76538 8.757 0.009

2457710.76803 8.786 0.010

2457710.77068 8.774 0.011

2457710.77332 8.778 0.011

2457710.77596 8.777 0.011

2457710.77859 8.769 0.009

2457710.78125 8.769 0.009

2457710.78389 8.770 0.009

2457710.78655 8.773 0.010

2457710.78919 8.779 0.009

2457710.79183 8.775 0.008

2457710.79448 8.782 0.008

2457710.79714 8.767 0.007

2457710.79979 8.773 0.008

2457710.80243 8.772 0.007

2457710.80509 8.776 0.007

2457710.80774 8.759 0.007

2457710.81039 8.761 0.007

2457710.81304 8.770 0.007

2457710.81568 8.767 0.007

2457710.81860 8.768 0.007

2457710.82154 8.765 0.007

2457710.82448 8.779 0.007

2457710.82742 8.767 0.007

2457710.83036 8.782 0.007

2457710.83329 8.761 0.007

2457710.83624 8.770 0.007

2457710.83919 8.781 0.007

2457710.84216 8.777 0.007

2457710.84497 8.781 0.007

2457710.84763 8.778 0.007

2457711.68738 8.777 0.009

2457711.68958 8.784 0.010

2457711.69178 8.788 0.009

2457711.69398 8.799 0.009

2457711.69617 8.792 0.009

2457711.69837 8.786 0.009

2457711.70057 8.798 0.009

2457711.70277 8.784 0.009

2457711.70498 8.785 0.009

2457711.70719 8.798 0.009

2457711.70941 8.773 0.008

2457711.71161 8.779 0.009

2457711.71382 8.793 0.009

2457711.71602 8.795 0.008

2457711.71824 8.819 0.008

2457711.72045 8.788 0.008

2457711.72267 8.788 0.008

2457711.72486 8.785 0.008

2457711.72706 8.784 0.008

2457711.72927 8.780 0.008

2457711.73147 8.791 0.008

2457711.73367 8.786 0.008

2457711.73588 8.776 0.008

2457711.73807 8.784 0.008

2457711.74028 8.804 0.008

2457711.74247 8.790 0.008

2457711.74466 8.773 0.008

2457711.74688 8.792 0.007

2457711.74909 8.786 0.007

2457711.75130 8.780 0.008

2457711.75351 8.789 0.007

2457711.75571 8.795 0.007

2457711.75792 8.768 0.007

2457711.76013 8.790 0.007

2457711.76234 8.797 0.007

2457711.76455 8.785 0.007

2457711.76675 8.781 0.007

2457711.76896 8.787 0.007

2457711.77117 8.786 0.007

2457711.77338 8.777 0.007

2457711.77559 8.775 0.007

2457711.77778 8.804 0.007

2457711.77999 8.770 0.007

2457711.78220 8.766 0.008

2457711.78440 8.754 0.011

2457711.78661 8.809 0.015

2457711.78880 8.774 0.017

2457711.79102 8.773 0.027

2457711.79321 8.783 0.015

2457711.79541 8.800 0.021

2457711.79760 8.809 0.023

2457711.80200 8.809 0.018

2457711.80420 8.792 0.019

2457711.80640 8.810 0.018

2457711.80860 8.792 0.013

2457711.81080 8.787 0.014

2457711.81299 8.784 0.015

2457711.81517 8.781 0.012

2457711.81737 8.815 0.010

2457711.81957 8.793 0.008

2457711.82177 8.785 0.008

2457711.82398 8.783 0.007

2457711.82617 8.786 0.008

2457711.82834 8.788 0.007

2457711.83053 8.790 0.008

2457711.83272 8.792 0.008

2457711.83492 8.783 0.008

2457711.83712 8.786 0.007

2457711.83944 8.793 0.007

2457711.84176 8.774 0.008

2457711.84391 8.788 0.009

2457711.84601 8.803 0.010

2457713.68182 8.734 0.009

2457713.68403 8.736 0.009

2457713.68623 8.742 0.009

2457713.68843 8.749 0.009

2457713.69061 8.752 0.008

2457713.69282 8.744 0.008

2457713.69502 8.751 0.008

2457713.69722 8.751 0.010

2457713.69944 8.776 0.008

2457713.70164 8.771 0.008

2457713.70384 8.734 0.008

2457713.70606 8.765 0.008

2457713.70828 8.767 0.008

2457713.71047 8.766 0.008

2457713.71269 8.767 0.008

2457713.71491 8.748 0.008

2457713.71712 8.736 0.008

2457713.71933 8.762 0.008

2457713.72152 8.747 0.008

2457713.72374 8.755 0.008

2457713.72597 8.752 0.007

2457713.72819 8.761 0.019

2457713.73484 8.739 0.010

2457713.75027 8.776 0.009

2457713.75690 8.760 0.008

2457713.75912 8.734 0.032

2457713.76354 8.741 0.011

2457713.76575 8.760 0.029

2457713.77676 8.717 0.027

2457713.79226 8.759 0.013

2457713.79666 8.803 0.050

2457713.79887 8.716 0.035

2457713.80109 8.779 0.029

2457713.80330 8.762 0.012

2457713.80552 8.746 0.010

2457713.80773 8.783 0.016

2457713.80994 8.781 0.059

2457713.81436 8.769 0.032

2457713.81656 8.719 0.009

2457713.81877 8.744 0.040

2457713.82098 8.761 0.019

2457713.82319 8.722 0.010

2457713.82541 8.758 0.008

2457713.82762 8.738 0.008

2457713.82981 8.763 0.010

2457713.83201 8.772 0.016

2457713.83435 8.738 0.011

2457713.83668 8.737 0.036

2457713.83882 8.794 0.033

2457713.84304 8.735 0.009

2457713.84515 8.753 0.010

2457714.74321 8.752 0.007

2457714.74541 8.759 0.007

2457714.74759 8.764 0.007

2457714.74977 8.737 0.007

2457714.75196 8.753 0.007

2457714.75414 8.750 0.007

2457714.75632 8.732 0.007

2457714.75851 8.755 0.007

2457714.76069 8.752 0.007

2457714.76288 8.742 0.007

2457714.76506 8.725 0.007

2457714.76723 8.733 0.007

2457714.76941 8.746 0.007

2457714.77159 8.747 0.007

2457714.77376 8.750 0.007

2457714.77594 8.737 0.007

2457714.77811 8.752 0.007

2457714.78030 8.746 0.007

2457714.78248 8.746 0.007

2457714.78465 8.756 0.007

2457714.78683 8.749 0.007

2457714.78900 8.741 0.007

2457714.79119 8.775 0.007

2457714.79338 8.747 0.007

2457714.79557 8.748 0.007

2457714.79774 8.757 0.007

2457714.79992 8.763 0.007

2457714.80209 8.746 0.007

2457714.80429 8.755 0.007

2457714.80648 8.748 0.007

2457714.80866 8.753 0.007

2457714.81083 8.734 0.007

2457714.81302 8.768 0.007

2457714.81522 8.762 0.007

2457714.81741 8.777 0.007

2457714.81961 8.770 0.007

2457714.82178 8.766 0.007

2457714.82396 8.765 0.007

2457714.82613 8.766 0.007

2457714.82831 8.770 0.007

2457714.83051 8.773 0.007

2457714.83281 8.779 0.007

2457714.83494 8.787 0.007

2457714.83704 8.808 0.007

2457714.83912 8.800 0.007

2457714.84120 8.810 0.007

2457714.84331 8.818 0.006

2457714.84539 8.814 0.007

2457715.67558 8.786 0.009

2457715.67788 8.775 0.009

2457715.67966 8.800 0.009

2457715.68144 8.795 0.015

2457715.68322 8.758 0.008

2457715.68499 8.747 0.008

2457715.68677 8.780 0.008

2457715.68856 8.790 0.008

2457715.69037 8.786 0.008

2457715.69258 8.774 0.008

2457715.69478 8.791 0.008

2457715.69698 8.807 0.008

2457715.69916 8.766 0.008

2457715.70137 8.776 0.008

2457715.70359 8.793 0.008

2457715.70579 8.788 0.007

2457715.70799 8.778 0.008

2457715.71021 8.784 0.007

2457715.71242 8.780 0.007

2457715.71461 8.772 0.007

2457715.71679 8.784 0.007

2457715.71902 8.766 0.007

2457715.72124 8.788 0.007

2457715.72341 8.782 0.007

2457715.72563 8.752 0.007

2457715.72782 8.754 0.007

2457715.73001 8.748 0.007

2457715.73223 8.765 0.007

2457715.73444 8.759 0.007

2457715.73666 8.760 0.007

2457715.73888 8.758 0.007

2457715.74106 8.775 0.007

2457715.74328 8.783 0.007

2457715.74547 8.792 0.007

2457715.74767 8.769 0.007

2457715.74988 8.767 0.007

2457715.75208 8.798 0.007

2457715.75428 8.779 0.007

2457715.75648 8.782 0.007

2457715.75868 8.773 0.007

2457715.76088 8.774 0.007

2457715.76307 8.762 0.007

2457715.76525 8.771 0.007

2457715.76747 8.769 0.007

2457715.76968 8.790 0.007

2457715.77188 8.787 0.007

2457715.77407 8.771 0.007

2457715.77627 8.784 0.007

2457715.77847 8.779 0.007

2457715.78067 8.779 0.007

2457715.78287 8.775 0.007

2457715.78507 8.772 0.007

2457715.78727 8.767 0.007

2457715.78947 8.780 0.007

2457715.79167 8.763 0.007

2457715.79387 8.767 0.007

2457715.79606 8.768 0.007

2457715.79826 8.774 0.007

2457715.80046 8.785 0.006

2457715.80266 8.771 0.007

2457715.80486 8.778 0.007

2457715.80706 8.767 0.007

2457715.80926 8.769 0.007

2457715.81147 8.770 0.007

2457715.81367 8.765 0.007

2457715.81587 8.773 0.007

2457715.81807 8.772 0.007

2457715.82027 8.769 0.007

2457715.82245 8.773 0.007

2457715.82465 8.767 0.007

2457715.82686 8.787 0.007

2457715.82906 8.764 0.007

2457715.83126 8.775 0.007

2457715.83341 8.776 0.007

2457715.83569 8.782 0.006

2457715.83799 8.790 0.007

2457715.84027 8.797 0.007

2457715.84253 8.785 0.007

2457716.67433 8.779 0.009

2457716.67609 8.787 0.009

2457716.67786 8.764 0.009

2457716.67963 8.768 0.009

2457716.68140 8.767 0.009

2457716.68318 8.776 0.008

2457716.68498 8.776 0.008

2457716.68675 8.778 0.008

2457716.68912 8.757 0.008

2457716.69149 8.766 0.008

2457716.69387 8.809 0.008

2457716.69627 8.770 0.008

2457716.69866 8.789 0.008

2457716.70104 8.775 0.008

2457716.70343 8.792 0.008

2457716.70581 8.800 0.007

2457716.70821 8.773 0.007

2457716.71060 8.785 0.007

2457716.71299 8.795 0.007

2457716.71537 8.784 0.007

2457716.71773 8.780 0.007

2457716.72012 8.782 0.007

2457716.72249 8.782 0.007

2457716.72486 8.794 0.007

2457716.72726 8.779 0.007

2457716.72964 8.803 0.007

2457716.73203 8.787 0.007

2457716.73441 8.785 0.007

2457716.73679 8.781 0.007

2457716.73916 8.802 0.007

2457716.74154 8.772 0.007

2457716.74394 8.787 0.007

2457716.74631 8.795 0.007

2457716.74868 8.776 0.007

2457716.75108 8.799 0.007

2457716.75344 8.779 0.007

2457716.75582 8.786 0.007

2457716.75821 8.788 0.007

2457716.76058 8.775 0.007

2457716.76297 8.801 0.007

2457716.76537 8.785 0.007

2457716.76775 8.775 0.007

2457716.77015 8.774 0.007

2457716.77255 8.787 0.007

2457716.77493 8.788 0.007

2457716.77731 8.796 0.007

2457716.77969 8.777 0.007

2457716.78208 8.778 0.007

2457716.78444 8.773 0.007

2457716.78683 8.769 0.007

2457716.78921 8.788 0.007

2457716.79160 8.770 0.007

2457716.79397 8.789 0.007

2457716.79635 8.785 0.007

2457716.79875 8.772 0.007

2457716.80112 8.782 0.007

2457716.80351 8.782 0.007

2457716.80589 8.771 0.007

2457716.80825 8.797 0.006

2457716.81066 8.774 0.007

2457716.81302 8.775 0.007

2457716.81539 8.775 0.007

2457716.81777 8.786 0.006

2457716.82014 8.766 0.007

2457716.82251 8.785 0.007

2457716.82488 8.787 0.006

2457716.82726 8.780 0.006

2457716.82958 8.776 0.006

2457716.83449 8.771 0.007

2457716.83693 8.775 0.007

2457716.83940 8.783 0.006

2457717.67152 8.735 0.009

2457717.67307 8.748 0.009

2457717.67461 8.761 0.009

2457717.67617 8.749 0.009

2457717.67773 8.753 0.009

2457717.67929 8.728 0.008

2457717.68084 8.760 0.008

2457717.68241 8.733 0.008

2457717.68397 8.746 0.008

2457717.68552 8.746 0.008

2457717.68708 8.768 0.008

2457717.68866 8.757 0.008

2457717.69022 8.774 0.008

2457717.69177 8.768 0.008

2457717.69333 8.742 0.008

2457717.69490 8.748 0.008

2457717.69644 8.741 0.008

2457717.69799 8.750 0.008

2457717.69954 8.778 0.008

2457717.70111 8.754 0.008

2457717.70265 8.768 0.008

2457717.70421 8.747 0.008

2457717.70578 8.779 0.007

2457717.70734 8.763 0.007

2457717.70891 8.769 0.007

2457717.71046 8.769 0.007

2457717.71203 8.755 0.007

2457717.71359 8.780 0.007

2457717.71514 8.780 0.007

2457717.71670 8.763 0.007

2457717.71825 8.775 0.007

2457717.71981 8.764 0.007

2457717.72138 8.764 0.007

2457717.72293 8.763 0.007

2457717.72448 8.777 0.007

2457717.72605 8.753 0.007

2457717.72762 8.774 0.007

2457717.72918 8.750 0.007

2457717.73073 8.764 0.007

2457717.73228 8.772 0.007

2457717.73384 8.776 0.007

2457717.73539 8.755 0.007

2457717.73694 8.774 0.007

2457717.73850 8.755 0.007

2457717.74003 8.760 0.007

2457717.74157 8.747 0.007

2457717.74311 8.757 0.007

2457717.74466 8.755 0.007

2457717.74620 8.764 0.007

2457717.74774 8.767 0.007

2457717.74927 8.773 0.007

2457717.75082 8.759 0.007

2457717.75237 8.750 0.007

2457717.75392 8.772 0.007

2457717.75547 8.761 0.007

2457717.75704 8.756 0.007

2457717.75858 8.777 0.007

2457717.76013 8.758 0.007

2457717.76168 8.760 0.007

2457717.76323 8.764 0.007

2457717.76478 8.758 0.007

2457717.76633 8.751 0.007

2457717.76788 8.760 0.007

2457717.76942 8.747 0.007

2457717.77096 8.749 0.007

2457717.77251 8.757 0.007

2457717.77406 8.784 0.007

2457717.77561 8.771 0.007

2457717.77716 8.753 0.007

2457717.77872 8.753 0.007

2457717.78025 8.753 0.007

2457717.78179 8.750 0.007

2457717.78334 8.751 0.007

2457717.78491 8.744 0.007

2457717.78646 8.756 0.007

2457717.78801 8.768 0.007

2457717.78956 8.760 0.007

2457717.79111 8.759 0.007

2457717.79266 8.753 0.007

2457717.79421 8.753 0.007

2457717.79575 8.741 0.007

2457717.79730 8.763 0.006

2457717.79885 8.753 0.007

2457717.80041 8.743 0.007

2457717.80194 8.758 0.007

2457717.80347 8.746 0.007

2457717.80502 8.740 0.007

2457717.80657 8.750 0.007

2457717.80813 8.744 0.007

2457717.80968 8.765 0.007

2457717.81123 8.747 0.007

2457717.81278 8.749 0.007

2457717.81433 8.741 0.007

2457717.81588 8.752 0.007

2457717.81743 8.760 0.007

2457717.81899 8.749 0.007

2457717.82054 8.758 0.007

2457717.82208 8.760 0.007

2457717.82363 8.763 0.007

2457717.82519 8.761 0.007

2457717.82669 8.760 0.007

2457717.82816 8.740 0.007

2457717.82963 8.760 0.007

2457717.83110 8.759 0.007

2457717.83256 8.765 0.007

2457717.83403 8.747 0.007

2457717.83694 8.748 0.007

2457717.83840 8.748 0.007

2457717.83985 8.764 0.007

2457717.84279 8.764 0.007

2457718.67025 8.751 0.009

2457718.67328 8.732 0.009

2457718.67631 8.766 0.009

2457718.67934 8.754 0.010

2457718.68235 8.775 0.009

2457718.68538 8.761 0.008

2457718.68841 8.747 0.008

2457718.69144 8.754 0.008

2457718.69447 8.762 0.008

2457718.69750 8.756 0.008

2457718.70052 8.770 0.008

2457718.70356 8.759 0.008

2457718.70657 8.759 0.008

2457718.70961 8.754 0.007

2457718.71263 8.746 0.008

2457718.71565 8.745 0.008

2457718.71867 8.755 0.008

2457718.72172 8.747 0.008

2457718.72476 8.758 0.008

2457718.72779 8.756 0.007

2457718.73083 8.763 0.007

2457718.73387 8.756 0.008

2457718.73690 8.746 0.007

2457718.73994 8.743 0.008

2457718.74297 8.757 0.008

2457718.74600 8.743 0.008

2457718.74905 8.743 0.008

2457718.75207 8.741 0.008

2457718.75510 8.757 0.007

2457718.75816 8.751 0.008

2457718.76118 8.761 0.008

2457718.76422 8.728 0.008

2457718.76728 8.758 0.008

2457718.77032 8.745 0.007

2457718.77337 8.755 0.007

2457718.77641 8.750 0.008

2457718.77946 8.755 0.008

2457718.78251 8.751 0.007

2457718.78557 8.747 0.008

2457718.78856 8.742 0.009

2457718.79159 8.740 0.008

2457718.79463 8.754 0.008

2457718.79767 8.750 0.007

2457718.80071 8.749 0.009

2457718.80374 8.758 0.007

2457718.80676 8.732 0.008

2457718.80978 8.750 0.009

2457718.81281 8.731 0.008

2457718.81582 8.747 0.007

2457718.81884 8.752 0.007

2457718.82186 8.750 0.007

2457718.82775 8.755 0.007

2457718.83069 8.735 0.009

2457718.83365 8.752 0.010

2457718.83661 8.740 0.011

2457718.83954 8.749 0.010

2457718.84247 8.776 0.010

2457719.66683 9.026 0.009

2457719.66922 9.032 0.009

2457719.67166 9.042 0.009

2457719.67405 9.028 0.009

2457719.67645 9.036 0.009

2457719.67884 9.071 0.008

2457719.68126 9.036 0.008

2457719.68366 9.064 0.008

2457719.68604 9.038 0.008

2457719.68844 9.071 0.008

2457719.69083 9.044 0.008

2457719.69323 9.053 0.008

2457719.69565 9.068 0.008

2457719.69804 9.054 0.008

2457719.70044 9.060 0.008

2457719.70285 9.059 0.008

2457719.70524 9.041 0.008

2457719.70763 9.075 0.008

2457719.71003 9.046 0.008

2457719.71243 9.059 0.007

2457719.71481 9.069 0.007

2457719.71722 9.067 0.007

2457719.71963 9.041 0.007

2457719.72204 9.036 0.007

2457719.72441 9.061 0.007

2457719.72682 9.050 0.007

2457719.72921 9.039 0.007

2457719.73163 9.042 0.007

2457719.73405 9.021 0.007

2457719.73646 9.020 0.007

2457719.73888 9.030 0.007

2457719.74131 9.001 0.007

2457719.74372 9.020 0.007

2457719.74612 9.008 0.007

2457719.74853 9.024 0.007

2457719.75093 8.992 0.007

2457719.75332 8.983 0.007

2457719.75572 8.987 0.007

2457719.75813 8.976 0.007

2457719.76052 8.964 0.007

2457719.76292 8.967 0.007

2457719.76534 8.957 0.007

2457719.76775 8.945 0.007

2457719.77016 8.933 0.007

2457719.77256 8.927 0.007

2457719.77494 8.927 0.007

2457719.77735 8.925 0.007

2457719.77977 8.911 0.007

2457719.78218 8.906 0.007

2457719.78457 8.913 0.007

2457719.78699 8.899 0.007

2457719.78939 8.873 0.007

2457719.79178 8.875 0.007

2457719.79418 8.866 0.007

2457719.79657 8.864 0.007

2457719.79897 8.858 0.007

2457719.80137 8.852 0.007

2457719.80376 8.867 0.007

2457719.80616 8.838 0.007

2457719.80854 8.828 0.007

2457719.81094 8.823 0.007

2457719.81333 8.808 0.007

2457719.81574 8.819 0.007

2457719.81814 8.803 0.007

2457719.82046 8.808 0.007

2457719.82274 8.810 0.007

2457719.82500 8.813 0.007

2457719.82727 8.794 0.007

2457719.82954 8.800 0.007

2457719.83410 8.776 0.007

2457719.83637 8.770 0.007

2457719.83863 8.787 0.007

2457720.66544 8.786 0.009

2457720.66848 8.794 0.009

2457720.67157 8.770 0.009

2457720.67465 8.780 0.009

2457720.67772 8.769 0.009

2457720.68079 8.785 0.008

2457720.68384 8.782 0.008

2457720.68690 8.763 0.008

2457720.68995 8.789 0.008

2457720.69304 8.797 0.008

2457720.69610 8.799 0.008

2457720.69913 8.781 0.008

2457720.70219 8.777 0.008

2457720.70525 8.785 0.008

2457720.70830 8.778 0.008

2457720.71134 8.775 0.008

2457720.71442 8.790 0.008

2457720.71745 8.787 0.008

2457720.72050 8.771 0.008

2457720.72355 8.785 0.008

2457720.72662 8.793 0.007

2457720.72965 8.802 0.007

2457720.73271 8.791 0.008

2457720.73576 8.779 0.008

2457720.73883 8.795 0.008

2457720.74190 8.776 0.007

2457720.74493 8.786 0.007

2457720.74799 8.773 0.008

2457720.75106 8.785 0.008

2457720.75414 8.778 0.008

2457720.75721 8.765 0.007

2457720.76028 8.790 0.007

2457720.76336 8.769 0.007

2457720.76641 8.779 0.007

2457720.76947 8.772 0.007

2457720.77252 8.782 0.007

2457720.77558 8.803 0.007

2457720.77862 8.780 0.007

2457720.78167 8.772 0.007

2457720.78478 8.772 0.007

2457720.78789 8.769 0.007

2457720.79102 8.766 0.007

2457720.79414 8.768 0.007

2457720.79727 8.766 0.007

2457720.80038 8.777 0.007

2457720.80352 8.787 0.007

2457720.80662 8.783 0.007

2457720.80972 8.770 0.007

2457720.81282 8.775 0.007

2457720.81593 8.786 0.007

2457720.81889 8.779 0.007

2457720.82183 8.779 0.007

2457720.82476 8.783 0.007

2457720.82767 8.778 0.007

2457720.83061 8.797 0.007

2457720.83646 8.772 0.007

2457720.83938 8.780 0.007

2457720.84230 8.785 0.007

2457721.66262 8.744 0.011

2457721.66566 8.799 0.009

2457721.66872 8.772 0.009

2457721.67179 8.783 0.009

2457721.67486 8.796 0.008

2457721.67792 8.784 0.009

2457721.68097 8.775 0.008

2457721.68403 8.792 0.008

2457721.68709 8.791 0.008

2457721.69014 8.777 0.008

2457721.69319 8.783 0.008

2457721.69625 8.772 0.008

2457721.69928 8.801 0.008

2457721.70234 8.778 0.008

2457721.70539 8.787 0.007

2457721.70844 8.797 0.008

2457721.71149 8.789 0.008

2457721.71455 8.783 0.007

2457721.71762 8.791 0.007

2457721.72067 8.768 0.007

2457721.72374 8.793 0.007

2457721.72682 8.775 0.007

2457721.72987 8.773 0.007

2457721.73295 8.780 0.007

2457721.73601 8.778 0.007

2457721.73905 8.772 0.008

2457721.74213 8.780 0.008

2457721.74519 8.778 0.007

2457721.74825 8.771 0.007

2457721.75132 8.782 0.007

2457721.75435 8.782 0.007

2457721.75742 8.772 0.007

2457721.76049 8.791 0.007

2457721.76353 8.787 0.007

2457721.76656 8.798 0.007

2457721.76962 8.763 0.007

2457721.77266 8.770 0.007

2457721.77571 8.775 0.007

2457721.77874 8.768 0.007

2457721.78185 8.764 0.007

2457721.78495 8.773 0.007

2457721.78804 8.756 0.007

2457721.79116 8.774 0.007

2457721.79426 8.770 0.007

2457721.79735 8.768 0.007

2457721.80045 8.782 0.007

2457721.80355 8.776 0.007

2457721.80666 8.788 0.007

2457721.80976 8.773 0.007

2457721.81287 8.780 0.007

2457721.81584 8.791 0.007

2457721.81877 8.768 0.007

2457721.82171 8.790 0.007

2457721.83051 8.772 0.007

2457721.83634 8.798 0.007

2457721.83927 8.781 0.007

2457721.84219 8.766 0.007

2457722.66292 8.791 0.021

2457722.66596 8.786 0.015

2457722.66900 8.795 0.016

2457722.67205 8.746 0.014

2457722.67512 8.757 0.021

2457722.68124 8.768 0.016

2457722.68432 8.737 0.012

2457722.68738 8.759 0.011

2457722.69403 8.737 0.019

2457722.69706 8.739 0.015

2457722.70013 8.767 0.014

2457722.70318 8.744 0.011

2457722.70625 8.753 0.010

2457722.71236 8.767 0.012

2457722.71542 8.768 0.012

2457722.71846 8.772 0.011

2457722.72152 8.781 0.015

2457722.73071 8.766 0.016

2457722.73377 8.751 0.018

2457722.73685 8.756 0.014

2457722.73991 8.793 0.019

2457722.74297 8.748 0.025

2457722.74600 8.780 0.029

2457724.65351 8.769 0.010

2457724.65571 8.775 0.014

2457724.65792 8.789 0.011

2457724.66016 8.759 0.010

2457724.66238 8.768 0.010

2457724.66684 8.742 0.011

2457724.66906 8.777 0.015

2457724.67131 8.758 0.026

2457724.67351 8.737 0.015

2457724.67573 8.787 0.023

2457724.67793 8.761 0.019

2457724.68016 8.773 0.018

2457724.68240 8.808 0.032

2457724.68685 8.757 0.028

2457724.68907 8.782 0.015

2457724.69130 8.781 0.014

2457724.69353 8.752 0.012

2457724.69575 8.774 0.011

2457724.69800 8.765 0.012

2457724.70021 8.799 0.020

2457724.70243 8.751 0.013

2457724.70464 8.796 0.024

2457724.70685 8.765 0.030

2457724.70907 8.763 0.013

2457724.71128 8.769 0.015

2457724.71351 8.774 0.026

2457724.71795 8.754 0.036

2457724.72019 8.812 0.030

2457724.72241 8.749 0.030

2457724.72686 8.749 0.021

2457724.72909 8.774 0.012

2457724.73130 8.774 0.011

2457724.73573 8.761 0.012

2457724.73796 8.785 0.012

2457724.74019 8.788 0.018

2457724.74242 8.786 0.012

2457724.74464 8.754 0.009

2457724.74686 8.768 0.009

2457724.74910 8.778 0.013

2457724.75131 8.782 0.014

2457724.75353 8.791 0.013

2457724.75575 8.760 0.007

2457724.75797 8.760 0.007

2457724.76019 8.771 0.007

2457724.76241 8.776 0.007

2457724.76464 8.752 0.007

2457724.76686 8.759 0.007

2457724.76909 8.774 0.007

2457724.77131 8.757 0.008

2457724.77360 8.763 0.007

2457724.77589 8.768 0.007

2457724.77817 8.767 0.007

2457724.78046 8.766 0.007

2457724.78277 8.773 0.007

2457724.78506 8.757 0.007

2457724.78736 8.768 0.007

2457724.78965 8.770 0.007

2457724.79194 8.771 0.007

2457724.79425 8.773 0.007

2457724.79653 8.764 0.007

2457724.79882 8.753 0.007

2457724.80111 8.782 0.007

2457724.80340 8.765 0.007

2457724.80569 8.771 0.007

2457724.80786 8.760 0.007

2457724.80998 8.771 0.007

2457724.81207 8.774 0.007

2457724.81418 8.800 0.007

2457724.81630 8.784 0.007

2457724.81840 8.776 0.007

2457724.82051 8.784 0.009

2457724.83535 8.759 0.007

2457724.83747 8.775 0.007

2457724.83958 8.774 0.007

2457724.84171 8.767 0.007

2457725.65106 8.793 0.009

2457725.65375 8.785 0.009

2457725.65646 8.804 0.009

2457725.65917 8.768 0.008

2457725.66186 8.785 0.009

2457725.66456 8.795 0.008

2457725.66726 8.774 0.008

2457725.66998 8.811 0.008

2457725.67266 8.780 0.008

2457725.67536 8.795 0.008

2457725.67807 8.776 0.008

2457725.68078 8.795 0.008

2457725.68347 8.783 0.008

2457725.68618 8.775 0.008

2457725.68889 8.783 0.008

2457725.69157 8.797 0.007

2457725.69427 8.790 0.007

2457725.69697 8.790 0.007

2457725.69966 8.754 0.008

2457725.70237 8.789 0.007

2457725.70508 8.779 0.007

2457725.70778 8.781 0.007

2457725.71049 8.772 0.007

2457725.71318 8.802 0.007

2457725.71590 8.793 0.007

2457725.71860 8.796 0.007

2457725.72131 8.801 0.007

2457725.72403 8.800 0.007

2457725.72675 8.796 0.007

2457725.72942 8.780 0.007

2457725.73211 8.793 0.007

2457725.73479 8.787 0.007

2457725.73749 8.788 0.007

2457725.74019 8.782 0.007

2457725.74288 8.794 0.007

2457725.74557 8.786 0.007

2457725.74830 8.780 0.007

2457725.75100 8.785 0.007

2457725.75380 8.796 0.007

2457725.75660 8.791 0.007

2457725.75940 8.774 0.007

2457725.76220 8.792 0.007

2457725.76501 8.797 0.007

2457725.76782 8.783 0.007

2457725.77061 8.778 0.007

2457725.77337 8.785 0.007

2457725.77615 8.780 0.007

2457725.77891 8.784 0.007

2457725.78170 8.780 0.007

2457725.78447 8.790 0.007

2457725.78726 8.771 0.007

2457725.79006 8.786 0.007

2457725.79282 8.777 0.007

2457725.79559 8.782 0.007

2457725.79836 8.777 0.007

2457725.80113 8.784 0.007

2457725.80392 8.782 0.007

2457725.80657 8.777 0.007

2457725.81174 8.796 0.007

2457725.81432 8.782 0.007

2457725.81692 8.788 0.007

2457725.82462 8.792 0.007

2457725.83238 8.783 0.007

2457725.83755 8.790 0.007

2457726.64847 8.749 0.014

2457726.65955 8.766 0.013

2457726.66327 8.841 0.013

2457726.66695 8.803 0.013

2457726.67064 8.746 0.013

2457726.67803 8.767 0.012

2457726.68172 8.766 0.012

2457726.68911 8.776 0.012

2457726.69282 8.796 0.012

2457726.69652 8.781 0.012

2457726.70025 8.795 0.012

2457726.70394 8.779 0.012

2457726.70761 8.798 0.012

2457726.71131 8.816 0.012

2457726.71497 8.797 0.013

2457726.71867 8.802 0.013

2457726.72237 8.809 0.012

2457726.72607 8.810 0.012

2457726.72979 8.828 0.011

2457726.73348 8.827 0.011

2457726.73718 8.814 0.011

2457726.74089 8.825 0.012

2457726.74458 8.823 0.011

2457726.74828 8.835 0.011

2457726.75203 8.830 0.011

2457726.75575 8.858 0.011

2457726.75946 8.849 0.012

2457726.76318 8.865 0.012

2457726.76689 8.854 0.011

2457726.77058 8.862 0.011

2457726.77428 8.853 0.012

2457726.77798 8.867 0.011

2457726.78168 8.857 0.011

2457726.78539 8.853 0.011

2457726.78909 8.858 0.011

2457726.79280 8.874 0.011

2457726.79651 8.884 0.011

2457726.80023 8.897 0.011

2457726.80383 8.859 0.011

2457726.80760 8.885 0.011

2457726.81136 8.900 0.011

2457726.81512 8.894 0.011

2457726.81888 8.885 0.011

2457726.82263 8.881 0.011

2457726.82642 8.894 0.011

2457726.82998 8.876 0.011

2457726.83356 8.888 0.011

2457727.64423 8.761 0.014

2457727.64645 8.751 0.014

2457727.64866 8.778 0.014

2457727.65087 8.739 0.014

2457727.65310 8.745 0.014

2457727.65530 8.754 0.013

2457727.65749 8.776 0.016

2457727.65972 8.763 0.013

2457727.66193 8.743 0.015

2457727.66414 8.727 0.014

2457727.66634 8.740 0.013

2457727.66855 8.767 0.013

2457727.67078 8.746 0.013

2457727.67300 8.756 0.013

2457727.67524 8.748 0.013

2457727.67742 8.748 0.013

2457727.67966 8.736 0.013

2457727.68189 8.721 0.013

2457727.68411 8.737 0.012

2457727.68634 8.752 0.012

2457727.68856 8.751 0.013

2457727.69077 8.755 0.012

2457727.69298 8.738 0.013

2457727.69519 8.750 0.013

2457727.69740 8.744 0.012

2457727.69961 8.765 0.012

2457727.70181 8.741 0.012

2457727.70401 8.751 0.012

2457727.70621 8.768 0.012

2457727.70842 8.734 0.012

2457727.71064 8.753 0.012

2457727.71289 8.748 0.012

2457727.71511 8.739 0.012

2457727.71733 8.755 0.012

2457727.71955 8.738 0.012

2457727.72177 8.757 0.012

2457727.72398 8.772 0.011

2457727.72616 8.746 0.012

2457727.72835 8.762 0.011

2457727.73056 8.726 0.012

2457727.73280 8.749 0.012

2457727.73501 8.745 0.012

2457727.73724 8.767 0.011

2457727.73943 8.752 0.011

2457727.74164 8.737 0.011

2457727.74385 8.768 0.011

2457727.74608 8.763 0.012

2457727.74828 8.742 0.011

2457727.75047 8.759 0.011

2457727.75268 8.744 0.011

2457727.75487 8.735 0.011

2457727.75708 8.752 0.011

2457727.75957 8.759 0.011

2457727.76207 8.768 0.011

2457727.76454 8.737 0.012

2457727.76704 8.726 0.011

2457727.76953 8.718 0.012

2457727.77201 8.752 0.011

2457727.77448 8.735 0.011

2457727.77690 8.739 0.012

2457727.77933 8.750 0.011

2457727.78175 8.721 0.014

2457727.78903 8.757 0.012

2457727.79144 8.724 0.012

2457727.79386 8.724 0.011

2457727.79630 8.748 0.011

2457727.79861 8.767 0.011

2457727.80112 8.739 0.011

2457727.80359 8.755 0.011

2457727.80609 8.739 0.011

2457727.80857 8.761 0.011

2457727.81107 8.742 0.011

2457727.81357 8.744 0.011

2457727.81607 8.766 0.011

2457727.81855 8.743 0.011

2457727.82104 8.760 0.011

2457727.82325 8.761 0.011

2457727.82547 8.742 0.011

2457727.82768 8.743 0.011

2457727.82989 8.748 0.011

2457727.83209 8.716 0.011

2457727.83429 8.751 0.011

2457728.64150 8.782 0.015

2457728.64373 8.771 0.014

2457728.64593 8.722 0.015

2457728.64817 8.748 0.015

2457728.65036 8.754 0.014

2457728.65256 8.773 0.014

2457728.65479 8.768 0.014

2457728.65700 8.774 0.013

2457728.65921 8.758 0.013

2457728.66143 8.766 0.013

2457728.66366 8.724 0.013

2457728.66586 8.759 0.013

2457728.66806 8.734 0.013

2457728.67028 8.775 0.013

2457728.67249 8.765 0.013

2457728.67470 8.719 0.013

2457728.67690 8.749 0.012

2457728.67913 8.762 0.012

2457728.68133 8.770 0.012

2457728.68356 8.733 0.012

2457728.68576 8.741 0.012

2457728.68797 8.753 0.012

2457728.69018 8.755 0.012

2457728.69240 8.771 0.012

2457728.69460 8.738 0.012

2457728.69681 8.742 0.012

2457728.69901 8.749 0.012

2457728.70123 8.761 0.012

2457728.70348 8.789 0.012

2457728.70569 8.745 0.012

2457728.70792 8.745 0.012

2457728.71013 8.727 0.012

2457728.71234 8.742 0.012

2457728.71455 8.750 0.012

2457728.71675 8.757 0.012

2457728.71895 8.760 0.012

2457728.72117 8.741 0.012

2457728.72340 8.781 0.011

2457728.72561 8.743 0.012

2457728.72782 8.757 0.012

2457728.73004 8.753 0.011

2457728.73225 8.743 0.012

2457728.73448 8.769 0.012

2457728.73670 8.739 0.011

2457728.73892 8.755 0.012

2457728.74114 8.745 0.011

2457728.74334 8.752 0.011

2457728.74555 8.749 0.011

2457728.74777 8.746 0.011

2457728.74997 8.736 0.011

2457728.75218 8.740 0.011

2457728.75438 8.747 0.011

2457728.75687 8.748 0.011

2457728.75935 8.764 0.011

2457728.76183 8.755 0.011

2457728.76433 8.741 0.011

2457728.76682 8.754 0.011

2457728.76931 8.741 0.011

2457728.77180 8.734 0.011

2457728.77423 8.747 0.011

2457728.77665 8.727 0.012

2457728.77907 8.717 0.011

2457728.78149 8.721 0.011

2457728.78392 8.740 0.011

2457728.78636 8.757 0.011

2457728.78878 8.724 0.012

2457728.79120 8.753 0.011

2457728.79364 8.738 0.011

2457728.79598 8.756 0.011

2457728.79844 8.747 0.011

2457728.80094 8.734 0.011

2457728.80342 8.733 0.011

2457728.80590 8.753 0.011

2457728.80839 8.759 0.011

2457728.81089 8.740 0.011

2457728.81336 8.744 0.011

2457728.81583 8.758 0.011

2457728.81830 8.757 0.011

2457728.82050 8.754 0.011

2457728.82270 8.737 0.011

2457728.82490 8.745 0.011

2457728.82712 8.740 0.011

2457728.82933 8.751 0.011

2457728.83155 8.762 0.011

2457728.83376 8.760 0.011

2457729.63998 8.791 0.015

2457729.64218 8.752 0.014

2457729.64438 8.776 0.014

2457729.64659 8.740 0.014

2457729.64881 8.747 0.014

2457729.65102 8.788 0.013

2457729.65323 8.792 0.013

2457729.65545 8.782 0.014

2457729.65763 8.814 0.014

2457729.65982 8.797 0.013

2457729.66204 8.809 0.013

2457729.66425 8.790 0.013

2457729.66646 8.794 0.013

2457729.66864 8.783 0.013

2457729.67086 8.785 0.013

2457729.67306 8.783 0.013

2457729.67527 8.784 0.012

2457729.67748 8.767 0.013

2457729.67968 8.778 0.013

2457729.68189 8.810 0.012

2457729.68410 8.773 0.012

2457729.68630 8.790 0.012

2457729.68852 8.807 0.012

2457729.69075 8.775 0.012

2457729.69296 8.770 0.012

2457729.69517 8.767 0.012

2457729.69739 8.762 0.012

2457729.69960 8.785 0.012

2457729.70183 8.775 0.012

2457729.70406 8.792 0.012

2457729.70628 8.764 0.012

2457729.70851 8.769 0.012

2457729.71071 8.796 0.011

2457729.71293 8.760 0.012

2457729.71518 8.790 0.012

2457729.71739 8.770 0.012

2457729.71959 8.755 0.011

2457729.72178 8.777 0.011

2457729.72400 8.739 0.011

2457729.72622 8.776 0.011

2457729.72844 8.756 0.011

2457729.73065 8.755 0.011

2457729.73289 8.780 0.011

2457729.73510 8.780 0.011

2457729.73732 8.782 0.011

2457729.73955 8.782 0.011

2457729.74178 8.779 0.011

2457729.74399 8.779 0.011

2457729.74620 8.754 0.011

2457729.74840 8.795 0.011

2457729.75062 8.773 0.011

2457729.75310 8.785 0.011

2457729.75556 8.760 0.011

2457729.75805 8.765 0.011

2457729.76053 8.772 0.011

2457729.76300 8.783 0.011

2457729.76549 8.753 0.011

2457729.76798 8.757 0.011

2457729.77048 8.760 0.011

2457729.77289 8.751 0.011

2457729.77531 8.787 0.011

2457729.77773 8.774 0.011

2457729.78014 8.767 0.011

2457729.78256 8.780 0.011

2457729.78498 8.768 0.011

2457729.78741 8.758 0.011

2457729.78983 8.773 0.011

2457729.79224 8.768 0.011

2457729.79454 8.784 0.011

2457729.79702 8.772 0.011

2457729.79953 8.768 0.011

2457729.80201 8.780 0.011

2457729.80452 8.796 0.011

2457729.80700 8.770 0.012

2457729.80947 8.778 0.011

2457729.81196 8.758 0.011

2457729.81442 8.749 0.011

2457729.81687 8.791 0.011

2457729.81909 8.765 0.011

2457729.82130 8.793 0.011

2457729.82350 8.795 0.011

2457729.82571 8.773 0.011

2457729.82791 8.766 0.011

2457729.83011 8.795 0.011

2457729.83231 8.787 0.011

2457729.83451 8.742 0.011
