# Supplementary material for: HD 66051, an eclipsing binary hosting a highly peculiar, HgMn-related star
Source: Sci Rep. 2017 Jul 19;7:5906. doi: 10.1038/s41598-017-05987-6 (PMC5517476; doi:10.1038/s41598-017-05987-6)
Supplement: Supplementary file 4 — Supplementary Dataset 4 [file 41598_2017_5987_MOESM4_ESM.doc]

HD66051, an eclipsing binary hosting a highly peculiar, HgMn-related star

Ewa Niemczura

Stefan Hümmerich

Fiorella Castelli

Ernst Paunzen

Klaus Bernhard

Franz-Josef Hambsch

Krzysztof Hełminiak

HIDES spectrum of HD66051

Wavelength Normalised Flux

4084.48039815734 0.1933778

4084.49846427895 0.5198962

4084.51653040055 0.8355485

4084.53459652215 0.5751579

4084.55266264375 0.476863

4084.57072876536 0.365466

4084.58879488696 0.4263904

4084.60686100856 0.6230224

4084.62492713016 0.6761874

4084.64299325176 0.3795129

4084.66105937337 0.2250205

4084.67912549497 0.7266065

4084.69719161657 1.160024

4084.71525773817 1.694861

4084.73332385978 1.230216

4084.75138998138 1.046466

4084.76945610298 0.7769199

4084.78752222458 0.7461693

4084.80558834618 1.184342

4084.82365446779 1.030391

4084.84172058939 0.4935443

4084.85978671099 0.4192889

4084.87785283259 0.6335107

4084.8959189542 0.8058455

4084.9139850758 0.9689497

4084.9320511974 0.6807116

4084.950117319 0.6281183

4084.9681834406 0.8864292

4084.98624956221 0.8793902

4085.00431568381 0.8044877

4085.02238180541 0.3512679

4085.04044792701 0.2959768

4085.05851404862 0.4480782

4085.07658017022 0.1249558

4085.09464629182 0.47534

4085.11271241342 0.6985117

4085.13077853502 0.9167761

4085.14884465663 0.5722238

4085.16691077823 0.5379972

4085.18497689983 0.3595805

4085.20304302143 0.4833073

4085.22110914304 0.9444122

4085.23917526464 0.6430568

4085.25724138624 0.6678523

4085.27530750784 0.560197

4085.29337362944 0.9370478

4085.31143975105 1.170612

4085.32950587265 1.013833

4085.34757199425 0.8799188

4085.36563811585 0.6786076

4085.38370423746 0.6113707

4085.40177035906 0.9702381

4085.41983648066 1.179171

4085.43790260226 0.9537039

4085.45596872386 0.6008267

4085.47403484547 0.6583038

4085.49210096707 1.164939

4085.51016708867 1.232283

4085.52823321027 1.116101

4085.54629933188 0.8945158

4085.56436545348 0.4113796

4085.58243157508 0.4336812

4085.60049769668 0.8506765

4085.61856381828 0.9367172

4085.63662993989 0.9321597

4085.65469606149 0.7966337

4085.67276218309 0.9104538

4085.69082830469 0.7342426

4085.70889442629 0.3676559

4085.7269605479 0.08806394

4085.7450266695 0.5031621

4085.7630927911 1.0144

4085.7811589127 0.7353031

4085.79922503431 0.378257

4085.81729115591 0.1888106

4085.83535727751 0.6293546

4085.85342339911 0.6567049

4085.87148952071 0.1212854

4085.88955564232 0.6361424

4085.90762176392 0.9455805

4085.92568788552 1.058017

4085.94375400712 1.042883

4085.96182012873 0.6355318

4085.97988625033 0.4483632

4085.99795237193 0.5033713

4086.01601849353 0.5191825

4086.03408461513 0.6402437

4086.05215073673 0.5383739

4086.07021685834 0.4446979

4086.08828297994 0.6200945

4086.10634910154 0.9041377

4086.12441522314 1.159825

4086.14248134475 0.8087378

4086.16054746635 0.620459

4086.17861358795 0.6741616

4086.19667970955 0.7615814

4086.21474583116 0.677681

4086.23281195276 0.2965983

4086.25087807436 0.360808

4086.26894419596 0.6129616

4086.28701031756 0.6004653

4086.30507643917 0.8233525

4086.32314256077 0.7739422

4086.34120868237 0.6028847

4086.35927480397 0.9726045

4086.37734092558 1.089386

4086.39540704718 0.869131

4086.41347316878 0.6421321

4086.43153929038 0.6376256

4086.44960541198 0.3941629

4086.46767153359 0.4970382

4086.48573765519 0.6475972

4086.50380377679 0.7198017

4086.52186989839 0.6044858

4086.53993602 0.4610907

4086.5580021416 0.7780555

4086.5760682632 0.8827519

4086.5941343848 0.573878

4086.6122005064 0.6603571

4086.63026662801 0.7481361

4086.64833274961 0.6142014

4086.66639887121 0.5760151

4086.68446499281 0.5068696

4086.70253111442 0.3756002

4086.72059723602 0.4240384

4086.73866335762 0.6346

4086.75672947922 0.864126

4086.77479560082 1.157291

4086.79286172243 0.9019096

4086.81092784403 0.8613568

4086.82899396563 0.7851628

4086.84706008723 0.1959125

4086.86512620884 0.3198733

4086.88319233044 0.4213392

4086.90125845204 0.6354871

4086.91932457364 0.390442

4086.93739069525 0.4440172

4086.95545681685 0.4360527

4086.97352293845 0.6007476

4086.99158906005 0.8497818

4087.00965518165 0.7872094

4087.02772130325 0.6068963

4087.04578742486 0.6705729

4087.06385354646 0.649284

4087.08191966806 0.3132346

4087.09998578966 0.3444062

4087.11805191127 0.8672764

4087.13611803287 0.9926164

4087.15418415447 0.6162607

4087.17225027607 0.5560449

4087.19031639767 0.6277057

4087.20838251928 0.561419

4087.22644864088 0.9057956

4087.24451476248 0.6340931

4087.26258088408 1.022798

4087.28064700569 1.005262

4087.29871312729 0.8189705

4087.31677924889 0.898742

4087.33484537049 0.9587659

4087.35291149209 0.7294216

4087.3709776137 0.849865

4087.3890437353 0.6975864

4087.4071098569 0.5708457

4087.4251759785 0.3850364

4087.44324210011 0.2679725

4087.46130822171 0.6056918

4087.47937434331 0.7415828

4087.49744046491 0.6050361

4087.51550658652 0.4524566

4087.53357270812 0.6140217

4087.55163882972 0.835832

4087.56970495132 0.6492476

4087.58777107292 0.6713799

4087.60583719453 0.5753148

4087.62390331613 0.5449648

4087.64196943773 0.8545129

4087.66003555933 1.015489

4087.67810168093 0.8330895

4087.69616780254 0.5898889

4087.71423392414 0.4988578

4087.73230004574 0.7208976

4087.75036616734 0.7032877

4087.76843228895 0.7111259

4087.78649841055 0.8385152

4087.80456453215 0.9525984

4087.82263065375 0.533352

4087.84069677535 0.1886116

4087.85876289696 0.1643697

4087.87682901856 0.5509545

4087.89489514016 0.6404538

4087.91296126176 0.650856

4087.93102738336 0.5222776

4087.94909350497 0.5387383

4087.96715962657 0.7597665

4087.98522574817 0.6235441

4088.00329186977 0.6058428

4088.02135799138 0.591216

4088.03942411298 0.6469045

4088.05749023458 0.4754609

4088.07555635618 0.3991207

4088.09362247778 0.4885882

4088.11168859939 0.7625263

4088.12975472099 1.15886

4088.14782084259 0.9147769

4088.16588696419 0.9204705

4088.1839530858 0.8622318

4088.2020192074 0.5909535

4088.220085329 0.7381665

4088.2381514506 0.8267382

4088.2562175722 0.7793756

4088.27428369381 0.7237788

4088.29234981541 0.7539463

4088.31041593701 0.8589851

4088.32848205861 0.8463079

4088.34654818022 0.6354153

4088.36461430182 0.4221807

4088.38268042342 0.4178749

4088.40074654502 0.7162138

4088.41881266662 0.8170111

4088.43687878823 0.5483326

4088.45494490983 0.4693065

4088.47301103143 0.3686308

4088.49107715303 0.4227485

4088.50914327464 0.7184728

4088.52720939624 0.8036327

4088.54527551784 0.8755334

4088.56334163944 0.7996948

4088.58140776104 1.044214

4088.59947388265 0.889738

4088.61754000425 0.9939262

4088.63560612585 0.6674635

4088.65367224745 0.5128921

4088.67173836905 0.5224184

4088.68980449066 0.6129435

4088.70787061226 0.8004994

4088.72593673386 0.7987975

4088.74400285546 0.7470945

4088.76206897707 0.9253231

4088.78013509867 0.948731

4088.79820122027 0.8306736

4088.81626734187 0.7284449

4088.83433346347 0.6088885

4088.85239958508 0.5840074

4088.87046570668 0.5285813

4088.88853182828 0.5121293

4088.90659794988 0.7043734

4088.92466407149 0.7810197

4088.94273019309 0.6835952

4088.96079631469 0.7790481

4088.97886243629 0.9338131

4088.99692855789 0.9114006

4089.0149946795 0.3575556

4089.0330608011 0.5352497

4089.0511269227 0.827029

4089.0691930443 0.7080917

4089.08725916591 0.5280595

4089.10532528751 0.4377825

4089.12339140911 0.7115449

4089.14145753071 0.5989236

4089.15952365232 0.5324992

4089.17758977392 0.663641

4089.19565589552 0.6670953

4089.21372201712 0.6290016

4089.23178813872 0.662579

4089.24985426032 0.6635893

4089.26792038193 0.515885

4089.28598650353 0.5751013

4089.30405262513 0.9091865

4089.32211874673 0.9982183

4089.34018486834 0.8706323

4089.35825098994 0.8251013

4089.37631711154 0.7037185

4089.39438323314 0.4919648

4089.41244935474 0.5865065

4089.43051547635 0.4703241

4089.44858159795 0.7566848

4089.46664771955 0.8450902

4089.48471384115 0.5581156

4089.50277996276 0.5431453

4089.52084608436 0.895903

4089.53891220596 1.09403

4089.55697832756 1.137142

4089.57504444916 0.5723794

4089.59311057077 0.5860444

4089.61117669237 0.7218459

4089.62924281397 0.6618971

4089.64730893557 0.8633676

4089.66537505718 0.6673105

4089.68344117878 0.5204722

4089.70150730038 0.6605667

4089.71957342198 0.415312

4089.73763954358 0.2109939

4089.75570566519 0.2230022

4089.77377178679 0.3558584

4089.79183790839 0.6815679

4089.80990402999 0.6844667

4089.8279701516 0.585346

4089.8460362732 0.6774831

4089.8641023948 0.9962363

4089.8821685164 0.9610987

4089.900234638 0.8592789

4089.91830075961 0.7318608

4089.93636688121 0.5503265

4089.95443300281 0.8135214

4089.97249912441 0.9419282

4089.99056524602 0.7725298

4090.00863136762 0.7981893

4090.02669748922 0.8106004

4090.04476361082 0.8166534

4090.06282973242 0.8783103

4090.08089585403 0.7848192

4090.09896197563 0.7526371

4090.11702809723 0.7062915

4090.13509421883 0.6293337

4090.15316034044 0.5212778

4090.17122646204 0.361651

4090.18929258364 0.6178753

4090.20735870524 0.7670965

4090.22542482684 0.5632083

4090.24349094845 0.7290683

4090.26155707005 0.7116373

4090.27962319165 0.6689624

4090.29768931325 0.7405659

4090.31575543485 0.4736794

4090.33382155646 0.5966756

4090.35188767806 0.717258

4090.36995379966 0.767169

4090.38801992126 0.6212018

4090.40608604287 0.5688262

4090.42415216447 0.6878996

4090.44221828607 0.9336063

4090.46028440767 0.9708943

4090.47835052928 0.8448047

4090.49641665088 0.6030096

4090.51448277248 0.6955827

4090.53254889408 0.7191013

4090.55061501568 0.5649278

4090.56868113729 0.5158377

4090.58674725889 0.6851818

4090.60481338049 0.5543153

4090.62287950209 0.473251

4090.64094562369 0.7627839

4090.6590117453 0.9728903

4090.6770778669 1.048983

4090.6951439885 0.9805071

4090.7132101101 0.8827518

4090.73127623171 0.8952221

4090.74934235331 0.9365741

4090.76740847491 0.8834997

4090.78547459651 0.7445831

4090.80354071812 0.6763442

4090.82160683972 0.6224907

4090.83967296132 0.5198128

4090.85773908292 0.5418431

4090.87580520452 0.554765

4090.89387132613 0.6121346

4090.91193744773 0.8189701

4090.93000356933 0.9537635

4090.94806969093 0.9923009

4090.96613581253 0.8260536

4090.98420193414 0.5650291

4091.00226805574 0.6406867

4091.02033417734 0.7165593

4091.03840029894 0.6882277

4091.05646642055 0.5397637

4091.07453254215 0.619952

4091.09259866375 0.680645

4091.11066478535 0.5365306

4091.12873090695 0.6623655

4091.14679702856 0.6951808

4091.16486315016 0.6465631

4091.18292927176 0.7699648

4091.20099539336 0.7491553

4091.21906151496 0.7341261

4091.23712763657 0.5785697

4091.25519375817 0.7720274

4091.27325987977 0.7982486

4091.29132600137 0.6952872

4091.30939212298 0.6891499

4091.32745824458 0.4138549

4091.34552436618 0.576958

4091.36359048778 0.8247896

4091.38165660938 0.8047148

4091.39972273099 0.8913515

4091.41778885259 0.7973195

4091.43585497419 0.856186

4091.45392109579 0.5911291

4091.4719872174 0.3898964

4091.490053339 0.5715342

4091.5081194606 0.7460518

4091.5261855822 0.4095766

4091.5442517038 0.3558042

4091.56231782541 0.6392792

4091.58038394701 0.5592176

4091.59845006861 0.4435418

4091.61651619021 0.4906617

4091.63458231182 0.5577042

4091.65264843342 0.6164526

4091.67071455502 0.6115553

4091.68878067662 0.6467827

4091.70684679823 0.6637515

4091.72491291983 0.7332075

4091.74297904143 0.8875009

4091.76104516303 0.8831872

4091.77911128463 0.5980577

4091.79717740624 0.4602807

4091.81524352784 0.4856672

4091.83330964944 0.6689767

4091.85137577104 0.557494

4091.86944189264 0.6595996

4091.88750801425 0.6589496

4091.90557413585 0.6293072

4091.92364025745 0.5889801

4091.94170637905 0.5787289

4091.95977250066 0.6860654

4091.97783862226 0.7322649

4091.99590474386 0.7683408

4092.01397086546 0.7220141

4092.03203698706 0.5311255

4092.05010310867 0.3570527

4092.06816923027 0.451386

4092.08623535187 0.5346407

4092.10430147347 0.55549

4092.12236759507 0.5344172

4092.14043371668 0.3612306

4092.15849983828 0.3972844

4092.17656595988 0.4964707

4092.19463208148 0.749743

4092.21269820309 0.779567

4092.23076432469 0.7933775

4092.24883044629 0.5566018

4092.26689656789 0.3674917

4092.28496268949 0.6711744

4092.3030288111 0.6947644

4092.3210949327 0.6505353

4092.3391610543 0.749301

4092.3572271759 0.8049716

4092.37529329751 0.6325923

4092.39335941911 0.7188706

4092.41142554071 0.9043239

4092.42949166231 0.8598374

4092.44755778391 0.7246832

4092.46562390552 0.6607819

4092.48369002712 0.4917067

4092.50175614872 0.4619471

4092.51982227032 0.6793618

4092.53788839193 0.8290721

4092.55595451353 0.6879618

4092.57402063513 0.5936019

4092.59208675673 0.6985925

4092.61015287833 0.8252881

4092.62821899994 0.9697535

4092.64628512154 0.8241985

4092.66435124314 0.5305605

4092.68241736474 0.5229803

4092.70048348635 0.6939182

4092.71854960795 0.7630438

4092.73661572955 0.6076978

4092.75468185115 0.7866917

4092.77274797275 0.9078383

4092.79081409436 0.7357674

4092.80888021596 0.6141081

4092.82694633756 0.7117271

4092.84501245916 0.7450238

4092.86307858076 0.5992676

4092.88114470237 0.6803551

4092.89921082397 0.7579471

4092.91727694557 0.7049516

4092.93534306717 0.7556208

4092.95340918878 0.8215395

4092.97147531038 0.6424424

4092.98954143198 0.4748785

4093.00760755358 0.4732757

4093.02567367518 0.6120483

4093.04373979679 0.6331664

4093.06180591839 0.7188057

4093.07987203999 0.8093151

4093.09793816159 0.7352315

4093.1160042832 0.8261884

4093.1340704048 0.7480152

4093.1521365264 0.5549006

4093.170202648 0.6277997

4093.1882687696 0.5387875

4093.20633489121 0.4394288

4093.22440101281 0.6569446

4093.24246713441 0.7151636

4093.26053325601 0.7177698

4093.27859937762 0.5785943

4093.29666549922 0.630082

4093.31473162082 0.7969765

4093.33279774242 0.9480073

4093.35086386402 0.7985477

4093.36892998563 0.6010055

4093.38699610723 0.5279057

4093.40506222883 0.5368016

4093.42312835043 0.5519918

4093.44119447203 0.6545163

4093.45926059364 0.5135149

4093.47732671524 0.4686889

4093.49539283684 0.609474

4093.51345895844 0.6810685

4093.53152508005 0.5942329

4093.54959120165 0.4950503

4093.56765732325 0.5727373

4093.58572344485 0.5859531

4093.60378956645 0.4587229

4093.62185568806 0.5982168

4093.63992180966 0.6640046

4093.65798793126 0.4832914

4093.67605405286 0.5899665

4093.69412017447 0.6451614

4093.71218629607 0.6770427

4093.73025241767 0.6821966

4093.74831853927 0.7344261

4093.76638466087 0.5946062

4093.78445078248 0.6091765

4093.80251690408 0.4423051

4093.82058302568 0.6228663

4093.83864914728 0.6570735

4093.85671526889 0.508015

4093.87478139049 0.494152

4093.89284751209 0.6445215

4093.91091363369 0.6303679

4093.9289797553 0.5810469

4093.9470458769 0.6331294

4093.9651119985 0.7453521

4093.9831781201 0.8153808

4094.0012442417 0.7927418

4094.01931036331 0.6383505

4094.03737648491 0.5806603

4094.05544260651 0.6112414

4094.07350872811 0.5389229

4094.09157484972 0.4065103

4094.10964097132 0.4615858

4094.12770709292 0.597882

4094.14577321452 0.5721539

4094.16383933612 0.5393513

4094.18190545773 0.5931448

4094.19997157933 0.6661245

4094.21803770093 0.6462037

4094.23610382253 0.6306287

4094.25416994413 0.5570047

4094.27223606574 0.651319

4094.29030218734 0.7240499

4094.30836830894 0.6969464

4094.32643443054 0.5760629

4094.34450055215 0.7025851

4094.36256667375 0.5351105

4094.38063279535 0.5486291

4094.39869891695 0.5636181

4094.41676503855 0.7195056

4094.43483116015 0.7124606

4094.45289728176 0.6248712

4094.47096340336 0.5876628

4094.48902952496 0.7219734

4094.50709564656 0.665881

4094.52516176817 0.6204486

4094.54322788977 0.7906728

4094.56129401137 0.7050108

4094.57936013297 0.544693

4094.59742625458 0.6222149

4094.61549237618 0.6258592

4094.63355849778 0.6317195

4094.65162461938 0.7010938

4094.66969074098 0.6634372

4094.68775686259 0.6077363

4094.70582298419 0.6290837

4094.72388910579 0.6496522

4094.74195522739 0.4763307

4094.760021349 0.5441024

4094.7780874706 0.6610959

4094.7961535922 0.5639786

4094.8142197138 0.5692704

4094.8322858354 0.7086249

4094.85035195701 0.6433926

4094.86841807861 0.6014825

4094.88648420021 0.6204192

4094.90455032181 0.5345527

4094.92261644342 0.5269095

4094.94068256502 0.6348485

4094.95874868662 0.739305

4094.97681480822 0.6310629

4094.99488092983 0.5461491

4095.01294705143 0.7074822

4095.03101317303 0.8306231

4095.04907929463 0.8201048

4095.06714541623 0.7178395

4095.08521153784 0.4701957

4095.10327765944 0.5055605

4095.12134378104 0.7326447

4095.13940990264 0.7075229

4095.15747602424 0.5980811

4095.17554214585 0.6016701

4095.19360826745 0.6318983

4095.21167438905 0.6689082

4095.22974051065 0.6794114

4095.24780663226 0.5891606

4095.26587275386 0.6190715

4095.28393887546 0.7436607

4095.30200499706 0.6481587

4095.32007111866 0.6138991

4095.33813724027 0.586868

4095.35620336187 0.503522

4095.37426948347 0.5624495

4095.39233560507 0.6844236

4095.41040172667 0.6300031

4095.42846784828 0.4876076

4095.44653396988 0.5812343

4095.46460009148 0.7352228

4095.48266621308 0.7118945

4095.50073233469 0.6113221

4095.51879845629 0.6589654

4095.53686457789 0.6209581

4095.55493069949 0.6314521

4095.57299682109 0.671149

4095.5910629427 0.7459854

4095.6091290643 0.8176711

4095.6271951859 0.7568206

4095.6452613075 0.7184961

4095.66332742911 0.7476454

4095.68139355071 0.7743346

4095.69945967231 0.7150019

4095.71752579391 0.6055747

4095.73559191552 0.6014841

4095.75365803712 0.6567805

4095.77172415872 0.5652692

4095.78979028032 0.4701385

4095.80785640192 0.5095455

4095.82592252353 0.5058638

4095.84398864513 0.6152226

4095.86205476673 0.6588895

4095.88012088833 0.6784891

4095.89818700994 0.580729

4095.91625313154 0.5611152

4095.93431925314 0.4976561

4095.95238537474 0.6021507

4095.97045149634 0.5169151

4095.98851761795 0.4971992

4096.00658373955 0.4877041

4096.02464986115 0.5993589

4096.04271598275 0.6102503

4096.06078210435 0.5822678

4096.07884822596 0.5408322

4096.09691434756 0.5430853

4096.11498046916 0.6538644

4096.13304659076 0.573481

4096.15111271236 0.5443374

4096.16917883397 0.5525733

4096.18724495557 0.538509

4096.20531107717 0.527865

4096.22337719877 0.6183423

4096.24144332038 0.731488

4096.25950944198 0.728784

4096.27757556358 0.738792

4096.29564168518 0.734305

4096.31370780678 0.6315516

4096.33177392839 0.651929

4096.34984004999 0.7153753

4096.36790617159 0.6524732

4096.38597229319 0.6540188

4096.40403841479 0.6867126

4096.4221045364 0.7823368

4096.440170658 0.652624

4096.4582367796 0.4232613

4096.4763029012 0.5267836

4096.49436902281 0.6953655

4096.51243514441 0.7031571

4096.53050126601 0.5236135

4096.54856738761 0.5967049

4096.56663350922 0.5907009

4096.58469963082 0.4906579

4096.60276575242 0.6849666

4096.62083187402 0.6664135

4096.63889799562 0.5876043

4096.65696411723 0.6333706

4096.67503023883 0.6554767

4096.69309636043 0.6364409

4096.71116248203 0.5737972

4096.72922860364 0.5118356

4096.74729472524 0.5470312

4096.76536084684 0.5230288

4096.78342696844 0.5192293

4096.80149309004 0.5808758

4096.81955921165 0.6019209

4096.83762533325 0.6118392

4096.85569145485 0.5889098

4096.87375757645 0.6354877

4096.89182369806 0.6472145

4096.90988981966 0.6028703

4096.92795594126 0.6761832

4096.94602206286 0.6955248

4096.96408818446 0.7361822

4096.98215430607 0.695564

4097.00022042767 0.6396467

4097.01828654927 0.6056125

4097.03635267087 0.6736397

4097.05441879247 0.7616755

4097.07248491408 0.6338154

4097.09055103568 0.5656616

4097.10861715728 0.533353

4097.12668327888 0.548149

4097.14474940049 0.6076881

4097.16281552209 0.6508005

4097.18088164369 0.5103565

4097.19894776529 0.5259508

4097.21701388689 0.5955654

4097.2350800085 0.6632991

4097.2531461301 0.6748873

4097.2712122517 0.6901489

4097.2892783733 0.5979607

4097.30734449491 0.5672852

4097.32541061651 0.5649538

4097.34347673811 0.5382621

4097.36154285971 0.5557407

4097.37960898131 0.6660718

4097.39767510292 0.6370601

4097.41574122452 0.5461354

4097.43380734612 0.5236165

4097.45187346772 0.5963815

4097.46993958933 0.6900412

4097.48800571093 0.6190889

4097.50607183253 0.5650182

4097.52413795413 0.5621817

4097.54220407573 0.5764995

4097.56027019734 0.603238

4097.57833631894 0.6108558

4097.59640244054 0.6652718

4097.61446856214 0.5322133

4097.63253468374 0.473862

4097.65060080535 0.5020453

4097.66866692695 0.5986597

4097.68673304855 0.7064307

4097.70479917015 0.6185825

4097.72286529176 0.5356977

4097.74093141336 0.506174

4097.75899753496 0.496689

4097.77706365656 0.5556926

4097.79512977816 0.6705397

4097.81319589977 0.6311966

4097.83126202137 0.5558085

4097.84932814297 0.5822862

4097.86739426457 0.5402612

4097.88546038618 0.536116

4097.90352650778 0.5521874

4097.92159262938 0.6359563

4097.93965875098 0.7066997

4097.95772487258 0.6065387

4097.97579099419 0.5479816

4097.99385711579 0.6380441

4098.01192323739 0.642433

4098.02998935899 0.6572191

4098.0480554806 0.6117843

4098.0661216022 0.5023488

4098.0841877238 0.5608891

4098.1022538454 0.6056882

4098.120319967 0.5363159

4098.13838608861 0.4675775

4098.15645221021 0.49899

4098.17451833181 0.5532673

4098.19258445341 0.6226422

4098.21065057501 0.6215574

4098.22871669662 0.5603264

4098.24678281822 0.5868163

4098.26484893982 0.5250646

4098.28291506142 0.6120928

4098.30098118303 0.6000128

4098.31904730463 0.6125131

4098.33711342623 0.5783586

4098.35517954783 0.5140985

4098.37324566943 0.5587842

4098.39131179104 0.4833337

4098.40937791264 0.499661

4098.42744403424 0.6128178

4098.44551015584 0.5340226

4098.46357627745 0.4467027

4098.48164239905 0.50859

4098.49970852065 0.5678155

4098.51777464225 0.4987425

4098.53584076386 0.4735405

4098.55390688546 0.5146706

4098.57197300706 0.5016618

4098.59003912866 0.488674

4098.60810525026 0.4694457

4098.62617137187 0.4882679

4098.64423749347 0.5200523

4098.66230361507 0.5242451

4098.68036973667 0.4576038

4098.69843585827 0.3621713

4098.71650197988 0.2955995

4098.73456810148 0.337679

4098.75263422308 0.4984401

4098.77070034468 0.5184189

4098.78876646629 0.5374603

4098.80683258789 0.5331383

4098.82489870949 0.5264124

4098.84296483109 0.5479001

4098.8610309527 0.5027769

4098.8790970743 0.5092418

4098.8971631959 0.5507197

4098.9152293175 0.5572983

4098.9332954391 0.6130137

4098.95136156071 0.5564205

4098.96942768231 0.4683828

4098.98749380391 0.5027828

4099.00555992551 0.5956343

4099.02362604712 0.579357

4099.04169216872 0.5182022

4099.05975829032 0.4633419

4099.07782441192 0.5233759

4099.09589053352 0.5594077

4099.11395665513 0.5233346

4099.13202277673 0.4912178

4099.15008889833 0.4676737

4099.16815501993 0.4321907

4099.18622114154 0.4128703

4099.20428726314 0.4420999

4099.22235338474 0.5200656

4099.24041950634 0.4930615

4099.25848562794 0.5132583

4099.27655174955 0.5725707

4099.29461787115 0.5958892

4099.31268399275 0.5525827

4099.33075011435 0.4848605

4099.34881623595 0.446121

4099.36688235756 0.4743808

4099.38494847916 0.5277029

4099.40301460076 0.4221447

4099.42108072236 0.4307939

4099.43914684397 0.4866933

4099.45721296557 0.5174595

4099.47527908717 0.4620881

4099.49334520877 0.4611604

4099.51141133037 0.5149585

4099.52947745198 0.5078223

4099.54754357358 0.463934

4099.56560969518 0.4306103

4099.58367581678 0.3562451

4099.60174193838 0.425385

4099.61980805999 0.4996809

4099.63787418159 0.517554

4099.65594030319 0.511047

4099.67400642479 0.5112688

4099.6920725464 0.4794266

4099.710138668 0.4450914

4099.7282047896 0.4323982

4099.7462709112 0.4200768

4099.7643370328 0.4553628

4099.78240315441 0.3851112

4099.80046927601 0.3598054

4099.81853539761 0.4409909

4099.83660151921 0.464178

4099.85466764082 0.4581656

4099.87273376242 0.359935

4099.89079988402 0.4469869

4099.90886600562 0.5756472

4099.92693212722 0.4633785

4099.94499824883 0.4070625

4099.96306437043 0.4663267

4099.98113049203 0.4200771

4099.99919661363 0.3789389

4100.01726273524 0.4022343

4100.03532885684 0.352267

4100.05339497844 0.3497314

4100.07146110004 0.4312559

4100.08952722164 0.4367611

4100.10759334325 0.3894045

4100.12565946485 0.3936093

4100.14372558645 0.4163897

4100.16179170805 0.3757983

4100.17985782965 0.3321475

4100.19792395126 0.4135379

4100.21599007286 0.3740652

4100.23405619446 0.3562396

4100.25212231606 0.3450206

4100.27018843767 0.2786626

4100.28825455927 0.2594889

4100.30632068087 0.4012654

4100.32438680247 0.3717746

4100.34245292407 0.3645332

4100.36051904568 0.3958549

4100.37858516728 0.4326347

4100.39665128888 0.4130268

4100.41471741048 0.3601312

4100.43278353209 0.3937577

4100.45084965369 0.3811353

4100.46891577529 0.3697743

4100.48698189689 0.3313046

4100.5050480185 0.3092756

4100.5231141401 0.3485858

4100.5411802617 0.3841805

4100.5592463833 0.3317216

4100.5773125049 0.3591154

4100.5953786265 0.4207539

4100.61344474811 0.3977635

4100.63151086971 0.3430447

4100.64957699131 0.4121756

4100.66764311291 0.3973121

4100.68570923452 0.305091

4100.70377535612 0.3986789

4100.72184147772 0.4129376

4100.73990759932 0.3514844

4100.75797372093 0.3864261

4100.77603984253 0.4353689

4100.79410596413 0.397043

4100.81217208573 0.3938457

4100.83023820733 0.3871755

4100.84830432894 0.3866617

4100.86637045054 0.3972318

4100.88443657214 0.4053438

4100.90250269374 0.3540872

4100.92056881535 0.2488169

4100.93863493695 0.1959289

4100.95670105855 0.2368708

4100.97476718015 0.3043781

4100.99283330175 0.3169132

4101.01089942336 0.3445367

4101.02896554496 0.3479111

4101.04703166656 0.3877904

4101.06509778816 0.3160121

4101.08316390977 0.258776

4101.10123003137 0.2499782

4101.11929615297 0.2825614

4101.13736227457 0.309063

4101.15542839617 0.2559087

4101.17349451778 0.2651131

4101.19156063938 0.2567333

4101.20962676098 0.3349172

4101.22769288258 0.3713343

4101.24575900418 0.3504512

4101.26382512579 0.3218997

4101.28189124739 0.2431716

4101.29995736899 0.2544765

4101.31802349059 0.295242

4101.3360896122 0.3212185

4101.3541557338 0.3005452

4101.3722218554 0.2412087

4101.390287977 0.1685089

4101.4083540986 0.1608986

4101.42642022021 0.2730152

4101.44448634181 0.2547776

4101.46255246341 0.1515808

4101.48061858501 0.1370292

4101.49868470662 0.2724185

4101.51675082822 0.3862579

4101.53481694982 0.3459582

4101.55288307142 0.2397566

4101.57094919303 0.1906096

4101.58901531463 0.265118

4101.60708143623 0.2709326

4101.62514755783 0.1560536

4101.64321367943 0.1655951

4101.66127980104 0.1569137

4101.67934592264 0.2090442

4101.69741204424 0.1621223

4101.71547816584 0.1390002

4101.73354428745 0.2125848

4101.75161040905 0.1892205

4101.76967653065 0.1467218

4101.78774265225 0.165111

4101.80580877385 0.1836558

4101.82387489545 0.1642641

4101.84194101706 0.13453

4101.86000713866 0.1474396

4101.87807326026 0.182873

4101.89613938186 0.2113417

4101.91420550347 0.226535

4101.93227162507 0.1500604

4101.95033774667 0.1448976

4101.96840386827 0.2203155

4101.98646998988 0.2097268

4102.00453611148 0.1551677

4102.02260223308 0.1782107

4102.04066835468 0.1949483

4102.05873447628 0.208125

4102.07680059789 0.2310934

4102.09486671949 0.2360074

4102.11293284109 0.2665995

4102.13099896269 0.2798601

4102.14906508429 0.2751968

4102.1671312059 0.2606125

4102.1851973275 0.2085958

4102.2032634491 0.135573

4102.2213295707 0.09978679

4102.23939569231 0.1750891

4102.25746181391 0.2150886

4102.27552793551 0.2094874

4102.29359405711 0.1859942

4102.31166017871 0.1378829

4102.32972630032 0.2136231

4102.34779242192 0.2137464

4102.36585854352 0.2793295

4102.38392466512 0.2574609

4102.40199078673 0.228377

4102.42005690833 0.1520941

4102.43812302993 0.2397023

4102.45618915153 0.2942611

4102.47425527313 0.3276831

4102.49232139474 0.2433535

4102.51038751634 0.2419871

4102.52845363794 0.247432

4102.54651975954 0.2430342

4102.56458588114 0.2052922

4102.58265200275 0.2274115

4102.60071812435 0.2686866

4102.61878424595 0.2547229

4102.63685036755 0.2766633

4102.65491648916 0.3136144

4102.67298261076 0.2869509

4102.69104873236 0.2894841

4102.70911485396 0.2782443

4102.72718097557 0.2845185

4102.74524709717 0.2779581

4102.76331321877 0.3006234

4102.78137934037 0.3299353

4102.79944546197 0.3510945

4102.81751158357 0.3366721

4102.83557770518 0.2839948

4102.85364382678 0.3282757

4102.87170994838 0.3253721

4102.88977606998 0.3083934

4102.90784219159 0.3706301

4102.92590831319 0.360016

4102.94397443479 0.3684673

4102.96204055639 0.3796566

4102.98010667799 0.4142048

4102.9981727996 0.4217557

4103.0162389212 0.3552243

4103.0343050428 0.4078753

4103.05237116441 0.3632818

4103.07043728601 0.3671667

4103.08850340761 0.4016002

4103.10656952921 0.3659542

4103.12463565081 0.3161781

4103.14270177242 0.333536

4103.16076789402 0.3696126

4103.17883401562 0.380415

4103.19690013722 0.3642461

4103.21496625882 0.397491

4103.23303238043 0.3750378

4103.25109850203 0.3242128

4103.26916462363 0.3641836

4103.28723074523 0.4283319

4103.30529686684 0.4374703

4103.32336298844 0.4010845

4103.34142911004 0.3669582

4103.35949523164 0.3174587

4103.37756135324 0.4027549

4103.39562747485 0.4724685

4103.41369359645 0.4561883

4103.43175971805 0.4219791

4103.44982583965 0.3951591

4103.46789196126 0.3560174

4103.48595808286 0.3276719

4103.50402420446 0.3720151

4103.52209032606 0.3511651

4103.54015644766 0.3826706

4103.55822256927 0.4420326

4103.57628869087 0.3037636

4103.59435481247 0.3062209

4103.61242093407 0.440339

4103.63048705568 0.4099582

4103.64855317728 0.3648809

4103.66661929888 0.3708032

4103.68468542048 0.3843905

4103.70275154208 0.477724

4103.72081766369 0.4875259

4103.73888378529 0.3805788

4103.75694990689 0.3865999

4103.77501602849 0.3980218

4103.79308215009 0.3522189

4103.8111482717 0.3761512

4103.8292143933 0.4361843

4103.8472805149 0.4738954

4103.8653466365 0.5076985

4103.88341275811 0.4835129

4103.90147887971 0.3927021

4103.91954500131 0.4043939

4103.93761112291 0.4127952

4103.95567724452 0.4029419

4103.97374336612 0.4053943

4103.99180948772 0.374175

4104.00987560932 0.355217

4104.02794173092 0.4379669

4104.04600785253 0.4468847

4104.06407397413 0.4560243

4104.08214009573 0.4599076

4104.10020621733 0.477526

4104.11827233894 0.4776354

4104.13633846054 0.462095

4104.15440458214 0.4478942

4104.17247070374 0.4404771

4104.19053682534 0.5056021

4104.20860294695 0.4809683

4104.22666906855 0.4662076

4104.24473519015 0.4289828

4104.26280131175 0.4719262

4104.28086743335 0.5052674

4104.29893355496 0.4744103

4104.31699967656 0.4671977

4104.33506579816 0.442807

4104.35313191976 0.4475199

4104.37119804137 0.503337

4104.38926416297 0.5166686

4104.40733028457 0.4779803

4104.42539640617 0.5224078

4104.44346252777 0.5122804

4104.46152864938 0.4786111

4104.47959477098 0.5032114

4104.49766089258 0.5160769

4104.51572701418 0.5111166

4104.53379313578 0.5032186

4104.55185925739 0.5185458

4104.56992537899 0.5083491

4104.58799150059 0.4603698

4104.60605762219 0.4921549

4104.6241237438 0.535583

4104.6421898654 0.4944397

4104.660255987 0.4724001

4104.6783221086 0.4616745

4104.6963882302 0.472071

4104.71445435181 0.4844442

4104.73252047341 0.4943836

4104.75058659501 0.4591155

4104.76865271661 0.4561596

4104.78671883822 0.4737235

4104.80478495982 0.5147177

4104.82285108142 0.5140349

4104.84091720302 0.5116913

4104.85898332462 0.5202016

4104.87704944623 0.5231839

4104.89511556783 0.505361

4104.91318168943 0.5124636

4104.93124781103 0.5147091

4104.94931393263 0.5117947

4104.96738005424 0.5288029

4104.98544617584 0.4616233

4105.00351229744 0.4645227

4105.02157841904 0.5558227

4105.03964454065 0.5753983

4105.05771066225 0.5918675

4105.07577678385 0.5397121

4105.09384290545 0.5309541

4105.11190902705 0.5304903

4105.12997514866 0.5309058

4105.14804127026 0.5450464

4105.16610739186 0.5278231

4105.18417351346 0.5393411

4105.20223963507 0.5396428

4105.22030575667 0.5001978

4105.23837187827 0.5194178

4105.25643799987 0.5568479

4105.27450412148 0.5207493

4105.29257024308 0.4722302

4105.31063636468 0.551149

4105.32870248628 0.597351

4105.34676860788 0.5758992

4105.36483472949 0.5257815

4105.38290085109 0.5576027

4105.40096697269 0.6128708

4105.41903309429 0.5391269

4105.43709921589 0.5302023

4105.4551653375 0.5828748

4105.4732314591 0.6002098

4105.4912975807 0.5649676

4105.5093637023 0.499711

4105.52742982391 0.5571797

4105.54549594551 0.5776641

4105.56356206711 0.5458404

4105.58162818871 0.5665348

4105.59969431031 0.5061746

4105.61776043192 0.503422

4105.63582655352 0.5748327

4105.65389267512 0.6010119

4105.67195879672 0.6118795

4105.69002491833 0.5929753

4105.70809103993 0.5788332

4105.72615716153 0.5941262

4105.74422328313 0.6514845

4105.76228940474 0.5957536

4105.78035552634 0.630411

4105.79842164794 0.6442421

4105.81648776954 0.5751275

4105.83455389114 0.6206345

4105.85262001275 0.5937198

4105.87068613435 0.5016666

4105.88875225595 0.6104231

4105.90681837755 0.5510786

4105.92488449916 0.5596187

4105.94295062076 0.6781886

4105.96101674236 0.6179932

4105.97908286396 0.5562304

4105.99714898556 0.5857287

4106.01521510716 0.6387532

4106.03328122877 0.6592737

4106.05134735037 0.6522869

4106.06941347197 0.6326958

4106.08747959357 0.6899937

4106.10554571518 0.6563517

4106.12361183678 0.631879

4106.14167795838 0.6028709

4106.15974407998 0.6341874

4106.17781020158 0.630614

4106.19587632319 0.6088107

4106.21394244479 0.6083972

4106.23200856639 0.6204453

4106.25007468799 0.6583765

4106.2681408096 0.6098182

4106.2862069312 0.668909

4106.3042730528 0.6709508

4106.3223391744 0.6047815

4106.34040529601 0.6115136

4106.35847141761 0.5790024

4106.37653753921 0.6190611

4106.39460366081 0.7017821

4106.41266978241 0.6925961

4106.43073590401 0.6428071

4106.44880202562 0.6007819

4106.46686814722 0.5882707

4106.48493426882 0.5966859

4106.50300039042 0.6115448

4106.52106651203 0.6067154

4106.53913263363 0.5984601

4106.55719875523 0.6490162

4106.57526487683 0.6550651

4106.59333099844 0.6124551

4106.61139712004 0.6572332

4106.62946324164 0.6818938

4106.64752936324 0.7200698

4106.66559548484 0.6605466

4106.68366160645 0.6248655

4106.70172772805 0.6584175

4106.71979384965 0.6093433

4106.73785997125 0.5803366

4106.75592609286 0.6461361

4106.77399221446 0.6395196

4106.79205833606 0.6088962

4106.81012445766 0.609308

4106.82819057926 0.6283824

4106.84625670087 0.6981361

4106.86432282247 0.6481708

4106.88238894407 0.6209167

4106.90045506567 0.6659333

4106.91852118728 0.667996

4106.93658730888 0.6380232

4106.95465343048 0.6520484

4106.97271955208 0.6660998

4106.99078567368 0.6762419

4107.00885179529 0.5925691

4107.02691791689 0.5707191

4107.04498403849 0.6151286

4107.06305016009 0.6831609

4107.08111628169 0.6731048

4107.0991824033 0.6169999

4107.1172485249 0.637584

4107.1353146465 0.6125873

4107.1533807681 0.6152026

4107.17144688971 0.5952839

4107.18951301131 0.60927

4107.20757913291 0.6636077

4107.22564525451 0.6532681

4107.24371137612 0.6346326

4107.26177749772 0.6612828

4107.27984361932 0.7097719

4107.29790974092 0.7266454

4107.31597586252 0.6514534

4107.33404198413 0.6387184

4107.35210810573 0.6281623

4107.37017422733 0.6492874

4107.38824034893 0.6847694

4107.40630647053 0.6423005

4107.42437259214 0.6593213

4107.44243871374 0.6692881

4107.46050483534 0.6588263

4107.47857095694 0.7406939

4107.49663707855 0.74504

4107.51470320015 0.7216855

4107.53276932175 0.7255831

4107.55083544335 0.6770911

4107.56890156495 0.6897793

4107.58696768656 0.7022295

4107.60503380816 0.7043417

4107.62309992976 0.7137284

4107.64116605136 0.6849099

4107.65923217297 0.6473328

4107.67729829457 0.6625746

4107.69536441617 0.6997986

4107.71343053777 0.7380127

4107.73149665937 0.7341915

4107.74956278098 0.7478961

4107.76762890258 0.6937785

4107.78569502418 0.6457096

4107.80376114578 0.7121606

4107.82182726739 0.7266289

4107.83989338899 0.6808488

4107.85795951059 0.6701123

4107.87602563219 0.626696

4107.89409175379 0.6856207

4107.9121578754 0.7107397

4107.930223997 0.7209334

4107.9482901186 0.7007682

4107.9663562402 0.6436775

4107.9844223618 0.6943648

4108.00248848341 0.8097469

4108.02055460501 0.7570884

4108.03862072661 0.6719792

4108.05668684821 0.6684635

4108.07475296982 0.7170097

4108.09281909142 0.7229641

4108.11088521302 0.6881944

4108.12895133462 0.6798393

4108.14701745622 0.7717221

4108.16508357783 0.7620239

4108.18314969943 0.6883653

4108.20121582103 0.6148198

4108.21928194263 0.7191483

4108.23734806424 0.7749497

4108.25541418584 0.7553937

4108.27348030744 0.8012763

4108.29154642904 0.7168027

4108.30961255064 0.6488892

4108.32767867225 0.687171

4108.34574479385 0.7039771

4108.36381091545 0.7239891

4108.38187703705 0.7478682

4108.39994315865 0.7945074

4108.41800928026 0.7271844

4108.43607540186 0.6487066

4108.45414152346 0.7252186

4108.47220764506 0.7965777

4108.49027376667 0.7619452

4108.50833988827 0.7309295

4108.52640600987 0.6966059

4108.54447213147 0.6349618

4108.56253825308 0.6887545

4108.58060437468 0.7259535

4108.59867049628 0.6881315

4108.61673661788 0.7029224

4108.63480273948 0.7157753

4108.65286886109 0.7039683

4108.67093498269 0.7312784

4108.68900110429 0.7541894

4108.70706722589 0.7183983

4108.7251333475 0.7199279

4108.7431994691 0.732914

4108.7612655907 0.7320856

4108.7793317123 0.7490085

4108.7973978339 0.7298134

4108.81546395551 0.726248

4108.83353007711 0.7071409

4108.85159619871 0.7165751

4108.86966232031 0.7745916

4108.88772844192 0.7660033

4108.90579456352 0.7047223

4108.92386068512 0.6905611

4108.94192680672 0.7599382

4108.95999292832 0.7441116

4108.97805904993 0.6915634

4108.99612517153 0.7194574

4109.01419129313 0.7167618

4109.03225741473 0.8048637

4109.05032353633 0.8189182

4109.06838965794 0.7422467

4109.08645577954 0.7420169

4109.10452190114 0.753716

4109.12258802274 0.7776753

4109.14065414435 0.7952453

4109.15872026595 0.7889702

4109.17678638755 0.7711707

4109.19485250915 0.7826157

4109.21291863075 0.7733843

4109.23098475236 0.7302749

4109.24905087396 0.7952142

4109.26711699556 0.802269

4109.28518311716 0.7501736

4109.30324923877 0.8027226

4109.32131536037 0.7843072

4109.33938148197 0.7965346

4109.35744760357 0.7624678

4109.37551372517 0.7946442

4109.39357984678 0.7500861

4109.41164596838 0.7361861

4109.42971208998 0.8390442

4109.44777821158 0.8040867

4109.46584433319 0.7854962

4109.48391045479 0.711832

4109.50197657639 0.7132269

4109.52004269799 0.7863379

4109.53810881959 0.7761278

4109.5561749412 0.7615549

4109.5742410628 0.7529486

4109.5923071844 0.7210532

4109.610373306 0.6950943

4109.6284394276 0.7413847

4109.64650554921 0.7289494

4109.66457167081 0.7168987

4109.68263779241 0.7827671

4109.70070391401 0.74319

4109.71877003562 0.7385404

4109.73683615722 0.7491689

4109.75490227882 0.775806

4109.77296840042 0.8072321

4109.79103452202 0.7463863

4109.80910064363 0.7596377

4109.82716676523 0.8083175

4109.84523288683 0.7931046

4109.86329900843 0.8076186

4109.88136513003 0.7743178

4109.89943125164 0.7434737

4109.91749737324 0.8110813

4109.93556349484 0.8573871

4109.95362961644 0.8113617

4109.97169573805 0.7414651

4109.98976185965 0.8172756

4110.00782798125 0.8431254

4110.02589410285 0.7643426

4110.04396022446 0.8356802

4110.06202634606 0.8568932

4110.08009246766 0.8235631

4110.09815858926 0.8365774

4110.11622471086 0.8132457

4110.13429083247 0.8279768

4110.15235695407 0.8364207

4110.17042307567 0.845689

4110.18848919727 0.8731292

4110.20655531887 0.832961

4110.22462144048 0.8019078

4110.24268756208 0.8419941

4110.26075368368 0.807583

4110.27881980528 0.7385969

4110.29688592689 0.7456393

4110.31495204849 0.7338868

4110.33301817009 0.7882265

4110.35108429169 0.8416426

4110.3691504133 0.8241882

4110.3872165349 0.7471451

4110.4052826565 0.8032388

4110.4233487781 0.7898896

4110.4414148997 0.7787797

4110.45948102131 0.7474858

4110.47754714291 0.7758827

4110.49561326451 0.8081274

4110.51367938611 0.7565598

4110.53174550772 0.7069595

4110.54981162932 0.7823967

4110.56787775092 0.8528741

4110.58594387252 0.7822145

4110.60400999412 0.7332612

4110.62207611572 0.7584609

4110.64014223733 0.7726179

4110.65820835893 0.7978294

4110.67627448053 0.8187716

4110.69434060213 0.79382

4110.71240672374 0.8103479

4110.73047284534 0.8273169

4110.74853896694 0.8398312

4110.76660508854 0.8076159

4110.78467121015 0.7987841

4110.80273733175 0.774827

4110.82080345335 0.7586257

4110.83886957495 0.781832

4110.85693569656 0.7491123

4110.87500181816 0.7962695

4110.89306793976 0.7480733

4110.91113406136 0.712038

4110.92920018296 0.7788322

4110.94726630457 0.7902491

4110.96533242617 0.7906375

4110.98339854777 0.8194661

4111.00146466937 0.8512117

4111.01953079097 0.7747183

4111.03759691258 0.8127362

4111.05566303418 0.8471208

4111.07372915578 0.8407629

4111.09179527738 0.850863

4111.10986139899 0.8560035

4111.12792752059 0.8612452

4111.14599364219 0.8490834

4111.16405976379 0.8514265

4111.18212588539 0.8556534

4111.200192007 0.7738157

4111.2182581286 0.7701851

4111.2363242502 0.8232641

4111.2543903718 0.820024

4111.27245649341 0.8364642

4111.29052261501 0.8633776

4111.30858873661 0.8374389

4111.32665485821 0.8068416

4111.34472097981 0.810764

4111.36278710142 0.8165336

4111.38085322302 0.7986117

4111.39891934462 0.7888941

4111.41698546622 0.835467

4111.43505158782 0.8270944

4111.45311770943 0.7834175

4111.47118383103 0.8079561

4111.48924995263 0.8106269

4111.50731607423 0.7937448

4111.52538219584 0.8350944

4111.54344831744 0.878364

4111.56151443904 0.8672879

4111.57958056064 0.8664365

4111.59764668224 0.8789452

4111.61571280385 0.8348045

4111.63377892545 0.7774069

4111.65184504705 0.8450702

4111.66991116865 0.9115441

4111.68797729026 0.848735

4111.70604341186 0.8202542

4111.72410953346 0.8527303

4111.74217565506 0.8766885

4111.76024177666 0.8937123

4111.77830789827 0.8828005

4111.79637401987 0.8303992

4111.81444014147 0.8255108

4111.83250626307 0.8484907

4111.85057238468 0.8469136

4111.86863850628 0.8003084

4111.88670462788 0.8605104

4111.90477074948 0.8684202

4111.92283687108 0.8854179

4111.94090299269 0.8282621

4111.95896911429 0.8317432

4111.97703523589 0.8406554

4111.99510135749 0.7940972

4112.0131674791 0.7724935

4112.0312336007 0.8631188

4112.0492997223 0.9198712

4112.0673658439 0.9557116

4112.0854319655 0.9351507

4112.10349808711 0.8817641

4112.12156420871 0.8805317

4112.13963033031 0.8388647

4112.15769645191 0.8654184

4112.17576257351 0.8778277

4112.19382869512 0.9091259

4112.21189481672 0.9283364

4112.22996093832 0.9086736

4112.24802705992 0.9108877

4112.26609318153 0.9047037

4112.28415930313 0.9332981

4112.30222542473 0.870501

4112.32029154633 0.8753669

4112.33835766793 0.8640438

4112.35642378954 0.9309497

4112.37448991114 0.9086909

4112.39255603274 0.8227765

4112.41062215434 0.8463726

4112.42868827595 0.8941864

4112.44675439755 0.8779755

4112.46482051915 0.9789207

4112.48288664075 0.9427846

4112.50095276235 0.9246788

4112.51901888396 0.8847461

4112.53708500556 0.8486767

4112.55515112716 0.8697867

4112.57321724876 0.8944787

4112.59128337037 0.9422202

4112.60934949197 0.9727228

4112.62741561357 0.8945255

4112.64548173517 0.9170011

4112.66354785677 0.9136889

4112.68161397838 0.9050581

4112.69968009998 0.9198281

4112.71774622158 0.9100995

4112.73581234318 0.9205045

4112.75387846479 0.9769413

4112.77194458639 0.9118623

4112.79001070799 0.9238051

4112.80807682959 0.9196635

4112.82614295119 0.8900722

4112.8442090728 0.9062393

4112.8622751944 0.9241486

4112.880341316 0.9734454

4112.8984074376 1.032032

4112.9164735592 0.9662792

4112.93453968081 0.8928473

4112.95260580241 0.9577226

4112.97067192401 0.9881331

4112.98873804561 0.9393302

4113.00680416722 0.9328443

4113.02487028882 0.9298751

4113.04293641042 0.8989887

4113.06100253202 0.8900912

4113.07906865363 0.9023047

4113.09713477523 0.913595

4113.11520089683 0.8570367

4113.13326701843 0.8707889

4113.15133314003 0.9009721

4113.16939926163 0.8646788

4113.18746538324 0.9183183

4113.20553150484 0.8849149

4113.22359762644 0.8425971

4113.24166374804 0.8521181

4113.25972986965 0.8972839

4113.27779599125 0.8998641

4113.29586211285 0.9214174

4113.31392823445 0.919143

4113.33199435605 0.8888843

4113.35006047766 0.8691066

4113.36812659926 0.9091154

4113.38619272086 0.8830163

4113.40425884246 0.8519251

4113.42232496407 0.9131162

4113.44039108567 0.9130225

4113.45845720727 0.9118056

4113.47652332887 0.8923036

4113.49458945048 0.8459749

4113.51265557208 0.8924689

4113.53072169368 0.8927293

4113.54878781528 0.8689471

4113.56685393688 0.8718735

4113.58492005849 0.8678817

4113.60298618009 0.9170644

4113.62105230169 0.9801482

4113.63911842329 0.9173023

4113.6571845449 0.8803231

4113.6752506665 0.8957668

4113.6933167881 0.853987

4113.7113829097 0.8643202

4113.7294490313 0.8577454

4113.74751515291 0.870261

4113.76558127451 0.8916312

4113.78364739611 0.8735915

4113.80171351771 0.871273

4113.81977963931 0.8874218

4113.83784576092 0.8967431

4113.85591188252 0.8803376

4113.87397800412 0.9184301

4113.89204412572 0.9100077

4113.91011024733 0.9029031

4113.92817636893 0.9455977

4113.94624249053 0.9455491

4113.96430861213 0.9736364

4113.98237473373 1.021485

4114.00044085534 0.9747579

4114.01850697694 0.9020076

4114.03657309854 0.9496505

4114.05463922014 0.9187236

4114.07270534175 0.8468498

4114.09077146335 0.9086147

4114.10883758495 0.8961378

4114.12690370655 0.8838384

4114.14496982815 0.9459336

4114.16303594976 0.9291466

4114.18110207136 0.8932083

4114.19916819296 0.9043765

4114.21723431456 0.8954098

4114.23530043617 0.9294738

4114.25336655777 0.9508419

4114.27143267937 0.9948843

4114.28949880097 0.9796926

4114.30756492257 0.9779767

4114.32563104418 0.9505484

4114.34369716578 0.9459547

4114.36176328738 0.9545715

4114.37982940898 0.9101346

4114.39789553058 0.9326671

4114.41596165219 0.9858065

4114.43402777379 0.9495845

4114.45209389539 0.9542082

4114.47016001699 0.9153268

4114.4882261386 0.9021661

4114.5062922602 0.9442817

4114.5243583818 0.8960534

4114.5424245034 0.9528091

4114.560490625 0.9990106

4114.57855674661 0.9096531

4114.59662286821 0.892011

4114.61468898981 0.9263079

4114.63275511141 0.9502472

4114.65082123302 0.947589

4114.66888735462 0.9444041

4114.68695347622 0.9030071

4114.70501959782 0.9388022

4114.72308571942 0.9607781

4114.74115184103 0.9154087

4114.75921796263 0.8877292

4114.77728408423 0.8977895

4114.79535020583 0.9355227

4114.81341632743 0.9705701

4114.83148244904 0.9214841

4114.84954857064 0.8865313

4114.86761469224 0.8854856

4114.88568081384 0.861984

4114.90374693545 0.9431265

4114.92181305705 0.978875

4114.93987917865 0.9221404

4114.95794530025 0.9320588

4114.97601142185 0.9487902

4114.99407754346 0.9483503

4115.01214366506 0.8776459

4115.03020978666 0.9008704

4115.04827590826 0.9343706

4115.06634202987 0.8735253

4115.08440815147 0.9738488

4115.10247427307 0.942284

4115.12054039467 0.9594856

4115.13860651628 0.9773418

4115.15667263788 0.9331655

4115.17473875948 0.8858804

4115.19280488108 0.8628424

4115.21087100268 0.9036625

4115.22893712429 0.9128521

4115.24700324589 0.8957771

4115.26506936749 0.8993266

4115.28313548909 0.9503773

4115.3012016107 0.9387166

4115.3192677323 0.9293484

4115.3373338539 0.9130357

4115.3553999755 0.9204091

4115.3734660971 0.9069719

4115.39153221871 0.8890952

4115.40959834031 0.9446313

4115.42766446191 0.9276382

4115.44573058351 0.9407395

4115.46379670512 0.9066453

4115.48186282672 0.8629838

4115.49992894832 0.9178065

4115.51799506992 0.9827865

4115.53606119152 0.9814273

4115.55412731313 0.9842046

4115.57219343473 0.9363245

4115.59025955633 0.9301479

4115.60832567793 0.9644664

4115.62639179954 0.9230691

4115.64445792114 0.945511

4115.66252404274 0.9172351

4115.68059016434 0.9617679

4115.69865628594 0.9459511

4115.71672240755 0.9437686

4115.73478852915 0.9334054

4115.75285465075 0.9470361

4115.77092077235 0.9702717

4115.78898689396 0.9568416

4115.80705301556 0.8898852

4115.82511913716 0.8855084

4115.84318525876 0.9287981

4115.86125138036 0.9559656

4115.87931750197 0.958174

4115.89738362357 0.9956537

4115.91544974517 0.989103

4115.93351586677 1.060321

4115.95158198837 1.043316

4115.96964810998 0.9997086

4115.98771423158 1.042041

4116.00578035318 1.120899

4116.02384647478 1.071576

4116.04191259639 0.7645062

4116.05997871799 0.7468565

4116.07804483959 0.7026936

4116.09611096119 0.7382293

4116.11417708279 0.8983767

4116.1322432044 0.9641045

4116.150309326 1.011939

4116.1683754476 1.029572

4116.1864415692 1.071337

4116.20450769081 1.081722

4116.22257381241 0.8766446

4116.24063993401 0.8360208

4116.25870605561 0.7945818

4116.27677217721 0.7587618

4116.29483829882 0.8388011

4116.31290442042 0.8715942

4116.33097054202 0.9184316

4116.34903666362 0.9392362

4116.36710278522 0.9568799

4116.38516890683 0.8874971

4116.40323502843 0.8323483

4116.42130115003 0.7803308

4116.43936727163 0.8897468

4116.45743339324 0.9909248

4116.47549951484 0.8503536

4116.49356563644 0.7806034

4116.51163175804 1.01214

4116.52969787964 0.918197

4116.54776400125 0.8362108

4116.56583012285 1.002043

4116.58389624445 1.053858

4116.60196236605 0.873235

4116.62002848766 0.6744612

4116.63809460926 0.7499939

4116.65616073086 0.7530466

4116.67422685246 0.7339164

4116.69229297406 0.8209859

4116.71035909567 0.7375835

4116.72842521727 0.6637387

4116.74649133887 0.7548737

4116.76455746047 0.8440118

4116.78262358207 0.8901793

4116.80068970368 0.9936794

4116.81875582528 0.8406851

4116.83682194688 0.8556254

4116.85488806848 0.8559858

4116.87295419009 0.8545551

4116.89102031169 0.9097282

4116.90908643329 0.8034596

4116.92715255489 0.7753774

4116.9452186765 0.8385903

4116.9632847981 0.7093058

4116.9813509197 0.5774992

4116.9994170413 0.7738711

4117.0174831629 0.9336878

4117.03554928451 0.9344455

4117.05361540611 0.9191741

4117.07168152771 0.965911

4117.08974764931 1.017285

4117.10781377091 0.8873169

4117.12587989252 0.8771014

4117.14394601412 0.8287082

4117.16201213572 0.6878605

4117.18007825732 0.6774684

4117.19814437893 0.8928264

4117.21621050053 0.8382962

4117.23427662213 0.8118633

4117.25234274373 0.6877496

4117.27040886534 0.6985396

4117.28847498694 0.7352287

4117.30654110854 0.705143

4117.32460723014 0.8822359

4117.34267335174 0.942575

4117.36073947334 0.9021596

4117.37880559495 0.8399438

4117.39687171655 0.8128662

4117.41493783815 0.8272498

4117.43300395975 0.7101893

4117.45107008136 0.6487381

4117.46913620296 0.8159475

4117.48720232456 0.9435103

4117.50526844616 0.9011304

4117.52333456777 0.89733

4117.54140068937 0.9226815

4117.55946681097 0.9256262

4117.57753293257 0.9940611

4117.59559905417 1.018376

4117.61366517578 0.9099871

4117.63173129738 0.8154756

4117.64979741898 1.034416

4117.66786354058 0.9780461

4117.68592966219 0.7962868

4117.70399578379 0.8244305

4117.72206190539 1.005124

4117.74012802699 1.148441

4117.75819414859 1.10623

4117.7762602702 0.9257676

4117.7943263918 0.8795053

4117.8123925134 0.890255

4117.830458635 0.9035532

4117.84852475661 0.9102504

4117.86659087821 0.8125635

4117.88465699981 0.7553571

4117.90272312141 0.7440498

4117.92078924301 0.7830226

4117.93885536462 0.9060189

4117.95692148622 0.8828266

4117.97498760782 0.7338462

4117.99305372942 0.9021599

4118.01111985102 0.9738314

4118.02918597263 0.776549

4118.04725209423 0.7492951

4118.06531821583 0.7569502

4118.08338433743 0.8337015

4118.10145045904 0.8614666

4118.11951658064 0.9600179

4118.13758270224 0.9394854

4118.15564882384 0.8910089

4118.17371494544 0.9427967

4118.19178106705 0.8983967

4118.20984718865 0.8154254

4118.22791331025 0.8578106

4118.24597943185 0.8618439

4118.26404555346 0.8476057

4118.28211167506 0.9113901

4118.30017779666 0.9237703

4118.31824391826 0.9134121

4118.33631003986 0.8166473

4118.35437616147 0.9130398

4118.37244228307 0.9988533

4118.39050840467 0.9274714

4118.40857452627 0.8530781

4118.42664064787 0.7672907

4118.44470676948 0.7489795

4118.46277289108 0.9021615

4118.48083901268 0.9283466

4118.49890513428 0.931379

4118.51697125589 0.9858967

4118.53503737749 0.8932821

4118.55310349909 0.820609

4118.57116962069 0.7263793

4118.58923574229 0.7416625

4118.6073018639 0.7584507

4118.6253679855 0.7848625

4118.6434341071 0.9334232

4118.6615002287 0.9444448

4118.67956635031 0.9362425

4118.69763247191 0.9907071

4118.71569859351 0.9789784

4118.73376471511 0.8975015

4118.75183083671 0.8257199

4118.76989695832 0.9428189

4118.78796307992 0.9662273

4118.80602920152 0.8374824

4118.82409532312 0.8130831

4118.84216144473 0.7451723

4118.86022756633 0.7785376

4118.87829368793 0.8989281

4118.89635980953 0.8282322

4118.91442593113 0.9674674

4118.93249205274 0.934211

4118.95055817434 0.8017503

4118.96862429594 0.8215331

4118.98669041754 0.7892028

4119.00475653914 0.8798677

4119.02282266075 0.915127

4119.04088878235 0.8281196

4119.05895490395 0.8600763

4119.07702102555 0.8464141

4119.09508714716 0.7990692

4119.11315326876 0.7827965

4119.13121939036 0.8564231

4119.14928551196 0.8668597

4119.16735163357 0.747227

4119.18541775517 0.6834818

4119.20348387677 0.8589484

4119.22154999837 0.8474585

4119.23961611997 0.8232625

4119.25768224158 0.7542265

4119.27574836318 0.7464777

4119.29381448478 0.7469857

4119.31188060638 0.7692742

4119.32994672799 0.8270528

4119.34801284959 0.8500437

4119.36607897119 0.7565864

4119.38414509279 0.735338

4119.40221121439 0.76351

4119.420277336 0.7775433

4119.4383434576 0.8102171

4119.4564095792 0.8091711

4119.4744757008 0.8076023

4119.49254182241 0.7904202

4119.51060794401 0.7790374

4119.52867406561 0.7877169

4119.54674018721 0.7254295

4119.56480630881 0.7798647

4119.58287243041 0.8038946

4119.60093855202 0.7185171

4119.61900467362 0.778384

4119.63707079522 0.8314708

4119.65513691682 0.869918

4119.67320303843 0.8735131

4119.69126916003 0.7773457

4119.70933528163 0.8123868

4119.72740140323 0.9309584

4119.74546752483 0.8524275

4119.76353364644 0.8130296

4119.78159976804 0.9374331

4119.79966588964 0.9100975

4119.81773201124 0.8962718

4119.83579813285 0.9379088

4119.85386425445 0.8935277

4119.87193037605 0.8659227

4119.88999649765 0.8310719

4119.90806261926 0.7742413

4119.92612874086 0.7976581

4119.94419486246 0.8230649

4119.96226098406 0.8257928

4119.98032710566 0.7250303

4119.99839322727 0.7317227

4120.01645934887 0.7829224

4120.03452547047 0.8321023

4120.05259159207 0.8546439

4120.07065771368 0.8298158

4120.08872383528 0.8595934

4120.10678995688 0.903232

4120.12485607848 0.8837596

4120.14292220008 0.9440352

4120.16098832169 0.9839641

4120.17905444329 0.9432723

4120.19712056489 0.8734434

4120.21518668649 0.883829

4120.2332528081 0.7712457

4120.2513189297 0.7578628

4120.2693850513 0.8868538

4120.2874511729 0.8423576

4120.3055172945 0.80356

4120.32358341611 0.8863658

4120.34164953771 0.9422079

4120.35971565931 0.9459534

4120.37778178091 0.9853465

4120.39584790252 0.9876771

4120.41391402412 0.9613086

4120.43198014572 0.9791665

4120.45004626732 0.9693722

4120.46811238892 0.8737464

4120.48617851053 0.9310029

4120.50424463213 1.010846

4120.52231075373 1.00055

4120.54037687533 0.9186459

4120.55844299693 0.9000548

4120.57650911854 0.9483677

4120.59457524014 0.9944608

4120.61264136174 1.023909

4120.63070748334 0.953031

4120.64877360495 0.8199865

4120.66683972655 0.7482543

4120.68490584815 0.8136682

4120.70297196975 0.9061348

4120.72103809135 0.8929633

4120.73910421296 0.7785163

4120.75717033456 0.6962116

4120.77523645616 0.7821059

4120.79330257776 0.8598637

4120.81136869937 0.8924156

4120.82943482097 0.9391081

4120.84750094257 0.9114606

4120.86556706417 0.9503025

4120.88363318577 0.8531902

4120.90169930738 0.7397898

4120.91976542898 0.7753654

4120.93783155058 0.9038815

4120.95589767218 0.9166795

4120.97396379379 0.8594139

4120.99202991539 0.8157232

4121.01009603699 0.8088951

4121.02816215859 0.8092344

4121.04622828019 0.8307759

4121.0642944018 0.7731626

4121.0823605234 0.7186637

4121.100426645 0.7948326

4121.1184927666 0.9233109

4121.13655888821 0.9310917

4121.15462500981 0.9590317

4121.17269113141 0.9787416

4121.19075725301 0.8981168

4121.20882337461 0.8900715

4121.22688949622 0.9125653

4121.24495561782 0.9146292

4121.26302173942 0.8471314

4121.28108786102 0.8501229

4121.29915398262 0.9187403

4121.31722010423 0.849838

4121.33528622583 0.8917836

4121.35335234743 0.8870199

4121.37141846903 0.814578

4121.38948459064 0.8184749

4121.40755071224 0.9309329

4121.42561683384 0.9842825

4121.44368295544 0.970713

4121.46174907704 1.008732

4121.47981519865 0.9571495

4121.49788132025 0.8700645

4121.51594744185 0.8049946

4121.53401356345 0.8746629

4121.55207968505 0.9013443

4121.57014580666 0.9479473

4121.58821192826 0.9795954

4121.60627804986 0.8645033

4121.62434417146 0.8160799

4121.64241029307 0.9020359

4121.66047641467 0.9014207

4121.67854253627 0.8941215

4121.69660865787 0.9715078

4121.71467477947 1.00998

4121.73274090108 0.9764431

4121.75080702268 0.9300546

4121.76887314428 0.9150791

4121.78693926588 0.889982

4121.80500538749 0.8495675

4121.82307150909 0.811985

4121.84113763069 0.8796774

4121.85920375229 1.006625

4121.8772698739 0.9144322

4121.8953359955 0.8596466

4121.9134021171 0.936657

4121.9314682387 0.9315217

4121.9495343603 0.9654905

4121.96760048191 0.9302134

4121.98566660351 0.7798383

4122.00373272511 0.8090433

4122.02179884671 0.9001403

4122.03986496832 0.9212708

4122.05793108992 0.8597897

4122.07599721152 0.8441094

4122.09406333312 0.8945365

4122.11212945472 0.883829

4122.13019557633 0.9478966

4122.14826169793 0.9554391

4122.16632781953 0.876191

4122.18439394113 0.7880975

4122.20246006273 0.9525381

4122.22052618434 0.9260224

4122.23859230594 0.7792999

4122.25665842754 0.9003811

4122.27472454914 0.9504449

4122.29279067075 0.8875425

4122.31085679235 0.9128808

4122.32892291395 0.912244

4122.34698903555 0.8095324

4122.36505515715 0.8570583

4122.38312127876 0.8618687

4122.40118740036 0.8411231

4122.41925352196 0.8936867

4122.43731964356 0.9054134

4122.45538576517 0.819031

4122.47345188677 0.74805

4122.49151800837 0.8200333

4122.50958412997 0.8776997

4122.52765025157 0.7990727

4122.54571637318 0.7562431

4122.56378249478 0.761844

4122.58184861638 0.7623724

4122.59991473798 0.7594151

4122.61798085958 0.8458515

4122.63604698119 0.9080222

4122.65411310279 0.8123382

4122.67217922439 0.7705096

4122.69024534599 0.7624575

4122.7083114676 0.7789286

4122.7263775892 0.895723

4122.7444437108 0.9072104

4122.7625098324 0.8132601

4122.780575954 0.7765838

4122.79864207561 0.7725798

4122.81670819721 0.6861957

4122.83477431881 0.7479949

4122.85284044041 0.8199607

4122.87090656202 0.8363178

4122.88897268362 0.7903001

4122.90703880522 0.761251

4122.92510492682 0.8030349

4122.94317104842 0.8220985

4122.96123717003 0.8027375

4122.97930329163 0.8175966

4122.99736941323 0.7843855

4123.01543553483 0.718768

4123.03350165644 0.7770199

4123.05156777804 0.8635333

4123.06963389964 0.8319834

4123.08770002124 0.7310816

4123.10576614284 0.8473946

4123.12383226445 0.8932926

4123.14189838605 0.8855946

4123.15996450765 0.7997949

4123.17803062925 0.8210104

4123.19609675085 0.9160025

4123.21416287246 0.9154234

4123.23222899406 0.9117054

4123.25029511566 0.8876002

4123.26836123726 0.9447703

4123.28642735887 0.9723056

4123.30449348047 0.9472357

4123.32255960207 0.8438344

4123.34062572367 0.8380725

4123.35869184528 0.8259448

4123.37675796688 0.8114909

4123.39482408848 0.8185072

4123.41289021008 0.8192407

4123.43095633168 0.8289764

4123.44902245329 0.800246

4123.46708857489 0.742516

4123.48515469649 0.7314745

4123.50322081809 0.7860378

4123.52128693969 0.9059988

4123.5393530613 0.8609267

4123.5574191829 0.8421321

4123.5754853045 0.8348404

4123.5935514261 0.8043399

4123.61161754771 0.8785363

4123.62968366931 0.8566264

4123.64774979091 0.8859248

4123.66581591251 0.8243576

4123.68388203411 0.8160565

4123.70194815572 0.8464938

4123.72001427732 0.7876306

4123.73808039892 0.8049722

4123.75614652052 0.8461558

4123.77421264212 0.8029649

4123.79227876373 0.7860876

4123.81034488533 0.8300673

4123.82841100693 0.8530478

4123.84647712853 0.8560656

4123.86454325014 0.8001841

4123.88260937174 0.779704

4123.90067549334 0.8825474

4123.91874161494 0.8930496

4123.93680773655 0.8090888

4123.95487385815 0.7759868

4123.97293997975 0.8189561

4123.99100610135 0.8729228

4124.00907222295 0.8564132

4124.02713834456 0.8652824

4124.04520446616 0.8781971

4124.06327058776 0.9182761

4124.08133670936 0.9214097

4124.09940283097 0.913093

4124.11746895257 0.8908775

4124.13553507417 0.8917484

4124.15360119577 0.9314562

4124.17166731737 0.9627463

4124.18973343898 0.9261514

4124.20779956058 0.9033952

4124.22586568218 0.8922722

4124.24393180378 0.9147404

4124.26199792539 0.9608693

4124.28006404699 1.002501

4124.29813016859 0.9912254

4124.31619629019 0.9544186

4124.3342624118 0.9307276

4124.3523285334 0.8516361

4124.370394655 0.8170519

4124.3884607766 0.8544983

4124.4065268982 0.931455

4124.42459301981 1.055451

4124.44265914141 1.001898

4124.46072526301 0.9190294

4124.47879138461 0.8749717

4124.49685750621 0.9092705

4124.51492362782 0.9320828

4124.53298974942 0.9097195

4124.55105587102 0.9351691

4124.56912199262 0.9500461

4124.58718811422 0.8971246

4124.60525423583 0.8336375

4124.62332035743 0.8216744

4124.64138647903 0.8627445

4124.65945260063 0.8940551

4124.67751872224 0.965293

4124.69558484384 0.9252806

4124.71365096544 0.8696399

4124.73171708704 0.8838693

4124.74978320864 0.9134855

4124.76784933025 0.9096539

4124.78591545185 0.8759718

4124.80398157345 0.9107785

4124.82204769505 0.8522203

4124.84011381666 0.8657583

4124.85817993826 0.9419717

4124.87624605986 0.8877243

4124.89431218146 0.7832417

4124.91237830306 0.8150969

4124.93044442467 0.9393537

4124.94851054627 0.9469223

4124.96657666787 0.934608

4124.98464278947 0.8773478

4125.00270891108 0.9272108

4125.02077503268 0.9076288

4125.03884115428 0.8380851

4125.05690727588 0.847086

4125.07497339748 0.9240901

4125.09303951909 0.9068916

4125.11110564069 0.8767006

4125.12917176229 0.9505519

4125.14723788389 0.9683663

4125.1653040055 0.8846993

4125.1833701271 0.8761839

4125.2014362487 0.8908725

4125.2195023703 0.9055619

4125.2375684919 0.9292053

4125.25563461351 0.8975176

4125.27370073511 0.9282167

4125.29176685671 0.962622

4125.30983297831 0.90977

4125.32789909992 0.8910967

4125.34596522152 0.9389941

4125.36403134312 0.895859

4125.38209746472 0.8285601

4125.40016358632 0.8594217

4125.41822970793 0.9797583

4125.43629582953 0.9656385

4125.45436195113 0.9761488

4125.47242807273 0.9843609

4125.49049419433 0.9027879

4125.50856031594 0.9161051

4125.52662643754 0.9327549

4125.54469255914 0.9047691

4125.56275868074 0.8982899

4125.58082480235 0.9748449

4125.59889092395 1.007548

4125.61695704555 1.026326

4125.63502316715 1.008737

4125.65308928875 0.9731181

4125.67115541036 0.9545538

4125.68922153196 0.9445071

4125.70728765356 0.9481381

4125.72535377516 0.8885689

4125.74341989677 0.8376273

4125.76148601837 0.9066886

4125.77955213997 0.9377013

4125.79761826157 0.929615

4125.81568438317 0.9684719

4125.83375050478 0.9724404

4125.85181662638 0.9694979

4125.86988274798 0.9276218

4125.88794886958 0.9339339

4125.90601499118 0.9464819

4125.92408111279 0.8951249

4125.94214723439 0.9384265

4125.96021335599 0.9055213

4125.97827947759 0.853093

4125.9963455992 0.9164636

4126.0144117208 0.9935663

4126.0324778424 1.014966

4126.050543964 0.9404491

4126.06861008561 0.9113965

4126.08667620721 0.8732424

4126.10474232881 0.8627632

4126.12280845041 0.9189301

4126.14087457201 0.9299328

4126.15894069362 0.9520525

4126.17700681522 0.9995

4126.19507293682 0.9615709

4126.21313905842 0.9162861

4126.23120518003 0.8886483

4126.24927130163 0.9032117

4126.26733742323 0.9396946

4126.28540354483 0.9352236

4126.30346966643 0.9074134

4126.32153578804 0.9406614

4126.33960190964 0.9594341

4126.35766803124 0.919492

4126.37573415284 0.9201322

4126.39380027444 0.9155911

4126.41186639605 0.9350145

4126.42993251765 0.9017062

4126.44799863925 0.8865529

4126.46606476085 0.9414229

4126.48413088246 0.9744153

4126.50219700406 0.9008768

4126.52026312566 0.8892086

4126.53832924726 0.9398136

4126.55639536886 0.9448197

4126.57446149047 0.9494898

4126.59252761207 0.9604785

4126.61059373367 0.9503521

4126.62865985527 0.8849396

4126.64672597688 0.8707963

4126.66479209848 0.9079463

4126.68285822008 0.8938061

4126.70092434168 0.8632139

4126.71899046328 0.8562522

4126.73705658488 0.8571669

4126.75512270649 0.8688695

4126.77318882809 0.9337147

4126.79125494969 0.9891862

4126.80932107129 0.9790902

4126.8273871929 0.9862212

4126.8454533145 1.003434

4126.8635194361 0.9582546

4126.8815855577 0.8994412

4126.89965167931 0.9129933

4126.91771780091 0.9153835

4126.93578392251 0.8773012

4126.95385004411 0.8638823

4126.97191616571 0.8947865

4126.98998228732 0.9405051

4127.00804840892 0.9189643

4127.02611453052 0.8952737

4127.04418065212 0.9369212

4127.06224677373 0.9107974

4127.08031289533 0.9497372

4127.09837901693 0.9794915

4127.11644513853 0.9685043

4127.13451126013 0.9314206

4127.15257738174 0.8589582

4127.17064350334 0.8860093

4127.18870962494 0.9335406

4127.20677574654 0.9162676

4127.22484186815 0.8953981

4127.24290798975 0.8632357

4127.26097411135 0.9034739

4127.27904023295 0.9389591

4127.29710635456 0.883693

4127.31517247616 0.9049062

4127.33323859776 0.9360025

4127.35130471936 0.9049453

4127.36937084096 0.8892825

4127.38743696257 0.8393306

4127.40550308417 0.8305871

4127.42356920577 0.8734086

4127.44163532737 0.891716

4127.45970144897 0.8439183

4127.47776757058 0.8373855

4127.49583369218 0.9006879

4127.51389981378 0.9268578

4127.53196593538 0.9033339

4127.55003205699 0.8885609

4127.56809817859 0.8645651

4127.58616430019 0.8373264

4127.60423042179 0.8284999

4127.62229654339 0.8088931

4127.640362665 0.8300344

4127.6584287866 0.8344582

4127.6764949082 0.8439934

4127.6945610298 0.8240059

4127.71262715141 0.8197981

4127.73069327301 0.8170946

4127.74875939461 0.8151274

4127.76682551621 0.8317398

4127.78489163781 0.7952651

4127.80295775942 0.7943047

4127.82102388102 0.8455679

4127.83909000262 0.8232861

4127.85715612422 0.7631909

4127.87522224582 0.7912117

4127.89328836743 0.7952794

4127.91135448903 0.7154113

4127.92942061063 0.6965793

4127.94748673223 0.7242135

4127.96555285383 0.7238851

4127.98361897544 0.6878014

4128.00168509704 0.6523386

4128.01975121864 0.6749227

4128.03781734024 0.6518157

4128.05588346185 0.63547

4128.07394958345 0.655232

4128.09201570505 0.6032153

4128.11008182665 0.6180118

4128.12814794826 0.6201899

4128.14621406986 0.5718583

4128.16428019146 0.5199963

4128.18234631306 0.5384752

4128.20041243466 0.5254309

4128.21847855627 0.5398505

4128.23654467787 0.5471606

4128.25461079947 0.5547762

4128.27267692107 0.5816206

4128.29074304267 0.5204173

4128.30880916428 0.5673128

4128.32687528588 0.5957799

4128.34494140748 0.5729713

4128.36300752908 0.5943435

4128.38107365069 0.6192477

4128.39913977229 0.584989

4128.41720589389 0.57532

4128.43527201549 0.6493605

4128.4533381371 0.6640013

4128.4714042587 0.6598178

4128.4894703803 0.6758609

4128.5075365019 0.6803997

4128.5256026235 0.709843

4128.5436687451 0.6964428

4128.56173486671 0.7274499

4128.57980098831 0.7407162

4128.59786710991 0.7569265

4128.61593323151 0.7526135

4128.63399935312 0.7513683

4128.65206547472 0.7249871

4128.67013159632 0.7601106

4128.68819771792 0.8117885

4128.70626383953 0.8039668

4128.72432996113 0.8535939

4128.74239608273 0.8461214

4128.76046220433 0.8465503

4128.77852832593 0.8942162

4128.79659444754 0.8849286

4128.81466056914 0.8776402

4128.83272669074 0.8597016

4128.85079281234 0.8503811

4128.86885893395 0.8340669

4128.88692505555 0.8566308

4128.90499117715 0.8835794

4128.92305729875 0.9166069

4128.94112342035 0.9197479

4128.95918954196 0.875119

4128.97725566356 0.8545519

4128.99532178516 0.8460315

4129.01338790676 0.863084

4129.03145402837 0.8852783

4129.04952014997 0.8775544

4129.06758627157 0.8331783

4129.08565239317 0.875415

4129.10371851477 0.8636603

4129.12178463638 0.8619097

4129.13985075798 0.8571719

4129.15791687958 0.8441041

4129.17598300118 0.8726673

4129.19404912279 0.9685073

4129.21211524439 0.9754144

4129.23018136599 0.9286062

4129.24824748759 0.9050924

4129.2663136092 0.954071

4129.2843797308 0.9269632

4129.3024458524 0.9495467

4129.320511974 0.8712562

4129.3385780956 0.8866726

4129.35664421721 0.9200149

4129.37471033881 0.9159397

4129.39277646041 0.878883

4129.41084258201 0.8640348

4129.42890870361 0.8716389

4129.44697482522 0.8609426

4129.46504094682 0.8606421

4129.48310706842 0.8814515

4129.50117319002 0.8722259

4129.51923931163 0.9011236

4129.53730543323 0.9161537

4129.55537155483 0.8702381

4129.57343767643 0.8504529

4129.59150379803 0.8755463

4129.60956991964 0.8704791

4129.62763604124 0.8606205

4129.64570216284 0.8697022

4129.66376828444 0.8861535

4129.68183440604 0.8767753

4129.69990052765 0.8879108

4129.71796664925 0.892608

4129.73603277085 0.8372531

4129.75409889245 0.8354664

4129.77216501406 0.8682411

4129.79023113566 0.7718539

4129.80829725726 0.7779067

4129.82636337886 0.7831386

4129.84442950046 0.768909

4129.86249562207 0.8012436

4129.88056174367 0.783764

4129.89862786527 0.7905661

4129.91669398687 0.8452116

4129.93476010847 0.8279736

4129.95282623008 0.781422

4129.97089235168 0.8039804

4129.98895847328 0.8432187

4130.00702459488 0.8179059

4130.02509071649 0.8505454

4130.04315683809 0.829802

4130.06122295969 0.8074514

4130.07928908129 0.8740726

4130.0973552029 0.8929218

4130.1154213245 0.8512332

4130.1334874461 0.8298815

4130.1515535677 0.7746183

4130.1696196893 0.7669879

4130.18768581091 0.8204901

4130.20575193251 0.8088977

4130.22381805411 0.8030686

4130.24188417571 0.8218985

4130.25995029732 0.804134

4130.27801641892 0.8373647

4130.29608254052 0.8428705

4130.31414866212 0.8395823

4130.33221478372 0.8871557

4130.35028090533 0.8411583

4130.36834702693 0.8279922

4130.38641314853 0.8975126

4130.40447927013 0.8546037

4130.42254539173 0.8470098

4130.44061151334 0.9141346

4130.45867763494 0.8570609

4130.47674375654 0.8646007

4130.49480987814 0.8364341

4130.51287599975 0.8195133

4130.53094212135 0.870725

4130.54900824295 0.8658649

4130.56707436455 0.8369408

4130.58514048615 0.8071513

4130.60320660776 0.7871923

4130.62127272936 0.7818671

4130.63933885096 0.7983534

4130.65740497256 0.8138535

4130.67547109417 0.8114432

4130.69353721577 0.8073755

4130.71160333737 0.7572955

4130.72966945897 0.6998155

4130.74773558057 0.733941

4130.76580170218 0.7543073

4130.78386782378 0.706

4130.80193394538 0.6400223

4130.82000006698 0.6512616

4130.83806618859 0.6485953

4130.85613231019 0.6726004

4130.87419843179 0.677265

4130.89226455339 0.6334779

4130.91033067499 0.6075116

4130.92839679659 0.5752581

4130.9464629182 0.6029074

4130.9645290398 0.5960416

4130.9825951614 0.5783155

4131.000661283 0.5877513

4131.01872740461 0.5761737

4131.03679352621 0.5639353

4131.05485964781 0.585001

4131.07292576941 0.5676539

4131.09099189102 0.5649314

4131.10905801262 0.6003853

4131.12712413422 0.5952441

4131.14519025582 0.6012222

4131.16325637742 0.6014248

4131.18132249903 0.6162541

4131.19938862063 0.5884566

4131.21745474223 0.5489162

4131.23552086383 0.5578496

4131.25358698544 0.6061792

4131.27165310704 0.6170592

4131.28971922864 0.5937372

4131.30778535024 0.5942338

4131.32585147184 0.6329747

4131.34391759345 0.6449202

4131.36198371505 0.6524557

4131.38004983665 0.704629

4131.39811595825 0.7022463

4131.41618207986 0.6789805

4131.43424820146 0.7199953

4131.45231432306 0.7444715

4131.47038044466 0.7673469

4131.48844656626 0.7846341

4131.50651268787 0.7958325

4131.52457880947 0.781889

4131.54264493107 0.7950321

4131.56071105267 0.8247639

4131.57877717427 0.8083223

4131.59684329588 0.8113257

4131.61490941748 0.8887528

4131.63297553908 0.9137677

4131.65104166068 0.9327196

4131.66910778229 0.901203

4131.68717390389 0.8746463

4131.70524002549 0.9124312

4131.72330614709 0.8853812

4131.74137226869 0.8761362

4131.7594383903 0.9114294

4131.7775045119 0.9266613

4131.7955706335 0.95162

4131.8136367551 0.9284145

4131.83170287671 0.9266129

4131.84976899831 0.9603869

4131.86783511991 0.9411521

4131.88590124151 0.8938004

4131.90396736312 0.8800781

4131.92203348472 0.8785017

4131.94009960632 0.9207478

4131.95816572792 0.9165606

4131.97623184952 0.9275677

4131.99429797113 0.9229929

4132.01236409273 0.9555694

4132.03043021433 0.9952044

4132.04849633593 0.9145131

4132.06656245754 0.8322667

4132.08462857914 0.9073813

4132.10269470074 0.9305613

4132.12076082234 0.9322276

4132.13882694394 0.9394145

4132.15689306555 0.8982562

4132.17495918715 0.8983077

4132.19302530875 0.8687004

4132.21109143035 0.8854504

4132.22915755195 0.9047737

4132.24722367356 0.8730657

4132.26528979516 0.8500215

4132.28335591676 0.870207

4132.30142203836 0.8384192

4132.31948815997 0.8701369

4132.33755428157 0.919039

4132.35562040317 0.917843

4132.37368652477 0.9291815

4132.39175264637 0.9052316

4132.40981876798 0.8538036

4132.42788488958 0.8331963

4132.44595101118 0.8097194

4132.46401713278 0.8289733

4132.48208325439 0.8641744

4132.50014937599 0.8725642

4132.51821549759 0.8639628

4132.53628161919 0.8412716

4132.55434774079 0.8183677

4132.5724138624 0.8147799

4132.590479984 0.8269299

4132.6085461056 0.8022488

4132.6266122272 0.8107796

4132.64467834881 0.8131635

4132.66274447041 0.8154758

4132.68081059201 0.835741

4132.69887671361 0.8303486

4132.71694283521 0.7830654

4132.73500895682 0.8291633

4132.75307507842 0.8405945

4132.77114120002 0.8246811

4132.78920732162 0.8393773

4132.80727344322 0.8433577

4132.82533956483 0.8097681

4132.84340568643 0.7989398

4132.86147180803 0.8302819

4132.87953792963 0.845024

4132.89760405124 0.8086751

4132.91567017284 0.8425258

4132.93373629444 0.8718388

4132.95180241604 0.8610907

4132.96986853764 0.8199471

4132.98793465925 0.8443332

4133.00600078085 0.9386762

4133.02406690245 0.9220523

4133.04213302405 0.8437058

4133.06019914566 0.8940296

4133.07826526726 0.9047728

4133.09633138886 0.875097

4133.11439751046 0.8824111

4133.13246363206 0.9122542

4133.15052975367 0.9738562

4133.16859587527 0.9476112

4133.18666199687 0.9015182

4133.20472811847 0.9272677

4133.22279424007 0.9416286

4133.24086036168 0.8971174

4133.25892648328 0.9361269

4133.27699260488 0.9840317

4133.29505872648 0.9885284

4133.31312484808 0.9722869

4133.33119096969 0.984657

4133.34925709129 0.979153

4133.36732321289 0.9194535

4133.3853893345 0.8739758

4133.4034554561 0.9104038

4133.4215215777 0.9323316

4133.4395876993 0.8958662

4133.4576538209 0.9421022

4133.47571994251 0.9847215

4133.49378606411 0.9728065

4133.51185218571 0.9395729

4133.52991830731 0.8956348

4133.54798442891 0.9307717

4133.56605055052 0.9752851

4133.58411667212 0.9231192

4133.60218279372 0.8913862

4133.62024891532 0.884281

4133.63831503693 0.9191521

4133.65638115853 0.9111204

4133.67444728013 0.8911297

4133.69251340173 0.8719566

4133.71057952333 0.8921236

4133.72864564494 0.9033184

4133.74671176654 0.892201

4133.76477788814 0.8701329

4133.78284400974 0.9062535

4133.80091013135 0.9346009

4133.81897625295 0.9213556

4133.83704237455 0.9072177

4133.85510849615 0.8537601

4133.87317461775 0.8683923

4133.89124073936 0.885502

4133.90930686096 0.8927314

4133.92737298256 0.9506814

4133.94543910416 0.9298629

4133.96350522577 0.8953781

4133.98157134737 0.9798059

4133.99963746897 0.9694481

4134.01770359057 0.8947775

4134.03576971218 0.8657117

4134.05383583378 0.9054537

4134.07190195538 0.9259258

4134.08996807698 0.95577

4134.10803419858 0.9818106

4134.12610032018 0.9660046

4134.14416644179 0.9586591

4134.16223256339 0.9432264

4134.18029868499 0.9422203

4134.19836480659 0.9362578

4134.2164309282 0.9338291

4134.2344970498 0.9431797

4134.2525631714 0.9348422

4134.270629293 0.9417467

4134.28869541461 0.931017

4134.30676153621 0.9665468

4134.32482765781 0.9484338

4134.34289377941 0.9553591

4134.36095990101 0.9821064

4134.37902602262 0.9957949

4134.39709214422 0.9790471

4134.41515826582 0.9753615

4134.43322438742 1.002705

4134.45129050903 1.016855

4134.46935663063 1.031025

4134.48742275223 0.9869181

4134.50548887383 0.9297273

4134.52355499543 0.9408998

4134.54162111704 0.9814932

4134.55968723864 0.9831063

4134.57775336024 0.9405029

4134.59581948184 0.9231366

4134.61388560345 0.9690496

4134.63195172505 1.02502

4134.65001784665 0.9952456

4134.66808396825 0.9710869

4134.68615008985 0.9844412

4134.70421621146 0.9948039

4134.72228233306 0.9968584

4134.74034845466 1.001335

4134.75841457626 1.001373

4134.77648069786 0.928695

4134.79454681947 0.9177154

4134.81261294107 0.9654793

4134.83067906267 0.9613823

4134.84874518427 0.9175537

4134.86681130588 0.9520234

4134.88487742748 0.9345235

4134.90294354908 0.9620558

4134.92100967068 0.9758502

4134.93907579228 0.9264207

4134.95714191389 0.9156926

4134.97520803549 0.9521703

4134.99327415709 0.9420748

4135.01134027869 0.9397919

4135.0294064003 0.9432304

4135.0474725219 0.9536091

4135.0655386435 0.8889939

4135.0836047651 0.9316537

4135.1016708867 0.9117285

4135.11973700831 0.9007049

4135.13780312991 0.9279174

4135.15586925151 0.9945908

4135.17393537311 0.9481684

4135.19200149471 0.9146377

4135.21006761632 0.9321967

4135.22813373792 0.8665469

4135.24619985952 0.8328592

4135.26426598112 0.8438767

4135.28233210273 0.853829

4135.30039822433 0.862864

4135.31846434593 0.8667221

4135.33653046753 0.8724234

4135.35459658913 0.8903729

4135.37266271074 0.8493314

4135.39072883234 0.8502775

4135.40879495394 0.8511059

4135.42686107554 0.8892204

4135.44492719715 0.9075671

4135.46299331875 0.8990247

4135.48105944035 0.9240905

4135.49912556195 0.9055084

4135.51719168355 0.8852233

4135.53525780516 0.8870556

4135.55332392676 0.9095392

4135.57139004836 0.885334

4135.58945616996 0.9024295

4135.60752229157 0.9468793

4135.62558841317 0.9065688

4135.64365453477 0.9193206

4135.66172065637 0.9178734

4135.67978677798 0.9350631

4135.69785289958 0.9582614

4135.71591902118 0.9768718

4135.73398514278 0.9471703

4135.75205126438 0.9125449

4135.77011738598 0.9271886

4135.78818350759 0.9603837

4135.80624962919 0.9654069

4135.82431575079 0.9348876

4135.84238187239 0.9767839

4135.860447994 1.012922

4135.8785141156 0.9722119

4135.8965802372 0.9725891

4135.9146463588 0.9858309

4135.9327124804 0.9592581

4135.95077860201 0.9466459

4135.96884472361 0.9533081

4135.98691084521 0.952593

4136.00497696681 0.9727086

4136.02304308842 1.018979

4136.04110921002 1.021742

4136.05917533162 0.935064

4136.07724145322 0.889144

4136.09530757483 0.9696318

4136.11337369643 0.9724865

4136.13143981803 0.932507

4136.14950593963 0.9345777

4136.16757206123 0.9455922

4136.18563818284 0.9374076

4136.20370430444 0.9614012

4136.22177042604 0.9905252

4136.23983654764 0.9769446

4136.25790266925 0.9272989

4136.27596879085 0.9036052

4136.29403491245 0.9191945

4136.31210103405 0.929388

4136.33016715565 0.9633843

4136.34823327725 0.9277278

4136.36629939886 0.8873739

4136.38436552046 0.8730302

4136.40243164206 0.8958663

4136.42049776366 0.8807857

4136.43856388527 0.9168079

4136.45663000687 0.9164636

4136.47469612847 0.9312464

4136.49276225007 0.9365392

4136.51082837168 0.9100901

4136.52889449328 0.8676383

4136.54696061488 0.8732436

4136.56502673648 0.9017003

4136.58309285809 0.9261265

4136.60115897969 0.8895834

4136.61922510129 0.8871835

4136.63729122289 0.9155741

4136.65535734449 0.8961713

4136.6734234661 0.9457191

4136.6914895877 0.8986886

4136.7095557093 0.8925586

4136.7276218309 0.9479454

4136.7456879525 0.91053

4136.76375407411 0.8445953

4136.78182019571 0.8131341

4136.79988631731 0.8434516

4136.81795243891 0.8969269

4136.83601856051 0.898781

4136.85408468212 0.8684651

4136.87215080372 0.9074572

4136.89021692532 0.9079874

4136.90828304692 0.9383847

4136.92634916853 0.9275528

4136.94441529013 0.8768793

4136.96248141173 0.8482725

4136.98054753333 0.8349469

4136.99861365494 0.8415043

4137.01667977654 0.8931559

4137.03474589814 0.9237277

4137.05281201974 0.9102006

4137.07087814134 0.8901808

4137.08894426295 0.9013108

4137.10701038455 0.8698048

4137.12507650615 0.8603057

4137.14314262775 0.8525633

4137.16120874935 0.8571063

4137.17927487096 0.8262844

4137.19734099256 0.8422723

4137.21540711416 0.896494

4137.23347323576 0.8952814

4137.25153935737 0.9403648

4137.26960547897 0.9014287

4137.28767160057 0.7993541

4137.30573772217 0.8604366

4137.32380384377 0.9171981

4137.34186996538 0.8823752

4137.35993608698 0.8651295

4137.37800220858 0.853277

4137.39606833018 0.8807821

4137.41413445179 0.9032693

4137.43220057339 0.9136747

4137.45026669499 0.9262134

4137.46833281659 0.8997387

4137.48639893819 0.8560579

4137.5044650598 0.8699443

4137.5225311814 0.9233712

4137.540597303 0.9253137

4137.5586634246 0.876226

4137.57672954621 0.8643473

4137.59479566781 0.880372

4137.61286178941 0.9025032

4137.63092791101 0.9021769

4137.64899403261 0.8737427

4137.66706015422 0.8773234

4137.68512627582 0.9007605

4137.70319239742 0.9235774

4137.72125851902 0.9046397

4137.73932464062 0.8673937

4137.75739076223 0.9225802

4137.77545688383 0.9346346

4137.79352300543 0.9145471

4137.81158912703 0.9056985

4137.82965524864 0.881677

4137.84772137024 0.8764856

4137.86578749184 0.8988855

4137.88385361344 0.9005211

4137.90191973505 0.8725437

4137.91998585665 0.8728013

4137.93805197825 0.9167098

4137.95611809985 0.9602886

4137.97418422145 0.8745597

4137.99225034306 0.8765441

4138.01031646466 0.8946898

4138.02838258626 0.8714706

4138.04644870786 0.8776067

4138.06451482946 0.8913977

4138.08258095107 0.9032594

4138.10064707267 0.8959228

4138.11871319427 0.8336864

4138.13677931587 0.841482

4138.15484543748 0.8821659

4138.17291155908 0.889257

4138.19097768068 0.8881499

4138.20904380228 0.8520707

4138.22710992388 0.8613376

4138.24517604549 0.8851711

4138.26324216709 0.8778874

4138.28130828869 0.8352556

4138.29937441029 0.8642869

4138.31744053189 0.9024544

4138.3355066535 0.8643484

4138.3535727751 0.8767809

4138.3716388967 0.879613

4138.3897050183 0.8807951

4138.40777113991 0.9011396

4138.42583726151 0.8878452

4138.44390338311 0.8676499

4138.46196950471 0.8458433

4138.48003562631 0.8884056

4138.49810174792 0.8851393

4138.51616786952 0.8626795

4138.53423399112 0.8914598

4138.55230011272 0.9144396

4138.57036623433 0.9161558

4138.58843235593 0.8860695

4138.60649847753 0.8862365

4138.62456459913 0.876834

4138.64263072073 0.8785318

4138.66069684234 0.8344685

4138.67876296394 0.8499311

4138.69682908554 0.8881071

4138.71489520714 0.8951412

4138.73296132875 0.8684929

4138.75102745035 0.8618795

4138.76909357195 0.8794119

4138.78715969355 0.8729192

4138.80522581515 0.8506021

4138.82329193676 0.8714038

4138.84135805836 0.8773854

4138.85942417996 0.8943319

4138.87749030156 0.8861503

4138.89555642317 0.8012819

4138.91362254477 0.8436989

4138.93168866637 0.908944

4138.94975478797 0.8740466

4138.96782090957 0.9332976

4138.98588703118 0.9287994

4139.00395315278 0.8987268

4139.02201927438 0.9569952

4139.04008539598 0.9560508

4139.05815151759 0.8930214

4139.07621763919 0.8540312

4139.09428376079 0.8808036

4139.11234988239 0.9331545

4139.13041600399 0.9138383

4139.1484821256 0.8992432

4139.1665482472 0.9102812

4139.1846143688 0.9363475

4139.2026804904 0.8948531

4139.22074661201 0.8685098

4139.23881273361 0.8744609

4139.25687885521 0.8979993

4139.27494497681 0.900236

4139.29301109841 0.9008014

4139.31107722002 0.8581482

4139.32914334162 0.8437388

4139.34720946322 0.9184657

4139.36527558482 0.9202378

4139.38334170642 0.8997785

4139.40140782803 0.9233886

4139.41947394963 0.9663304

4139.43754007123 0.9473679

4139.45560619283 0.9221239

4139.47367231444 0.9046284

4139.49173843604 0.9058756

4139.50980455764 0.9254582

4139.52787067924 0.9210539

4139.54593680084 0.8610954

4139.56400292245 0.8888718

4139.58206904405 0.8678573

4139.60013516565 0.9135967

4139.61820128725 0.966622

4139.63626740886 0.943463

4139.65433353046 0.914407

4139.67239965206 0.9200692

4139.69046577366 0.9339088

4139.70853189526 0.903755

4139.72659801687 0.9065669

4139.74466413847 0.946301

4139.76273026007 0.9681686

4139.78079638167 0.9403465

4139.79886250328 0.920108

4139.81692862488 0.9237545

4139.83499474648 0.94763

4139.85306086808 0.9515991

4139.87112698968 0.9129413

4139.88919311129 0.9370809

4139.90725923289 0.9781514

4139.92532535449 0.9450382

4139.94339147609 0.9108883

4139.96145759769 0.8977643

4139.9795237193 0.8888128

4139.9975898409 0.9156156

4140.0156559625 0.9590634

4140.0337220841 0.9411055

4140.05178820571 0.9210874

4140.06985432731 0.9473477

4140.08792044891 0.8866352

4140.10598657051 0.8550794

4140.12405269211 0.9104832

4140.14211881372 0.932417

4140.16018493532 0.8856528

4140.17825105692 0.8827864

4140.19631717852 0.9264125

4140.21438330013 0.8943149

4140.23244942173 0.8692824

4140.25051554333 0.8929569

4140.26858166493 0.9141313

4140.28664778653 0.9491365

4140.30471390814 0.9513392

4140.32278002974 0.9264033

4140.34084615134 0.8908105

4140.35891227294 0.8876549

4140.37697839455 0.8955386

4140.39504451615 0.8954242

4140.41311063775 0.9231089

4140.43117675935 0.9307038

4140.44924288096 0.9335939

4140.46730900256 0.9462433

4140.48537512416 0.9727007

4140.50344124576 0.9886767

4140.52150736736 0.9628217

4140.53957348896 0.9134258

4140.55763961057 0.9261209

4140.57570573217 0.8921323

4140.59377185377 0.8473347

4140.61183797537 0.8752249

4140.62990409698 0.8659192

4140.64797021858 0.8490704

4140.66603634018 0.8816243

4140.68410246178 0.8831741

4140.70216858339 0.8956108

4140.72023470499 0.9438791

4140.73830082659 0.9288036

4140.75636694819 0.928252

4140.77443306979 0.9289814

4140.7924991914 0.9277844

4140.810565313 0.8758169

4140.8286314346 0.8872411

4140.8466975562 0.8908411

4140.86476367781 0.8797861

4140.88282979941 0.8263724

4140.90089592101 0.8667225

4140.91896204261 0.9101269

4140.93702816421 0.8747855

4140.95509428582 0.8582669

4140.97316040742 0.8363966

4140.99122652902 0.8483655

4141.00929265062 0.8374146

4141.02735877222 0.8282799

4141.04542489383 0.8665929

4141.06349101543 0.8866482

4141.08155713703 0.8917004

4141.09962325863 0.8869901

4141.11768938024 0.8711079

4141.13575550184 0.8513031

4141.15382162344 0.8750083

4141.17188774504 0.8394823

4141.18995386665 0.8904042

4141.20801998825 0.8925686

4141.22608610985 0.889704

4141.24415223145 0.8257894

4141.26221835305 0.8566946

4141.28028447466 0.9329264

4141.29835059626 0.8992804

4141.31641671786 0.8735363

4141.33448283946 0.8654503

4141.35254896106 0.8749264

4141.37061508267 0.8520832

4141.38868120427 0.8435717

4141.40674732587 0.8427736

4141.42481344747 0.8475012

4141.44287956908 0.8531389

4141.46094569068 0.89781

4141.47901181228 0.9140284

4141.49707793388 0.8981708

4141.51514405548 0.8586106

4141.53321017709 0.8901548

4141.55127629869 0.8889993

4141.56934242029 0.8057041

4141.58740854189 0.8034089

4141.6054746635 0.8016366

4141.6235407851 0.8145966

4141.6416069067 0.8640547

4141.6596730283 0.8797194

4141.6777391499 0.8612443

4141.69580527151 0.8786602

4141.71387139311 0.8640355

4141.73193751471 0.8905787

4141.75000363631 0.9693226

4141.76806975792 0.9151108

4141.78613587952 0.8499192

4141.80420200112 0.916835

4141.82226812272 0.9014928

4141.84033424432 0.8930733

4141.85840036593 0.8715689

4141.87646648753 0.8882728

4141.89453260913 0.9250691

4141.91259873073 0.9661615

4141.93066485234 0.9543769

4141.94873097394 0.8945561

4141.96679709554 0.8641224

4141.98486321714 0.8421581

4142.00292933874 0.9017807

4142.02099546035 0.965064

4142.03906158195 0.9226676

4142.05712770355 0.9094068

4142.07519382515 0.8628278

4142.09325994675 0.9286147

4142.11132606836 0.9083534

4142.12939218996 0.9354103

4142.14745831156 0.9223801

4142.16552443316 0.862221

4142.18359055477 0.8725454

4142.20165667637 0.9468608

4142.21972279797 0.9356837

4142.23778891957 0.8970279

4142.25585504117 0.9130259

4142.27392116278 0.9381466

4142.29198728438 0.8900672

4142.31005340598 0.8566857

4142.32811952758 0.8771629

4142.34618564919 0.9091287

4142.36425177079 0.8591442

4142.38231789239 0.8631846

4142.40038401399 0.8536197

4142.41845013559 0.8623036

4142.4365162572 0.8549708

4142.4545823788 0.8994831

4142.4726485004 0.9558175

4142.490714622 0.9489852

4142.5087807436 0.9538584

4142.52684686521 0.9181554

4142.54491298681 0.8987717

4142.56297910841 0.950019

4142.58104523001 0.8983057

4142.59911135162 0.8561419

4142.61717747322 0.8636019

4142.63524359482 0.904595

4142.65330971642 0.9277122

4142.67137583802 0.8916762

4142.68944195963 0.8796624

4142.70750808123 0.9198952

4142.72557420283 0.8976147

4142.74364032443 0.9115607

4142.76170644604 0.9123001

4142.77977256764 0.8918166

4142.79783868924 0.8885647

4142.81590481084 0.8412784

4142.83397093245 0.8813172

4142.85203705405 0.8679081

4142.87010317565 0.864291

4142.88816929725 0.8902272

4142.90623541885 0.8383553

4142.92430154046 0.8259198

4142.94236766206 0.8649026

4142.96043378366 0.9122422

4142.97849990526 0.9097377

4142.99656602686 0.8663623

4143.01463214847 0.829881

4143.03269827007 0.8539881

4143.05076439167 0.8666744

4143.06883051327 0.8586606

4143.08689663488 0.82075

4143.10496275648 0.8419406

4143.12302887808 0.9077343

4143.14109499968 0.870174

4143.15916112128 0.8233875

4143.17722724289 0.8365058

4143.19529336449 0.8092197

4143.21335948609 0.8276604

4143.23142560769 0.8479815

4143.24949172929 0.8884563

4143.2675578509 0.8324884

4143.2856239725 0.8042918

4143.3036900941 0.8497518

4143.3217562157 0.8539376

4143.33982233731 0.8683038

4143.35788845891 0.8536512

4143.37595458051 0.8455495

4143.39402070211 0.9390274

4143.41208682372 0.8545964

4143.43015294532 0.7667667

4143.44821906692 0.8450872

4143.46628518852 0.9159982

4143.48435131012 0.8549632

4143.50241743173 0.9079033

4143.52048355333 0.8634837

4143.53854967493 0.8836337

4143.55661579653 0.9057752

4143.57468191813 0.8637894

4143.59274803974 0.8473133

4143.61081416134 0.9204034

4143.62888028294 0.9530716

4143.64694640454 0.8674184

4143.66501252615 0.8936852

4143.68307864775 0.9307683

4143.70114476935 0.9258071

4143.71921089095 0.9308195

4143.73727701255 0.8772497

4143.75534313416 0.84337

4143.77340925576 0.8799461

4143.79147537736 0.8804026

4143.80954149896 0.8586257

4143.82760762057 0.8320513

4143.84567374217 0.8188812

4143.86373986377 0.8384066

4143.88180598537 0.8615489

4143.89987210698 0.8813308

4143.91793822858 0.8820333

4143.93600435018 0.8502614

4143.95407047178 0.8810595

4143.97213659338 0.8696433

4143.99020271499 0.8735815

4144.00826883659 0.9359037

4144.02633495819 0.9415858

4144.04440107979 0.927415

4144.06246720139 0.8715434

4144.080533323 0.8988948

4144.0985994446 0.9166283

4144.1166655662 0.8286343

4144.1347316878 0.88254

4144.1527978094 0.8959914

4144.17086393101 0.8498331

4144.18893005261 0.8439249

4144.20699617421 0.8267962

4144.22506229581 0.8283083

4144.24312841742 0.8836795

4144.26119453902 0.9869026

4144.27926066062 0.9419966

4144.29732678222 0.8488368

4144.31539290383 0.8293543

4144.33345902543 0.782281

4144.35152514703 0.8335663

4144.36959126863 0.971146

4144.38765739023 0.9869721

4144.40572351184 0.9611991

4144.42378963344 0.8940092

4144.44185575504 0.8891034

4144.45992187664 0.9152418

4144.47798799825 0.8947887

4144.49605411985 0.8782703

4144.51412024145 0.9112288

4144.53218636305 0.8888841

4144.55025248465 0.8576544

4144.56831860626 0.9076051

4144.58638472786 0.9235373

4144.60445084946 0.8876692

4144.62251697106 0.875495

4144.64058309267 0.9134074

4144.65864921427 0.9018946

4144.67671533587 0.8929293

4144.69478145747 0.8521572

4144.71284757907 0.9139252

4144.73091370067 0.8581403

4144.74897982228 0.8852099

4144.76704594388 0.9196795

4144.78511206548 0.7966948

4144.80317818708 0.804706

4144.82124430869 0.7637957

4144.83931043029 0.8391675

4144.85737655189 0.9067971

4144.87544267349 0.8834881

4144.89350879509 0.8309397

4144.9115749167 0.8296034

4144.9296410383 0.900264

4144.9477071599 0.8695295

4144.9657732815 0.8562207

4144.98383940311 0.8786825

4145.00190552471 0.9306617

4145.01997164631 0.9289301

4145.03803776791 0.875086

4145.05610388951 0.8708674

4145.07417001112 0.8202243

4145.09223613272 0.827852

4145.11030225432 0.8956134

4145.12836837592 0.8667498

4145.14643449752 0.8912554

4145.16450061913 0.8801923

4145.18256674073 0.7950357

4145.20063286233 0.8949093

4145.21869898393 0.9016987

4145.23676510554 0.8638998

4145.25483122714 0.8625568

4145.27289734874 0.916611

4145.29096347034 0.908863

4145.30902959195 0.8960019

4145.32709571355 0.8906066

4145.34516183515 0.8839658

4145.36322795675 0.9322356

4145.38129407836 0.8827883

4145.39936019996 0.885614

4145.41742632156 0.8100883

4145.43549244316 0.9065592

4145.45355856476 0.9630709

4145.47162468637 0.9511496

4145.48969080797 0.9130712

4145.50775692957 0.9245738

4145.52582305117 1.010154

4145.54388917277 1.063371

4145.56195529438 0.9635352

4145.58002141598 0.886604

4145.59808753758 0.8929746

4145.61615365918 0.9183911

4145.63421978079 0.998413

4145.65228590239 0.9242773

4145.67035202399 0.9178088

4145.68841814559 0.9700224

4145.70648426719 0.9106547

4145.7245503888 0.9746029

4145.7426165104 0.9513274

4145.760682632 0.966957

4145.7787487536 0.8750318

4145.79681487521 0.9153591

4145.81488099681 0.9084797

4145.83294711841 0.9956838

4145.85101324001 1.027179

4145.86907936161 0.9242231

4145.88714548322 0.9177744

4145.90521160482 0.9081841

4145.92327772642 0.8524852

4145.94134384802 0.8153031

4145.95940996963 0.7936383

4145.97747609123 0.8925185

4145.99554221283 0.9531631

4146.01360833443 0.9197823

4146.03167445603 0.834151

4146.04974057764 0.7776604

4146.06780669924 0.8361187

4146.08587282084 0.8801242

4146.10393894244 0.8151225

4146.12200506405 0.8224323

4146.14007118565 0.8621906

4146.15813730725 0.8443385

4146.17620342885 0.8732003

4146.19426955045 0.8429967

4146.21233567206 0.8318001

4146.23040179366 0.8452809

4146.24846791526 0.8504915

4146.26653403686 0.8739975

4146.28460015847 0.8134928

4146.30266628007 0.8614624

4146.32073240167 0.7865833

4146.33879852327 0.8404896

4146.35686464487 0.920163

4146.37493076648 0.9185749

4146.39299688808 0.8935852

4146.41106300968 0.9282842

4146.42912913128 0.9452921

4146.44719525288 0.926177

4146.46526137449 0.9118658

4146.48332749609 0.9640378

4146.50139361769 0.9618847

4146.51945973929 0.9174003

4146.5375258609 0.9215072

4146.5555919825 0.8699899

4146.5736581041 0.8889896

4146.5917242257 0.9070072

4146.6097903473 0.9122607

4146.62785646891 0.8732263

4146.64592259051 0.9784563

4146.66398871211 0.9863318

4146.68205483371 0.9893579

4146.70012095531 1.02843

4146.71818707692 1.012554

4146.73625319852 0.9384762

4146.75431932012 0.9916794

4146.77238544172 0.9690859

4146.79045156333 0.9350082

4146.80851768493 0.8869429

4146.82658380653 0.9419698

4146.84464992813 0.9364854

4146.86271604973 0.947065

4146.88078217134 0.939396

4146.89884829294 1.000486

4146.91691441454 0.9264042

4146.93498053614 0.899568

4146.95304665775 0.938671

4146.97111277935 0.9040346

4146.98917890095 0.9191668

4147.00724502255 0.9429983

4147.02531114415 0.9189443

4147.04337726576 0.8717944

4147.06144338736 0.9246528

4147.07950950896 0.9292953

4147.09757563056 0.9206387

4147.11564175216 1.003242

4147.13370787377 0.9777756

4147.15177399537 0.9215013

4147.16984011697 0.8713899

4147.18790623857 0.9273926

4147.20597236018 1.007549

4147.22403848178 0.9172456

4147.24210460338 0.9325352

4147.26017072498 0.944506

4147.27823684659 0.9061214

4147.29630296819 0.9133919

4147.31436908979 0.9113368

4147.33243521139 0.9125233

4147.35050133299 0.8878157

4147.3685674546 0.8664948

4147.3866335762 0.8579392

4147.4046996978 0.8628521

4147.4227658194 0.7737707

4147.440831941 0.8546293

4147.45889806261 0.9561443

4147.47696418421 0.9287998

4147.49503030581 0.8882504

4147.51309642741 0.902127

4147.53116254902 0.9062003

4147.54922867062 0.8119938

4147.56729479222 0.8491632

4147.58536091382 0.9051594

4147.60342703543 0.8947901

4147.62149315703 0.8488917

4147.63955927863 0.8772145

4147.65762540023 0.9486294

4147.67569152183 0.9722623

4147.69375764344 0.9597909

4147.71182376504 0.8580111

4147.72988988664 0.9193933

4147.74795600824 0.9477167

4147.76602212984 0.960605

4147.78408825145 0.8861933

4147.80215437305 0.9416802

4147.82022049465 1.013189

4147.83828661625 0.9049923

4147.85635273786 0.9342909

4147.87441885946 0.9372474

4147.89248498106 0.9785814

4147.91055110266 0.9685925

4147.92861722426 1.025816

4147.94668334587 0.8729272

4147.96474946747 0.9189473

4147.98281558907 0.8688699

4148.00088171067 0.9742758

4148.01894783228 1.005791

4148.03701395388 0.9746314

4148.05508007548 1.018802

4148.07314619708 1.014899

4148.09121231868 1.004334

4148.10927844029 1.038152

4148.12734456189 0.9989409

4148.14541068349 0.9427481

4148.16347680509 1.035188

4148.1815429267 0.995708

4148.1996090483 0.8960303

4148.2176751699 0.9840305

4148.2357412915 0.9931725

4148.2538074131 0.913065

4148.27187353471 0.883711

4148.28993965631 0.9819938

4148.30800577791 0.9478313

4148.32607189951 0.9245994

4148.34413802111 0.9776913

4148.36220414272 0.9533019

4148.38027026432 1.002312

4148.39833638592 1.031176

4148.41640250752 0.9196547

4148.43446862913 0.8700496

4148.45253475073 0.9831463

4148.47060087233 1.114595

4148.48866699393 1.043826

4148.50673311553 0.9954413

4148.52479923714 0.9394839

4148.54286535874 0.9821518

4148.56093148034 1.086519

4148.57899760194 1.02343

4148.59706372355 0.9267822

4148.61512984515 0.8826658

4148.63319596675 0.9191165

4148.65126208835 0.9105842

4148.66932820996 0.8648419

4148.68739433156 0.9132154

4148.70546045316 0.9797963

4148.72352657476 0.7973338

4148.74159269636 0.7510651

4148.75965881797 0.9728866

4148.77772493957 0.9666346

4148.79579106117 0.8281543

4148.81385718277 0.8189416

4148.83192330438 0.7826374

4148.84998942598 0.8002893

4148.86805554758 0.9369056

4148.88612166918 0.9798819

4148.90418779078 0.815769

4148.92225391239 0.85793

4148.94032003399 0.9256644

4148.95838615559 0.9966917

4148.97645227719 0.960273

4148.99451839879 0.8526511

4149.0125845204 0.8189039

4149.030650642 0.8603536

4149.0487167636 0.9610616

4149.0667828852 0.9478104

4149.08484900681 1.004269

4149.10291512841 0.8576162

4149.12098125001 0.7437194

4149.13904737161 0.9245454

4149.15711349321 0.7942159

4149.17517961482 0.8238403

4149.19324573642 0.8337612

4149.21131185802 0.8569703

4149.22937797962 0.8385973

4149.24744410123 0.848551

4149.26551022283 0.8026534

4149.28357634443 0.7710143

4149.30164246603 0.7332234

4149.31970858763 0.7245609

4149.33777470924 0.9263896

4149.35584083084 0.8994739

4149.37390695244 0.8111979

4149.39197307404 0.9298207

4149.41003919564 1.049168

4149.42810531725 0.9122095

4149.44617143885 0.8923926

4149.46423756045 0.8680048

4149.48230368205 0.7884443

4149.50036980366 0.7958676

4149.51843592526 0.8446478

4149.53650204686 0.863373

4149.55456816846 0.9741302

4149.57263429007 0.9019345

4149.59070041167 0.8221627

4149.60876653327 0.7857598

4149.62683265487 0.7768136

4149.64489877647 0.9064394

4149.66296489808 0.8199782

4149.68103101968 0.7095003

4149.69909714128 0.9320107

4149.71716326288 0.9275188

4149.73522938448 0.9678195

4149.75329550609 0.8356227

4149.77136162769 0.7771482

4149.78942774929 0.8453096

4149.80749387089 0.7746757

4149.8255599925 0.7107177

4149.8436261141 0.7278203

4149.8616922357 0.7427436

4149.8797583573 0.7853142

4149.8978244789 0.8072774

4149.91589060051 0.8836542

4149.93395672211 0.8001696

4149.95202284371 0.7710286

4149.97008896531 0.8194109

4149.98815508691 0.7752065

4150.00622120852 0.8807537

4150.02428733012 0.9508399

4150.04235345172 0.9472917

4150.06041957332 0.8925475

4150.07848569493 0.9608136

4150.09655181653 0.9580904

4150.11461793813 0.9194249

4150.13268405973 0.8797388

4150.15075018134 0.8649723

4150.16881630294 0.8704599

4150.18688242454 0.8074614

4150.20494854614 0.846819

4150.22301466774 0.7146875

4150.24108078935 0.8878178

4150.25914691095 1.023369

4150.27721303255 0.903599

4150.29527915415 0.7452797

4150.31334527576 0.8298774

4150.33141139736 0.9248844

4150.34947751896 0.965386

4150.36754364056 0.8877861

4150.38560976216 0.7724242

4150.40367588377 0.9576938

4150.42174200537 0.9244728

4150.43980812697 0.9372814

4150.45787424857 0.9597167

4150.47594037018 0.8653922

4150.49400649178 0.892341

4150.51207261338 0.9697254

4150.53013873498 0.9865273

4150.54820485658 0.9570932

4150.56627097819 0.9229735

4150.58433709979 0.9820861

4150.60240322139 0.9471155

4150.62046934299 0.9233552

4150.63853546459 0.9321759

4150.6566015862 0.9580168

4150.6746677078 0.9745194

4150.6927338294 0.9280697

4150.710799951 0.8961576

4150.72886607261 0.8487797

4150.74693219421 0.9156474

4150.76499831581 0.8941295

4150.78306443741 0.7287403

4150.80113055902 0.7443909

4150.81919668062 0.8715935

4150.83726280222 0.7800313

4150.85532892382 0.9061227

4150.87339504542 0.8870714

4150.89146116702 0.8940032

4150.90952728863 0.8902805

4150.92759341023 0.9113673

4150.94565953183 1.152806

4150.96372565343 1.015254

4150.98179177504 0.8684018

4150.99985789664 0.8271509

4151.01792401824 0.8418837

4151.03599013984 0.9370526

4151.05405626145 0.9814236

4151.07212238305 0.9405247

4151.09018850465 0.8448758

4151.10825462625 0.8682692

4151.12632074785 0.9783547

4151.14438686946 1.012393

4151.16245299106 0.9458974

4151.18051911266 0.846316

4151.19858523426 0.8333661

4151.21665135587 0.8552792

4151.23471747747 0.8305799

4151.25278359907 0.840211

4151.27084972067 0.8916648

4151.28891584227 0.8864466

4151.30698196388 0.9596783

4151.32504808548 0.901441

4151.34311420708 0.8716367

4151.36118032868 0.9175885

4151.37924645028 0.9166863

4151.39731257189 0.9566869

4151.41537869349 0.984494

4151.43344481509 0.968242

4151.45151093669 0.9607317

4151.4695770583 0.9054664

4151.4876431799 0.9225414

4151.5057093015 0.9976017

4151.5237754231 1.019559

4151.5418415447 0.9858344

4151.55990766631 0.9282311

4151.57797378791 0.9260716

4151.59603990951 0.9568797

4151.61410603111 0.9481672

4151.63217215271 0.9344724

4151.65023827432 0.9584378

4151.66830439592 0.9045881

4151.68637051752 0.9023103

4151.70443663912 0.9579289

4151.72250276073 0.9481153

4151.74056888233 1.004717

4151.75863500393 0.9370242

4151.77670112553 0.8451813

4151.79476724714 0.8128372

4151.81283336874 0.7814133

4151.83089949034 0.8182211

4151.84896561194 0.8493356

4151.86703173354 0.8305551

4151.88509785514 0.8592901

4151.90316397675 0.8900176

4151.92123009835 0.8645689

4151.93929621995 0.8364426

4151.95736234155 0.8313292

4151.97542846316 0.8603086

4151.99349458476 0.9134197

4152.01156070636 0.9039615

4152.02962682796 0.8735445

4152.04769294956 0.8760568

4152.06575907117 0.8488854

4152.08382519277 0.85434

4152.10189131437 0.9413369

4152.11995743597 0.9513803

4152.13802355758 0.9730651

4152.15608967918 0.9662898

4152.17415580078 0.8849409

4152.19222192238 0.8693458

4152.21028804399 0.8469926

4152.22835416559 0.8598696

4152.24642028719 0.9326641

4152.26448640879 0.9184892

4152.28255253039 0.8663839

4152.300618652 0.8660543

4152.3186847736 0.9198472

4152.3367508952 0.9456795

4152.3548170168 0.9204646

4152.37288313841 0.8810188

4152.39094926001 0.8874967

4152.40901538161 0.9208967

4152.42708150321 0.9414437

4152.44514762481 0.9302454

4152.46321374642 0.932648

4152.48127986802 0.9498835

4152.49934598962 0.9238425

4152.51741211122 0.8912163

4152.53547823282 0.8833426

4152.55354435443 0.9613516

4152.57161047603 0.993848

4152.58967659763 0.8828416

4152.60774271923 0.8155539

4152.62580884084 0.9424203

4152.64387496244 1.013373

4152.66194108404 0.9470541

4152.68000720564 0.9333509

4152.69807332725 0.8953663

4152.71613944885 0.9204312

4152.73420557045 0.9398438

4152.75227169205 0.9263382

4152.77033781365 0.9418361

4152.78840393526 0.9584504

4152.80647005686 1.003298

4152.82453617846 1.05293

4152.84260230006 0.9852563

4152.86066842167 0.9505313

4152.87873454327 0.9762499

4152.89680066487 0.9493748

4152.91486678647 0.9104985

4152.93293290807 0.876393

4152.95099902968 0.8428985

4152.96906515128 0.9018714

4152.98713127288 0.9133943

4153.00519739448 0.9118642

4153.02326351608 0.9117558

4153.04132963769 0.9234728

4153.05939575929 0.8933055

4153.07746188089 0.9064283

4153.09552800249 0.9576646

4153.11359412409 0.9749429

4153.1316602457 0.9380236

4153.1497263673 0.9148992

4153.1677924889 0.8937397

4153.1858586105 0.8858387

4153.20392473211 0.8929251

4153.22199085371 0.9483385

4153.24005697531 0.9326315

4153.25812309691 0.9149674

4153.27618921851 0.9652336

4153.29425534012 0.9077277

4153.31232146172 0.8844832

4153.33038758332 0.9330457

4153.34845370492 0.9555936

4153.36651982653 0.9719569

4153.38458594813 0.960253

4153.40265206973 0.9070053

4153.42071819133 0.904318

4153.43878431294 0.9079682

4153.45685043454 0.9428662

4153.47491655614 0.9556993

4153.49298267774 0.9565181

4153.51104879934 0.921382

4153.52911492095 0.8935894

4153.54718104255 0.9031281

4153.56524716415 0.9255261

4153.58331328575 0.9270582

4153.60137940736 0.928959

4153.61944552896 0.9220886

4153.63751165056 0.9443249

4153.65557777216 0.980076

4153.67364389376 0.9404283

4153.69171001537 0.9164275

4153.70977613697 0.884288

4153.72784225857 0.854622

4153.74590838017 0.9112934

4153.76397450177 0.9567147

4153.78204062338 0.8944867

4153.80010674498 0.8903157

4153.81817286658 0.9283772

4153.83623898818 0.9001187

4153.85430510979 0.8947345

4153.87237123139 0.9163697

4153.89043735299 0.952337

4153.90850347459 0.9424965

4153.92656959619 0.8528081

4153.9446357178 0.88445

4153.9627018394 0.9543118

4153.980767961 0.950915

4153.9988340826 0.9460961

4154.01690020421 0.9465545

4154.03496632581 0.8958161

4154.05303244741 0.9058721

4154.07109856901 0.9942842

4154.08916469061 0.9594454

4154.10723081222 0.9484186

4154.12529693382 0.9369889

4154.14336305542 0.8553143

4154.16142917702 0.8504167

4154.17949529863 0.9092586

4154.19756142023 0.9153652

4154.21562754183 0.9083279

4154.23369366343 0.9380962

4154.25175978503 0.9509386

4154.26982590664 0.951046

4154.28789202824 0.9653829

4154.30595814984 0.9742992

4154.32402427144 0.9493694

4154.34209039305 0.9240105

4154.36015651465 0.9903731

4154.37822263625 1.008884

4154.39628875785 0.9674295

4154.41435487945 0.974528

4154.43242100106 0.9592246

4154.45048712266 0.9921894

4154.46855324426 1.061995

4154.48661936586 0.9324726

4154.50468548747 0.9000649

4154.52275160907 0.9808123

4154.54081773067 1.001971

4154.55888385227 0.9825807

4154.57694997387 0.9395283

4154.59501609548 0.9962248

4154.61308221708 1.015202

4154.63114833868 0.9325824

4154.64921446028 0.9385254

4154.66728058189 0.9609333

4154.68534670349 0.9457904

4154.70341282509 0.8981284

4154.72147894669 0.8878666

4154.73954506829 0.9877938

4154.7576111899 0.9659674

4154.7756773115 0.9167341

4154.7937434331 0.8890722

4154.8118095547 0.8190147

4154.8298756763 0.8457434

4154.84794179791 0.8726101

4154.86600791951 0.9277665

4154.88407404111 0.9636593

4154.90214016271 0.952275

4154.92020628432 0.9441544

4154.93827240592 0.9373927

4154.95633852752 0.9320582

4154.97440464912 0.921113

4154.99247077072 0.8397987

4155.01053689233 0.8885844

4155.02860301393 0.9305323

4155.04666913553 0.8879678

4155.06473525713 0.922214

4155.08280137873 0.9527996

4155.10086750034 0.8960662

4155.11893362194 0.8684519

4155.13699974354 0.9081831

4155.15506586514 0.9090711

4155.17313198675 0.895216

4155.19119810835 0.8785651

4155.20926422995 0.9026077

4155.22733035155 0.9550539

4155.24539647315 0.9274811

4155.26346259476 0.9470085

4155.28152871636 0.9791721

4155.29959483796 0.9872383

4155.31766095956 0.9284039

4155.33572708117 0.9251535

4155.35379320277 0.9458551

4155.37185932437 0.9641392

4155.38992544597 0.9414274

4155.40799156758 0.9310688

4155.42605768918 0.9333704

4155.44412381078 0.9235157

4155.46218993238 0.9791743

4155.48025605398 0.9720054

4155.49832217559 0.928982

4155.51638829719 0.9712404

4155.53445441879 0.9989968

4155.55252054039 0.9988717

4155.57058666199 1.004501

4155.5886527836 0.9805514

4155.6067189052 0.9414284

4155.6247850268 0.9266815

4155.6428511484 0.8922727

4155.66091727001 0.895831

4155.67898339161 0.9369674

4155.69704951321 0.9472081

4155.71511563481 0.9162303

4155.73318175641 0.9629618

4155.75124787802 1.007209

4155.76931399962 0.914013

4155.78738012122 0.8656304

4155.80544624282 0.9296705

4155.82351236443 0.9339253

4155.84157848603 0.8907977

4155.85964460763 0.9180214

4155.87771072923 0.9288002

4155.89577685083 0.9688463

4155.91384297244 0.942398

4155.93190909404 0.9255181

4155.94997521564 0.9465659

4155.96804133724 0.9569311

4155.98610745885 0.984177

4156.00417358045 0.9706937

4156.02223970205 0.9415946

4156.04030582365 0.9131522

4156.05837194525 0.8839641

4156.07643806686 0.9101076

4156.09450418846 0.9376673

4156.11257031006 0.9132965

4156.13063643166 0.8328004

4156.14870255326 0.8381078

4156.16676867487 0.8461252

4156.18483479647 0.862686

4156.20290091807 0.8467617

4156.22096703967 0.8161914

4156.23903316128 0.8509818

4156.25709928288 0.8708911

4156.27516540448 0.8804008

4156.29323152608 0.8711984

4156.31129764768 0.8274642

4156.32936376929 0.8402048

4156.34742989089 0.8406069

4156.36549601249 0.8368776

4156.38356213409 0.8735451

4156.4016282557 0.8720552

4156.4196943773 0.8926313

4156.4377604989 0.893077

4156.4558266205 0.8516883

4156.4738927421 0.8478513

4156.49195886371 0.875415

4156.51002498531 0.9062078

4156.52809110691 0.8945981

4156.54615722851 0.8834792

4156.56422335012 0.9009837

4156.58228947172 0.8882371

4156.60035559332 0.9006795

4156.61842171492 0.88762

4156.63648783652 0.8812405

4156.65455395813 0.9384484

4156.67262007973 0.9083593

4156.69068620133 0.8950452

4156.70875232293 0.9108925

4156.72681844453 0.912578

4156.74488456614 0.9176271

4156.76295068774 0.9400098

4156.78101680934 0.9680931

4156.79908293094 0.9656498

4156.81714905255 0.9312464

4156.83521517415 0.9435058

4156.85328129575 0.9561694

4156.87134741735 0.9560267

4156.88941353895 0.9871351

4156.90747966056 0.9902841

4156.92554578216 0.9716433

4156.94361190376 0.9684678

4156.96167802536 0.9289855

4156.97974414697 0.9264853

4156.99781026857 0.9579589

4157.01587639017 0.9426812

4157.03394251177 0.942277

4157.05200863337 0.9725659

4157.07007475498 0.982392

4157.08814087658 0.9896994

4157.10620699818 1.020044

4157.12427311978 0.995124

4157.14233924138 1.004913

4157.16040536299 1.041322

4157.17847148459 0.9998219

4157.19653760619 0.9640983

4157.21460372779 0.938464

4157.2326698494 0.9670298

4157.250735971 1.045665

4157.2688020926 1.008689

4157.2868682142 0.9571022

4157.3049343358 0.9928727

4157.32300045741 0.9977097

4157.34106657901 0.9655527

4157.35913270061 0.9773672

4157.37719882221 0.9769006

4157.39526494382 0.9712166

4157.41333106542 0.9212087

4157.43139718702 0.9353602

4157.44946330862 0.9537348

4157.46752943023 0.9112073

4157.48559555183 0.93211

4157.50366167343 0.9490972

4157.52172779503 0.9444658

4157.53979391663 0.9351287

4157.55786003824 0.8741404

4157.57592615984 0.8906849

4157.59399228144 0.9177678

4157.61205840304 0.8938951

4157.63012452465 0.8771275

4157.64819064625 0.8961426

4157.66625676785 0.9085078

4157.68432288945 0.9587867

4157.70238901105 0.937324

4157.72045513266 0.8927258

4157.73852125426 0.8698434

4157.75658737586 0.8642589

4157.77465349746 0.8625658

4157.79271961907 0.9371334

4157.81078574067 0.9935231

4157.82885186227 0.950086

4157.84691798387 0.9164555

4157.86498410547 0.9357363

4157.88305022708 0.9316728

4157.90111634868 0.9006256

4157.91918247028 0.8870798

4157.93724859188 0.8923477

4157.95531471349 0.9069636

4157.97338083509 0.920526

4157.99144695669 0.9270862

4158.00951307829 0.9238182

4158.02757919989 0.9167007

4158.0456453215 0.951676

4158.0637114431 0.9969004

4158.0817775647 1.006374

4158.0998436863 0.95063

4158.1179098079 0.9376816

4158.13597592951 0.9707973

4158.15404205111 0.9887962

4158.17210817271 0.9235054

4158.19017429431 0.9017161

4158.20824041592 0.9630373

4158.22630653752 0.956377

4158.24437265912 0.9518833

4158.26243878072 0.9914232

4158.28050490232 0.9489526

4158.29857102393 0.9311486

4158.31663714553 0.9250594

4158.33470326713 0.9351855

4158.35276938873 1.026775

4158.37083551034 1.001776

4158.38890163194 0.9412819

4158.40696775354 0.9611864

4158.42503387514 0.9381424

4158.44309999674 0.9500248

4158.46116611835 0.9774289

4158.47923223995 0.9427922

4158.49729836155 0.9485645

4158.51536448315 0.9886153

4158.53343060475 0.9708835

4158.55149672636 0.908291

4158.56956284796 0.9413335

4158.58762896956 0.9891522

4158.60569509116 0.9744018

4158.62376121277 0.9707448

4158.64182733437 0.9586896

4158.65989345597 1.000958

4158.67795957757 0.9757481

4158.69602569917 0.9710394

4158.71409182078 1.004782

4158.73215794238 1.00199

4158.75022406398 0.9714216

4158.76829018558 0.9844865

4158.78635630719 1.003592

4158.80442242879 1.00336

4158.82248855039 1.007832

4158.84055467199 0.9830122

4158.85862079359 0.942017

4158.8766869152 0.9572601

4158.8947530368 0.9316885

4158.9128191584 0.936766

4158.93088528 0.9727566

4158.94895140161 0.9951489

4158.96701752321 0.966427

4158.98508364481 0.9746722

4159.00314976641 0.9944986

4159.02121588801 0.9822029

4159.03928200962 0.9624569

4159.05734813122 0.9269642

4159.07541425282 0.9396138

4159.09348037442 0.9220934

4159.11154649602 0.963685

4159.12961261763 1.003651

4159.14767873923 0.9828511

4159.16574486083 0.9318221

4159.18381098243 0.9253799

4159.20187710404 0.9422512

4159.21994322564 0.9123446

4159.23800934724 0.9759086

4159.25607546884 0.9752104

4159.27414159044 0.949071

4159.29220771205 0.9516915

4159.31027383365 0.9737133

4159.32833995525 0.9067888

4159.34640607685 0.9497836

4159.36447219846 0.978772

4159.38253832006 0.9757261

4159.40060444166 0.9993541

4159.41867056326 1.001686

4159.43673668486 1.01467

4159.45480280647 0.9874212

4159.47286892807 0.9238952

4159.49093504967 0.8943186

4159.50900117127 0.9243391

4159.52706729288 0.9341877

4159.54513341448 0.9197478

4159.56319953608 0.9645395

4159.58126565768 0.9689398

4159.59933177929 0.9236874

4159.61739790089 0.9609672

4159.63546402249 0.9929738

4159.65353014409 1.015451

4159.67159626569 1.008834

4159.6896623873 1.011775

4159.7077285089 1.017947

4159.7257946305 1.007368

4159.7438607521 0.9859814

4159.7619268737 1.008289

4159.77999299531 0.9860876

4159.79805911691 1.008463

4159.81612523851 0.99568

4159.83419136011 0.9745958

4159.85225748172 1.014078

4159.87032360332 1.029858

4159.88838972492 1.007001

4159.90645584652 0.9698871

4159.92452196812 0.982338

4159.94258808973 1.008301

4159.96065421133 1.019804

4159.97872033293 0.9141705

4159.99678645453 0.8514166

4160.01485257614 0.9419991

4160.03291869774 0.9905689

4160.05098481934 0.977483

4160.06905094094 0.9869669

4160.08711706254 0.9250335

4160.10518318415 0.9226732

4160.12324930575 0.9540787

4160.14131542735 0.9538993

4160.15938154895 0.9349258

4160.17744767056 0.9506729

4160.19551379216 0.9436368

4160.21357991376 0.9429501

4160.23164603536 0.9770967

4160.24971215696 0.9539118

4160.26777827856 0.9510038

4160.28584440017 0.9512634

4160.30391052177 0.946853

4160.32197664337 0.9357324

4160.34004276497 0.9586586

4160.35810888658 0.9726466

4160.37617500818 0.9358038

4160.39424112978 0.950762

4160.41230725138 0.9888841

4160.43037337299 0.9702072

4160.44843949459 0.9454902

4160.46650561619 0.936079

4160.48457173779 0.9432628

4160.50263785939 0.9111758

4160.520703981 0.8764561

4160.5387701026 0.9349891

4160.5568362242 0.9705487

4160.5749023458 0.9705431

4160.59296846741 0.9683836

4160.61103458901 0.9354978

4160.62910071061 0.9230821

4160.64716683221 0.9341716

4160.66523295381 0.9174786

4160.68329907542 0.937183

4160.70136519702 0.9407107

4160.71943131862 0.9724499

4160.73749744022 1.017069

4160.75556356183 0.968588

4160.77362968343 0.9086193

4160.79169580503 0.907285

4160.80976192663 0.9271674

4160.82782804823 0.9085636

4160.84589416984 0.9242219

4160.86396029144 0.9511416

4160.88202641304 0.9834579

4160.90009253464 0.9961724

4160.91815865624 0.9732637

4160.93622477785 0.9925395

4160.95429089945 1.01011

4160.97235702105 0.9870669

4160.99042314265 0.9662936

4161.00848926426 0.9444395

4161.02655538586 0.9322333

4161.04462150746 0.9653096

4161.06268762906 1.013323

4161.08075375066 1.022682

4161.09881987227 0.965165

4161.11688599387 0.8997216

4161.13495211547 0.9422733

4161.15301823707 0.9410672

4161.17108435868 0.8966529

4161.18915048028 0.8681619

4161.20721660188 0.8758283

4161.22528272348 0.9294757

4161.24334884508 0.9590474

4161.26141496669 0.9293312

4161.27948108829 0.9066826

4161.29754720989 0.9156474

4161.31561333149 0.9004141

4161.33367945309 0.8770543

4161.3517455747 0.8881993

4161.3698116963 0.882795

4161.3878778179 0.8768641

4161.4059439395 0.8947317

4161.42401006111 0.9064493

4161.44207618271 0.8705206

4161.46014230431 0.8819842

4161.47820842591 0.8844403

4161.49627454751 0.8898032

4161.51434066912 0.8815732

4161.53240679072 0.8733125

4161.55047291232 0.9026748

4161.56853903392 0.940245

4161.58660515553 0.9121164

4161.60467127713 0.869414

4161.62273739873 0.8777819

4161.64080352033 0.8932564

4161.65886964193 0.8708881

4161.67693576354 0.8218997

4161.69500188514 0.827064

4161.71306800674 0.8861853

4161.73113412834 0.918653

4161.74920024995 0.8989799

4161.76726637155 0.8751481

4161.78533249315 0.8916683

4161.80339861475 0.8491645

4161.82146473636 0.8261878

4161.83953085796 0.860898

4161.85759697956 0.8630877

4161.87566310116 0.8304594

4161.89372922276 0.8875552

4161.91179534436 0.8984518

4161.92986146597 0.8468877

4161.94792758757 0.8376849

4161.96599370917 0.856095

4161.98405983077 0.8689632

4162.00212595238 0.942883

4162.02019207398 0.9052867

4162.03825819558 0.8591895

4162.05632431718 0.8926872

4162.07439043878 0.9197421

4162.09245656039 0.9169937

4162.11052268199 0.8830408

4162.12858880359 0.940895

4162.14665492519 0.9546031

4162.1647210468 0.9593629

4162.1827871684 0.9325088

4162.20085329 0.906433

4162.2189194116 0.9464757

4162.23698553321 0.9272549

4162.25505165481 0.8948674

4162.27311777641 0.9476554

4162.29118389801 0.9794257

4162.30925001961 1.008713

4162.32731614122 0.9987742

4162.34538226282 0.9719529

4162.36344838442 0.9763909

4162.38151450602 0.9663171

4162.39958062763 0.9875174

4162.41764674923 0.9921653

4162.43571287083 0.9991608

4162.45377899243 1.002489

4162.47184511403 0.9545648

4162.48991123564 0.9490131

4162.50797735724 0.9572607

4162.52604347884 0.985597

4162.54410960044 1.037125

4162.56217572205 1.033894

4162.58024184365 0.9873641

4162.59830796525 0.9563587

4162.61637408685 0.9769232

4162.63444020845 0.9898326

4162.65250633006 0.9000417

4162.67057245166 0.9210429

4162.68863857326 0.9784187

4162.70670469486 0.9797506

4162.72477081647 0.9743389

4162.74283693807 0.9425077

4162.76090305967 0.9486282

4162.77896918127 0.9483665

4162.79703530287 0.9396434

4162.81510142448 0.9278076

4162.83316754608 0.9176427

4162.85123366768 0.9378982

4162.86929978928 0.976644

4162.88736591089 0.9808288

4162.90543203249 0.9294331

4162.92349815409 0.9165517

4162.94156427569 0.9218557

4162.95963039729 0.9178714

4162.9776965189 0.9520449

4162.9957626405 0.9746006

4163.0138287621 0.9609123

4163.0318948837 0.9582893

4163.0499610053 0.9254266

4163.06802712691 0.9164966

4163.08609324851 0.9692115

4163.10415937011 0.9869974

4163.12222549171 0.9450815

4163.14029161332 0.9391332

4163.15835773492 0.9964234

4163.17642385652 0.989694

4163.19448997812 0.9624104

4163.21255609972 0.9819963

4163.23062222133 0.9703817

4163.24868834293 0.9330046

4163.26675446453 0.952374

4163.28482058613 0.9834793

4163.30288670773 0.9731518

4163.32095282934 0.930654

4163.33901895094 0.9232393

4163.35708507254 0.9893488

4163.37515119414 1.002526

4163.39321731575 0.9815884

4163.41128343735 0.9867949

4163.42934955895 0.9741579

4163.44741568055 0.9579393

4163.46548180215 0.9328453

4163.48354792376 0.9076478

4163.50161404536 0.8773885

4163.51968016696 0.9297466

4163.53774628856 0.9098169

4163.55581241017 0.8871329

4163.57387853177 0.8778194

4163.59194465337 0.8627365

4163.61001077497 0.8990976

4163.62807689657 0.9334887

4163.64614301818 0.8677572

4163.66420913978 0.8374504

4163.68227526138 0.8781124

4163.70034138298 0.9146961

4163.71840750459 0.8816714

4163.73647362619 0.8556118

4163.75453974779 0.8424416

4163.77260586939 0.8183193

4163.79067199099 0.8129746

4163.8087381126 0.8327056

4163.8268042342 0.8669877

4163.8448703558 0.8894753

4163.8629364774 0.8910484

4163.88100259901 0.8735841

4163.89906872061 0.8543161

4163.91713484221 0.7939607

4163.93520096381 0.8427546

4163.95326708541 0.8820832

4163.97133320702 0.8553272

4163.98939932862 0.8320116

4164.00746545022 0.8617749

4164.02553157182 0.8832734

4164.04359769343 0.8671474

4164.06166381503 0.8789247

4164.07972993663 0.8739726

4164.09779605823 0.8832142

4164.11586217983 0.9211274

4164.13392830144 0.9064623

4164.15199442304 0.9050188

4164.17006054464 0.9161924

4164.18812666624 0.9349446

4164.20619278785 0.931386

4164.22425890945 0.9031961

4164.24232503105 0.8955085

4164.26039115265 0.9382495

4164.27845727425 0.9806598

4164.29652339586 0.9442437

4164.31458951746 0.9313838

4164.33265563906 0.9847312

4164.35072176066 0.9583391

4164.36878788227 0.9734058

4164.38685400387 0.9860829

4164.40492012547 0.9790502

4164.42298624707 1.016184

4164.44105236867 0.9738785

4164.45911849027 0.9291744

4164.47718461188 0.9997711

4164.49525073348 0.9975902

4164.51331685508 0.9836794

4164.53138297668 0.9552843

4164.54944909829 0.9222448

4164.56751521989 0.9430227

4164.58558134149 0.9966453

4164.60364746309 0.9928033

4164.6217135847 0.9307138

4164.6397797063 0.9504472

4164.6578458279 0.9602861

4164.6759119495 0.9334905

4164.6939780711 0.9163071

4164.71204419271 0.9243867

4164.73011031431 0.9248002

4164.74817643591 0.9502903

4164.76624255751 0.9796546

4164.78430867912 0.9735014

4164.80237480072 0.9375343

4164.82044092232 0.953043

4164.83850704392 0.9775238

4164.85657316552 0.9626521

4164.87463928713 0.9345831

4164.89270540873 0.9261485

4164.91077153033 0.941264

4164.92883765193 0.9870564

4164.94690377354 0.9707854

4164.96496989514 0.9457655

4164.98303601674 1.007494

4165.00110213834 0.988053

4165.01916825994 0.9112989

4165.03723438155 0.9358555

4165.05530050315 0.9108421

4165.07336662475 0.8697384

4165.09143274635 0.8992082

4165.10949886795 0.9075748

4165.12756498956 0.9277216

4165.14563111116 0.9225752

4165.16369723276 0.9158524

4165.18176335436 0.913447

4165.19982947597 0.927295

4165.21789559757 0.9349603

4165.23596171917 0.9376312

4165.25402784077 0.9227555

4165.27209396237 0.9240354

4165.29016008398 0.9743184

4165.30822620558 0.969045

4165.32629232718 0.9398553

4165.34435844878 0.9891322

4165.36242457039 1.00914

4165.38049069199 0.9691185

4165.39855681359 0.9604495

4165.41662293519 0.9765225

4165.43468905679 0.9576417

4165.4527551784 0.9221079

4165.4708213 0.9254773

4165.4888874216 0.9223714

4165.5069535432 0.8891771

4165.5250196648 0.9357659

4165.54308578641 0.9449804

4165.56115190801 0.950974

4165.57921802961 0.9803333

4165.59728415121 0.9734601

4165.61535027282 0.9844159

4165.63341639442 0.9841328

4165.65148251602 0.9319848

4165.66954863762 0.9377173

4165.68761475922 0.9422023

4165.70568088083 0.9361359

4165.72374700243 0.9484932

4165.74181312403 0.9547745

4165.75987924563 0.9144554

4165.77794536724 0.9293213

4165.79601148884 0.9620389

4165.81407761044 0.9569994

4165.83214373204 1.006187

4165.85020985364 0.9817435

4165.86827597525 0.9514948

4165.88634209685 0.9408814

4165.90440821845 0.9202063

4165.92247434005 0.9181103

4165.94054046166 0.9716066

4165.95860658326 0.9845512

4165.97667270486 0.9534388

4165.99473882646 0.9282607

4166.01280494807 0.9686497

4166.03087106967 0.9613519

4166.04893719127 0.9655049

4166.06700331287 0.9758792

4166.08506943447 0.9448825

4166.10313555607 0.9744744

4166.12120167768 0.9539394

4166.13926779928 0.9477894

4166.15733392088 0.9678446

4166.17540004248 0.9858903

4166.19346616409 0.988711

4166.21153228569 0.9799929

4166.22959840729 0.9624839

4166.24766452889 1.00238

4166.2657306505 0.9740901

4166.2837967721 0.9508952

4166.3018628937 1.000832

4166.3199290153 0.9999225

4166.3379951369 0.9531795

4166.35606125851 0.9391382

4166.37412738011 0.9259389

4166.39219350171 0.9153169

4166.41025962331 0.951105

4166.42832574492 0.9624699

4166.44639186652 0.9560047

4166.46445798812 0.9835054

4166.48252410972 1.00033

4166.50059023132 0.9625798

4166.51865635293 0.9427533

4166.53672247453 0.9417887

4166.55478859613 0.9782867

4166.57285471773 0.9540606

4166.59092083934 0.9801707

4166.60898696094 1.026432

4166.62705308254 0.9446137

4166.64511920414 0.8601846

4166.66318532574 0.8841183

4166.68125144734 0.921856

4166.69931756895 0.9345771

4166.71738369055 0.9414629

4166.73544981215 0.9278068

4166.75351593375 0.873037

4166.77158205536 0.8749783

4166.78964817696 0.899408

4166.80771429856 0.8997573

4166.82578042016 0.8889835

4166.84384654177 0.9016569

4166.86191266337 0.8806103

4166.87997878497 0.8515275

4166.89804490657 0.8604665

4166.91611102818 0.8703027

4166.93417714978 0.8533748

4166.95224327138 0.8127364

4166.97030939298 0.857281

4166.98837551458 0.8811563

4167.00644163619 0.8573927

4167.02450775779 0.8295876

4167.04257387939 0.8854114

4167.06064000099 0.8513955

4167.07870612259 0.8076292

4167.0967722442 0.85958

4167.1148383658 0.8646407

4167.1329044874 0.8156828

4167.150970609 0.8363129

4167.16903673061 0.85158

4167.18710285221 0.8249173

4167.20516897381 0.8372632

4167.22323509541 0.8420014

4167.24130121701 0.8467989

4167.25936733862 0.9077107

4167.27743346022 0.8875192

4167.29549958182 0.8369166

4167.31356570342 0.8293787

4167.33163182503 0.8627783

4167.34969794663 0.8753964

4167.36776406823 0.9178865

4167.38583018983 0.942589

4167.40389631143 0.897365

4167.42196243304 0.8787464

4167.44002855464 0.8888488

4167.45809467624 0.9002321

4167.47616079784 0.9041876

4167.49422691944 0.8431431

4167.51229304105 0.8575477

4167.53035916265 0.9301761

4167.54842528425 0.9044199

4167.56649140585 0.8656617

4167.58455752746 0.8616613

4167.60262364906 0.9351838

4167.62068977066 0.9533259

4167.63875589226 0.8779478

4167.65682201387 0.8747488

4167.67488813547 0.8846138

4167.69295425707 0.8800342

4167.71102037867 0.8751882

4167.72908650027 0.8860054

4167.74715262188 0.9143954

4167.76521874348 0.8858759

4167.78328486508 0.873928

4167.80135098668 0.9247971

4167.81941710828 0.9368069

4167.83748322989 0.9015683

4167.85554935149 0.870877

4167.87361547309 0.8781627

4167.89168159469 0.8777896

4167.9097477163 0.9064996

4167.9278138379 0.9453365

4167.9458799595 0.9377438

4167.9639460811 0.9289283

4167.9820122027 0.9365599

4168.00007832431 0.9574103

4168.01814444591 0.9185388

4168.03621056751 0.9175342

4168.05427668911 0.9427627

4168.07234281072 0.9163216

4168.09040893232 0.9150863

4168.10847505392 0.9230199

4168.12654117552 0.9515634

4168.14460729712 0.9616423

4168.16267341873 1.006057

4168.18073954033 0.9826701

4168.19880566193 0.951763

4168.21687178353 0.9439771

4168.23493790514 0.9295902

4168.25300402674 0.9196098

4168.27107014834 0.9327918

4168.28913626994 0.9754808

4168.30720239154 0.9296109

4168.32526851315 0.9380491

4168.34333463475 1.002166

4168.36140075635 0.9884704

4168.37946687795 0.932067

4168.39753299955 0.9247553

4168.41559912116 0.9641156

4168.43366524276 0.99843

4168.45173136436 1.012247

4168.46979748596 0.9786319

4168.48786360757 0.9441648

4168.50592972917 0.9450865

4168.52399585077 0.934558

4168.54206197237 0.9265059

4168.56012809397 0.9391412

4168.57819421558 0.9044124

4168.59626033718 0.9268007

4168.61432645878 0.9613582

4168.63239258038 0.9199822

4168.65045870198 0.9537023

4168.66852482359 0.9825777

4168.68659094519 0.9122392

4168.70465706679 0.9191681

4168.72272318839 0.9522661

4168.74078931 0.9476807

4168.7588554316 0.949115

4168.7769215532 0.9557273

4168.7949876748 0.9607471

4168.81305379641 0.8925427

4168.83111991801 0.882231

4168.84918603961 0.9347439

4168.86725216121 0.9303156

4168.88531828281 0.9403231

4168.90338440442 0.94583

4168.92145052602 0.8985767

4168.93951664762 0.9336871

4168.95758276922 0.9386007

4168.97564889083 0.917547

4168.99371501243 0.9259633

4169.01178113403 0.9355118

4169.02984725563 0.9243684

4169.04791337723 0.9388474

4169.06597949884 0.9396601

4169.08404562044 0.9282172

4169.10211174204 0.9357505

4169.12017786364 0.9201889

4169.13824398525 0.9352372

4169.15631010685 0.9243863

4169.17437622845 0.8981159

4169.19244235005 0.90866

4169.21050847165 0.9033397

4169.22857459326 0.9237471

4169.24664071486 0.9464912

4169.26470683646 0.9459605

4169.28277295806 0.9704868

4169.30083907966 0.9397526

4169.31890520127 0.9121612

4169.33697132287 0.9404411

4169.35503744447 0.9300016

4169.37310356607 0.9289752

4169.39116968768 0.9621181

4169.40923580928 0.9892895

4169.42730193088 0.954726

4169.44536805248 0.9323161

4169.46343417408 0.9410976

4169.48150029569 0.9038459

4169.49956641729 0.8749686

4169.51763253889 0.9054998

4169.53569866049 0.9240698

4169.5537647821 0.9082081

4169.5718309037 0.8948776

4169.5898970253 0.8948563

4169.6079631469 0.9110304

4169.62602926851 0.9110206

4169.64409539011 0.9214498

4169.66216151171 0.9131638

4169.68022763331 0.909656

4169.69829375491 0.9240416

4169.71635987651 0.9253743

4169.73442599812 0.9113141

4169.75249211972 0.8886155

4169.77055824132 0.8949483

4169.78862436292 0.8864002

4169.80669048453 0.886593

4169.82475660613 0.9106816

4169.84282272773 0.8964009

4169.86088884933 0.8551471

4169.87895497093 0.8634669

4169.89702109254 0.8834203

4169.91508721414 0.8649688

4169.93315333574 0.8664064

4169.95121945734 0.8561077

4169.96928557895 0.853493

4169.98735170055 0.8958322

4170.00541782215 0.8834307

4170.02348394375 0.8729682

4170.04155006536 0.8711176

4170.05961618696 0.8510141

4170.07768230856 0.8591018

4170.09574843016 0.8577175

4170.11381455176 0.8593645

4170.13188067337 0.8707733

4170.14994679497 0.873687

4170.16801291657 0.8721849

4170.18607903817 0.8749409

4170.20414515977 0.8462451

4170.22221128138 0.8510921

4170.24027740298 0.863768

4170.25834352458 0.9479913

4170.27640964618 0.9578179

4170.29447576778 0.902508

4170.31254188939 0.9117264

4170.33060801099 0.930038

4170.34867413259 0.9140226

4170.36674025419 0.8885053

4170.3848063758 0.8609301

4170.4028724974 0.8693643

4170.420938619 0.9277256

4170.4390047406 0.9364467

4170.45707086221 0.8923261

4170.47513698381 0.889033

4170.49320310541 0.8662176

4170.51126922701 0.8591229

4170.52933534861 0.8952615

4170.54740147022 0.9189001

4170.56546759182 0.897958

4170.58353371342 0.9101508

4170.60159983502 0.9296824

4170.61966595663 0.9382216

4170.63773207823 0.9156696

4170.65579819983 0.9026128

4170.67386432143 0.9064432

4170.69193044303 0.8896906

4170.70999656464 0.9265119

4170.72806268624 0.8991739

4170.74612880784 0.9210622

4170.76419492944 0.957274

4170.78226105105 0.9032099

4170.80032717265 0.8484381

4170.81839329425 0.88004

4170.83645941585 0.8910161

4170.85452553745 0.8678565

4170.87259165905 0.8373889

4170.89065778066 0.8386811

4170.90872390226 0.8498424

4170.92679002386 0.876496

4170.94485614546 0.8865061

4170.96292226707 0.8783098

4170.98098838867 0.8587441

4170.99905451027 0.8335899

4171.01712063187 0.8455937

4171.03518675348 0.8606699

4171.05325287508 0.814204

4171.07131899668 0.7961481

4171.08938511828 0.8310629

4171.10745123989 0.7918605

4171.12551736149 0.7760173

4171.14358348309 0.8486227

4171.16164960469 0.8586589

4171.17971572629 0.8296303

4171.1977818479 0.8085513

4171.2158479695 0.8191863

4171.2339140911 0.7973968

4171.2519802127 0.7854859

4171.2700463343 0.8738046

4171.28811245591 0.8676662

4171.30617857751 0.8554876

4171.32424469911 0.8816875

4171.34231082071 0.9329534

4171.36037694232 0.9471712

4171.37844306392 0.9204615

4171.39650918552 0.9107416

4171.41457530712 0.8787292

4171.43264142872 0.8664588

4171.45070755033 0.9052128

4171.46877367193 0.8843461

4171.48683979353 0.895157

4171.50490591513 0.9342743

4171.52297203674 0.9200783

4171.54103815834 0.9167827

4171.55910427994 0.8945206

4171.57717040154 0.8649964

4171.59523652314 0.8669823

4171.61330264475 0.8803229

4171.63136876635 0.9047527

4171.64943488795 0.870655

4171.66750100955 0.8574228

4171.68556713115 0.9036863

4171.70363325276 0.9227455

4171.72169937436 0.8840175

4171.73976549596 0.8879894

4171.75783161756 0.8774495

4171.77589773917 0.8503582

4171.79396386077 0.8457997

4171.81202998237 0.8145828

4171.83009610397 0.8232863

4171.84816222557 0.8768644

4171.86622834718 0.8680037

4171.88429446878 0.8399042

4171.90236059038 0.8287683

4171.92042671198 0.8020777

4171.93849283359 0.7572578

4171.95655895519 0.7845907

4171.97462507679 0.7833571

4171.99269119839 0.7541783

4172.01075731999 0.7232403

4172.0288234416 0.7211025

4172.0468895632 0.7512566

4172.0649556848 0.7512434

4172.0830218064 0.7590563

4172.10108792801 0.7869898

4172.11915404961 0.7813551

4172.13722017121 0.7824367

4172.15528629281 0.8061086

4172.17335241441 0.7975691

4172.19141853602 0.7861249

4172.20948465762 0.81981

4172.22755077922 0.8247536

4172.24561690082 0.8150868

4172.26368302243 0.7948974

4172.28174914403 0.7884929

4172.29981526563 0.7891002

4172.31788138723 0.7913733

4172.33594750883 0.8433095

4172.35401363044 0.8678126

4172.37207975204 0.8682886

4172.39014587364 0.8271816

4172.40821199524 0.836152

4172.42627811685 0.8789672

4172.44434423845 0.8961169

4172.46241036005 0.9028629

4172.48047648165 0.8886862

4172.49854260325 0.9027888

4172.51660872486 0.8657188

4172.53467484646 0.8600408

4172.55274096806 0.8937055

4172.57080708966 0.9188458

4172.58887321126 0.8828803

4172.60693933287 0.8758773

4172.62500545447 0.8888052

4172.64307157607 0.8920022

4172.66113769767 0.8761945

4172.67920381928 0.8794516

4172.69726994088 0.8985661

4172.71533606248 0.8996989

4172.73340218408 0.8965505

4172.75146830568 0.83653

4172.76953442729 0.8232223

4172.78760054889 0.8368981

4172.80566667049 0.8628128

4172.82373279209 0.903093

4172.8417989137 0.8928739

4172.8598650353 0.8905616

4172.8779311569 0.8531997

4172.8959972785 0.9109435

4172.9140634001 0.9002886

4172.93212952171 0.8991219

4172.95019564331 0.9521033

4172.96826176491 0.9278013

4172.98632788651 0.9322318

4173.00439400812 0.9190512

4173.02246012972 0.8998227

4173.04052625132 0.8397103

4173.05859237292 0.859075

4173.07665849452 0.9280258

4173.09472461613 0.9321854

4173.11279073773 0.9218754

4173.13085685933 0.9062493

4173.14892298093 0.8895763

4173.16698910254 0.9207478

4173.18505522414 0.9532672

4173.20312134574 0.9178426

4173.22118746734 0.8958548

4173.23925358894 0.8901442

4173.25731971055 0.8850338

4173.27538583215 0.8567722

4173.29345195375 0.8465613

4173.31151807535 0.8596258

4173.32958419695 0.8792608

4173.34765031856 0.8327295

4173.36571644016 0.7835708

4173.38378256176 0.7625405

4173.40184868336 0.729107

4173.41991480497 0.7330425

4173.43798092657 0.7963743

4173.45604704817 0.789494

4173.47411316977 0.7685808

4173.49217929137 0.7901065

4173.51024541298 0.7689234

4173.52831153458 0.7833467

4173.54637765618 0.7913448

4173.56444377778 0.8011009

4173.58250989939 0.834263

4173.60057602099 0.8021053

4173.61864214259 0.7564141

4173.63670826419 0.741792

4173.65477438579 0.7367877

4173.6728405074 0.7215427

4173.690906629 0.7204021

4173.7089727506 0.7067626

4173.7270388722 0.686011

4173.74510499381 0.7209029

4173.76317111541 0.7866054

4173.78123723701 0.7792507

4173.79930335861 0.7782985

4173.81736948021 0.7814904

4173.83543560182 0.7464691

4173.85350172342 0.8030078

4173.87156784502 0.7691497

4173.88963396662 0.7324449

4173.90770008822 0.7789616

4173.92576620983 0.8103378

4173.94383233143 0.7753105

4173.96189845303 0.7254218

4173.97996457463 0.784732

4173.99803069624 0.8554773

4174.01609681784 0.834589

4174.03416293944 0.8353624

4174.05222906104 0.9175652

4174.07029518264 0.9289457

4174.08836130425 0.8572953

4174.10642742585 0.8927387

4174.12449354745 0.8787645

4174.14255966906 0.8205147

4174.16062579066 0.8646324

4174.17869191226 0.8430231

4174.19675803386 0.8148947

4174.21482415546 0.7994297

4174.23289027707 0.8228606

4174.25095639867 0.8823839

4174.26902252027 0.876381

4174.28708864187 0.8583542

4174.30515476347 0.8088078

4174.32322088508 0.7494001

4174.34128700668 0.8041739

4174.35935312828 0.8499205

4174.37741924988 0.8555275

4174.39548537149 0.891957

4174.41355149309 0.8808985

4174.43161761469 0.8417092

4174.44968373629 0.81499

4174.46774985789 0.867584

4174.4858159795 0.8920509

4174.5038821011 0.8636172

4174.5219482227 0.8535131

4174.5400143443 0.8387466

4174.55808046591 0.8642215

4174.57614658751 0.9037545

4174.59421270911 0.9118069

4174.61227883071 0.8811289

4174.63034495231 0.9223784

4174.64841107392 0.9427958

4174.66647719552 0.9323418

4174.68454331712 0.8999821

4174.70260943872 0.8952981

4174.72067556032 0.9885005

4174.73874168193 0.9942681

4174.75680780353 0.9704967

4174.77487392513 0.9564859

4174.79294004673 0.9491334

4174.81100616834 0.9603626

4174.82907228994 0.9776632

4174.84713841154 0.9856549

4174.86520453314 0.9801347

4174.88327065474 0.92931

4174.90133677635 0.9108187

4174.91940289795 0.9489206

4174.93746901955 0.9930347

4174.95553514115 1.001864

4174.97360126276 1.014584

4174.99166738436 0.9476988

4175.00973350596 0.9316725

4175.02779962756 0.9383212

4175.04586574916 0.9016205

4175.06393187077 0.9050821

4175.08199799237 0.9360412

4175.10006411397 0.9140933

4175.11813023557 0.9306811

4175.13619635717 0.9438677

4175.15426247878 0.8999624

4175.17232860038 0.9074949

4175.19039472198 0.9633952

4175.20846084358 0.9457099

4175.22652696519 0.8898976

4175.24459308679 0.8985212

4175.26265920839 0.9308846

4175.28072532999 0.9418984

4175.29879145159 0.9189347

4175.3168575732 0.9363856

4175.3349236948 0.9692835

4175.3529898164 0.9685131

4175.371055938 0.9719253

4175.38912205961 0.9378881

4175.40718818121 0.9369264

4175.42525430281 0.9613538

4175.44332042441 0.9230253

4175.46138654601 0.9020119

4175.47945266762 0.9461058

4175.49751878922 0.9246224

4175.51558491082 0.9362186

4175.53365103242 0.9857789

4175.55171715402 0.9673193

4175.56978327563 0.9213152

4175.58784939723 0.940188

4175.60591551883 0.9619913

4175.62398164043 0.9369475

4175.64204776204 0.9510921

4175.66011388364 0.9325677

4175.67818000524 0.9265621

4175.69624612684 0.9286637

4175.71431224845 0.8812732

4175.73237837005 0.9170831

4175.75044449165 0.9126874

4175.76851061325 0.9437628

4175.78657673485 0.9177121

4175.80464285646 0.9396665

4175.82270897806 0.9019189

4175.84077509966 0.9092442

4175.85884122126 0.901646

4175.87690734286 0.9196703

4175.89497346447 0.9694352

4175.91303958607 0.9107479

4175.93110570767 0.906876

4175.94917182927 0.9784857

4175.96723795088 0.9590403

4175.98530407248 0.9206859

4176.00337019408 0.9245566

4176.02143631568 0.9395673

4176.03950243728 0.9275

4176.05756855889 0.9269792

4176.07563468049 0.9060261

4176.09370080209 0.90899

4176.11176692369 0.9354272

4176.1298330453 0.8793908

4176.1478991669 0.9750491

4176.1659652885 0.9668723

4176.1840314101 0.9300683

4176.2020975317 0.9070517

4176.22016365331 0.9580427

4176.23822977491 0.8994942

4176.25629589651 0.8704158

4176.27436201811 0.9547222

4176.29242813972 0.9613791

4176.31049426132 0.9165614

4176.32856038292 0.9142421

4176.34662650452 0.9385565

4176.36469262613 0.9715872

4176.38275874773 0.9959143

4176.40082486933 1.001743

4176.41889099093 1.046596

4176.43695711253 0.997858

4176.45502323414 1.02052

4176.47308935574 0.9896264

4176.49115547734 0.928442

4176.50922159894 0.925746

4176.52728772054 0.9679725

4176.54535384215 0.9350493

4176.56341996375 0.8894079

4176.58148608535 0.9073896

4176.59955220695 0.9679569

4176.61761832856 0.9735771

4176.63568445016 0.9767683

4176.65375057176 0.9774724

4176.67181669336 0.9288143

4176.68988281496 0.963634

4176.70794893657 0.9583296

4176.72601505817 0.9559349

4176.74408117977 1.023841

4176.76214730137 1.005191

4176.78021342297 0.9388509

4176.79827954458 0.9406234

4176.81634566618 0.9491714

4176.83441178778 1.001874

4176.85247790938 0.9584489

4176.87054403099 1.015009

4176.88861015259 0.9960555

4176.90667627419 0.9063511

4176.92474239579 0.8837085

4176.9428085174 0.9518557

4176.960874639 0.9380741

4176.9789407606 0.95074

4176.9970068822 0.9550366

4177.0150730038 0.9322119

4177.03313912541 0.9720001

4177.05120524701 1.003015

4177.06927136861 0.9739286

4177.08733749021 0.9780239

4177.10540361181 1.012031

4177.12346973342 1.007274

4177.14153585502 1.01162

4177.15960197662 0.9584798

4177.17766809822 0.8846706

4177.19573421983 0.8824456

4177.21380034143 0.9205471

4177.23186646303 0.9220086

4177.24993258463 0.9604587

4177.26799870623 0.9048971

4177.28606482784 0.8881326

4177.30413094944 0.8670707

4177.32219707104 0.9270859

4177.34026319264 0.9227695

4177.35832931425 0.8995261

4177.37639543585 0.9739993

4177.39446155745 0.9634855

4177.41252767905 0.8492986

4177.43059380065 0.8193306

4177.44865992226 0.8877813

4177.46672604386 0.9370559

4177.48479216546 0.8913743

4177.50285828706 0.9044205

4177.52092440866 0.9569263

4177.53899053027 1.021762

4177.55705665187 1.050216

4177.57512277347 0.9195678

4177.59318889507 0.8883235

4177.61125501668 0.8403659

4177.62932113828 0.8563467

4177.64738725988 0.9297884

4177.66545338148 0.8585079

4177.68351950308 0.7797071

4177.70158562469 0.8070256

4177.71965174629 0.7930216

4177.73771786789 0.8998239

4177.75578398949 0.9046365

4177.7738501111 0.7977934

4177.7919162327 0.8224048

4177.8099823543 0.8568957

4177.8280484759 0.8636507

4177.8461145975 0.9296652

4177.86418071911 0.8107761

4177.88224684071 0.8170436

4177.90031296231 0.8060586

4177.91837908391 0.8332086

4177.93644520552 0.7959796

4177.95451132712 0.7825508

4177.97257744872 0.8357177

4177.99064357032 0.8911164

4178.00870969193 0.8653153

4178.02677581353 0.8424047

4178.04484193513 0.8800461

4178.06290805673 0.9109681

4178.08097417833 0.9217652

4178.09904029993 0.8204634

4178.11710642154 0.789566

4178.13517254314 0.8156396

4178.15323866474 0.8540018

4178.17130478634 0.8868761

4178.18937090795 0.964762

4178.20743702955 1.024993

4178.22550315115 1.014445

4178.24356927275 1.007476

4178.26163539435 0.945518

4178.27970151596 0.9753558

4178.29776763756 0.9158552

4178.31583375916 0.9007192

4178.33389988076 0.8833871

4178.35196600237 0.887289

4178.37003212397 0.9631724

4178.38809824557 1.010636

4178.40616436717 1.033859

4178.42423048877 0.9711016

4178.44229661038 0.8795155

4178.46036273198 0.8641837

4178.47842885358 0.9456205

4178.49649497518 1.033199

4178.51456109679 0.9457733

4178.53262721839 0.9437414

4178.55069333999 1.014811

4178.56875946159 1.031131

4178.58682558319 0.9912832

4178.6048917048 0.9816339

4178.6229578264 0.9753308

4178.641023948 0.9674219

4178.6590900696 0.9974079

4178.6771561912 0.9190561

4178.69522231281 0.8451797

4178.71328843441 0.8438023

4178.73135455601 0.8262934

4178.74942067761 0.8256564

4178.76748679922 0.8320199

4178.78555292082 0.7743294

4178.80361904242 0.7843343

4178.82168516402 0.841424

4178.83975128563 0.8630375

4178.85781740723 0.8754277

4178.87588352883 0.8897104

4178.89394965043 0.8868881

4178.91201577204 0.8477263

4178.93008189364 0.7363014

4178.94814801524 0.7188426

4178.96621413684 0.7519951

4178.98428025844 0.7521421

4179.00234638005 0.7895572

4179.02041250165 0.8118092

4179.03847862325 0.8070363

4179.05654474485 0.7319205

4179.07461086645 0.7098504

4179.09267698806 0.7773913

4179.11074310966 0.6803598

4179.12880923126 0.6515972

4179.14687535286 0.6817174

4179.16494147447 0.7541187

4179.18300759607 0.7454798

4179.20107371767 0.7966941

4179.21913983927 0.7071397

4179.23720596087 0.6748386

4179.25527208248 0.7582353

4179.27333820408 0.7411389

4179.29140432568 0.7906431

4179.30947044728 0.8321452

4179.32753656889 0.8192497

4179.34560269049 0.8138275

4179.36366881209 0.7515363

4179.38173493369 0.8133402

4179.39980105529 0.8883446

4179.4178671769 0.8371606

4179.4359332985 0.785831

4179.4539994201 0.7729862

4179.4720655417 0.7892478

4179.4901316633 0.8478853

4179.50819778491 0.7839488

4179.52626390651 0.7613128

4179.54433002811 0.8424304

4179.56239614971 0.9001923

4179.58046227132 0.8801017

4179.59852839292 0.7985499

4179.61659451452 0.8543916

4179.63466063612 0.9744643

4179.65272675772 1.025815

4179.67079287933 0.9382796

4179.68885900093 0.8549273

4179.70692512253 0.8919495

4179.72499124413 0.7540327

4179.74305736574 0.6816564

4179.76112348734 0.8142953

4179.77918960894 0.8846908

4179.79725573054 0.7911094

4179.81532185214 0.8369431

4179.83338797375 1.021676

4179.85145409535 1.064879

4179.86952021695 0.8418454

4179.88758633855 0.6132928

4179.90565246016 0.6975031

4179.92371858176 0.8308516

4179.94178470336 0.8474571

4179.95985082496 0.8744764

4179.97791694656 0.890627

4179.99598306817 0.9907666

4180.01404918977 0.9669366

4180.03211531137 0.9085185

4180.05018143297 0.8540246

4180.06824755457 0.8251447

4180.08631367618 0.845173

4180.10437979778 0.887678

4180.12244591938 0.8716373

4180.14051204098 0.8933967

4180.15857816259 0.9367289

4180.17664428419 0.9645453

4180.19471040579 0.8752319

4180.21277652739 0.9627797

4180.23084264899 0.8565421

4180.2489087706 0.9550103

4180.2669748922 0.9376247

4180.2850410138 0.7714128

4180.3031071354 0.7292832

4180.32117325701 0.7011127

4180.33923937861 0.7522692

4180.35730550021 0.84421

4180.37537162181 0.8782537

4180.39343774341 0.9747336

4180.41150386502 0.9446954

4180.42956998662 0.9334552

4180.44763610822 0.9571051

4180.46570222982 0.9665308

4180.48376835143 0.9604113

4180.50183447303 0.8005751

4180.51990059463 0.8776473

4180.53796671623 0.9462763

4180.55603283783 0.8661717

4180.57409895944 0.839547

4180.59216508104 0.9033766

4180.61023120264 0.9141352

4180.62829732424 0.9249828

4180.64636344585 0.9483559

4180.66442956745 0.904395

4180.68249568905 0.9694022

4180.70056181065 0.9653261

4180.71862793225 0.9738256

4180.73669405386 0.9542009

4180.75476017546 0.9439935

4180.77282629706 0.8233621

4180.79089241866 0.8898293

4180.80895854027 0.9120241

4180.82702466187 0.8706704

4180.84509078347 0.8550681

4180.86315690507 0.9281431

4180.88122302667 0.8947161

4180.89928914828 0.8386107

4180.91735526988 0.6988769

4180.93542139148 0.757478

4180.95348751308 0.8592857

4180.97155363468 0.7697598

4180.98961975629 0.8392302

4181.00768587789 0.8881578

4181.02575199949 0.8350126

4181.04381812109 0.7930484

4181.0618842427 0.8036538

4181.0799503643 0.8468219

4181.0980164859 0.8512738

4181.1160826075 0.808479

4181.13414872911 0.8370291

4181.15221485071 0.8329322

4181.17028097231 0.847275

4181.18834709391 0.8128526

4181.20641321551 0.7562928

4181.22447933712 0.7879465

4181.24254545872 0.8502417

4181.26061158032 0.8453442

4181.27867770192 0.8625653

4181.29674382352 0.8728213

4181.31480994513 0.8703538

4181.33287606673 0.8756751

4181.35094218833 0.8744438

4181.36900830993 0.9490258

4181.38707443154 1.019795

4181.40514055314 0.9547682

4181.42320667474 0.8483469

4181.44127279634 0.8924496

4181.45933891794 0.9946373

4181.47740503955 0.9623082

4181.49547116115 0.8812131

4181.51353728275 0.9013043

4181.53160340435 0.9977192

4181.54966952596 0.9612895

4181.56773564756 0.9147491

4181.58580176916 0.9668114

4181.60386789076 0.9316497

4181.62193401236 0.9305998

4181.64000013397 0.9458946

4181.65806625557 1.008559

4181.67613237717 0.9869777

4181.69419849877 0.9677863

4181.71226462037 1.018583

4181.73033074198 0.961976

4181.74839686358 0.9674653

4181.76646298518 0.8776141

4181.78452910678 0.8364025

4181.80259522839 0.9382404

4181.82066134999 0.9627555

4181.83872747159 0.9620781

4181.85679359319 0.9320291

4181.87485971479 0.8959129

4181.8929258364 0.9013532

4181.910991958 0.9229784

4181.9290580796 0.8799892

4181.9471242012 0.8520421

4181.96519032281 0.9303901

4181.98325644441 0.9031632

4182.00132256601 0.9215454

4182.01938868761 0.905542

4182.03745480921 0.915615

4182.05552093082 0.9694167

4182.07358705242 0.9234178

4182.09165317402 0.8883356

4182.10971929562 0.9045646

4182.12778541723 0.9566554

4182.14585153883 0.9320629

4182.16391766043 0.8390442

4182.18198378203 0.7708131

4182.20004990363 0.8105546

4182.21811602524 0.8694837

4182.23618214684 0.8529243

4182.25424826844 0.8144882

4182.27231439004 0.8514393

4182.29038051164 0.946043

4182.30844663325 0.9508889

4182.32651275485 0.9156169

4182.34457887645 0.8711585

4182.36264499805 0.8529879

4182.38071111966 0.8630196

4182.39877724126 0.8652323

4182.41684336286 0.8426032

4182.43490948446 0.8317234

4182.45297560606 0.9059207

4182.47104172767 0.9849942

4182.48910784927 0.9433985

4182.50717397087 0.8903725

4182.52524009247 0.9022354

4182.54330621408 0.9153028

4182.56137233568 0.9320481

4182.57943845728 0.8854053

4182.59750457888 0.8295676

4182.61557070048 0.8131734

4182.63363682209 0.8582498

4182.65170294369 0.8766009

4182.66976906529 0.871569

4182.68783518689 0.9089311

4182.7059013085 0.9320616

4182.7239674301 0.93492

4182.7420335517 0.9139954

4182.7600996733 0.8814862

4182.77816579491 0.924472

4182.79623191651 0.9818665

4182.81429803811 0.9754222

4182.83236415971 0.9316759

4182.85043028131 0.9590939

4182.86849640291 0.9885914

4182.88656252452 1.031664

4182.90462864612 0.9925816

4182.92269476772 0.940575

4182.94076088933 0.9242169

4182.95882701093 0.9863458

4182.97689313253 0.9447548

4182.99495925413 0.8741804

4183.01302537573 0.9060816

4183.03109149734 0.8510303

4183.04915761894 0.8803308

4183.06722374054 0.9480557

4183.08528986214 0.9696294

4183.10335598375 0.9602848

4183.12142210535 0.9468738

4183.13948822695 0.9873534

4183.15755434855 0.9899261

4183.17562047015 0.9957112

4183.19368659176 0.9850883

4183.21175271336 0.9324156

4183.22981883496 0.9458604

4183.24788495656 0.949661

4183.26595107816 0.9148325

4183.28401719977 0.9256605

4183.30208332137 0.9960123

4183.32014944297 0.9789854

4183.33821556457 0.9483942

4183.35628168618 0.990113

4183.37434780778 0.9391693

4183.39241392938 0.8670352

4183.41048005098 0.9503758

4183.42854617258 0.9707645

4183.44661229419 0.8815074

4183.46467841579 0.9131212

4183.48274453739 0.9565635

4183.50081065899 0.9133111

4183.51887678059 0.9303346

4183.5369429022 0.9389816

4183.5550090238 0.8910912

4183.5730751454 0.869105

4183.591141267 0.8476659

4183.60920738861 0.8745981

4183.62727351021 0.9533741

4183.64533963181 0.9400693

4183.66340575341 0.9371639

4183.68147187501 0.9936527

4183.69953799662 0.9345568

4183.71760411822 0.8931683

4183.73567023982 0.9579872

4183.75373636142 0.9506406

4183.77180248303 0.897715

4183.78986860463 0.8917756

4183.80793472623 0.8538624

4183.82600084783 0.9220548

4183.84406696944 0.9015263

4183.86213309104 0.9156704

4183.88019921264 0.894262

4183.89826533424 0.8900484

4183.91633145584 0.9054462

4183.93439757745 0.8959639

4183.95246369905 0.9711468

4183.97052982065 1.007028

4183.98859594225 0.9424171

4184.00666206385 0.9243804

4184.02472818546 0.9396092

4184.04279430706 0.959716

4184.06086042866 0.9818342

4184.07892655026 0.9537027

4184.09699267187 0.9237363

4184.11505879347 0.9136138

4184.13312491507 0.9072255

4184.15119103667 0.8165544

4184.16925715827 0.8252913

4184.18732327988 0.8594629

4184.20538940148 0.8993223

4184.22345552308 0.877784

4184.24152164468 0.9194853

4184.25958776628 0.8988954

4184.27765388789 0.8877422

4184.29572000949 0.8596381

4184.31378613109 0.8241614

4184.33185225269 0.8843674

4184.3499183743 0.8717123

4184.3679844959 0.8246786

4184.3860506175 0.8097422

4184.4041167391 0.7876966

4184.4221828607 0.8105756

4184.44024898231 0.8216602

4184.45831510391 0.7915145

4184.47638122551 0.7825848

4184.49444734711 0.7630014

4184.51251346872 0.7536371

4184.53057959032 0.7566762

4184.54864571192 0.7403606

4184.56671183352 0.7909875

4184.58477795512 0.8398426

4184.60284407673 0.8248479

4184.62091019833 0.8183513

4184.63897631993 0.7939048

4184.65704244153 0.8061389

4184.67510856314 0.8377187

4184.69317468474 0.8200564

4184.71124080634 0.7867062

4184.72930692794 0.8841239

4184.74737304954 0.925246

4184.76543917115 0.8362932

4184.78350529275 0.869728

4184.80157141435 0.9155935

4184.81963753595 0.8970991

4184.83770365756 0.8963873

4184.85576977916 0.930854

4184.87383590076 0.9219592

4184.89190202236 0.9092829

4184.90996814396 0.8989114

4184.92803426557 0.8718718

4184.94610038717 0.8528001

4184.96416650877 0.8822759

4184.98223263037 0.9192529

4185.00029875198 0.9058932

4185.01836487358 0.8849454

4185.03643099518 0.86092

4185.05449711678 0.9025301

4185.07256323838 0.9415518

4185.09062935999 0.8585944

4185.10869548159 0.8888701

4185.12676160319 0.8726423

4185.14482772479 0.8860939

4185.16289384639 0.9498526

4185.180959968 0.9406373

4185.1990260896 0.89387

4185.2170922112 0.9614428

4185.2351583328 0.9817418

4185.25322445441 0.970208

4185.27129057601 0.9533113

4185.28935669761 0.9573095

4185.30742281921 0.9626607

4185.32548894082 0.9487447

4185.34355506242 0.9810946

4185.36162118402 1.016692

4185.37968730562 0.9606709

4185.39775342722 0.9405293

4185.41581954882 0.9948808

4185.43388567043 0.9780694

4185.45195179203 0.9306762

4185.47001791363 0.9197271

4185.48808403523 0.9423018

4185.50615015684 0.9827244

4185.52421627844 1.022877

4185.54228240004 0.9839664

4185.56034852164 0.9057418

4185.57841464324 0.9388585

4185.59648076485 0.9550757

4185.61454688645 0.9519411

4185.63261300805 0.9953861

4185.65067912965 1.018656

4185.66874525126 1.013944

4185.68681137286 1.007397

4185.70487749446 0.9925209

4185.72294361606 0.9716803

4185.74100973767 0.9526774

4185.75907585927 0.9441961

4185.77714198087 0.9127969

4185.79520810247 0.973684

4185.81327422407 0.9565214

4185.83134034568 0.9111846

4185.84940646728 0.9299105

4185.86747258888 0.9199711

4185.88553871048 0.9182947

4185.90360483208 0.9288357

4185.92167095369 0.9497353

4185.93973707529 0.9590557

4185.95780319689 0.9507272

4185.97586931849 0.9222201

4185.9939354401 0.9785058

4186.0120015617 0.9992177

4186.0300676833 0.9721804

4186.0481338049 0.9904261

4186.0661999265 0.9631636

4186.08426604811 0.9442415

4186.10233216971 0.9504688

4186.12039829131 0.9498309

4186.13846441291 0.923351

4186.15653053452 0.9195662

4186.17459665612 0.9725077

4186.19266277772 0.9315739

4186.21072889932 0.9864128

4186.22879502093 0.9956598

4186.24686114253 0.9477763

4186.26492726413 0.9525779

4186.28299338573 0.938457

4186.30105950733 0.9241841

4186.31912562894 0.9333789

4186.33719175054 0.9303583

4186.35525787214 0.9366331

4186.37332399374 0.980719

4186.39139011534 0.958693

4186.40945623695 0.9867911

4186.42752235855 0.9686169

4186.44558848015 0.887969

4186.46365460175 0.9129542

4186.48172072335 0.8525681

4186.49978684496 0.8089294

4186.51785296656 0.8931954

4186.53591908816 0.9459864

4186.55398520976 0.9225937

4186.57205133137 0.890565

4186.59011745297 0.9122332

4186.60818357457 0.9341239

4186.62624969617 0.9489353

4186.64431581777 0.9285433

4186.66238193938 0.9249513

4186.68044806098 0.9363509

4186.69851418258 0.8916999

4186.71658030418 0.910737

4186.73464642579 0.9019663

4186.75271254739 0.9155486

4186.77077866899 0.9267012

4186.78884479059 0.9050577

4186.80691091219 0.8708463

4186.8249770338 0.8887644

4186.8430431554 0.9528995

4186.861109277 0.9336549

4186.8791753986 0.895609

4186.89724152021 0.9384826

4186.91530764181 0.9744847

4186.93337376341 0.9477563

4186.95143988501 0.8825769

4186.96950600661 0.8734014

4186.98757212822 0.8868843

4187.00563824982 0.8313919

4187.02370437142 0.8760722

4187.04177049302 0.89354

4187.05983661462 0.8243042

4187.07790273623 0.8052001

4187.09596885783 0.8079487

4187.11403497943 0.9011835

4187.13210110103 0.9554185

4187.15016722264 0.9144206

4187.16823334424 0.8785762

4187.18629946584 0.8588479

4187.20436558744 0.8701609

4187.22243170904 0.8812712

4187.24049783065 0.9139944

4187.25856395225 0.8911317

4187.27663007385 0.9029998

4187.29469619545 0.908741

4187.31276231706 0.8972998

4187.33082843866 0.861831

4187.34889456026 0.8363174

4187.36696068186 0.8797053

4187.38502680346 0.9330822

4187.40309292507 0.881145

4187.42115904667 0.8290959

4187.43922516827 0.8651803

4187.45729128987 0.9058441

4187.47535741148 0.9152318

4187.49342353308 0.903031

4187.51148965468 0.9020692

4187.52955577628 0.9110361

4187.54762189788 0.8707456

4187.56568801949 0.8458405

4187.58375414109 0.858205

4187.60182026269 0.9029493

4187.61988638429 0.9161028

4187.6379525059 0.8727778

4187.6560186275 0.8702786

4187.6740847491 0.8552356

4187.6921508707 0.8946557

4187.71021699231 0.9399675

4187.72828311391 0.9172886

4187.74634923551 0.8671429

4187.76441535711 0.859167

4187.78248147871 0.8821679

4187.80054760032 0.8784671

4187.81861372192 0.8830824

4187.83667984352 0.8852227

4187.85474596512 0.8899767

4187.87281208672 0.8821229

4187.89087820833 0.9232557

4187.90894432993 0.9440722

4187.92701045153 0.8849066

4187.94507657313 0.8668016

4187.96314269474 0.8692236

4187.98120881634 0.8701149

4187.99927493794 0.8857903

4188.01734105954 0.8974292

4188.03540718115 0.8953257

4188.05347330275 0.9104142

4188.07153942435 0.9208886

4188.08960554595 0.8530279

4188.10767166755 0.8702835

4188.12573778916 0.921717

4188.14380391076 0.9405289

4188.16187003236 0.9204021

4188.17993615396 0.9307097

4188.19800227556 0.9332911

4188.21606839717 0.9190177

4188.23413451877 0.9162881

4188.25220064037 0.9360237

4188.27026676197 0.9111311

4188.28833288358 0.9531139

4188.30639900518 0.9361205

4188.32446512678 0.9318102

4188.34253124838 0.9372106

4188.36059736998 0.9693629

4188.37866349159 0.9773417

4188.39672961319 0.9957241

4188.41479573479 0.9879599

4188.43286185639 1.008132

4188.45092797799 1.021406

4188.4689940996 0.9933933

4188.4870602212 0.9693835

4188.5051263428 0.9778912

4188.5231924644 0.9910518

4188.54125858601 1.002909

4188.55932470761 0.9928695

4188.57739082921 0.9510376

4188.59545695081 0.9489933

4188.61352307241 0.9787639

4188.63158919402 0.9746514

4188.64965531562 0.9754962

4188.66772143722 0.9608704

4188.68578755882 0.9758544

4188.70385368043 0.9818903

4188.72191980203 0.9455041

4188.73998592363 0.9827136

4188.75805204523 1.020035

4188.77611816683 0.9823863

4188.79418428844 0.9706454

4188.81225041004 0.9926312

4188.83031653164 1.023542

4188.84838265324 0.9813998

4188.86644877485 0.9230458

4188.88451489645 0.9466863

4188.90258101805 0.939907

4188.92064713965 0.9218006

4188.93871326125 0.8970652

4188.95677938286 0.92272

4188.97484550446 0.9148777

4188.99291162606 0.9076078

4189.01097774766 0.9282175

4189.02904386927 0.8954228

4189.04710999087 0.8804331

4189.06517611247 0.8897716

4189.08324223407 0.9097663

4189.10130835567 0.94197

4189.11937447728 0.9880564

4189.13744059888 0.991

4189.15550672048 0.9301398

4189.17357284208 0.937061

4189.19163896369 0.9679169

4189.20970508529 0.908362

4189.22777120689 0.9268179

4189.24583732849 0.9885046

4189.26390345009 0.9891511

4189.2819695717 0.9062458

4189.3000356933 0.8578731

4189.3181018149 0.9247101

4189.3361679365 0.9266105

4189.3542340581 0.9139556

4189.37230017971 0.9084626

4189.39036630131 0.935445

4189.40843242291 0.9583291

4189.42649854451 0.9396191

4189.44456466612 0.9303317

4189.46263078772 0.9426603

4189.48069690932 0.9442

4189.49876303092 0.9350028

4189.51682915252 0.9663351

4189.53489527413 0.9681135

4189.55296139573 0.9477526

4189.57102751733 0.9801831

4189.58909363893 0.9818695

4189.60715976053 0.960018

4189.62522588214 0.9717829

4189.64329200374 0.9658599

4189.66135812534 0.9582229

4189.67942424694 0.9706435

4189.69749036855 0.990562

4189.71555649015 1.020981

4189.73362261175 1.006658

4189.75168873335 0.9547279

4189.76975485495 0.9629416

4189.78782097656 0.975361

4189.80588709816 0.9477216

4189.82395321976 0.9534569

4189.84201934136 0.9380873

4189.86008546297 0.9362773

4189.87815158457 0.9514344

4189.89621770617 0.9806878

4189.91428382777 0.9771463

4189.93234994938 0.9618825

4189.95041607098 0.9706486

4189.96848219258 0.978151

4189.98654831418 0.9873012

4190.00461443578 0.9379307

4190.02268055739 0.9145541

4190.04074667899 0.9690773

4190.05881280059 0.968285

4190.07687892219 0.93328

4190.09494504379 0.9157625

4190.1130111654 0.9433365

4190.131077287 0.9392208

4190.1491434086 0.8903775

4190.1672095302 0.8815938

4190.18527565181 0.9043671

4190.20334177341 0.9226862

4190.22140789501 0.9600189

4190.23947401661 0.997945

4190.25754013821 0.9656762

4190.27560625982 0.8943315

4190.29367238142 0.865556

4190.31173850302 0.871094

4190.32980462462 0.9123999

4190.34787074623 0.9363829

4190.36593686783 0.9235224

4190.38400298943 0.8958821

4190.40206911103 0.9094257

4190.42013523263 0.8948494

4190.43820135424 0.9027991

4190.45626747584 0.9373002

4190.47433359744 0.9661905

4190.49239971904 0.9858846

4190.51046584065 0.9119335

4190.52853196225 0.8906137

4190.54659808385 0.9315395

4190.56466420545 0.9648398

4190.58273032705 0.9380627

4190.60079644866 0.9083616

4190.61886257026 0.9174455

4190.63692869186 0.9438808

4190.65499481346 0.9361023

4190.67306093506 0.9327077

4190.69112705667 0.9391325

4190.70919317827 0.925261

4190.72725929987 0.9028118

4190.74532542147 0.8892217

4190.76339154308 0.8885137

4190.78145766468 0.8836489

4190.79952378628 0.8603483

4190.81758990788 0.8784301

4190.83565602948 0.9088074

4190.85372215109 0.9075445

4190.87178827269 0.8785092

4190.88985439429 0.900172

4190.90792051589 0.8920596

4190.9259866375 0.8418962

4190.9440527591 0.8045255

4190.9621188807 0.8412201

4190.9801850023 0.8876674

4190.99825112391 0.856214

4191.01631724551 0.8538742

4191.03438336711 0.9167018

4191.05244948871 0.9080962

4191.07051561031 0.8778551

4191.08858173192 0.8710672

4191.10664785352 0.8873751

4191.12471397512 0.8693769

4191.14278009672 0.8624651

4191.16084621832 0.9095038

4191.17891233993 0.9114292

4191.19697846153 0.8928316

4191.21504458313 0.8911952

4191.23311070473 0.9278911

4191.25117682634 0.9752905

4191.26924294794 0.9363835

4191.28730906954 0.9143184

4191.30537519114 0.9866121

4191.32344131274 1.010807

4191.34150743435 0.9504468

4191.35957355595 0.952052

4191.37763967755 0.952137

4191.39570579915 0.9784708

4191.41377192076 0.9708663

4191.43183804236 0.9656807

4191.44990416396 0.9833608

4191.46797028556 1.000273

4191.48603640716 1.026641

4191.50410252877 0.9895251

4191.52216865037 0.9926476

4191.54023477197 0.9891487

4191.55830089357 0.9904043

4191.57636701518 1.004065

4191.59443313678 1.046407

4191.61249925838 1.021989

4191.63056537998 0.9963707

4191.64863150158 1.004772

4191.66669762319 1.011234

4191.68476374479 1.005094

4191.70282986639 0.9830193

4191.72089598799 0.9758945

4191.73896210959 0.9390932

4191.7570282312 0.9650308

4191.7750943528 1.037236

4191.7931604744 1.032877

4191.811226596 0.9898344

4191.8292927176 0.9597917

4191.84735883921 0.9817957

4191.86542496081 1.016122

4191.88349108241 0.9964778

4191.90155720401 0.9854421

4191.91962332562 0.9855641

4191.93768944722 0.9807767

4191.95575556882 1.031106

4191.97382169042 1.020534

4191.99188781202 0.9794801

4192.00995393363 1.018579

4192.02802005523 1.042819

4192.04608617683 1.034761

4192.06415229843 1.001431

4192.08221842004 0.9796445

4192.10028454164 0.9980183

4192.11835066324 0.9966844

4192.13641678484 1.003695

4192.15448290645 1.017149

4192.17254902805 0.9879028

4192.19061514965 0.995666

4192.20868127125 0.9736017

4192.22674739285 0.9537197

4192.24481351446 0.9487914

4192.26287963606 0.9973088

4192.28094575766 1.016986

4192.29901187926 1.006146

4192.31707800086 0.9892724

4192.33514412247 1.041616

4192.35321024407 1.035269

4192.37127636567 1.011345

4192.38934248727 0.9591655

4192.40740860888 0.9510227

4192.42547473048 0.9855154

4192.44354085208 0.9709071

4192.46160697368 1.006072

4192.47967309529 1.057142

4192.49773921689 1.015536

4192.51580533849 0.9787329

4192.53387146009 0.9783539

4192.55193758169 1.048169

4192.5700037033 1.109834

4192.5880698249 1.025107

4192.6061359465 1.01634

4192.6242020681 1.024922

4192.6422681897 0.9749776

4192.66033431131 0.9671394

4192.67840043291 0.9667554

4192.69646655451 0.9850476

4192.71453267611 1.024213

4192.73259879772 1.014762

4192.75066491932 1.018311

4192.76873104092 1.046609

4192.78679716252 1.070379

4192.80486328412 1.080524

4192.82292940573 1.060056

4192.84099552733 1.032128

4192.85906164893 1.048393

4192.87712777053 1.051823

4192.89519389214 1.005055

4192.91326001374 1.030359

4192.93132613534 1.006322

4192.94939225694 1.038067

4192.96745837855 1.02892

4192.98552450015 1.021596

4193.00359062175 1.003944

4193.02165674335 1.004616

4193.03972286495 1.032344

4193.05778898656 1.010963

4193.07585510816 0.9274254

4193.09392122976 0.9542475

4193.11198735136 0.9583185

4193.13005347297 0.9946672

4193.14811959457 0.9540547

4193.16618571617 0.9524259

4193.18425183777 0.9430101

4193.20231795937 0.9394163

4193.22038408098 0.9859223

4193.23845020258 1.042718

4193.25651632418 1.008075

4193.27458244578 1.016418

4193.29264856738 1.025899

4193.31071468899 1.017239

4193.32878081059 0.9782538

4193.34684693219 0.9546024

4193.36491305379 0.982279

4193.3829791754 0.976571

4193.401045297 0.9042167

4193.4191114186 0.9341463

4193.4371775402 1.018045

4193.4552436618 1.013503

4193.47330978341 0.9997766

4193.49137590501 0.9905643

4193.50944202661 0.993793

4193.52750814821 0.9730989

4193.54557426981 0.9560893

4193.56364039142 0.9566087

4193.58170651302 0.9823447

4193.59977263462 0.9633387

4193.61783875622 0.9509312

4193.63590487783 0.9615595

4193.65397099943 0.9778488

4193.67203712103 0.9897414

4193.69010324263 0.9960859

4193.70816936423 0.9544684

4193.72623548584 0.9085609

4193.74430160744 0.9561585

4193.76236772904 1.007245

4193.78043385064 0.9717435

4193.79849997225 0.9715044

4193.81656609385 1.0083

4193.83463221545 1.027281

4193.85269833705 1.002052

4193.87076445865 0.9599769

4193.88883058026 0.9255437

4193.90689670186 0.9421464

4193.92496282346 0.9919075

4193.94302894506 1.005419

4193.96109506666 0.9585993

4193.97916118827 0.9360025

4193.99722730987 0.9855001

4194.01529343147 0.9857616

4194.03335955307 0.928371

4194.05142567468 0.9007152

4194.06949179628 0.9359366

4194.08755791788 0.9559224

4194.10562403948 0.9357073

4194.12369016109 0.9559767

4194.14175628269 0.9930351

4194.15982240429 0.9888053

4194.17788852589 0.9531454

4194.19595464749 0.9924755

4194.2140207691 0.935114

4194.2320868907 0.9329875

4194.2501530123 0.9822569

4194.2682191339 0.9437967

4194.2862852555 0.9011959

4194.30435137711 0.9326037

4194.32241749871 0.9653511

4194.34048362031 0.9307216

4194.35854974191 0.9296997

4194.37661586352 0.9760984

4194.39468198512 0.9626192

4194.41274810672 0.9262809

4194.43081422832 0.9056548

4194.44888034992 0.9362931

4194.46694647153 0.9850197

4194.48501259313 0.9886602

4194.50307871473 0.9479752

4194.52114483633 0.9966297

4194.53921095794 1.066187

4194.55727707954 1.043982

4194.57534320114 1.028448

4194.59340932274 1.025197

4194.61147544434 0.9711319

4194.62954156595 0.9546371

4194.64760768755 0.9613966

4194.66567380915 0.9860263

4194.68373993075 1.035559

4194.70180605236 0.9725611

4194.71987217396 0.9541267

4194.73793829556 0.9943322

4194.75600441716 1.042823

4194.77407053876 0.9924952

4194.79213666037 0.9487441

4194.81020278197 1.006175

4194.82826890357 1.018954

4194.84633502517 0.9817257

4194.86440114677 0.9719657

4194.88246726838 0.9563003

4194.90053338998 0.9523641

4194.91859951158 1.016194

4194.93666563318 0.9831789

4194.95473175479 0.9801414

4194.97279787639 0.9941602

4194.99086399799 0.9823813

4195.00893011959 0.9547353

4195.02699624119 0.972324

4195.0450623628 0.9965652

4195.0631284844 1.002355

4195.081194606 0.9289836

4195.0992607276 0.9387734

4195.11732684921 0.9903882

4195.13539297081 0.9904191

4195.15345909241 0.9445242

4195.17152521401 0.9431287

4195.18959133561 0.987404

4195.20765745722 0.9721389

4195.22572357882 0.9277142

4195.24378970042 0.9009713

4195.26185582202 0.9543694

4195.27992194363 0.9578238

4195.29798806523 0.9338704

4195.31605418683 0.9627573

4195.33412030843 0.9779087

4195.35218643004 0.9304561

4195.37025255164 0.9133607

4195.38831867324 0.9258384

4195.40638479484 0.9601665

4195.42445091644 0.9863607

4195.44251703804 0.9644227

4195.46058315965 0.944779

4195.47864928125 0.9830821

4195.49671540285 0.9983087

4195.51478152445 0.9893575

4195.53284764606 0.9722037

4195.55091376766 0.9291097

4195.56897988926 0.8916683

4195.58704601086 0.9179609

4195.60511213246 0.9064299

4195.62317825407 0.9309061

4195.64124437567 0.9199387

4195.65931049727 0.9163235

4195.67737661887 0.971903

4195.69544274048 0.9787958

4195.71350886208 0.9689758

4195.73157498368 0.9380738

4195.74964110528 0.9311249

4195.76770722689 0.9488009

4195.78577334849 0.8966272

4195.80383947009 0.8690022

4195.82190559169 0.9101454

4195.83997171329 0.9640117

4195.8580378349 0.9583975

4195.8761039565 0.9440579

4195.8941700781 0.9405866

4195.9122361997 0.935109

4195.93030232131 0.912449

4195.94836844291 0.8885539

4195.96643456451 0.9208504

4195.98450068611 0.9828858

4196.00256680771 0.9576939

4196.02063292932 0.9268294

4196.03869905092 0.9466443

4196.05676517252 0.9630606

4196.07483129412 0.9550797

4196.09289741572 0.9516892

4196.11096353733 1.011136

4196.12902965893 1.042788

4196.14709578053 0.9580563

4196.16516190213 0.9051351

4196.18322802374 0.9226328

4196.20129414534 0.9196982

4196.21936026694 0.926183

4196.23742638854 0.9719821

4196.25549251014 0.9306389

4196.27355863175 0.9029721

4196.29162475335 0.9388182

4196.30969087495 0.9290968

4196.32775699655 0.9078939

4196.34582311816 0.9203305

4196.36388923976 0.9281846

4196.38195536136 0.9168983

4196.40002148296 0.9328425

4196.41808760456 0.9469811

4196.43615372617 0.9525776

4196.45421984777 0.9956564

4196.47228596937 0.970928

4196.49035209097 0.9671597

4196.50841821258 0.9814796

4196.52648433418 0.9519226

4196.54455045578 0.9172949

4196.56261657738 0.9418509

4196.58068269898 0.9689498

4196.59874882059 0.9701535

4196.61681494219 0.9868526

4196.63488106379 1.010278

4196.65294718539 0.9916787

4196.671013307 0.9811172

4196.6890794286 0.9709377

4196.7071455502 0.9432253

4196.7252116718 0.9507445

4196.7432777934 0.9933395

4196.76134391501 1.013987

4196.77941003661 1.0296

4196.79747615821 1.066308

4196.81554227981 1.022827

4196.83360840141 0.9858267

4196.85167452302 1.003933

4196.86974064462 0.9634064

4196.88780676622 0.9887695

4196.90587288782 1.007374

4196.92393900943 0.9845319

4196.94200513103 0.999718

4196.96007125263 1.025416

4196.97813737423 1.012758

4196.99620349583 0.9832265

4197.01426961744 1.006275

4197.03233573904 0.9968419

4197.05040186064 0.9860321

4197.06846798224 1.000518

4197.08653410384 0.9710131

4197.10460022545 0.9452429

4197.12266634705 0.9290746

4197.14073246865 0.9648076

4197.15879859025 0.9265944

4197.17686471186 0.9257494

4197.19493083346 0.9767482

4197.21299695506 1.002178

4197.23106307666 1.033398

4197.24912919827 1.045281

4197.26719531987 1.007838

4197.28526144147 0.9848776

4197.30332756307 0.9811409

4197.32139368467 0.9288536

4197.33945980628 0.9332242

4197.35752592788 0.9913304

4197.37559204948 0.9819031

4197.39365817108 0.9893647

4197.41172429269 0.9757875

4197.42979041429 0.9869322

4197.44785653589 0.9952766

4197.46592265749 1.003474

4197.48398877909 0.9549184

4197.5020549007 0.9311954

4197.5201210223 0.9429325

4197.5381871439 0.9285874

4197.5562532655 0.9292643

4197.57431938711 0.9408275

4197.59238550871 0.9508139

4197.61045163031 0.9628528

4197.62851775191 0.9670255

4197.64658387351 0.8971288

4197.66464999512 0.9492208

4197.68271611672 0.9684815

4197.70078223832 0.9020215

4197.71884835992 0.8921399

4197.73691448153 0.9445513

4197.75498060313 0.981855

4197.77304672473 0.9222036

4197.79111284633 0.9430764

4197.80917896793 0.9080915

4197.82724508953 0.8948437

4197.84531121114 0.9241974

4197.86337733274 0.888968

4197.88144345434 0.8656229

4197.89950957594 0.8539451

4197.91757569755 0.858043

4197.93564181915 0.9146589

4197.95370794075 0.9318174

4197.97177406235 0.9033442

4197.98984018396 0.9293365

4198.00790630556 0.8744938

4198.02597242716 0.841579

4198.04403854876 0.888056

4198.06210467036 0.9083372

4198.08017079197 0.953904

4198.09823691357 0.9660295

4198.11630303517 0.9306121

4198.13436915677 0.9442875

4198.15243527838 0.887434

4198.17050139998 0.8926241

4198.18856752158 0.9369686

4198.20663364318 0.8893248

4198.22469976478 0.9278815

4198.24276588639 0.9721438

4198.26083200799 0.9170181

4198.27889812959 0.8654867

4198.29696425119 0.8199539

4198.3150303728 0.8237928

4198.3330964944 0.8636745

4198.351162616 0.8651324

4198.3692287376 0.8874385

4198.3872948592 0.9234331

4198.40536098081 0.8845022

4198.42342710241 0.8163357

4198.44149322401 0.7814245

4198.45955934561 0.7961857

4198.47762546721 0.8677844

4198.49569158882 0.9029601

4198.51375771042 0.8482534

4198.53182383202 0.8148685

4198.54988995362 0.8492705

4198.56795607523 0.8811485

4198.58602219683 0.8486309

4198.60408831843 0.842194

4198.62215444003 0.8328257

4198.64022056163 0.7986668

4198.65828668324 0.8074137

4198.67635280484 0.8294262

4198.69441892644 0.8058696

4198.71248504804 0.8091339

4198.73055116964 0.8411716

4198.74861729125 0.8233542

4198.76668341285 0.8259491

4198.78474953445 0.898845

4198.80281565605 0.9143311

4198.82088177766 0.9208718

4198.83894789926 0.8934082

4198.85701402086 0.8556247

4198.87508014246 0.8587325

4198.89314626407 0.857241

4198.91121238567 0.8914777

4198.92927850727 0.8841366

4198.94734462887 0.8669571

4198.96541075047 0.9222275

4198.98347687207 0.9575338

4199.00154299368 0.9747908

4199.01960911528 0.9451583

4199.03767523688 0.9233148

4199.05574135848 0.9288708

4199.07380748009 0.9447398

4199.09187360169 0.9519318

4199.10993972329 0.9645758

4199.12800584489 0.9488884

4199.1460719665 0.9528823

4199.1641380881 0.9828575

4199.1822042097 0.9672247

4199.2002703313 0.9340017

4199.2183364529 0.9460253

4199.23640257451 0.9140193

4199.25446869611 0.8957027

4199.27253481771 0.9245369

4199.29060093931 0.9057701

4199.30866706092 0.9350049

4199.32673318252 0.9446131

4199.34479930412 0.9148166

4199.36286542572 0.9118217

4199.38093154732 0.9365451

4199.39899766893 0.9029639

4199.41706379053 0.9342468

4199.43512991213 0.8889314

4199.45319603373 0.8881046

4199.47126215534 0.9384088

4199.48932827694 0.9445469

4199.50739439854 0.898955

4199.52546052014 0.861055

4199.54352664175 0.8909763

4199.56159276335 0.8926095

4199.57965888495 0.8541106

4199.59772500655 0.909271

4199.61579112815 0.9000045

4199.63385724975 0.8389333

4199.65192337136 0.8634621

4199.66998949296 0.8732222

4199.68805561456 0.8580464

4199.70612173616 0.8886184

4199.72418785777 0.8845767

4199.74225397937 0.8939816

4199.76032010097 0.9344043

4199.77838622257 0.8876297

4199.79645234418 0.8988758

4199.81451846578 0.894577

4199.83258458738 0.8675719

4199.85065070898 0.8939393

4199.86871683058 0.8926338

4199.88678295219 0.8528409

4199.90484907379 0.9011413

4199.92291519539 0.9439795

4199.94098131699 0.9635406

4199.9590474386 0.9320259

4199.9771135602 0.8897808

4199.9951796818 0.9614027

4200.0132458034 0.9780569

4200.031311925 0.9175844

4200.0493780466 0.9456416

4200.06744416821 0.9254474

4200.08551028981 0.9131449

4200.10357641141 0.9759768

4200.12164253301 0.9283434

4200.13970865462 0.9140934

4200.15777477622 0.9569306

4200.17584089782 0.9486015

4200.19390701942 0.8753992

4200.21197314102 0.881246

4200.23003926263 0.9602817

4200.24810538423 0.9904704

4200.26617150583 0.9511924

4200.28423762744 0.9172361

4200.30230374904 0.8818998

4200.32036987064 0.8926247

4200.33843599224 0.8931175

4200.35650211384 0.8902703

4200.37456823545 0.927106

4200.39263435705 0.9091029

4200.41070047865 0.87957

4200.42876660025 0.8809248

4200.44683272185 0.8841655

4200.46489884346 0.8985088

4200.48296496506 0.8531804

4200.50103108666 0.8421941

4200.51909720826 0.9182683

4200.53716332987 0.9059484

4200.55522945147 0.8233431

4200.57329557307 0.8059272

4200.59136169467 0.8079609

4200.60942781627 0.7545307

4200.62749393788 0.7214732

4200.64556005948 0.7817363

4200.66362618108 0.8141828

4200.68169230268 0.8136072

4200.69975842429 0.78065

4200.71782454589 0.8027786

4200.73589066749 0.8315047

4200.75395678909 0.8223146

4200.77202291069 0.7615336

4200.7900890323 0.725538

4200.8081551539 0.766547

4200.8262212755 0.7905606

4200.8442873971 0.8003813

4200.8623535187 0.784823

4200.88041964031 0.7627214

4200.89848576191 0.7182523

4200.91655188351 0.7427109

4200.93461800511 0.798537

4200.95268412672 0.8554607

4200.97075024832 0.8208815

4200.98881636992 0.7614082

4201.00688249152 0.7752905

4201.02494861312 0.7950056

4201.04301473473 0.7784274

4201.06108085633 0.7456489

4201.07914697793 0.7415215

4201.09721309953 0.8179683

4201.11527922114 0.8535436

4201.13334534274 0.8283651

4201.15141146434 0.8570772

4201.16947758594 0.8609239

4201.18754370754 0.8265133

4201.20560982915 0.8881118

4201.22367595075 0.8084809

4201.24174207235 0.8199034

4201.25980819395 0.8383335

4201.27787431556 0.8183179

4201.29594043716 0.8348083

4201.31400655876 0.8537399

4201.33207268036 0.8194683

4201.35013880196 0.8532351

4201.36820492357 0.9132757

4201.38627104517 0.9344206

4201.40433716677 0.8981932

4201.42240328837 0.8962208

4201.44046940998 0.9192655

4201.45853553158 0.9774361

4201.47660165318 0.9821656

4201.49466777478 1.002997

4201.51273389638 1.003084

4201.53080001799 1.001852

4201.54886613959 0.9480388

4201.56693226119 0.9705494

4201.58499838279 1.027288

4201.6030645044 0.971533

4201.621130626 0.9284335

4201.6391967476 0.9552433

4201.6572628692 0.9742516

4201.6753289908 0.9969038

4201.69339511241 0.9016038

4201.71146123401 0.9092429

4201.72952735561 0.9757125

4201.74759347721 0.9174556

4201.76565959881 0.9556881

4201.78372572042 0.9631509

4201.80179184202 0.9334961

4201.81985796362 0.9016838

4201.83792408522 0.9519872

4201.85599020683 0.9871833

4201.87405632843 0.9384006

4201.89212245003 0.8498962

4201.91018857163 0.8677365

4201.92825469323 0.9522408

4201.94632081484 1.009316

4201.96438693644 1.019672

4201.98245305804 0.98625

4202.00051917964 0.9580473

4202.01858530124 0.9347371

4202.03665142285 0.9217904

4202.05471754445 0.9448234

4202.07278366605 0.9456341

4202.09084978765 0.9623567

4202.10891590926 0.9710054

4202.12698203086 1.006816

4202.14504815246 0.9402595

4202.16311427406 0.9486261

4202.18118039566 0.9240869

4202.19924651727 0.8847263

4202.21731263887 0.8736954

4202.23537876047 0.8498549

4202.25344488207 0.8606446

4202.27151100368 0.9932673

4202.28957712528 0.947148

4202.30764324688 0.8501538

4202.32570936848 0.8659576

4202.34377549009 0.8568068

4202.36184161169 0.8584572

4202.37990773329 0.8970243

4202.39797385489 0.8716551

4202.41603997649 0.8704378

4202.4341060981 0.8880478

4202.4521722197 0.8904051

4202.4702383413 0.8744093

4202.4883044629 0.8815005

4202.50637058451 0.8785885

4202.52443670611 0.9523709

4202.54250282771 0.9278612

4202.56056894931 0.8666583

4202.57863507091 0.9066556

4202.59670119252 0.9269294

4202.61476731412 0.9041084

4202.63283343572 0.9280256

4202.65089955732 0.9725648

4202.66896567892 0.8810781

4202.68703180053 0.8655077

4202.70509792213 1.023816

4202.72316404373 0.9389929

4202.74123016533 0.8848777

4202.75929628694 1.000768

4202.77736240854 0.9499858

4202.79542853014 0.8297635

4202.81349465174 0.8274781

4202.83156077334 0.9034383

4202.84962689495 0.8974108

4202.86769301655 0.8448637

4202.88575913815 0.8411953

4202.90382525975 0.8600445

4202.92189138136 0.9148296

4202.93995750296 0.9433624

4202.95802362456 0.9265637

4202.97608974616 0.8974623

4202.99415586776 0.8584453

4203.01222198937 0.8686647

4203.03028811097 0.8736615

4203.04835423257 0.8741693

4203.06642035417 0.914041

4203.08448647578 0.9237795

4203.10255259738 0.9157126

4203.12061871898 0.8880523

4203.13868484058 0.9520639

4203.15675096218 0.8932278

4203.17481708379 0.8758793

4203.19288320539 0.9456018

4203.21094932699 0.8741006

4203.22901544859 0.9111027

4203.24708157019 0.9487047

4203.2651476918 0.8642401

4203.2832138134 0.8912307

4203.301279935 0.9582268

4203.3193460566 0.9273711

4203.33741217821 0.8359548

4203.35547829981 0.8319151

4203.37354442141 0.8876047

4203.39161054301 0.9517965

4203.40967666461 0.970417

4203.42774278622 0.9114302

4203.44580890782 0.9061022

4203.46387502942 0.9282184

4203.48194115102 0.9111396

4203.50000727263 0.873016

4203.51807339423 0.8950304

4203.53613951583 0.8861941

4203.55420563743 0.895746

4203.57227175903 0.9061475

4203.59033788064 0.9070904

4203.60840400224 0.9001367

4203.62647012384 0.9113791

4203.64453624544 0.9529276

4203.66260236705 0.9627979

4203.68066848865 0.9273679

4203.69873461025 0.970896

4203.71680073185 1.005625

4203.73486685345 1.013755

4203.75293297506 0.966605

4203.77099909666 1.004311

4203.78906521826 1.033033

4203.80713133986 0.9324582

4203.82519746146 0.9160838

4203.84326358307 0.9888476

4203.86132970467 0.9732875

4203.87939582627 1.003048

4203.89746194787 0.9171501

4203.91552806948 0.8152577

4203.93359419108 0.8656785

4203.95166031268 0.9833254

4203.96972643428 0.9446115

4203.98779255588 1.042284

4204.00585867749 0.9162325

4204.02392479909 0.8966999

4204.04199092069 0.9790825

4204.06005704229 0.9511814

4204.0781231639 0.871543

4204.0961892855 0.897642

4204.1142554071 0.8869988

4204.1323215287 0.87799

4204.15038765031 0.9702824

4204.16845377191 0.9968725

4204.18651989351 0.9311575

4204.20458601511 0.9769363

4204.22265213671 0.9892269

4204.24071825831 1.020985

4204.25878437992 1.074555

4204.27685050152 1.060574

4204.29491662312 1.084885

4204.31298274472 1.11322

4204.33104886633 1.062116

4204.34911498793 0.9945492

4204.36718110953 0.9500128

4204.38524723113 0.9897227

4204.40331335273 1.000681

4204.42137947434 0.8696647

4204.43944559594 0.8661007

4204.45751171754 0.9554858

4204.47557783915 0.9940557

4204.49364396075 0.9289981

4204.51171008235 0.8628777

4204.52977620395 1.012188

4204.54784232555 1.030101

4204.56590844716 0.9622552

4204.58397456876 0.9223592

4204.60204069036 0.9926765

4204.62010681196 0.9618406

4204.63817293356 0.8868643

4204.65623905517 0.9179924

4204.67430517677 0.9825433

4204.69237129837 0.9487021

4204.71043741997 0.9353142

4204.72850354158 0.9373521

4204.74656966318 0.9006773

4204.76463578478 0.9490942

4204.78270190638 0.9673333

4204.80076802798 0.9616064

4204.81883414959 0.8825848

4204.83690027119 0.8978689

4204.85496639279 0.9067993

4204.87303251439 0.8485708

4204.891098636 0.8303752

4204.9091647576 0.8935511

4204.9272308792 0.9607313

4204.9452970008 0.9051906

4204.9633631224 0.8129837

4204.98142924401 0.8005086

4204.99949536561 0.7237179

4205.01756148721 0.6921078

4205.03562760881 0.7639123

4205.05369373042 0.6895663

4205.07175985202 0.6922312

4205.08982597362 0.8471814

4205.10789209522 0.8337137

4205.12595821682 0.7740169

4205.14402433843 0.7749323

4205.16209046003 0.7620027

4205.18015658163 0.7830963

4205.19822270323 0.7908173

4205.21628882483 0.7847844

4205.23435494644 0.7565352

4205.25242106804 0.7734263

4205.27048718964 0.8204232

4205.28855331124 0.7918676

4205.30661943285 0.7549562

4205.32468555445 0.7544802

4205.34275167605 0.7781481

4205.36081779765 0.7932295

4205.37888391925 0.8788242

4205.39695004086 0.8778179

4205.41501616246 0.8511758

4205.43308228406 0.8223175

4205.45114840566 0.7180886

4205.46921452727 0.7251687

4205.48728064887 0.8185902

4205.50534677047 0.8418318

4205.52341289207 0.8553507

4205.54147901367 0.8705364

4205.55954513528 0.878721

4205.57761125688 0.8993869

4205.59567737848 0.904532

4205.61374350008 0.8557837

4205.63180962169 0.7837052

4205.64987574329 0.777104

4205.66794186489 0.8250616

4205.68600798649 0.8688246

4205.70407410809 0.9131755

4205.7221402297 0.9313663

4205.7402063513 0.7800461

4205.7582724729 0.8253968

4205.7763385945 0.8607688

4205.7944047161 0.8517475

4205.81247083771 0.8746824

4205.83053695931 0.923954

4205.84860308091 0.9450696

4205.86666920251 0.8585114

4205.88473532411 0.8553318

4205.90280144572 0.8355942

4205.92086756732 0.8805686

4205.93893368892 0.907514

4205.95699981052 0.915517

4205.97506593213 0.8790244

4205.99313205373 0.8854806

4206.01119817533 0.9728275

4206.02926429693 0.9678642

4206.04733041854 0.9051712

4206.06539654014 1.044787

4206.08346266174 0.8827553

4206.10152878334 0.8592122

4206.11959490494 0.9873059

4206.13766102655 0.942102

4206.15572714815 0.9604819

4206.17379326975 1.001834

4206.19185939135 0.9444619

4206.20992551295 0.9304543

4206.22799163456 0.9572926

4206.24605775616 1.018462

4206.26412387776 0.9842619

4206.28218999936 1.01751

4206.30025612097 0.9969748

4206.31832224257 0.8454852

4206.33638836417 0.7593929

4206.35445448577 0.8255063

4206.37252060738 0.9808489

4206.39058672898 0.9325975

4206.40865285058 0.7939857

4206.42671897218 0.8109454

4206.44478509378 0.8662877

4206.46285121539 0.9561234

4206.48091733699 0.9254188

4206.49898345859 0.8371563

4206.51704958019 0.860775

4206.5351157018 0.8613377

4206.5531818234 1.000337

4206.571247945 1.005991

4206.5893140666 0.8591

4206.6073801882 0.8494124

4206.62544630981 0.8573102

4206.64351243141 0.9024628

4206.66157855301 0.8612233

4206.67964467461 0.8488123

4206.69771079622 0.8795612

4206.71577691782 0.9672676

4206.73384303942 0.9600381

4206.75190916102 0.9126897

4206.76997528262 0.8625886

4206.78804140423 0.9339864

4206.80610752583 1.039

4206.82417364743 1.074762

4206.84223976903 1.015728

4206.86030589063 0.9265313

4206.87837201224 0.9330897

4206.89643813384 1.034431

4206.91450425544 0.9514785

4206.93257037704 0.8534184

4206.95063649865 0.9016985

4206.96870262025 1.011687

4206.98676874185 0.9985006

4207.00483486345 0.9223662

4207.02290098505 0.932861

4207.04096710666 0.989365

4207.05903322826 0.9607354

4207.07709934986 0.9976403

4207.09516547146 1.054193

4207.11323159307 1.03658

4207.13129771467 0.9311651

4207.14936383627 0.8280045

4207.16742995787 0.9184716

4207.18549607947 0.945946

4207.20356220108 0.9417441

4207.22162832268 0.951115

4207.23969444428 0.9428632

4207.25776056588 0.9595511

4207.27582668749 0.9365928

4207.29389280909 0.9011028

4207.31195893069 0.9525048

4207.33002505229 1.043969

4207.34809117389 0.948787

4207.3661572955 0.8449396

4207.3842234171 0.9505953

4207.4022895387 0.991725

4207.4203556603 0.9966479

4207.4384217819 1.038866

4207.45648790351 0.9710783

4207.47455402511 0.9098568

4207.49262014671 0.9221177

4207.51068626831 0.9375305

4207.52875238992 0.9579606

4207.54681851152 0.973237

4207.56488463312 0.9975542

4207.58295075472 0.9122665

4207.60101687633 0.7924067

4207.61908299793 0.7855201

4207.63714911953 0.7746817

4207.65521524113 0.9622416

4207.67328136273 1.02993

4207.69134748434 0.9054601

4207.70941360594 0.879696

4207.72747972754 0.8543065

4207.74554584914 0.8342941

4207.76361197074 0.9004692

4207.78167809235 0.9183983

4207.79974421395 0.9405004

4207.81781033555 0.9050785

4207.83587645715 0.8460175

4207.85394257875 0.9030806

4207.87200870036 0.9061597

4207.89007482196 0.8521327

4207.90814094356 0.8491074

4207.92620706516 0.9127347

4207.94427318677 0.9170986

4207.96233930837 0.8275056

4207.98040542997 0.941167

4207.99847155157 0.947803

4208.01653767317 0.8979006

4208.03460379478 0.9248375

4208.05266991638 0.7700415

4208.07073603798 0.8110788

4208.08880215958 0.8259851

4208.10686828119 0.8406706

4208.12493440279 0.8543953

4208.14300052439 0.8576885

4208.16106664599 0.8077682

4208.17913276759 0.8679758

4208.1971988892 0.9912257

4208.2152650108 0.9360231

4208.2333311324 0.8403504

4208.251397254 0.8169399

4208.26946337561 0.7881187

4208.28752949721 0.8486679

4208.30559561881 0.9257126

4208.32366174041 1.066454

4208.34172786202 1.037372

4208.35979398362 0.9568889

4208.37786010522 0.8665993

4208.39592622682 0.7972912

4208.41399234842 0.891791

4208.43205847002 1.050789

4208.45012459163 0.9844211

4208.46819071323 0.8476443

4208.48625683483 0.8244379

4208.50432295643 0.9727951

4208.52238907804 1.01212

4208.54045519964 0.9435046

4208.55852132124 0.9337152

4208.57658744284 0.9212753

4208.59465356444 0.9931202

4208.61271968605 0.9074345

4208.63078580765 0.8771891

4208.64885192925 0.8826215

4208.66691805085 0.9759164

4208.68498417246 1.123304

4208.70305029406 0.9262238

4208.72111641566 0.9471664

4208.73918253726 1.082653

4208.75724865887 1.00774

4208.77531478047 0.9646307

4208.79338090207 1.032823

4208.81144702367 0.931626

4208.82951314527 0.9535253

4208.84757926688 0.9079354

4208.86564538848 0.9401076

4208.88371151008 0.9445405

4208.90177763168 0.9742368

4208.91984375329 1.012

4208.93790987489 1.060501

4208.95597599649 0.9980429

4208.97404211809 0.9737099

4208.99210823969 0.9613279

4209.01017436129 0.8555324

4209.0282404829 0.898638

4209.0463066045 0.9905031

4209.0643727261 0.9326658

4209.08243884771 0.9788992

4209.10050496931 0.9869895

4209.11857109091 1.015921

4209.13663721251 1.041023

4209.15470333411 1.021186

4209.17276945572 0.9850696

4209.19083557732 0.9650968

4209.20890169892 1.012779

4209.22696782052 1.008125

4209.24503394213 0.7809714

4209.26310006373 0.7067906

4209.28116618533 0.7519553

4209.29923230693 0.7645304

4209.31729842853 0.9170841

4209.33536455014 1.009823

4209.35343067174 1.018921

4209.37149679334 0.949733

4209.38956291494 0.8826064

4209.40762903654 0.8530962

4209.42569515815 0.8661573

4209.44376127975 0.9084001

4209.46182740135 0.9853835

4209.47989352295 0.9995999

4209.49795964456 1.01388

4209.51602576616 1.028411

4209.53409188776 0.9693103

4209.55215800936 0.8855319

4209.57022413096 0.8680437

4209.58829025257 1.040609

4209.60635637417 1.102696

4209.62442249577 0.9633

4209.64248861737 0.8747519

4209.66055473898 0.8535981

4209.67862086058 0.8927571

4209.69668698218 0.8615593

4209.71475310378 0.8447409

4209.73281922538 0.8898066

4209.75088534699 0.7662444

4209.76895146859 0.7122459

4209.78701759019 0.8787049

4209.80508371179 0.8718467

4209.8231498334 0.839943

4209.841215955 1.032955

4209.8592820766 1.055905

4209.8773481982 0.8577231

4209.8954143198 0.7800456

4209.91348044141 0.8285398

4209.93154656301 0.7297143

4209.94961268461 0.875168

4209.96767880621 1.006855

4209.98574492782 0.9455968

4210.00381104942 0.965169

4210.02187717102 0.9637002

4210.03994329262 0.9186063

4210.05800941422 0.8723961

4210.07607553583 0.9613779

4210.09414165743 1.098781

4210.11220777903 0.9523015

4210.13027390063 1.04171

4210.14834002223 0.9223987

4210.16640614384 0.7590775

4210.18447226544 0.6761644

4210.20253838704 0.8752947

4210.22060450864 0.925862

4210.23867063025 0.856065

4210.25673675185 0.7472821

4210.27480287345 0.7321371

4210.29286899505 0.8241692

4210.31093511665 0.9203417

4210.32900123826 0.8962268

4210.34706735986 0.9298692

4210.36513348146 0.9457993

4210.38319960306 0.92729

4210.40126572466 0.9354361

4210.41933184627 0.9769635

4210.43739796787 0.9137787

4210.45546408947 0.8190686

4210.47353021107 0.9103151

4210.49159633268 0.8750448

4210.50966245428 0.9382836

4210.52772857588 0.9629568

4210.54579469748 0.9560433

4210.56386081908 0.9177486

4210.58192694069 0.8229685

4210.59999306229 0.7194397

4210.61805918389 0.8377141

4210.63612530549 0.9415243

4210.6541914271 0.9240896

4210.6722575487 0.8711423

4210.6903236703 0.9254752

4210.7083897919 0.8658988

4210.7264559135 0.972091

4210.74452203511 1.005919

4210.76258815671 0.9702399

4210.78065427831 0.9245272

4210.79872039991 0.9108319

4210.81678652152 0.938501

4210.83485264312 0.7297112

4210.85291876472 0.8178344

4210.87098488632 0.9506803

4210.88905100792 0.9604801

4210.90711712953 0.9186717

4210.92518325113 0.8206217

4210.94324937273 0.9234917

4210.96131549433 0.8784736

4210.97938161594 0.9281873

4210.99744773754 0.9668423

4211.01551385914 0.9725719

4211.03357998074 0.9331281

4211.05164610234 0.8878155

4211.06971222395 0.8753906

4211.08777834555 0.9164366

4211.10584446715 0.9084106

4211.12391058875 0.8736741

4211.14197671036 0.8880031

4211.16004283196 0.8821211

4211.17810895356 0.8736409

4211.19617507516 0.8793492

4211.21424119676 0.9254467

4211.23230731837 0.877562

4211.25037343997 0.8700557

4211.26843956157 0.8776361

4211.28650568317 0.9431664

4211.30457180478 1.014402

4211.32263792638 0.9532418

4211.34070404798 0.953782

4211.35877016958 0.9492867

4211.37683629118 0.9226426

4211.39490241279 0.9192954

4211.41296853439 0.9283674

4211.43103465599 0.9024679

4211.44910077759 0.8490593

4211.4671668992 0.8877552

4211.4852330208 0.9951048

4211.5032991424 0.9897634

4211.521365264 0.8981992

4211.53943138561 0.879195

4211.55749750721 0.9242895

4211.57556362881 0.97597

4211.59362975041 0.9687607

4211.61169587201 0.8536956

4211.62976199361 0.9230541

4211.64782811522 0.9871105

4211.66589423682 0.9748101

4211.68396035842 1.04052

4211.70202648002 0.9639776

4211.72009260163 0.8802216

4211.73815872323 0.8745719

4211.75622484483 0.9417765

4211.77429096643 0.93308

4211.79235708803 0.9305785

4211.81042320964 0.9453954

4211.82848933124 0.9640403

4211.84655545284 0.9391125

4211.86462157444 0.916136

4211.88268769605 1.013318

4211.90075381765 1.041595

4211.91881993925 0.9323224

4211.93688606085 0.8593482

4211.95495218245 0.8532936

4211.97301830406 0.8806209

4211.99108442566 0.9134982

4212.00915054726 0.9642522

4212.02721666886 0.9786668

4212.04528279046 0.997052

4212.06334891207 0.9524969

4212.08141503367 0.8415316

4212.09948115527 0.863646

4212.11754727687 0.9461296

4212.13561339848 0.9610436

4212.15367952008 0.8830348

4212.17174564168 0.8620716

4212.18981176328 0.9421622

4212.20787788489 0.9824612

4212.22594400649 0.9553378

4212.24401012809 0.9554062

4212.26207624969 0.974627

4212.28014237129 0.9402846

4212.2982084929 0.9485136

4212.3162746145 1.045583

4212.3343407361 0.9890112

4212.3524068577 0.8873093

4212.37047297931 0.9353592

4212.38853910091 0.9675797

4212.40660522251 0.9666865

4212.42467134411 0.9845257

4212.44273746571 0.9558409

4212.46080358732 0.88703

4212.47886970892 0.9616835

4212.49693583052 1.036689

4212.51500195212 1.076719

4212.53306807372 0.9851357

4212.55113419533 0.9927325

4212.56920031693 1.027473

4212.58726643853 0.9796323

4212.60533256013 0.9854878

4212.62339868174 1.011206

4212.64146480334 1.010154

4212.65953092494 1.027755

4212.67759704654 1.034087

4212.69566316814 0.9764816

4212.71372928975 0.9363965

4212.73179541135 0.9458864

4212.74986153295 0.9632556

4212.76792765455 0.9841596

4212.78599377615 0.9624482

4212.80405989776 0.9462689

4212.82212601936 0.9754013

4212.84019214096 0.997587

4212.85825826256 0.9904399

4212.87632438417 0.9894822

4212.89439050577 1.02325

4212.91245662737 0.9694957

4212.93052274897 0.9672965

4212.94858887058 1.033923

4212.96665499218 0.9517049

4212.98472111378 0.9552844

4213.00278723538 0.9966697

4213.02085335698 0.9651507

4213.03891947859 0.959334

4213.05698560019 0.9717399

4213.07505172179 0.9563399

4213.09311784339 0.9495111

4213.111183965 0.949192

4213.1292500866 0.9446778

4213.1473162082 0.9613736

4213.1653823298 0.9621768

4213.1834484514 0.9540972

4213.201514573 0.9403539

4213.21958069461 0.9333429

4213.23764681621 0.9731601

4213.25571293781 0.9034386

4213.27377905942 0.7810388

4213.29184518102 0.8149416

4213.30991130262 0.8322138

4213.32797742422 0.8909503

4213.34604354582 0.8757919

4213.36410966743 0.8673854

4213.38217578903 0.8991102

4213.40024191063 0.8906876

4213.41830803223 0.9080309

4213.43637415384 0.9309297

4213.45444027544 0.8912696

4213.47250639704 0.8762885

4213.49057251864 0.9310399

4213.50863864024 0.9681412

4213.52670476185 0.9328883

4213.54477088345 0.888667

4213.56283700505 0.880021

4213.58090312665 0.89082

4213.59896924825 0.9177034

4213.61703536986 0.9192711

4213.63510149146 0.9103053

4213.65316761306 0.9399931

4213.67123373466 0.9515883

4213.68929985627 0.9269822

4213.70736597787 0.9348677

4213.72543209947 0.9864866

4213.74349822107 0.9813106

4213.76156434267 0.9299107

4213.77963046428 0.9042127

4213.79769658588 0.9124603

4213.81576270748 0.8897524

4213.83382882908 0.9096947

4213.85189495069 0.9688453

4213.86996107229 1.006523

4213.88802719389 0.9843569

4213.90609331549 0.960251

4213.92415943709 0.9890286

4213.9422255587 0.958932

4213.9602916803 0.9296308

4213.9783578019 0.9179814

4213.9964239235 0.8970516

4214.0144900451 0.9007372

4214.03255616671 0.9078673

4214.05062228831 0.9290694

4214.06868840991 0.9587359

4214.08675453151 0.9617156

4214.10482065312 0.9400421

4214.12288677472 0.9047623

4214.14095289632 0.9087317

4214.15901901792 0.9523906

4214.17708513953 0.9612336

4214.19515126113 0.9093208

4214.21321738273 0.9279425

4214.23128350433 0.928681

4214.24934962593 0.8960395

4214.26741574754 0.9176148

4214.28548186914 0.9050763

4214.30354799074 0.8655127

4214.32161411234 0.906833

4214.33968023394 0.9809057

4214.35774635555 0.953981

4214.37581247715 0.9160036

4214.39387859875 0.9601648

4214.41194472035 0.984741

4214.43001084196 0.9891942

4214.44807696356 0.9448959

4214.46614308516 0.9084511

4214.48420920676 0.933934

4214.50227532837 0.9468532

4214.52034144997 0.9652988

4214.53840757157 0.9558532

4214.55647369317 0.9346349

4214.57453981477 0.9469452

4214.59260593638 0.9819638

4214.61067205798 0.9772594

4214.62873817958 0.95855

4214.64680430118 0.9763668

4214.66487042278 0.9935935

4214.68293654439 0.9901378

4214.70100266599 0.9772775

4214.71906878759 0.9513761

4214.73713490919 0.9293633

4214.7552010308 0.9871478

4214.7732671524 0.9689651

4214.791333274 0.9105968

4214.8093993956 0.9279656

4214.8274655172 0.9329168

4214.84553163881 0.9349731

4214.86359776041 0.9617129

4214.88166388201 0.9461596

4214.89973000361 0.9210744

4214.91779612521 0.9390694

4214.93586224682 0.9359182

4214.95392836842 0.930855

4214.97199449002 0.9212496

4214.99006061162 0.9287006

4215.00812673323 0.9712429

4215.02619285483 0.9426513

4215.04425897643 0.9021342

4215.06232509803 0.8861892

4215.08039121963 0.8529425

4215.09845734124 0.8945028

4215.11652346284 0.9563456

4215.13458958444 0.9211839

4215.15265570604 0.8952549

4215.17072182765 0.9258196

4215.18878794925 0.9625981

4215.20685407085 0.955986

4215.22492019245 0.9564098

4215.24298631405 0.9690514

4215.26105243566 0.9296108

4215.27911855726 0.9260741

4215.29718467886 0.9299653

4215.31525080046 0.8582809

4215.33331692207 0.8771235

4215.35138304367 0.8858231

4215.36944916527 0.8694302

4215.38751528687 0.8828496

4215.40558140847 0.8828757

4215.42364753008 0.8721076

4215.44171365168 0.8494015

4215.45977977328 0.8330883

4215.47784589488 0.8340035

4215.49591201648 0.8739245

4215.51397813809 0.8400896

4215.53204425969 0.7777361

4215.55011038129 0.7702489

4215.56817650289 0.7720473

4215.5862426245 0.7847329

4215.6043087461 0.7775325

4215.6223748677 0.7337375

4215.6404409893 0.6865075

4215.65850711091 0.7068326

4215.67657323251 0.713047

4215.69463935411 0.7458153

4215.71270547571 0.7902957

4215.73077159731 0.781

4215.74883771891 0.7847717

4215.76690384052 0.791006

4215.78496996212 0.78422

4215.80303608372 0.7904372

4215.82110220532 0.8248169

4215.83916832693 0.8484383

4215.85723444853 0.8199066

4215.87530057013 0.8063411

4215.89336669173 0.8162646

4215.91143281334 0.8416044

4215.92949893494 0.850192

4215.94756505654 0.8577724

4215.96563117814 0.8732618

4215.98369729974 0.8673922

4216.00176342135 0.8359779

4216.01982954295 0.8558723

4216.03789566455 0.897892

4216.05596178615 0.9146957

4216.07402790776 0.87756

4216.09209402936 0.8334596

4216.11016015096 0.932902

4216.12822627256 0.9707202

4216.14629239416 0.9784747

4216.16435851577 0.959435

4216.18242463737 1.008312

4216.20049075897 0.974729

4216.21855688057 0.9927477

4216.23662300217 0.9933602

4216.25468912378 0.9999626

4216.27275524538 0.934298

4216.29082136698 0.9674089

4216.30888748858 1.031436

4216.32695361019 1.00661

4216.34501973179 1.00322

4216.36308585339 1.009445

4216.38115197499 0.9800459

4216.3992180966 0.9864031

4216.4172842182 1.013665

4216.4353503398 1.005241

4216.4534164614 1.026038

4216.47148258301 1.007257

4216.48954870461 1.0113

4216.50761482621 1.010831

4216.52568094781 0.9700605

4216.54374706941 1.000069

4216.56181319102 1.035228

4216.57987931262 1.050815

4216.59794543422 1.031358

4216.61601155582 0.9977816

4216.63407767742 0.9740275

4216.65214379903 0.9676661

4216.67020992063 0.9887832

4216.68827604223 1.046971

4216.70634216383 1.037551

4216.72440828544 0.9809523

4216.74247440704 1.008471

4216.76054052864 1.024498

4216.77860665024 0.9732283

4216.79667277184 0.9550925

4216.81473889344 0.9789344

4216.83280501505 0.9980648

4216.85087113665 0.9797298

4216.86893725825 0.9396751

4216.88700337985 0.9379937

4216.90506950146 0.955484

4216.92313562306 0.9562647

4216.94120174466 0.9438829

4216.95926786626 0.9448841

4216.97733398787 0.9508003

4216.99540010947 0.9619389

4217.01346623107 0.9781818

4217.03153235267 0.9459779

4217.04959847427 0.8920605

4217.06766459588 0.9047465

4217.08573071748 0.9056212

4217.10379683908 0.9255297

4217.12186296068 0.8824202

4217.13992908229 0.9296015

4217.15799520389 1.007486

4217.17606132549 1.018638

4217.19412744709 0.9921308

4217.21219356869 0.9391124

4217.2302596903 0.9051679

4217.2483258119 0.8985711

4217.2663919335 0.9192103

4217.2844580551 0.9446628

4217.3025241767 0.9201549

4217.32059029831 0.916701

4217.33865641991 0.9349519

4217.35672254151 0.9301597

4217.37478866311 0.9031653

4217.39285478472 0.910454

4217.41092090632 0.9082206

4217.42898702792 0.9152615

4217.44705314952 0.8905593

4217.46511927112 0.8832818

4217.48318539273 0.940039

4217.50125151433 0.9298359

4217.51931763593 0.8750402

4217.53738375753 0.8521258

4217.55544987914 0.8841362

4217.57351600074 0.8895603

4217.59158212234 0.8692803

4217.60964824394 0.8765411

4217.62771436554 0.8986245

4217.64578048715 0.9525844

4217.66384660875 0.9996169

4217.68191273035 0.9500159

4217.69997885195 0.8819264

4217.71804497356 0.9187998

4217.73611109516 0.9522555

4217.75417721676 0.8919783

4217.77224333836 0.8324322

4217.79030945996 0.8488288

4217.80837558157 0.8999622

4217.82644170317 0.926913

4217.84450782477 0.8995513

4217.86257394637 0.8720952

4217.88064006798 0.906324

4217.89870618958 0.9162097

4217.91677231118 0.916567

4217.93483843278 0.9271832

4217.95290455438 0.9236302

4217.97097067599 0.9447416

4217.98903679759 0.9557952

4218.00710291919 0.9353074

4218.02516904079 0.9171743

4218.0432351624 0.8931382

4218.061301284 0.9126003

4218.0793674056 0.9324236

4218.0974335272 0.9334023

4218.1154996488 0.9238162

4218.13356577041 0.9762077

4218.15163189201 1.004671

4218.16969801361 0.9563569

4218.18776413521 0.9360375

4218.20583025681 0.950231

4218.22389637842 0.9429492

4218.24196250002 0.9604211

4218.26002862162 0.9505514

4218.27809474322 0.926505

4218.29616086483 0.9177884

4218.31422698643 0.9343292

4218.33229310803 0.9513922

4218.35035922963 0.9618421

4218.36842535124 0.9722149

4218.38649147284 0.9747856

4218.40455759444 0.9539812

4218.42262371604 0.968531

4218.44068983764 0.9514959

4218.45875595925 1.00367

4218.47682208085 1.000067

4218.49488820245 0.9896039

4218.51295432405 0.9236228

4218.53102044565 0.9466055

4218.54908656726 1.00718

4218.56715268886 0.9950134

4218.58521881046 0.9607395

4218.60328493206 0.9908267

4218.62135105367 0.9757899

4218.63941717527 0.9291955

4218.65748329687 0.9493994

4218.67554941847 0.9663252

4218.69361554007 0.9895151

4218.71168166168 1.005555

4218.72974778328 1.003549

4218.74781390488 1.030577

4218.76588002648 1.014534

4218.78394614808 0.9792048

4218.80201226969 0.9747472

4218.82007839129 0.9655372

4218.83814451289 0.9814706

4218.85621063449 1.004776

4218.8742767561 0.9906636

4218.8923428777 0.9763225

4218.9104089993 0.9893165

4218.9284751209 1.008289

4218.9465412425 0.9779201

4218.96460736411 0.9441724

4218.98267348571 0.9606365

4219.00073960731 1.01943

4219.01880572891 1.028775

4219.03687185052 1.010008

4219.05493797212 0.9922682

4219.07300409372 1.020314

4219.09107021532 1.019252

4219.10913633693 0.925035

4219.12720245853 0.9548957

4219.14526858013 0.9590991

4219.16333470173 0.9435476

4219.18140082333 0.9929872

4219.19946694494 0.9664167

4219.21753306654 0.9120243

4219.23559918814 0.9055459

4219.25366530974 0.9075509

4219.27173143135 0.9170637

4219.28979755295 0.923195

4219.30786367455 0.9563643

4219.32592979615 0.9854558

4219.34399591775 0.9958293

4219.36206203936 0.9873516

4219.38012816096 0.9958693

4219.39819428256 0.9967801

4219.41626040416 0.9745561

4219.43432652576 0.9501228

4219.45239264737 0.9665095

4219.47045876897 0.9693599

4219.48852489057 0.9814887

4219.50659101217 0.9713284

4219.52465713378 0.9804534

4219.54272325538 1.021944

4219.56078937698 1.018401

4219.57885549858 0.9863375

4219.59692162018 0.9673933

4219.61498774179 0.9351921

4219.63305386339 0.945011

4219.65111998499 0.9499325

4219.66918610659 0.8948337

4219.68725222819 0.9067794

4219.7053183498 0.9540846

4219.7233844714 1.00787

4219.741450593 1.001943

4219.7595167146 1.016138

4219.77758283621 0.9907225

4219.79564895781 0.9751933

4219.81371507941 0.9808855

4219.83178120101 0.9493132

4219.84984732262 0.9499631

4219.86791344422 0.9780248

4219.88597956582 0.9961892

4219.90404568742 0.9799314

4219.92211180902 0.9627823

4219.94017793063 0.9900653

4219.95824405223 1.009848

4219.97631017383 1.028224

4219.99437629543 1.01834

4220.01244241703 1.017267

4220.03050853864 0.9994302

4220.04857466024 0.9850826

4220.06664078184 1.015965

4220.08470690344 1.022586

4220.10277302505 0.9920758

4220.12083914665 0.9831276

4220.13890526825 0.9904356

4220.15697138985 1.000458

4220.17503751145 0.9944773

4220.19310363306 0.9819814

4220.21116975466 1.016787

4220.22923587626 1.026747

4220.24730199786 0.9976248

4220.26536811947 0.9936901

4220.28343424107 0.983768

4220.30150036267 0.995032

4220.31956648427 1.007474

4220.33763260587 0.9902638

4220.35569872748 0.9579212

4220.37376484908 0.9722003

4220.39183097068 1.032954

4220.40989709228 1.058807

4220.42796321388 1.036284

4220.44602933549 0.9762825

4220.46409545709 0.962286

4220.48216157869 0.9963234

4220.50022770029 0.9895008

4220.5182938219 0.9906672

4220.5363599435 0.9704981

4220.5544260651 0.9848121

4220.5724921867 1.016464

4220.5905583083 0.9869033

4220.60862442991 0.9899429

4220.62669055151 1.049916

4220.64475667311 1.073721

4220.66282279471 1.004211

4220.68088891632 0.994787

4220.69895503792 1.03297

4220.71702115952 1.015113

4220.73508728112 0.9720617

4220.75315340272 1.008205

4220.77121952433 0.9857448

4220.78928564593 0.9951511

4220.80735176753 1.018333

4220.82541788913 0.9735718

4220.84348401074 0.9701009

4220.86155013234 0.9816961

4220.87961625394 0.9959232

4220.89768237554 1.023657

4220.91574849714 1.004629

4220.93381461875 0.9670947

4220.95188074035 0.966009

4220.96994686195 0.9650794

4220.98801298355 0.9519876

4221.00607910516 0.9592226

4221.02414522676 0.9693874

4221.04221134836 0.9942397

4221.06027746996 1.012

4221.07834359156 1.004865

4221.09640971317 0.9857286

4221.11447583477 0.9469958

4221.13254195637 0.981509

4221.15060807797 1.010083

4221.16867419958 0.9855742

4221.18674032118 1.008077

4221.20480644278 0.9986952

4221.22287256438 0.9683318

4221.24093868599 0.9351749

4221.25900480759 0.955119

4221.27707092919 0.9824835

4221.29513705079 0.9918246

4221.31320317239 1.004954

4221.331269294 0.9760839

4221.3493354156 0.9523996

4221.3674015372 0.9385923

4221.3854676588 0.9296103

4221.4035337804 0.948263

4221.42159990201 0.9867771

4221.43966602361 0.9875541

4221.45773214521 0.9460504

4221.47579826681 0.9698099

4221.49386438842 1.046134

4221.51193051002 1.020384

4221.52999663162 0.9601444

4221.54806275322 0.9702175

4221.56612887482 0.9666179

4221.58419499643 0.9647847

4221.60226111803 1.007959

4221.62032723963 1.022556

4221.63839336123 1.019184

4221.65645948284 1.001451

4221.67452560444 0.9657781

4221.69259172604 0.9503939

4221.71065784764 0.954567

4221.72872396924 0.9675576

4221.74679009085 0.9726378

4221.76485621245 0.9951578

4221.78292233405 0.9765056

4221.80098845565 0.9651102

4221.81905457726 0.9964106

4221.83712069886 0.9953228

4221.85518682046 0.979822

4221.87325294206 0.9973736

4221.89131906366 1.018411

4221.90938518527 0.9762492

4221.92745130687 0.9158992

4221.94551742847 0.9220197

4221.96358355007 0.9769244

4221.98164967167 0.9931681

4221.99971579328 0.9599601

4222.01778191488 0.9351445

4222.03584803648 0.9280677

4222.05391415808 0.923963

4222.07198027968 0.9507985

4222.09004640129 0.9976671

4222.10811252289 0.9843093

4222.12617864449 0.9444053

4222.14424476609 0.9585031

4222.1623108877 0.9638294

4222.1803770093 0.9600203

4222.1984431309 0.9642957

4222.2165092525 0.9730208

4222.2345753741 0.9441881

4222.25264149571 0.933744

4222.27070761731 0.9534613

4222.28877373891 0.9891087

4222.30683986051 0.9902787

4222.32490598212 0.9679193

4222.34297210372 0.9520522

4222.36103822532 0.9742881

4222.37910434692 0.9516704

4222.39717046852 0.9408337

4222.41523659013 0.9358752

4222.43330271173 0.9233609

4222.45136883333 0.9303855

4222.46943495493 0.9344096

4222.48750107654 0.9314435

4222.50556719814 0.9391116

4222.52363331974 0.9297788

4222.54169944134 0.9566841

4222.55976556294 0.973724

4222.57783168455 0.9431778

4222.59589780615 0.9079887

4222.61396392775 0.903353

4222.63203004935 0.924463

4222.65009617096 0.9394152

4222.66816229256 0.9870355

4222.68622841416 0.9665382

4222.70429453576 0.9014832

4222.72236065736 0.9019448

4222.74042677897 0.9211439

4222.75849290057 0.9225085

4222.77655902217 0.909855

4222.79462514377 0.898024

4222.81269126538 0.918245

4222.83075738698 0.929943

4222.84882350858 0.8940157

4222.86688963018 0.9128596

4222.88495575179 0.9175593

4222.90302187339 0.9171338

4222.92108799499 0.9206358

4222.93915411659 0.9575404

4222.95722023819 0.9410189

4222.97528635979 0.9085354

4222.9933524814 0.9373165

4223.011418603 0.956629

4223.0294847246 0.9375489

4223.0475508462 0.9061449

4223.06561696781 0.9150379

4223.08368308941 0.9293206

4223.10174921101 0.9271626

4223.11981533261 0.9104206

4223.13788145421 0.9047475

4223.15594757582 0.9432171

4223.17401369742 0.9649681

4223.19207981902 0.9638803

4223.21014594062 0.9526218

4223.22821206223 0.9387107

4223.24627818383 0.9422855

4223.26434430543 0.9312806

4223.28241042703 0.9141337

4223.30047654864 0.9521475

4223.31854267024 0.9611834

4223.33660879184 0.9528332

4223.35467491344 0.9987448

4223.37274103504 1.010439

4223.39080715665 0.9837244

4223.40887327825 0.9449534

4223.42693939985 0.941449

4223.44500552145 0.9523365

4223.46307164306 0.9848355

4223.48113776466 0.9965233

4223.49920388626 0.9938475

4223.51727000786 0.9439111

4223.53533612946 0.9554919

4223.55340225107 0.9612901

4223.57146837267 0.9726089

4223.58953449427 0.9554337

4223.60760061587 0.9814754

4223.62566673747 0.9839639

4223.64373285908 0.9681573

4223.66179898068 0.968447

4223.67986510228 0.9819052

4223.69793122388 1.004015

4223.71599734549 1.005393

4223.73406346709 1.004024

4223.75212958869 0.9780803

4223.77019571029 0.9575521

4223.78826183189 0.967097

4223.8063279535 0.957512

4223.8243940751 0.9735458

4223.8424601967 0.992974

4223.8605263183 0.972872

4223.87859243991 0.966684

4223.89665856151 0.9766985

4223.91472468311 0.9707647

4223.93279080471 0.9985691

4223.95085692631 0.9866878

4223.96892304792 0.9302959

4223.98698916952 0.9520605

4224.00505529112 0.9898383

4224.02312141272 1.015427

4224.04118753432 0.9987502

4224.05925365593 0.9607704

4224.07731977753 1.000874

4224.09538589913 1.015387

4224.11345202073 0.9852873

4224.13151814234 0.9625814

4224.14958426394 0.9338524

4224.16765038554 0.9466482

4224.18571650714 0.950541

4224.20378262874 0.9382393

4224.22184875035 0.9386628

4224.23991487195 0.9535244

4224.25798099355 0.9313203

4224.27604711515 0.9296669

4224.29411323676 0.9419716

4224.31217935836 0.9360234

4224.33024547996 0.9180232

4224.34831160156 0.9159337

4224.36637772316 0.8984952

4224.38444384477 0.9321821

4224.40250996637 0.949079

4224.42057608797 0.9376833

4224.43864220957 0.9331338

4224.45670833118 0.910731

4224.47477445278 0.8790345

4224.49284057438 0.8801849

4224.51090669598 0.9124452

4224.52897281758 0.9307606

4224.54703893919 0.920853

4224.56510506079 0.916912

4224.58317118239 0.8892421

4224.60123730399 0.898968

4224.61930342559 0.9518588

4224.6373695472 0.9676372

4224.6554356688 0.9148905

4224.6735017904 0.890178

4224.691567912 0.9082965

4224.70963403361 0.9172329

4224.72770015521 0.9283147

4224.74576627681 0.9346004

4224.76383239841 0.9228922

4224.78189852001 0.906858

4224.79996464162 0.9225825

4224.81803076322 0.9019355

4224.83609688482 0.9011402

4224.85416300642 0.9372602

4224.87222912803 0.9527862

4224.89029524963 0.9067591

4224.90836137123 0.891721

4224.92642749283 0.947507

4224.94449361443 0.9438508

4224.96255973604 0.8626893

4224.98062585764 0.897176

4224.99869197924 0.9430966

4225.01675810084 0.95475

4225.03482422245 0.9289323

4225.05289034405 0.8994376

4225.07095646565 0.9114803

4225.08902258725 0.9126611

4225.10708870886 0.8934109

4225.12515483046 0.9163353

4225.14322095206 0.9348802

4225.16128707366 0.9100869

4225.17935319526 0.899875

4225.19741931686 0.8830728

4225.21548543847 0.8697413

4225.23355156007 0.9020569

4225.25161768167 0.9209102

4225.26968380327 0.8882983

4225.28774992488 0.8715253

4225.30581604648 0.8875358

4225.32388216808 0.8808692

4225.34194828968 0.8900815

4225.36001441128 0.9029019

4225.37808053289 0.9033539

4225.39614665449 0.9405726

4225.41421277609 0.9274189

4225.43227889769 0.9200039

4225.4503450193 0.9155299

4225.4684111409 0.9311595

4225.4864772625 0.9283692

4225.5045433841 0.9173408

4225.5226095057 0.9510373

4225.54067562731 0.9726801

4225.55874174891 0.9627308

4225.57680787051 0.9602221

4225.59487399211 0.9178834

4225.61294011372 0.9117232

4225.63100623532 0.9247679

4225.64907235692 0.9535293

4225.66713847852 0.9592507

4225.68520460013 0.9523249

4225.70327072173 0.9459315

4225.72133684333 0.9779786

4225.73940296493 0.9779762

4225.75746908653 0.9454765

4225.77553520814 0.9311042

4225.79360132974 0.9500086

4225.81166745134 0.9695508

4225.82973357294 0.9955534

4225.84779969454 0.9759666

4225.86586581615 0.9467148

4225.88393193775 0.9567086

4225.90199805935 0.9579196

4225.92006418095 0.9688189

4225.93813030256 0.962005

4225.95619642416 0.9506493

4225.97426254576 0.9630618

4225.99232866736 0.9868916

4226.01039478897 0.9466982

4226.02846091057 0.9298602

4226.04652703217 0.9507122

4226.06459315377 0.9343387

4226.08265927537 0.9534169

4226.10072539698 0.9623057

4226.11879151858 0.9641854

4226.13685764018 0.9407051

4226.15492376178 0.9328098

4226.17298988338 0.9267441

4226.19105600499 0.93888

4226.20912212659 0.9732373

4226.22718824819 0.9761794

4226.24525436979 0.9709917

4226.2633204914 0.9628888

4226.281386613 0.9395974

4226.2994527346 0.9473292

4226.3175188562 0.9580492

4226.3355849778 0.9524273

4226.35365109941 0.9575199

4226.37171722101 0.9456382

4226.38978334261 0.9616527

4226.40784946421 0.9720802

4226.42591558582 0.9745488

4226.44398170742 0.9265835

4226.46204782902 0.9594567

4226.48011395062 0.9971261

4226.49818007222 0.9626462

4226.51624619383 0.9518704

4226.53431231543 0.9599298

4226.55237843703 0.9456122

4226.57044455863 0.9543397

4226.58851068023 0.9988065

4226.60657680184 0.9875906

4226.62464292344 0.9580162

4226.64270904504 0.9489871

4226.66077516664 0.947777

4226.67884128825 0.9280658

4226.69690740985 0.9343485

4226.71497353145 1.007017

4226.73303965305 1.014639

4226.75110577466 0.9786121

4226.76917189626 0.9414476

4226.78723801786 0.9151241

4226.80530413946 0.9197407

4226.82337026106 0.9425185

4226.84143638267 0.9433004

4226.85950250427 0.9385636

4226.87756862587 0.9244788

4226.89563474747 0.9317013

4226.91370086907 0.9432319

4226.93176699068 0.965348

4226.94983311228 0.9858449

4226.96789923388 0.9472252

4226.98596535548 0.8989968

4227.00403147709 0.9136809

4227.02209759869 0.9216943

4227.04016372029 0.9384436

4227.05822984189 0.9370065

4227.07629596349 0.931562

4227.0943620851 0.9443874

4227.1124282067 0.9221623

4227.1304943283 0.8883876

4227.1485604499 0.8978438

4227.1666265715 0.914444

4227.18469269311 0.9160396

4227.20275881471 0.9189285

4227.22082493631 0.9270908

4227.23889105791 0.9124408

4227.25695717952 0.8893605

4227.27502330112 0.8810457

4227.29308942272 0.8935001

4227.31115554432 0.9142746

4227.32922166592 0.9067595

4227.34728778753 0.8990045

4227.36535390913 0.8997353

4227.38342003073 0.9158835

4227.40148615233 0.9334613

4227.41955227394 0.9057951

4227.43761839554 0.9069266

4227.45568451714 0.9144979

4227.47375063874 0.8926097

4227.49181676034 0.8925494

4227.50988288195 0.8796244

4227.52794900355 0.9160463

4227.54601512515 0.9073436

4227.56408124675 0.8661413

4227.58214736836 0.8679018

4227.60021348996 0.8618146

4227.61827961156 0.8687319

4227.63634573316 0.8757871

4227.65441185477 0.8711229

4227.67247797637 0.9038926

4227.69054409797 0.8996272

4227.70861021957 0.8770046

4227.72667634117 0.883794

4227.74474246278 0.8682724

4227.76280858438 0.891095

4227.78087470598 0.894624

4227.79894082758 0.8889109

4227.81700694918 0.895854

4227.83507307079 0.9030859

4227.85313919239 0.9204068

4227.87120531399 0.9203166

4227.88927143559 0.9291968

4227.9073375572 0.9130005

4227.9254036788 0.8992849

4227.9434698004 0.8880559

4227.961535922 0.8789997

4227.9796020436 0.8930926

4227.99766816521 0.9247557

4228.01573428681 0.9364023

4228.03380040841 0.9315637

4228.05186653001 0.9444826

4228.06993265162 0.9508433

4228.08799877322 0.9331881

4228.10606489482 0.9484928

4228.12413101642 0.9516539

4228.14219713802 0.9294695

4228.16026325963 0.9191135

4228.17832938123 0.9431796

4228.19639550283 0.9660723

4228.21446162443 0.9720429

4228.23252774603 0.9776587

4228.25059386764 0.9549106

4228.26865998924 0.9428099

4228.28672611084 0.9503278

4228.30479223244 0.9192213

4228.32285835405 0.910737

4228.34092447565 0.9448112

4228.35899059725 0.9484074

4228.37705671885 0.9539613

4228.39512284045 0.9419444

4228.41318896206 0.9314815

4228.43125508366 0.9386285

4228.44932120526 0.9312114

4228.46738732686 0.9037552

4228.48545344847 0.9034355

4228.50351957007 0.9336264

4228.52158569167 0.9586979

4228.53965181327 0.9408523

4228.55771793487 0.9238127

4228.57578405648 0.9534562

4228.59385017808 0.9385858

4228.61191629968 0.9295883

4228.62998242128 0.9399472

4228.64804854289 0.9443698

4228.66611466449 0.9565449

4228.68418078609 0.944321

4228.70224690769 0.9079614

4228.72031302929 0.9131659

4228.7383791509 0.9503539

4228.7564452725 0.9549533

4228.7745113941 0.9343663

4228.7925775157 0.951207

4228.8106436373 0.9524338

4228.82870975891 0.9411217

4228.84677588051 0.9460437

4228.86484200211 0.9539708

4228.88290812371 0.956558

4228.90097424532 0.9538326

4228.91904036692 0.9383729

4228.93710648852 0.9402368

4228.95517261012 0.9244024

4228.97323873172 0.9074703

4228.99130485333 0.9272054

4229.00937097493 0.932657

4229.02743709653 0.9241748

4229.04550321813 0.959439

4229.06356933974 0.9917577

4229.08163546134 0.9863139

4229.09970158294 0.9772143

4229.11776770454 0.9683528

4229.13583382614 0.9582658

4229.15389994775 0.9309798

4229.17196606935 0.9050913

4229.19003219095 0.9447209

4229.20809831255 0.9530259

4229.22616443416 0.9068216

4229.24423055576 0.9148313

4229.26229667736 0.947314

4229.28036279896 0.9309191

4229.29842892056 0.9256486

4229.31649504217 0.9355944

4229.33456116377 0.9165435

4229.35262728537 0.9161007

4229.37069340697 0.940165

4229.38875952857 0.9345419

4229.40682565018 0.9205765

4229.42489177178 0.9411742

4229.44295789338 0.9383795

4229.46102401498 0.9063566

4229.47909013659 0.9046365

4229.49715625819 0.9455848

4229.51522237979 0.9297265

4229.53328850139 0.8821954

4229.55135462299 0.8870355

4229.5694207446 0.8946368

4229.5874868662 0.9147002

4229.6055529878 0.9178359

4229.6236191094 0.8889115

4229.64168523101 0.8735636

4229.65975135261 0.872794

4229.67781747421 0.8803612

4229.69588359581 0.9142601

4229.71394971741 0.9076095

4229.73201583902 0.9026419

4229.75008196062 0.8952894

4229.76814808222 0.9142771

4229.78621420382 0.9088496

4229.80428032543 0.9059795

4229.82234644703 0.914427

4229.84041256863 0.906232

4229.85847869023 0.8914458

4229.87654481184 0.918968

4229.89461093344 0.9226409

4229.91267705504 0.9148766

4229.93074317664 0.9036434

4229.94880929824 0.8920743

4229.96687541985 0.9061227

4229.98494154145 0.9311677

4230.00300766305 0.920979

4230.02107378465 0.9041409

4230.03913990626 0.9285975

4230.05720602786 0.9377239

4230.07527214946 0.8920879

4230.09333827106 0.8959069

4230.11140439266 0.9011899

4230.12947051427 0.9302808

4230.14753663587 0.917648

4230.16560275747 0.9264129

4230.18366887907 0.9434861

4230.20173500067 0.9339578

4230.21980112228 0.9322594

4230.23786724388 0.880998

4230.25593336548 0.8944406

4230.27399948708 0.9315776

4230.29206560869 0.9255828

4230.31013173029 0.9481976

4230.32819785189 0.9534098

4230.34626397349 0.9545227

4230.36433009509 0.8975327

4230.38239621669 0.8743783

4230.4004623383 0.9292496

4230.4185284599 0.9204561

4230.4365945815 0.9041693

4230.4546607031 0.9075183

4230.47272682471 0.915062

4230.49079294631 0.9207276

4230.50885906791 0.9101332

4230.52692518951 0.9249169

4230.54499131112 0.9413692

4230.56305743272 0.9548849

4230.58112355432 0.9619769

4230.59918967592 0.932845

4230.61725579753 0.944983

4230.63532191913 0.9477698

4230.65338804073 0.9299729

4230.67145416233 0.9533443

4230.68952028393 0.9687017

4230.70758640554 0.9354882

4230.72565252714 0.9184996

4230.74371864874 0.9503623

4230.76178477034 1.009501

4230.77985089194 0.9802172

4230.79791701355 0.924883

4230.81598313515 0.8857003

4230.83404925675 0.9102485

4230.85211537835 0.9559474

4230.87018149996 0.9468026

4230.88824762156 0.884436

4230.90631374316 0.8868387

4230.92437986476 0.9016445

4230.94244598637 0.9405798

4230.96051210797 0.9194704

4230.97857822957 0.8930392

4230.99664435117 0.9213899

4231.01471047277 0.9677438

4231.03277659438 0.9590412

4231.05084271598 0.9333113

4231.06890883758 0.9258246

4231.08697495918 0.9402338

4231.10504108078 0.9192649

4231.12310720239 0.9607781

4231.14117332399 0.9422268

4231.15923944559 0.9043048

4231.17730556719 0.905625

4231.1953716888 0.9293048

4231.2134378104 0.9351225

4231.231503932 0.9469267

4231.2495700536 0.9622988

4231.2676361752 0.8963838

4231.28570229681 0.9032072

4231.30376841841 0.9381682

4231.32183454001 0.965615

4231.33990066161 0.9737525

4231.35796678321 0.9600228

4231.37603290482 0.9459832

4231.39409902642 0.9469017

4231.41216514802 0.9649225

4231.43023126962 0.9369859

4231.44829739123 0.9051993

4231.46636351283 0.9386656

4231.48442963443 0.9618447

4231.50249575603 0.9511446

4231.52056187763 0.9773691

4231.53862799924 0.9999672

4231.55669412084 0.9886786

4231.57476024244 0.9720752

4231.59282636404 0.9497426

4231.61089248565 0.9319202

4231.62895860725 0.9303699

4231.64702472885 0.9161615

4231.66509085045 0.9240035

4231.68315697206 0.9340313

4231.70122309366 0.9342779

4231.71928921526 0.9569188

4231.73735533686 0.9476478

4231.75542145846 0.9548932

4231.77348758007 0.9466865

4231.79155370167 0.9202005

4231.80961982327 0.9302592

4231.82768594487 0.9441098

4231.84575206648 0.938596

4231.86381818808 0.9421117

4231.88188430968 0.9186207

4231.89995043128 0.9000375

4231.91801655288 0.9212625

4231.93608267449 0.9209914

4231.95414879609 0.9199104

4231.97221491769 0.9267582

4231.99028103929 0.8925033

4232.00834716089 0.8823274

4232.0264132825 0.8886619

4232.0444794041 0.882997

4232.0625455257 0.9159466

4232.0806116473 0.9412284

4232.09867776891 0.9162391

4232.11674389051 0.8984659

4232.13481001211 0.912138

4232.15287613371 0.9474403

4232.17094225531 0.9504814

4232.18900837692 0.9087549

4232.20707449852 0.8887084

4232.22514062012 0.932538

4232.24320674172 0.9659317

4232.26127286332 0.9402953

4232.27933898493 0.9082541

4232.29740510653 0.8958831

4232.31547122813 0.8867589

4232.33353734973 0.9215672

4232.35160347133 0.9307208

4232.36966959294 0.8983375

4232.38773571454 0.8767631

4232.40580183614 0.9021431

4232.42386795774 0.9031403

4232.44193407935 0.8735582

4232.46000020095 0.8700241

4232.47806632255 0.8921006

4232.49613244415 0.9101673

4232.51419856576 0.9001693

4232.53226468736 0.9141116

4232.55033080896 0.9146692

4232.56839693056 0.8998393

4232.58646305216 0.8898357

4232.60452917377 0.8927718

4232.62259529537 0.9137601

4232.64066141697 0.8905531

4232.65872753857 0.9033284

4232.67679366018 0.8996219

4232.69485978178 0.8630368

4232.71292590338 0.8946121

4232.73099202498 0.9424853

4232.74905814658 0.9086151

4232.76712426819 0.9057016

4232.78519038979 0.9229825

4232.80325651139 0.8996682

4232.82132263299 0.8940556

4232.8393887546 0.9228538

4232.8574548762 0.9085747

4232.8755209978 0.8722764

4232.8935871194 0.8634163

4232.91165324101 0.9001981

4232.92971936261 0.869575

4232.94778548421 0.8502183

4232.96585160581 0.8406377

4232.98391772741 0.8354356

4233.00198384901 0.7970166

4233.02004997062 0.815396

4233.03811609222 0.8218471

4233.05618221382 0.7697693

4233.07424833542 0.7416874

4233.09231445703 0.7575973

4233.11038057863 0.741274

4233.12844670023 0.7461437

4233.14651282183 0.7341444

4233.16457894343 0.7453821

4233.18264506504 0.7120337

4233.20071118664 0.6876214

4233.21877730824 0.7025942

4233.23684342984 0.7249995

4233.25490955145 0.7321146

4233.27297567305 0.7356448

4233.29104179465 0.7229018

4233.30910791625 0.7119704

4233.32717403786 0.7177593

4233.34524015946 0.7256258

4233.36330628106 0.7066262

4233.38137240266 0.7256361

4233.39943852426 0.7442815

4233.41750464587 0.7495487

4233.43557076747 0.7484319

4233.45363688907 0.7281756

4233.47170301067 0.6985832

4233.48976913227 0.6947781

4233.50783525388 0.7368374

4233.52590137548 0.7663615

4233.54396749708 0.7751347

4233.56203361868 0.7811676

4233.58009974028 0.7970188

4233.59816586189 0.8210456

4233.61623198349 0.8207451

4233.63429810509 0.794376

4233.65236422669 0.7386247

4233.6704303483 0.7617623

4233.6884964699 0.8300825

4233.7065625915 0.7914096

4233.7246287131 0.8031906

4233.74269483471 0.8327156

4233.76076095631 0.8482891

4233.77882707791 0.8235536

4233.79689319951 0.8517668

4233.81495932111 0.9040466

4233.83302544272 0.8759916

4233.85109156432 0.8235028

4233.86915768592 0.8342974

4233.88722380752 0.8444837

4233.90528992912 0.8655168

4233.92335605073 0.8560526

4233.94142217233 0.8635375

4233.95948829393 0.8601449

4233.97755441553 0.8674437

4233.99562053714 0.902873

4234.01368665874 0.8751782

4234.03175278034 0.8405536

4234.04981890194 0.8116785

4234.06788502355 0.8219857

4234.08595114515 0.8813238

4234.10401726675 0.9091116

4234.12208338835 0.8900783

4234.14014950995 0.8576245

4234.15821563155 0.8885988

4234.17628175316 0.9019753

4234.19434787476 0.8536916

4234.21241399636 0.782496

4234.23048011796 0.8379742

4234.24854623957 0.8991061

4234.26661236117 0.8874189

4234.28467848277 0.9018928

4234.30274460437 0.9173248

4234.32081072597 0.9116422

4234.33887684758 0.9014237

4234.35694296918 0.9320592

4234.37500909078 0.9390771

4234.39307521238 0.8915899

4234.41114133399 0.8715724

4234.42920745559 0.8895905

4234.44727357719 0.8863558

4234.46533969879 0.9068207

4234.4834058204 0.9286906

4234.501471942 0.8941144

4234.5195380636 0.8913617

4234.5376041852 0.9237348

4234.5556703068 0.91665

4234.57373642841 0.9116088

4234.59180255001 0.9398022

4234.60986867161 0.9234791

4234.62793479321 0.8766477

4234.64600091481 0.8896238

4234.66406703642 0.9307073

4234.68213315802 0.9840628

4234.70019927962 0.9744612

4234.71826540122 0.8996496

4234.73633152283 0.9514716

4234.75439764443 0.989148

4234.77246376603 0.9361854

4234.79052988763 0.9319844

4234.80859600924 0.9320635

4234.82666213084 0.9284986

4234.84472825244 0.9048792

4234.86279437404 0.8928208

4234.88086049564 0.9062561

4234.89892661725 0.9640151

4234.91699273885 0.9838774

4234.93505886045 0.9668615

4234.95312498205 0.9343797

4234.97119110366 0.9296285

4234.98925722526 0.8924154

4235.00732334686 0.8899087

4235.02538946846 0.8813783

4235.04345559006 0.8855325

4235.06152171167 0.8993783

4235.07958783327 0.8744172

4235.09765395487 0.8772868

4235.11572007647 0.880728

4235.13378619808 0.8826459

4235.15185231968 0.8694643

4235.16991844128 0.8505893

4235.18798456288 0.8836145

4235.20605068448 0.894176

4235.22411680609 0.8714432

4235.24218292769 0.8352708

4235.26024904929 0.8437645

4235.27831517089 0.868534

4235.29638129249 0.8534954

4235.3144474141 0.8382685

4235.3325135357 0.8744538

4235.3505796573 0.9041013

4235.3686457789 0.879537

4235.38671190051 0.8782141

4235.40477802211 0.9245436

4235.42284414371 0.8937712

4235.44091026531 0.8635104

4235.45897638691 0.874907

4235.47704250852 0.8662341

4235.49510863012 0.8772084

4235.51317475172 0.8929913

4235.53124087332 0.9025755

4235.54930699492 0.8407019

4235.56737311653 0.816321

4235.58543923813 0.8315358

4235.60350535973 0.8823849

4235.62157148133 0.9144605

4235.63963760294 0.8472679

4235.65770372454 0.8026501

4235.67576984614 0.8352883

4235.69383596774 0.8594949

4235.71190208934 0.8556544

4235.72996821095 0.8876593

4235.74803433255 0.9199755

4235.76610045415 0.8955445

4235.78416657575 0.8469092

4235.80223269736 0.8587919

4235.82029881896 0.8738861

4235.83836494056 0.8981187

4235.85643106216 0.8763973

4235.87449718376 0.839406

4235.89256330537 0.8706395

4235.91062942697 0.8990633

4235.92869554857 0.9166377

4235.94676167017 0.8509691

4235.96482779178 0.8728291

4235.98289391338 0.881242

4236.00096003498 0.9005488

4236.01902615658 0.9134092

4236.03709227818 0.95516

4236.05515839979 0.9140825

4236.07322452139 0.9069638

4236.09129064299 0.9181607

4236.10935676459 0.9122771

4236.12742288619 0.9276065

4236.1454890078 0.9089323

4236.1635551294 0.8653307

4236.181621251 0.8693029

4236.1996873726 0.8471795

4236.21775349421 0.893487

4236.23581961581 0.9289206

4236.25388573741 0.9788153

4236.27195185901 0.9674

4236.29001798061 0.9726149

4236.30808410222 0.9305934

4236.32615022382 0.9554621

4236.34421634542 0.9557612

4236.36228246702 0.948693

4236.38034858863 0.9445727

4236.39841471023 0.960261

4236.41648083183 0.9617373

4236.43454695343 0.9462596

4236.45261307504 0.9216342

4236.47067919664 0.9564114

4236.48874531824 0.9546368

4236.50681143984 0.9203476

4236.52487756144 0.9424375

4236.54294368304 0.9598662

4236.56100980465 0.9152321

4236.57907592625 0.8888389

4236.59714204785 0.931908

4236.61520816945 0.9517584

4236.63327429106 0.8993206

4236.65134041266 0.9014436

4236.66940653426 0.9470561

4236.68747265586 0.9751625

4236.70553877747 0.9636598

4236.72360489907 0.9666375

4236.74167102067 0.9858489

4236.75973714227 0.9468368

4236.77780326387 0.926417

4236.79586938548 0.9017835

4236.81393550708 0.9048588

4236.83200162868 0.9557772

4236.85006775028 0.9591979

4236.86813387189 0.9212608

4236.88619999349 0.9026473

4236.90426611509 0.9148412

4236.92233223669 0.9280316

4236.94039835829 0.962914

4236.9584644799 0.9226866

4236.9765306015 0.9152921

4236.9945967231 0.899826

4237.0126628447 0.8736626

4237.03072896631 0.9347705

4237.04879508791 1.02082

4237.06686120951 0.9786986

4237.08492733111 0.9345779

4237.10299345271 0.9446383

4237.12105957432 0.9061596

4237.13912569592 0.9642408

4237.15719181752 1.04957

4237.17525793912 1.015312

4237.19332406072 0.9970703

4237.21139018233 0.9850563

4237.22945630393 0.9575914

4237.24752242553 0.9863082

4237.26558854713 0.9810511

4237.28365466874 0.9753619

4237.30172079034 0.9891673

4237.31978691194 1.013835

4237.33785303354 0.9549179

4237.35591915514 0.9513187

4237.37398527675 0.9498805

4237.39205139835 0.9707285

4237.41011751995 0.9284428

4237.42818364155 0.9763927

4237.44624976316 1.013049

4237.46431588476 0.966489

4237.48238200636 0.9468982

4237.50044812796 0.9387294

4237.51851424957 0.9477217

4237.53658037117 0.9802391

4237.55464649277 1.019308

4237.57271261437 1.019073

4237.59077873597 0.9677852

4237.60884485758 0.9293423

4237.62691097918 0.9746951

4237.64497710078 1.014738

4237.66304322238 0.9464388

4237.68110934399 0.9414687

4237.69917546559 1.007572

4237.71724158719 1.027143

4237.73530770879 0.9869606

4237.75337383039 0.9442697

4237.77143995199 0.9819858

4237.7895060736 1.053712

4237.8075721952 1.030139

4237.8256383168 1.004157

4237.8437044384 1.051016

4237.86177056001 1.005614

4237.87983668161 0.9703373

4237.89790280321 0.9723739

4237.91596892481 0.9863974

4237.93403504642 0.9961063

4237.95210116802 1.0085

4237.97016728962 1.021899

4237.98823341122 1.024845

4238.00629953282 0.9783922

4238.02436565443 0.9853641

4238.04243177603 0.9790972

4238.06049789763 0.9252852

4238.07856401923 0.8806369

4238.09663014084 0.9139833

4238.11469626244 0.9347231

4238.13276238404 0.9144341

4238.15082850564 0.9175281

4238.16889462724 0.948176

4238.18696074885 0.8948865

4238.20502687045 0.8691821

4238.22309299205 0.894215

4238.24115911365 0.9597803

4238.25922523525 0.954437

4238.27729135686 0.8999941

4238.29535747846 0.9014004

4238.31342360006 0.9506935

4238.33148972166 1.023339

4238.34955584327 0.915512

4238.36762196487 0.846307

4238.38568808647 0.8916398

4238.40375420807 0.9016904

4238.42182032967 0.9374641

4238.43988645128 0.9236087

4238.45795257288 0.9019367

4238.47601869448 0.9120978

4238.49408481608 0.9733282

4238.51215093769 0.9565238

4238.53021705929 0.9182519

4238.54828318089 0.9365518

4238.56634930249 0.8892025

4238.58441542409 0.9130628

4238.6024815457 0.9644248

4238.6205476673 0.8855308

4238.6386137889 0.9417847

4238.6566799105 1.010109

4238.67474603211 0.9296466

4238.69281215371 0.9038333

4238.71087827531 0.9319556

4238.72894439691 0.9802014

4238.74701051851 0.9527093

4238.76507664012 0.8512367

4238.78314276172 0.8445476

4238.80120888332 0.8789813

4238.81927500492 0.8964581

4238.83734112652 0.9481771

4238.85540724813 0.9857699

4238.87347336973 0.9890946

4238.89153949133 0.9521915

4238.90960561293 0.8588712

4238.92767173453 0.8406949

4238.94573785614 0.8964731

4238.96380397774 0.879284

4238.98187009934 0.899183

4238.99993622095 0.9697174

4239.01800234255 0.9238873

4239.03606846415 0.9259864

4239.05413458575 0.9521273

4239.07220070735 0.9122881

4239.09026682896 0.8595172

4239.10833295056 0.9279805

4239.12639907216 0.91192

4239.14446519376 0.9177515

4239.16253131536 0.9838353

4239.18059743697 0.9302514

4239.19866355857 0.8796486

4239.21672968017 0.9298841

4239.23479580177 0.9564922

4239.25286192338 0.9474647

4239.27092804498 0.9373364

4239.28899416658 0.9475087

4239.30706028818 0.929234

4239.32512640978 0.967929

4239.34319253139 1.00895

4239.36125865299 0.9370118

4239.37932477459 0.9459743

4239.39739089619 0.9635931

4239.4154570178 0.9494976

4239.4335231394 0.8914924

4239.451589261 0.9119034

4239.4696553826 0.9675274

4239.4877215042 0.9395573

4239.50578762581 0.9340636

4239.52385374741 0.9578044

4239.54191986901 0.9482257

4239.55998599061 0.9162195

4239.57805211222 0.8836095

4239.59611823382 0.904457

4239.61418435542 0.8811431

4239.63225047702 0.8978121

4239.65031659862 0.9514285

4239.66838272023 0.8879899

4239.68644884183 0.8824644

4239.70451496343 0.9452907

4239.72258108503 0.8904958

4239.74064720663 0.9144124

4239.75871332824 0.9386045

4239.77677944984 0.8669987

4239.79484557144 0.8819554

4239.81291169304 0.8874232

4239.83097781465 0.8816421

4239.84904393625 0.908978

4239.86711005785 0.9302109

4239.88517617945 0.9580188

4239.90324230106 0.9618114

4239.92130842266 0.9590689

4239.93937454426 0.9439616

4239.95744066586 0.8905225

4239.97550678746 0.9093087

4239.99357290907 0.897231

4240.01163903067 0.9531532

4240.02970515227 0.911696

4240.04777127387 0.9006139

4240.06583739548 0.8631002

4240.08390351708 0.9302328

4240.10196963868 0.9250231

4240.12003576028 0.928119

4240.13810188188 0.9291201

4240.15616800349 0.9708778

4240.17423412509 0.9615368

4240.19230024669 0.9491874

4240.21036636829 0.9386203

4240.2284324899 0.965413

4240.2464986115 0.9797792

4240.2645647331 0.9695815

4240.2826308547 0.9178428

4240.3006969763 0.9742009

4240.31876309791 0.9537092

4240.33682921951 0.9165356

4240.35489534111 0.8977503

4240.37296146271 0.9135984

4240.39102758431 0.9237441

4240.40909370592 0.9470741

4240.42715982752 0.9298324

4240.44522594912 0.9350364

4240.46329207072 0.9881151

4240.48135819232 1.032551

4240.49942431393 0.9762014

4240.51749043553 0.9692427

4240.53555655713 0.955204

4240.55362267873 0.9446101

4240.57168880034 0.9439569

4240.58975492194 0.9401994

4240.60782104354 0.867806

4240.62588716514 0.8564776

4240.64395328674 0.7521563

4240.66201940835 0.8187963

4240.68008552995 0.9414816

4240.69815165155 0.9455864

4240.71621777315 0.9408507

4240.73428389476 0.9847635

4240.75235001636 0.9391818

4240.77041613796 0.9767618

4240.78848225956 0.9735875

4240.80654838116 0.9936152

4240.82461450277 1.014618

4240.84268062437 0.9130408

4240.86074674597 0.9766147

4240.87881286757 1.002924

4240.89687898918 0.944215

4240.91494511078 0.9533361

4240.93301123238 0.9812533

4240.95107735398 1.006211

4240.96914347558 0.9487938

4240.98720959719 1.006856

4241.00527571879 0.9687018

4241.02334184039 0.9504924

4241.04140796199 0.9788235

4241.05947408359 1.005468

4241.0775402052 1.082977

4241.0956063268 1.038292

4241.1136724484 0.970235

4241.13173857 0.9209341

4241.14980469161 0.89332

4241.16787081321 0.9109935

4241.18593693481 0.9422901

4241.20400305641 0.9534027

4241.22206917802 0.9461575

4241.24013529962 0.9805588

4241.25820142122 1.011034

4241.27626754282 0.9627969

4241.29433366442 0.9608871

4241.31239978603 1.030778

4241.33046590763 1.002258

4241.34853202923 0.9407873

4241.36659815083 0.944634

4241.38466427243 0.9764874

4241.40273039404 0.9972904

4241.42079651564 0.9716378

4241.43886263724 0.933239

4241.45692875884 0.914138

4241.47499488045 0.9535778

4241.49306100205 1.038085

4241.51112712365 0.9801127

4241.52919324525 0.9557122

4241.54725936685 1.044413

4241.56532548846 1.070295

4241.58339161006 0.9715211

4241.60145773166 0.9003556

4241.61952385326 0.8929598

4241.63758997487 0.944846

4241.65565609647 1.017992

4241.67372221807 1.045028

4241.69178833967 1.016147

4241.70985446128 0.9880966

4241.72792058288 0.9659773

4241.74598670448 0.9878776

4241.76405282608 1.014522

4241.78211894768 1.033372

4241.80018506929 0.9912877

4241.81825119089 0.9667886

4241.83631731249 1.019801

4241.85438343409 1.022511

4241.8724495557 0.9663025

4241.8905156773 0.9484596

4241.9085817989 1.043122

4241.9266479205 1.022764

4241.9447140421 0.8841518

4241.9627801637 0.8908438

4241.98084628531 0.9441156

4241.99891240691 0.9845465

4242.01697852851 0.9944795

4242.03504465011 0.9697612

4242.05311077172 0.9478875

4242.07117689332 0.9295191

4242.08924301492 0.9656545

4242.10730913652 0.9553359

4242.12537525812 0.9525941

4242.14344137973 0.9087529

4242.16150750133 0.8400587

4242.17957362293 0.9239385

4242.19763974453 0.963952

4242.21570586614 0.9341727

4242.23377198774 0.8320614

4242.25183810934 0.8364236

4242.26990423094 0.8708493

4242.28797035255 0.8929713

4242.30603647415 0.872541

4242.32410259575 0.8867922

4242.34216871735 0.9386026

4242.36023483895 0.9206187

4242.37830096056 0.91291

4242.39636708216 0.8745279

4242.41443320376 0.7581171

4242.43249932536 0.7694498

4242.45056544696 0.8401861

4242.46863156857 0.8639452

4242.48669769017 0.8481075

4242.50476381177 0.8474082

4242.52282993337 0.8944395

4242.54089605498 0.8368282

4242.55896217658 0.7536289

4242.57702829818 0.8024496

4242.59509441978 0.8722534

4242.61316054139 0.830369

4242.63122666299 0.8738657

4242.64929278459 0.8791263

4242.66735890619 0.8350952

4242.68542502779 0.7723224

4242.7034911494 0.7706806

4242.721557271 0.8924458

4242.7396233926 0.8830038

4242.7576895142 0.8201295

4242.7757556358 0.8835331

4242.79382175741 0.9062676

4242.81188787901 0.8966036

4242.82995400061 0.8763695

4242.84802012221 0.8439741

4242.86608624382 0.9273572

4242.88415236542 0.9780412

4242.90221848702 0.8833863

4242.92028460862 0.8610778

4242.93835073022 0.9150738

4242.95641685183 0.9984848

4242.97448297343 1.014124

4242.99254909503 0.9267698

4243.01061521663 0.9407209

4243.02868133824 1.022321

4243.04674745984 1.03702

4243.06481358144 1.003399

4243.08287970304 0.9652569

4243.10094582464 0.9695699

4243.11901194625 0.9894163

4243.13707806785 0.9690517

4243.15514418945 0.9826846

4243.17321031105 0.9727395

4243.19127643266 0.9190911

4243.20934255426 0.8894059

4243.22740867586 0.9086339

4243.24547479746 0.927276

4243.26354091906 0.9616339

4243.28160704067 1.044482

4243.29967316227 1.051409

4243.31773928387 0.9838711

4243.33580540547 0.9321169

4243.35387152707 0.9746176

4243.37193764868 1.040907

4243.39000377028 1.018462

4243.40806989188 0.9583459

4243.42613601348 0.9094102

4243.44420213509 0.9911796

4243.46226825669 1.050617

4243.48033437829 0.9391348

4243.49840049989 0.8859776

4243.51646662149 0.9639386

4243.5345327431 0.9966128

4243.5525988647 0.9357305

4243.5706649863 0.9047208

4243.5887311079 0.9196351

4243.60679722951 0.9308366

4243.62486335111 0.9624415

4243.64292947271 0.9860864

4243.66099559431 0.9791554

4243.67906171591 0.9897985

4243.69712783752 1.010345

4243.71519395912 1.01213

4243.73326008072 0.9850451

4243.75132620232 0.9402099

4243.76939232393 0.8878218

4243.78745844553 0.8897756

4243.80552456713 0.9356862

4243.82359068873 0.9197015

4243.84165681033 0.9318861

4243.85972293194 0.8850423

4243.87778905354 0.9657548

4243.89585517514 1.016838

4243.91392129674 0.9681125

4243.93198741834 0.9500279

4243.95005353995 0.9474208

4243.96811966155 0.9111328

4243.98618578315 0.9578506

4244.00425190475 0.9379036

4244.02231802636 0.9241971

4244.04038414796 1.00488

4244.05845026956 0.9882815

4244.07651639116 0.9229459

4244.09458251276 0.9336942

4244.11264863437 0.9892551

4244.13071475597 0.9666988

4244.14878087757 0.9202907

4244.16684699917 0.960179

4244.18491312078 0.968377

4244.20297924238 0.959825

4244.22104536398 1.030085

4244.23911148558 1.032289

4244.25717760718 1.007218

4244.27524372879 1.020099

4244.29330985039 0.9393096

4244.31137597199 0.9169893

4244.32944209359 0.9447623

4244.3475082152 0.9710777

4244.3655743368 0.9183381

4244.3836404584 0.8637561

4244.40170658 0.8723581

4244.4197727016 0.876417

4244.43783882321 0.9313682

4244.45590494481 0.8966606

4244.47397106641 0.8720931

4244.49203718801 0.9268912

4244.51010330962 0.96504

4244.52816943122 0.9939351

4244.54623555282 0.9602519

4244.56430167442 0.9641865

4244.58236779602 1.000429

4244.60043391763 0.9545287

4244.61850003923 0.929581

4244.63656616083 0.965183

4244.65463228243 0.9857845

4244.67269840404 1.008031

4244.69076452564 0.9984648

4244.70883064724 0.9445876

4244.72689676884 0.9594513

4244.74496289044 1.01127

4244.76302901205 0.968125

4244.78109513365 0.9152693

4244.79916125525 0.9119532

4244.81722737685 0.9591373

4244.83529349846 0.9985776

4244.85335962006 0.9314157

4244.87142574166 0.9194257

4244.88949186326 0.9708884

4244.90755798486 0.9794846

4244.92562410647 0.9318587

4244.94369022807 0.9019306

4244.96175634967 0.9403166

4244.97982247127 1.013525

4244.99788859287 1.045049

4245.01595471448 0.9895614

4245.03402083608 0.9645765

4245.05208695768 1.005555

4245.07015307928 0.941554

4245.08821920089 0.9379265

4245.10628532249 0.9617995

4245.12435144409 0.8874081

4245.14241756569 0.8761154

4245.16048368729 0.9176059

4245.1785498089 0.9692388

4245.1966159305 1.046046

4245.2146820521 1.0216

4245.2327481737 0.981491

4245.25081429531 0.9768265

4245.26888041691 0.9762852

4245.28694653851 0.967018

4245.30501266011 0.9328679

4245.32307878171 0.923437

4245.34114490332 0.9449821

4245.35921102492 0.9381641

4245.37727714652 0.962352

4245.39534326812 1.003084

4245.41340938973 0.9890006

4245.43147551133 0.9739541

4245.44954163293 0.9919056

4245.46760775453 0.9553682

4245.48567387613 0.9092274

4245.50373999774 0.9625353

4245.52180611934 1.024664

4245.53987224094 0.9656279

4245.55793836254 0.9241155

4245.57600448414 0.9362855

4245.59407060575 0.9520588

4245.61213672735 0.9614633

4245.63020284895 0.9738686

4245.64826897055 0.9762943

4245.66633509216 0.9531946

4245.68440121376 0.9293971

4245.70246733536 0.9329017

4245.72053345696 1.003922

4245.73859957856 1.049188

4245.75666570017 1.01744

4245.77473182177 0.9581817

4245.79279794337 0.9427547

4245.81086406497 0.9701822

4245.82893018657 0.9937445

4245.84699630818 1.003556

4245.86506242978 0.9716099

4245.88312855138 0.9193563

4245.90119467298 0.9186464

4245.91926079459 0.9342924

4245.93732691619 0.9594535

4245.95539303779 0.951882

4245.97345915939 0.9326307

4245.991525281 0.9051849

4246.0095914026 0.8935967

4246.0276575242 0.951324

4246.0457236458 0.9433451

4246.0637897674 0.9451377

4246.08185588901 0.9085563

4246.09992201061 0.8858475

4246.11798813221 0.890741

4246.13605425381 0.8695307

4246.15412037541 0.9144244

4246.17218649702 0.9526434

4246.19025261862 0.9418162

4246.20831874022 0.9132558

4246.22638486182 0.9007995

4246.24445098343 0.9331125

4246.26251710503 0.9145239

4246.28058322663 0.8587283

4246.29864934823 0.8592593

4246.31671546984 0.9186835

4246.33478159144 0.9654096

4246.35284771304 0.9429576

4246.37091383464 0.9164085

4246.38897995624 0.9364624

4246.40704607785 0.9431022

4246.42511219945 0.9131538

4246.44317832105 0.905892

4246.46124444265 0.8899835

4246.47931056426 0.8601736

4246.49737668586 0.9184108

4246.51544280746 0.9357425

4246.53350892906 0.9117056

4246.55157505066 0.9298592

4246.56964117226 0.9064299

4246.58770729387 0.8750389

4246.60577341547 0.8987048

4246.62383953707 0.9368455

4246.64190565867 0.9273272

4246.65997178028 0.9154356

4246.67803790188 0.951341

4246.69610402348 0.9447811

4246.71417014508 0.9289579

4246.73223626669 0.9285592

4246.75030238829 0.8971217

4246.76836850989 0.8862664

4246.78643463149 0.9185821

4246.8045007531 0.9205707

4246.8225668747 0.9018525

4246.8406329963 0.9061154

4246.8586991179 0.9314753

4246.8767652395 0.9270883

4246.89483136111 0.8793831

4246.91289748271 0.8907979

4246.93096360431 0.9216591

4246.94902972591 0.9404335

4246.96709584751 0.9533118

4246.98516196912 0.9347333

4247.00322809072 0.9600488

4247.02129421232 0.995955

4247.03936033392 0.9512393

4247.05742645553 0.9314643

4247.07549257713 0.9694537

4247.09355869873 0.9272581

4247.11162482033 0.8461851

4247.12969094193 0.8910087

4247.14775706354 0.9140501

4247.16582318514 0.8533133

4247.18388930674 0.8588035

4247.20195542834 0.9086754

4247.22002154995 0.9078315

4247.23808767155 0.9056501

4247.25615379315 0.9271519

4247.27421991475 0.9303366

4247.29228603635 0.9539912

4247.31035215796 0.9718039

4247.32841827956 0.9258111

4247.34648440116 0.9136394

4247.36455052276 0.8920212

4247.38261664437 0.8303213

4247.40068276597 0.8735878

4247.41874888757 0.935093

4247.43681500917 0.9290425

4247.45488113077 0.9254207

4247.47294725238 0.9569616

4247.49101337398 0.9641991

4247.50907949558 0.9171262

4247.52714561718 0.8960018

4247.54521173878 0.9093072

4247.56327786039 0.9056838

4247.58134398199 0.8794075

4247.59941010359 0.8914187

4247.61747622519 0.9713622

4247.6355423468 0.9657282

4247.6536084684 0.902366

4247.67167459 0.9209683

4247.6897407116 1.004816

4247.7078068332 0.9780478

4247.72587295481 0.9339979

4247.74393907641 0.9433679

4247.76200519801 0.9565375

4247.78007131961 0.9883556

4247.79813744122 1.000567

4247.81620356282 0.9481866

4247.83426968442 0.9471116

4247.85233580602 0.9956487

4247.87040192762 0.9616452

4247.88846804923 0.9472733

4247.90653417083 0.9490008

4247.92460029243 0.9583005

4247.94266641403 0.960143

4247.96073253564 0.93994

4247.97879865724 0.9044359

4247.99686477884 0.9110206

4248.01493090044 0.9610783

4248.03299702204 0.9761313

4248.05106314365 0.9609885

4248.06912926525 0.9577377

4248.08719538685 0.9736892

4248.10526150845 0.9694018

4248.12332763005 0.9389018

4248.14139375166 0.9414551

4248.15945987326 0.9604164

4248.17752599486 0.9532224

4248.19559211646 0.9509931

4248.21365823807 0.931018

4248.23172435967 0.9677252

4248.24979048127 0.9800756

4248.26785660287 0.9421459

4248.28592272447 0.9557039

4248.30398884608 1.00337

4248.32205496768 1.004716

4248.34012108928 1.001212

4248.35818721088 0.967827

4248.37625333249 0.9395787

4248.39431945409 0.9799607

4248.41238557569 0.9498894

4248.43045169729 0.9097299

4248.44851781889 0.957312

4248.4665839405 0.9780575

4248.4846500621 0.9478889

4248.5027161837 0.9696863

4248.5207823053 0.96323

4248.53884842691 0.9583469

4248.55691454851 0.9691734

4248.57498067011 0.9766541

4248.59304679171 0.9653958

4248.61111291331 0.9523014

4248.62917903492 0.9404151

4248.64724515652 0.922163

4248.66531127812 0.9330354

4248.68337739972 0.9608447

4248.70144352133 0.9354792

4248.71950964293 0.9230224

4248.73757576453 0.9494956

4248.75564188613 0.966452

4248.77370800773 0.9576161

4248.79177412934 0.9156372

4248.80984025094 0.9015812

4248.82790637254 0.8914772

4248.84597249414 0.9112031

4248.86403861574 0.9196751

4248.88210473735 0.9092237

4248.90017085895 0.8912656

4248.91823698055 0.9380291

4248.93630310215 0.9479237

4248.95436922376 0.9467943

4248.97243534536 0.9659424

4248.99050146696 0.9303066

4249.00856758856 0.9084613

4249.02663371017 0.9135096

4249.04469983177 0.892136

4249.06276595337 0.8831056

4249.08083207497 0.88156

4249.09889819657 0.8715659

4249.11696431817 0.8845672

4249.13503043978 0.9196135

4249.15309656138 0.8669308

4249.17116268298 0.8498037

4249.18922880458 0.9293786

4249.20729492619 0.9553772

4249.22536104779 0.949609

4249.24342716939 0.9412906

4249.26149329099 0.9176862

4249.27955941259 0.9209512

4249.2976255342 0.9317299

4249.3156916558 0.9228741

4249.3337577774 0.9317282

4249.351823899 0.9394724

4249.36989002061 0.9232009

4249.38795614221 0.9427502

4249.40602226381 0.9586163

4249.42408838541 0.9595548

4249.44215450702 0.9877723

4249.46022062862 1.00859

4249.47828675022 0.9828596

4249.49635287182 0.9533598

4249.51441899342 0.9833618

4249.53248511503 0.9788549

4249.55055123663 0.9335271

4249.56861735823 0.922267

4249.58668347983 0.9344393

4249.60474960144 0.9654574

4249.62281572304 0.9918125

4249.64088184464 0.9810119

4249.65894796624 0.9857223

4249.67701408784 1.009557

4249.69508020945 0.989467

4249.71314633105 0.9579257

4249.73121245265 0.962187

4249.74927857425 0.9768329

4249.76734469585 0.9605838

4249.78541081746 0.9450734

4249.80347693906 0.9534067

4249.82154306066 0.9218901

4249.83960918226 0.8891829

4249.85767530387 0.9452928

4249.87574142547 0.9444426

4249.89380754707 0.9289435

4249.91187366867 0.9412692

4249.92993979027 0.8684263

4249.94800591188 0.8875785

4249.96607203348 0.9407008

4249.98413815508 0.9339976

4250.00220427668 0.9181709

4250.02027039829 0.9197894

4250.03833651989 0.8972633

4250.05640264149 0.8936058

4250.07446876309 0.9134283

4250.09253488469 0.9344569

4250.1106010063 0.900991

4250.1286671279 0.9154568

4250.1467332495 0.9646928

4250.1647993711 0.9686118

4250.18286549271 0.9592665

4250.20093161431 0.9603758

4250.21899773591 0.9498413

4250.23706385751 0.9359496

4250.25512997911 0.9025307

4250.27319610072 0.8493155

4250.29126222232 0.8635134

4250.30932834392 0.9342253

4250.32739446552 0.9570556

4250.34546058712 0.9097978

4250.36352670873 0.9037573

4250.38159283033 0.9314911

4250.39965895193 0.9370259

4250.41772507353 0.9358128

4250.43579119514 0.9413279

4250.45385731674 0.9364966

4250.47192343834 0.8988334

4250.48998955994 0.8965886

4250.50805568154 0.9147629

4250.52612180315 0.8944259

4250.54418792475 0.9037503

4250.56225404635 0.9358363

4250.58032016795 0.8999326

4250.59838628956 0.8827779

4250.61645241116 0.9116724

4250.63451853276 0.9017595

4250.65258465436 0.8927882

4250.67065077596 0.8685468

4250.68871689757 0.8640537

4250.70678301917 0.8752992

4250.72484914077 0.8862022

4250.74291526237 0.8530242

4250.76098138397 0.8687957

4250.77904750558 0.8963944

4250.79711362718 0.8837796

4250.81517974878 0.8875532

4250.83324587038 0.912147

4250.85131199199 0.9091022

4250.86937811359 0.8910307

4250.88744423519 0.8930271

4250.90551035679 0.9177834

4250.92357647839 0.9281616

4250.9416426 0.9293714

4250.9597087216 0.9359927

4250.9777748432 0.9326546

4250.9958409648 0.9279345

4251.01390708641 0.9324613

4251.03197320801 0.9366959

4251.05003932961 0.9217613

4251.06810545121 0.9234995

4251.08617157282 0.9231185

4251.10423769442 0.9381343

4251.12230381602 0.9708138

4251.14036993762 0.9835224

4251.15843605922 0.974643

4251.17650218083 0.9710279

4251.19456830243 0.941987

4251.21263442403 0.9227762

4251.23070054563 0.9528278

4251.24876666724 0.9741414

4251.26683278884 0.9659444

4251.28489891044 0.9459665

4251.30296503204 0.9197032

4251.32103115364 0.9163941

4251.33909727525 0.9464862

4251.35716339685 0.9204234

4251.37522951845 0.9148675

4251.39329564005 0.9718965

4251.41136176166 0.9782908

4251.42942788326 0.9341914

4251.44749400486 0.9159214

4251.46556012646 0.9275903

4251.48362624806 0.9350368

4251.50169236967 0.9309499

4251.51975849127 0.9247934

4251.53782461287 0.9377964

4251.55589073447 0.9687982

4251.57395685608 0.9465756

4251.59202297768 0.9169122

4251.61008909928 0.9007008

4251.62815522088 0.9261099

4251.64622134248 0.9546896

4251.66428746409 0.9408094

4251.68235358569 0.9321803

4251.70041970729 0.9224185

4251.71848582889 0.9136959

4251.7365519505 0.8950727

4251.7546180721 0.8907044

4251.7726841937 0.9260201

4251.7907503153 0.9019793

4251.8088164369 0.8764656

4251.82688255851 0.8979466

4251.84494868011 0.8643088

4251.86301480171 0.8648282

4251.88108092331 0.8928696

4251.89914704492 0.9095935

4251.91721316652 0.9073684

4251.93527928812 0.8736789

4251.95334540972 0.8742248

4251.97141153132 0.9312568

4251.98947765293 0.9403232

4252.00754377453 0.9105157

4252.02560989613 0.955878

4252.04367601773 0.943203

4252.06174213933 0.922263

4252.07980826094 0.8955032

4252.09787438254 0.8881665

4252.11594050414 0.9197646

4252.13400662574 0.9303798

4252.15207274735 0.9088119

4252.17013886895 0.9030708

4252.18820499055 0.8893743

4252.20627111215 0.9318551

4252.22433723375 0.9636523

4252.24240335536 0.9566202

4252.26046947696 0.9306905

4252.27853559856 0.9071933

4252.29660172016 0.9010601

4252.31466784176 0.9153676

4252.33273396337 0.9221866

4252.35080008497 0.9153962

4252.36886620657 0.9405769

4252.38693232817 0.9483418

4252.40499844978 0.9254529

4252.42306457138 0.9477446

4252.44113069298 0.9504368

4252.45919681458 0.9254091

4252.47726293618 0.9079906

4252.49532905779 0.8840806

4252.51339517939 0.8702009

4252.53146130099 0.8864824

4252.54952742259 0.9033116

4252.5675935442 0.9052484

4252.5856596658 0.8715626

4252.6037257874 0.8235298

4252.621791909 0.8385151

4252.6398580306 0.8617823

4252.65792415221 0.8551133

4252.67599027381 0.8827837

4252.69405639541 0.922559

4252.71212251701 0.9112313

4252.73018863861 0.8742023

4252.74825476022 0.8998218

4252.76632088182 0.8904977

4252.78438700342 0.8928288

4252.80245312502 0.8767154

4252.82051924663 0.8659817

4252.83858536823 0.8874975

4252.85665148983 0.882382

4252.87471761143 0.8484695

4252.89278373304 0.84153

4252.91084985464 0.8628291

4252.92891597624 0.882677

4252.94698209784 0.8670607

4252.96504821944 0.8240994

4252.98311434105 0.8197712

4253.00118046265 0.8642054

4253.01924658425 0.8794421

4253.03731270585 0.8721097

4253.05537882745 0.8665674

4253.07344494906 0.8899271

4253.09151107066 0.9008445

4253.10957719226 0.8863825

4253.12764331386 0.8822255

4253.14570943547 0.8945063

4253.16377555707 0.8864686

4253.18184167867 0.9087869

4253.19990780027 0.9207752

4253.21797392188 0.9076391

4253.23604004348 0.9291052

4253.25410616508 0.9398971

4253.27217228668 0.9446387

4253.29023840828 0.9591018

4253.30830452988 0.9543638

4253.32637065149 0.9264611

4253.34443677309 0.9340774

4253.36250289469 0.9431198

4253.38056901629 0.9246408

4253.3986351379 0.8737827

4253.4167012595 0.8830184

4253.4347673811 0.9167798

4253.4528335027 0.9288599

4253.47089962431 0.9344202

4253.48896574591 0.9210741

4253.50703186751 0.9384346

4253.52509798911 0.9211442

4253.54316411071 0.9073853

4253.56123023232 0.9194002

4253.57929635392 0.9327931

4253.59736247552 0.9387894

4253.61542859712 0.9268779

4253.63349471873 0.9081414

4253.65156084033 0.9285625

4253.66962696193 0.9116703

4253.68769308353 0.9474779

4253.70575920513 0.983644

4253.72382532674 0.9511993

4253.74189144834 0.935873

4253.75995756994 0.9436486

4253.77802369154 0.9562817

4253.79608981315 0.9449207

4253.81415593475 0.9049237

4253.83222205635 0.921607

4253.85028817795 0.9565171

4253.86835429955 0.9689466

4253.88642042116 0.9890754

4253.90448654276 0.9544967

4253.92255266436 0.9479706

4253.94061878596 0.9723021

4253.95868490756 0.9383464

4253.97675102917 0.9268206

4253.99481715077 0.9574847

4254.01288327237 0.9981476

4254.03094939397 0.972801

4254.04901551558 0.9427303

4254.06708163718 0.9427658

4254.08514775878 0.9351394

4254.10321388038 0.9457228

4254.12128000198 0.9519477

4254.13934612359 0.9515629

4254.15741224519 0.9625696

4254.17547836679 0.9524616

4254.19354448839 0.9398829

4254.21161061 0.9534217

4254.2296767316 0.9710212

4254.2477428532 0.9740578

4254.2658089748 1.001312

4254.2838750964 0.9600886

4254.30194121801 0.9514595

4254.32000733961 0.9394493

4254.33807346121 0.9283876

4254.35613958281 0.9475229

4254.37420570442 0.925355

4254.39227182602 0.9371113

4254.41033794762 0.9797283

4254.42840406922 0.9765627

4254.44647019082 0.9802542

4254.46453631243 0.970622

4254.48260243403 0.9516947

4254.50066855563 0.9534621

4254.51873467723 0.9388267

4254.53680079884 0.936659

4254.55486692044 0.9539042

4254.57293304204 0.9508791

4254.59099916364 0.9574597

4254.60906528524 1.002157

4254.62713140685 0.9951269

4254.64519752845 0.9772

4254.66326365005 0.9953942

4254.68132977165 1.008111

4254.69939589326 0.9810134

4254.71746201486 0.9533814

4254.73552813646 0.9504418

4254.75359425806 0.9276339

4254.77166037966 0.9218134

4254.78972650127 0.9428413

4254.80779262287 0.9540536

4254.82585874447 0.9270218

4254.84392486607 0.9229211

4254.86199098767 0.9509629

4254.88005710928 0.9585328

4254.89812323088 0.957567

4254.91618935248 0.939741

4254.93425547408 0.9358014

4254.95232159569 0.9596477

4254.97038771729 0.9263263

4254.98845383889 0.9254795

4255.00651996049 0.9061673

4255.02458608209 0.9214908

4255.0426522037 0.9483154

4255.0607183253 0.9057728

4255.0787844469 0.9178721

4255.0968505685 0.9597688

4255.11491669011 0.9468849

4255.13298281171 0.9724522

4255.15104893331 0.9869764

4255.16911505491 0.9744872

4255.18718117651 0.9535558

4255.20524729812 0.9923031

4255.22331341972 0.9972628

4255.24137954132 0.9865557

4255.25944566292 0.9765858

4255.27751178453 0.9819657

4255.29557790613 0.9830757

4255.31364402773 0.9581002

4255.33171014933 0.9659855

4255.34977627093 0.9598204

4255.36784239254 0.9596171

4255.38590851414 0.962349

4255.40397463574 0.9743098

4255.42204075734 0.9945029

4255.44010687895 0.9792598

4255.45817300055 1.002827

4255.47623912215 1.008208

4255.49430524375 0.9705132

4255.51237136535 0.977843

4255.53043748695 1.014336

4255.54850360856 1.019866

4255.56656973016 0.9841169

4255.58463585176 0.9512398

4255.60270197336 0.9514138

4255.62076809497 0.972371

4255.63883421657 0.9344872

4255.65690033817 0.914417

4255.67496645977 0.9545673

4255.69303258137 0.9651805

4255.71109870298 0.9552332

4255.72916482458 0.9605982

4255.74723094618 0.9078006

4255.76529706778 0.8930935

4255.78336318939 0.9101724

4255.80142931099 0.910642

4255.81949543259 0.9251002

4255.83756155419 0.8997675

4255.8556276758 0.9047107

4255.8736937974 0.9093026

4255.891759919 0.9138538

4255.9098260406 0.9316382

4255.9278921622 0.9247427

4255.94595828381 0.9209521

4255.96402440541 0.9392053

4255.98209052701 0.9511727

4256.00015664861 0.9269395

4256.01822277022 0.9166973

4256.03628889182 0.9249488

4256.05435501342 0.9184641

4256.07242113502 0.9280595

4256.09048725662 0.9301109

4256.10855337823 0.9209232

4256.12661949983 0.9359116

4256.14468562143 0.9341924

4256.16275174303 0.9197319

4256.18081786464 0.9403028

4256.19888398624 0.9314206

4256.21695010784 0.9325231

4256.23501622944 0.9512777

4256.25308235104 0.9414474

4256.27114847265 0.9241136

4256.28921459425 0.951208

4256.30728071585 0.9209496

4256.32534683745 0.9078737

4256.34341295906 0.9328731

4256.36147908066 0.9451416

4256.37954520226 0.9164628

4256.39761132386 0.9180948

4256.41567744547 0.9414541

4256.43374356707 0.9585222

4256.45180968867 0.9339534

4256.46987581027 0.9014351

4256.48794193187 0.911625

4256.50600805347 0.9284977

4256.52407417508 0.9224659

4256.54214029668 0.9200939

4256.56020641828 0.9407176

4256.57827253988 0.9517642

4256.59633866149 0.9472686

4256.61440478309 0.9427418

4256.63247090469 0.9164576

4256.65053702629 0.927016

4256.66860314789 0.950727

4256.6866692695 0.9383423

4256.7047353911 0.9485737

4256.7228015127 0.9819453

4256.7408676343 0.9812943

4256.75893375591 0.9452268

4256.77699987751 0.9546217

4256.79506599911 1.027779

4256.81313212071 1.009981

4256.83119824231 0.9446173

4256.84926436392 0.9412999

4256.86733048552 0.955066

4256.88539660712 0.9349746

4256.90346272872 0.9732755

4256.92152885033 0.9889314

4256.93959497193 0.9837213

4256.95766109353 0.9786244

4256.97572721513 0.9440629

4256.99379333673 0.9624978

4257.01185945834 0.9986507

4257.02992557994 0.9982241

4257.04799170154 0.9784489

4257.06605782314 0.9732946

4257.08412394475 1.006154

4257.10219006635 0.9982431

4257.12025618795 0.9832017

4257.13832230955 0.9812117

4257.15638843115 0.9818422

4257.17445455276 0.9784728

4257.19252067436 0.9785095

4257.21058679596 1.000536

4257.22865291756 1.007756

4257.24671903916 0.9987638

4257.26478516077 0.9798387

4257.28285128237 0.9904922

4257.30091740397 0.9860409

4257.31898352557 0.95802

4257.33704964718 0.9633595

4257.35511576878 0.9844867

4257.37318189038 0.9939663

4257.39124801198 1.006409

4257.40931413358 1.003486

4257.42738025519 0.9831838

4257.44544637679 0.9694433

4257.46351249839 0.9889177

4257.48157861999 1.00679

4257.49964474159 1.005919

4257.5177108632 1.005635

4257.5357769848 0.9966806

4257.5538431064 1.007545

4257.571909228 0.9857829

4257.58997534961 0.9722058

4257.60804147121 0.976588

4257.62610759281 0.9500359

4257.64417371441 0.9565089

4257.66223983601 0.9499568

4257.68030595762 0.9638436

4257.69837207922 1.008744

4257.71643820082 1.003208

4257.73450432242 0.9895039

4257.75257044403 0.9933985

4257.77063656563 1.012793

4257.78870268723 1.002603

4257.80676880883 0.9797103

4257.82483493044 0.9710477

4257.84290105204 0.9363294

4257.86096717364 0.9391997

4257.87903329524 0.9492234

4257.89709941684 0.9565154

4257.91516553845 0.9476768

4257.93323166005 0.8976729

4257.95129778165 0.9219089

4257.96936390325 0.9613148

4257.98743002486 0.9425042

4258.00549614646 0.9422966

4258.02356226806 0.9633579

4258.04162838966 0.9395202

4258.05969451126 0.9250205

4258.07776063287 0.9436209

4258.09582675447 0.9399167

4258.11389287607 0.9511952

4258.13195899767 0.9276155

4258.15002511927 0.9062512

4258.16809124088 0.9056643

4258.18615736248 0.8965217

4258.20422348408 0.8916179

4258.22228960568 0.8860672

4258.24035572729 0.8863324

4258.25842184889 0.8902103

4258.27648797049 0.8711278

4258.29455409209 0.8627433

4258.31262021369 0.8719792

4258.3306863353 0.8530191

4258.3487524569 0.8538629

4258.3668185785 0.8810059

4258.3848847001 0.8955466

4258.40295082171 0.8965631

4258.42101694331 0.8828762

4258.43908306491 0.8727931

4258.45714918651 0.8850067

4258.47521530811 0.8997235

4258.49328142972 0.9075617

4258.51134755132 0.8941968

4258.52941367292 0.8845946

4258.54747979452 0.901305

4258.56554591613 0.9014778

4258.58361203773 0.9346429

4258.60167815933 0.9379836

4258.61974428093 0.9239373

4258.63781040253 0.9689593

4258.65587652414 0.9580299

4258.67394264574 0.937391

4258.69200876734 0.9428334

4258.71007488894 0.9437181

4258.72814101054 0.9373488

4258.74620713215 0.9391744

4258.76427325375 0.9595987

4258.78233937535 0.9791923

4258.80040549695 0.9880959

4258.81847161856 1.001784

4258.83653774016 0.9996451

4258.85460386176 0.9957716

4258.87266998336 0.9831614

4258.89073610496 0.9688331

4258.90880222657 0.9862292

4258.92686834817 1.006544

4258.94493446977 0.9862582

4258.96300059137 0.988116

4258.98106671298 0.9678259

4258.99913283458 0.9621837

4259.01719895618 0.9499537

4259.03526507778 0.9437219

4259.05333119939 0.9726298

4259.07139732099 1.002352

4259.08946344259 0.9526032

4259.10752956419 0.9286659

4259.12559568579 0.9417583

4259.14366180739 0.9349194

4259.161727929 0.9211656

4259.1797940506 0.9254544

4259.1978601722 0.9551619

4259.2159262938 0.9701494

4259.23399241541 0.92834

4259.25205853701 0.8864188

4259.27012465861 0.9240073

4259.28819078021 0.9346287

4259.30625690182 0.9012135

4259.32432302342 0.903244

4259.34238914502 0.9489558

4259.36045526662 0.951912

4259.37852138822 0.9043621

4259.39658750983 0.8769634

4259.41465363143 0.8913549

4259.43271975303 0.8703001

4259.45078587463 0.8647197

4259.46885199624 0.9125941

4259.48691811784 0.9083605

4259.50498423944 0.9059755

4259.52305036104 0.9129806

4259.54111648264 0.9066001

4259.55918260425 0.8817505

4259.57724872585 0.8765051

4259.59531484745 0.9317714

4259.61338096905 0.9478769

4259.63144709065 0.9268508

4259.64951321226 0.9093675

4259.66757933386 0.9239047

4259.68564545546 0.9424571

4259.70371157706 0.9206789

4259.72177769867 0.9316747

4259.73984382027 0.9306285

4259.75790994187 0.9368858

4259.77597606347 0.9550635

4259.79404218507 0.9625779

4259.81210830668 0.9852852

4259.83017442828 0.9576745

4259.84824054988 0.9622742

4259.86630667148 0.9975271

4259.88437279309 0.9877173

4259.90243891469 0.9707813

4259.92050503629 0.9664559

4259.93857115789 0.9638485

4259.95663727949 0.9629521

4259.9747034011 0.9726058

4259.9927695227 0.9883249

4260.0108356443 0.9999521

4260.0289017659 0.9885563

4260.04696788751 0.9872464

4260.06503400911 1.002617

4260.08310013071 1.0095

4260.10116625231 1.007442

4260.11923237391 0.9981139

4260.13729849552 0.9947765

4260.15536461712 1.016484

4260.17343073872 0.9951019

4260.19149686032 0.9888883

4260.20956298193 1.000257

4260.22762910353 1.012489

4260.24569522513 0.9983563

4260.26376134673 1.002963

4260.28182746834 1.036915

4260.29989358994 1.013746

4260.31795971154 0.9818584

4260.33602583314 0.9604191

4260.35409195474 0.9612052

4260.37215807635 0.9396772

4260.39022419795 0.9734781

4260.40829031955 0.9967904

4260.42635644115 0.9946936

4260.44442256275 0.9730031

4260.46248868436 0.9715227

4260.48055480596 0.9677171

4260.49862092756 0.9308035

4260.51668704916 0.9033852

4260.53475317077 0.9131763

4260.55281929237 0.9415331

4260.57088541397 0.9479424

4260.58895153557 0.9282742

4260.60701765717 0.9498693

4260.62508377878 0.9902558

4260.64314990038 0.9761503

4260.66121602198 0.9313939

4260.67928214358 0.9226997

4260.69734826518 0.9812099

4260.71541438679 0.9890975

4260.73348050839 0.9372311

4260.75154662999 0.9198218

4260.76961275159 0.8983176

4260.7876788732 0.9070883

4260.8057449948 0.9402437

4260.8238111164 0.9262339

4260.841877238 0.9486468

4260.8599433596 0.9570701

4260.87800948121 0.9683929

4260.89607560281 0.9322834

4260.91414172441 0.8983334

4260.93220784601 0.9225571

4260.95027396762 0.9303046

4260.96834008922 0.9214113

4260.98640621082 0.9303401

4261.00447233242 0.9678029

4261.02253845402 0.956284

4261.04060457563 0.9516618

4261.05867069723 0.978221

4261.07673681883 0.9658011

4261.09480294043 0.9454358

4261.11286906204 0.9368494

4261.13093518364 0.9320976

4261.14900130524 0.9541389

4261.16706742684 0.9449579

4261.18513354844 0.9258885

4261.20319967005 0.9367005

4261.22126579165 0.9403031

4261.23933191325 0.9699916

4261.25739803485 0.9800241

4261.27546415646 0.9238566

4261.29353027806 0.9206537

4261.31159639966 0.9539672

4261.32966252126 0.9575627

4261.34772864286 0.9349579

4261.36579476447 0.933558

4261.38386088607 0.9794366

4261.40192700767 0.9786156

4261.41999312927 0.9521005

4261.43805925088 0.9473219

4261.45612537248 0.9484048

4261.47419149408 0.9369695

4261.49225761568 0.9503019

4261.51032373728 0.9232355

4261.52838985889 0.902213

4261.54645598049 0.9302231

4261.56452210209 0.9414446

4261.58258822369 0.9882365

4261.6006543453 0.9772753

4261.6187204669 0.9372069

4261.6367865885 0.9335105

4261.6548527101 0.9401237

4261.6729188317 0.9639189

4261.69098495331 0.9437221

4261.70905107491 0.8926835

4261.72711719651 0.9129558

4261.74518331811 0.9196823

4261.76324943971 0.9150041

4261.78131556132 0.8714629

4261.79938168292 0.8745846

4261.81744780452 0.8615752

4261.83551392612 0.8744661

4261.85358004773 0.8808988

4261.87164616933 0.8772346

4261.88971229093 0.8831242

4261.90777841253 0.85927

4261.92584453413 0.8809869

4261.94391065574 0.8823403

4261.96197677734 0.8231719

4261.98004289894 0.7761804

4261.99810902054 0.7837275

4262.01617514215 0.8093388

4262.03424126375 0.8274789

4262.05230738535 0.8153502

4262.07037350695 0.7761811

4262.08843962855 0.7833138

4262.10650575016 0.8121485

4262.12457187176 0.8280903

4262.14263799336 0.7924483

4262.16070411496 0.7724846

4262.17877023657 0.8225498

4262.19683635817 0.83272

4262.21490247977 0.7950522

4262.23296860137 0.7915457

4262.25103472297 0.8298643

4262.26910084458 0.8531399

4262.28716696618 0.8289322

4262.30523308778 0.8197155

4262.32329920938 0.8262917

4262.34136533098 0.828303

4262.35943145259 0.8117292

4262.37749757419 0.8132306

4262.39556369579 0.8316511

4262.41362981739 0.8665235

4262.431695939 0.8807579

4262.4497620606 0.8531008

4262.4678281822 0.8263608

4262.4858943038 0.8501341

4262.5039604254 0.8889862

4262.52202654701 0.9009645

4262.54009266861 0.9506699

4262.55815879021 0.9757079

4262.57622491181 0.9543924

4262.59429103342 0.9211919

4262.61235715502 0.9359957

4262.63042327662 0.9154158

4262.64848939822 0.9350899

4262.66655551982 0.9700695

4262.68462164143 0.9956158

4262.70268776303 0.961007

4262.72075388463 0.9275073

4262.73882000623 0.9625666

4262.75688612783 0.969892

4262.77495224944 0.9882193

4262.79301837104 0.9779616

4262.81108449264 0.9534183

4262.82915061424 0.9481022

4262.84721673585 0.934697

4262.86528285745 0.9529207

4262.88334897905 0.9771374

4262.90141510065 0.9506029

4262.91948122225 0.9158895

4262.93754734386 0.8953998

4262.95561346546 0.9117585

4262.97367958706 0.9529706

4262.99174570866 0.944491

4263.00981183027 0.9223799

4263.02787795187 0.9463508

4263.04594407347 0.9636443

4263.06401019507 0.9610255

4263.08207631667 0.9769111

4263.10014243828 0.9677678

4263.11820855988 0.9675024

4263.13627468148 0.9709095

4263.15434080308 0.9486036

4263.17240692469 0.9417892

4263.19047304629 0.943951

4263.20853916789 0.946514

4263.22660528949 0.9351035

4263.2446714111 0.9405572

4263.2627375327 0.9282331

4263.2808036543 0.9098386

4263.2988697759 0.8926821

4263.3169358975 0.8979685

4263.33500201911 0.9501377

4263.35306814071 0.9501281

4263.37113426231 0.9222935

4263.38920038391 0.9367706

4263.40726650551 0.9422798

4263.42533262712 0.9344475

4263.44339874872 0.9576147

4263.46146487032 0.9303445

4263.47953099192 0.885988

4263.49759711353 0.9101714

4263.51566323513 0.9352769

4263.53372935673 0.9669827

4263.55179547833 0.9718192

4263.56986159994 0.9108158

4263.58792772154 0.9049014

4263.60599384314 0.9481854

4263.62405996474 0.9550995

4263.64212608634 0.9193429

4263.66019220795 0.9278384

4263.67825832955 0.9794514

4263.69632445115 0.9854859

4263.71439057275 0.9371582

4263.73245669435 0.9071823

4263.75052281596 0.8760816

4263.76858893756 0.844914

4263.78665505916 0.8559563

4263.80472118076 0.9094123

4263.82278730237 0.8929599

4263.84085342397 0.8586451

4263.85891954557 0.8778294

4263.87698566717 0.8930409

4263.89505178877 0.8972718

4263.91311791037 0.9225892

4263.93118403198 0.9216984

4263.94925015358 0.8948442

4263.96731627518 0.8851988

4263.98538239678 0.892087

4264.00344851839 0.8865643

4264.02151463999 0.8955814

4264.03958076159 0.8996509

4264.05764688319 0.908358

4264.0757130048 0.9226223

4264.0937791264 0.915659

4264.111845248 0.9136281

4264.1299113696 0.8979517

4264.1479774912 0.8916613

4264.16604361281 0.8751017

4264.18410973441 0.8835831

4264.20217585601 0.8974527

4264.22024197761 0.8904695

4264.23830809922 0.8670932

4264.25637422082 0.9087145

4264.27444034242 0.9095041

4264.29250646402 0.9184295

4264.31057258562 0.8997759

4264.32863870723 0.9223075

4264.34670482883 0.9455011

4264.36477095043 0.9413837

4264.38283707203 0.9297612

4264.40090319364 0.9080767

4264.41896931524 0.9228145

4264.43703543684 0.9203928

4264.45510155844 0.9238646

4264.47316768004 0.9379843

4264.49123380165 0.9136055

4264.50929992325 0.9181501

4264.52736604485 0.949874

4264.54543216645 0.982951

4264.56349828805 0.9785357

4264.58156440966 0.9346877

4264.59963053126 0.9156676

4264.61769665286 0.8997111

4264.63576277446 0.9123185

4264.65382889607 0.9199288

4264.67189501767 0.938265

4264.68996113927 0.936076

4264.70802726087 0.9167451

4264.72609338247 0.9056313

4264.74415950408 0.9056841

4264.76222562568 0.9192161

4264.78029174728 0.93137

4264.79835786888 0.9495754

4264.81642399049 0.9553535

4264.83449011209 0.9394263

4264.85255623369 0.9149032

4264.87062235529 0.9420331

4264.88868847689 0.954945

4264.9067545985 0.9389048

4264.9248207201 0.9605792

4264.9428868417 0.9465537

4264.9609529633 0.9502813

4264.97901908491 0.9331769

4264.99708520651 0.9070494

4265.01515132811 0.9204538

4265.03321744971 0.9403077

4265.05128357131 0.9319903

4265.06934969292 0.9124628

4265.08741581452 0.9075885

4265.10548193612 0.9138945

4265.12354805772 0.9134107

4265.14161417933 0.9345415

4265.15968030093 0.9813243

4265.17774642253 0.9845618

4265.19581254413 0.9632484

4265.21387866574 0.9449829

4265.23194478734 0.9363307

4265.25001090894 0.950858

4265.26807703054 0.939217

4265.28614315214 0.9462347

4265.30420927375 0.9557101

4265.32227539535 0.9605544

4265.34034151695 0.9808134

4265.35840763855 0.935454

4265.37647376015 0.9132842

4265.39453988176 0.94403

4265.41260600336 0.9465486

4265.43067212496 0.9889749

4265.44873824656 0.9929519

4265.46680436817 0.9488727

4265.48487048977 0.9304041

4265.50293661137 0.9088019

4265.52100273297 0.9210931

4265.53906885457 0.9448802

4265.55713497618 0.9516326

4265.57520109778 0.9831775

4265.59326721938 0.9466964

4265.61133334098 0.9214029

4265.62939946258 0.9759663

4265.64746558419 1.006658

4265.66553170579 0.9649929

4265.68359782739 0.9222646

4265.70166394899 0.9679995

4265.7197300706 0.984454

4265.7377961922 0.9702227

4265.7558623138 0.9599478

4265.7739284354 0.9492808

4265.791994557 0.954214

4265.81006067861 0.9742002

4265.82812680021 0.9911197

4265.84619292181 0.9996414

4265.86425904341 0.9686943

4265.88232516501 0.9483485

4265.90039128662 0.9621069

4265.91845740822 0.9533925

4265.93652352982 0.9543834

4265.95458965142 0.9232323

4265.97265577303 0.8996643

4265.99072189463 0.9165468

4266.00878801623 0.9141161

4266.02685413783 0.886483

4266.04492025944 0.9287928

4266.06298638104 0.9521706

4266.08105250264 0.9241447

4266.09911862424 0.910081

4266.11718474584 0.8921217

4266.13525086745 0.9125577

4266.15331698905 0.9247812

4266.17138311065 0.9101151

4266.18944923225 0.8938259

4266.20751535386 0.8746777

4266.22558147546 0.9159984

4266.24364759706 0.9353248

4266.26171371866 0.887313

4266.27977984026 0.8791705

4266.29784596187 0.9193702

4266.31591208347 0.9439205

4266.33397820507 0.923618

4266.35204432667 0.9049371

4266.37011044828 0.9029397

4266.38817656988 0.9106247

4266.40624269148 0.8915192

4266.42430881308 0.8758454

4266.44237493468 0.8773544

4266.46044105629 0.8564633

4266.47850717789 0.8835549

4266.49657329949 0.9066179

4266.51463942109 0.9216561

4266.53270554269 0.9119898

4266.5507716643 0.9351211

4266.5688377859 0.9059173

4266.5869039075 0.8218622

4266.6049700291 0.8688375

4266.62303615071 0.8773503

4266.64110227231 0.8657677

4266.65916839391 0.8920835

4266.67723451551 0.8563001

4266.69530063711 0.8515505

4266.71336675872 0.8777274

4266.73143288032 0.8944831

4266.74949900192 0.8964999

4266.76756512352 0.8880427

4266.78563124513 0.8776705

4266.80369736673 0.9039526

4266.82176348833 0.9328369

4266.83982960993 0.9144641

4266.85789573153 0.8877373

4266.87596185314 0.8978904

4266.89402797474 0.9197217

4266.91209409634 0.9035993

4266.93016021794 0.8637543

4266.94822633954 0.8733386

4266.96629246115 0.9077178

4266.98435858275 0.8992552

4267.00242470435 0.9084999

4267.02049082595 0.8693436

4267.03855694756 0.906161

4267.05662306916 0.9226298

4267.07468919076 0.9002352

4267.09275531236 0.8681991

4267.11082143396 0.8770411

4267.12888755557 0.9077983

4267.14695367717 0.8917943

4267.16501979877 0.8638431

4267.18308592037 0.9041312

4267.20115204198 0.9032288

4267.21921816358 0.8981101

4267.23728428518 0.8605579

4267.25535040678 0.8208252

4267.27341652838 0.8507711

4267.29148264999 0.8650355

4267.30954877159 0.8488361

4267.32761489319 0.8354995

4267.34568101479 0.8401765

4267.3637471364 0.8520044

4267.381813258 0.8333418

4267.3998793796 0.7998201

4267.4179455012 0.8081781

4267.4360116228 0.8461548

4267.45407774441 0.8521401

4267.47214386601 0.8818477

4267.49020998761 0.8981355

4267.50827610921 0.8540258

4267.52634223081 0.8688431

4267.54440835242 0.8952672

4267.56247447402 0.9013076

4267.58054059562 0.8574598

4267.59860671722 0.8603262

4267.61667283883 0.8532801

4267.63473896043 0.8808894

4267.65280508203 0.9122501

4267.67087120363 0.9140654

4267.68893732523 0.9164531

4267.70700344684 0.9327691

4267.72506956844 0.9278896

4267.74313569004 0.9269032

4267.76120181164 0.9146237

4267.77926793325 0.9113803

4267.79733405485 0.9316351

4267.81540017645 0.9556383

4267.83346629805 0.9542539

4267.85153241966 0.9619008

4267.86959854126 0.9253026

4267.88766466286 0.9691768

4267.90573078446 0.9652261

4267.92379690606 0.9484025

4267.94186302767 0.9325168

4267.95992914927 0.9856614

4267.97799527087 1.017322

4267.99606139247 0.9777598

4268.01412751408 0.9568818

4268.03219363568 0.9525114

4268.05025975728 0.9092168

4268.06832587888 0.9427905

4268.08639200048 0.9739206

4268.10445812209 0.9662786

4268.12252424369 0.9718853

4268.14059036529 0.9580486

4268.15865648689 0.9280136

4268.17672260849 0.9332908

4268.1947887301 0.9934881

4268.2128548517 1.000598

4268.2309209733 0.9105887

4268.2489870949 0.8948729

4268.26705321651 0.9666218

4268.28511933811 0.9554189

4268.30318545971 0.954673

4268.32125158131 0.9620417

4268.33931770292 0.9812156

4268.35738382452 0.9763865

4268.37544994612 0.9262857

4268.39351606772 0.9308327

4268.41158218932 0.9484302

4268.42964831093 0.9460701

4268.44771443253 0.929991

4268.46578055413 0.9454365

4268.48384667573 0.9458842

4268.50191279733 0.9880028

4268.51997891894 0.9923369

4268.53804504054 0.9367718

4268.55611116214 0.9408899

4268.57417728374 0.9638896

4268.59224340535 0.9778715

4268.61030952695 0.9452895

4268.62837564855 0.9400233

4268.64644177015 0.9468131

4268.66450789175 0.9478486

4268.68257401336 0.9752616

4268.70064013496 0.9531474

4268.71870625656 0.9252076

4268.73677237816 0.9329697

4268.75483849977 0.9645586

4268.77290462137 0.9462167

4268.79097074297 0.9309847

4268.80903686457 0.9106053

4268.82710298617 0.9187008

4268.84516910778 0.9692068

4268.86323522938 1.023781

4268.88130135098 0.9973673

4268.89936747258 0.970068

4268.91743359418 1.000064

4268.93549971579 1.011094

4268.95356583739 0.9814622

4268.97163195899 1.003489

4268.98969808059 1.043123

4269.0077642022 1.050091

4269.0258303238 1.005692

4269.0438964454 0.9400996

4269.061962567 0.8946279

4269.0800286886 0.9014615

4269.09809481021 0.9439399

4269.11616093181 0.9400716

4269.13422705341 0.9234053

4269.15229317501 0.9158542

4269.17035929661 0.9320737

4269.18842541822 0.9271889

4269.20649153982 0.9018602

4269.22455766142 0.9405865

4269.24262378302 0.9845345

4269.26068990463 0.9748228

4269.27875602623 0.9470793

4269.29682214783 0.9248142

4269.31488826943 0.9599965

4269.33295439104 0.983496

4269.35102051264 0.9303963

4269.36908663424 0.8952093

4269.38715275584 0.9204886

4269.40521887744 0.95693

4269.42328499905 0.9273049

4269.44135112065 0.9568763

4269.45941724225 0.9632365

4269.47748336385 0.9320092

4269.49554948545 0.9705632

4269.51361560706 1.01576

4269.53168172866 0.9697421

4269.54974785026 0.8920695

4269.56781397186 0.9299088

4269.58588009347 0.9518653

4269.60394621507 0.9458648

4269.62201233667 0.9273698

4269.64007845827 0.9188259

4269.65814457987 0.9606034

4269.67621070148 0.9367494

4269.69427682308 0.865394

4269.71234294468 0.8917682

4269.73040906628 0.9703233

4269.74847518789 0.9712516

4269.76654130949 0.9115896

4269.78460743109 0.9048903

4269.80267355269 0.9600857

4269.82073967429 0.9682438

4269.8388057959 0.9432912

4269.8568719175 0.911536

4269.8749380391 0.9695165

4269.8930041607 1.003774

4269.91107028231 0.9862878

4269.92913640391 1.03276

4269.94720252551 0.9885337

4269.96526864711 0.9560503

4269.98333476872 0.9679032

4270.00140089032 0.9793062

4270.01946701192 1.016002

4270.03753313352 1.009897

4270.05559925512 1.006514

4270.07366537672 0.963989

4270.09173149833 0.9530313

4270.10979761993 0.9268864

4270.12786374153 0.9357892

4270.14592986313 0.9943165

4270.16399598474 1.01396

4270.18206210634 1.012073

4270.20012822794 1.002931

4270.21819434954 0.9905884

4270.23626047115 0.973137

4270.25432659275 0.9391688

4270.27239271435 0.9262418

4270.29045883595 0.9840924

4270.30852495755 0.9784317

4270.32659107916 0.9639909

4270.34465720076 0.9696789

4270.36272332236 0.9749799

4270.38078944396 0.9573622

4270.39885556557 0.9594789

4270.41692168717 0.9796345

4270.43498780877 0.9731818

4270.45305393037 0.9924676

4270.47112005197 0.92264

4270.48918617358 0.9182447

4270.50725229518 0.9180322

4270.52531841678 0.9231888

4270.54338453838 0.9929392

4270.56145065999 1.00131

4270.57951678159 0.959267

4270.59758290319 0.9330589

4270.61564902479 0.8908044

4270.63371514639 0.9071016

4270.651781268 0.9236425

4270.6698473896 0.9563626

4270.6879135112 0.9539965

4270.7059796328 0.9238696

4270.7240457544 0.9363799

4270.74211187601 0.9159273

4270.76017799761 0.94662

4270.77824411921 0.863868

4270.79631024081 0.8771355

4270.81437636242 0.9828988

4270.83244248402 0.9097936

4270.85050860562 0.8452559

4270.86857472722 0.965135

4270.88664084882 0.9857053

4270.90470697043 0.8921142

4270.92277309203 0.8859891

4270.94083921363 0.9082044

4270.95890533523 0.8639166

4270.97697145684 0.8449177

4270.99503757844 0.8806733

4271.01310370004 0.9122809

4271.03116982164 0.8739017

4271.04923594324 0.8831313

4271.06730206485 0.9790971

4271.08536818645 0.9986417

4271.10343430805 0.9335676

4271.12150042965 0.900712

4271.13956655125 0.9135807

4271.15763267286 0.9463581

4271.17569879446 0.9582554

4271.19376491606 0.9681528

4271.21183103766 0.9739848

4271.22989715927 0.9285779

4271.24796328087 0.9311209

4271.26602940247 0.9758848

4271.28409552407 0.9541568

4271.30216164567 0.9408562

4271.32022776728 0.9074523

4271.33829388888 0.8920648

4271.35636001048 0.9449393

4271.37442613208 0.9687082

4271.39249225369 0.9443189

4271.41055837529 0.9719717

4271.42862449689 0.9223878

4271.44669061849 0.887396

4271.46475674009 0.9663054

4271.4828228617 1.009174

4271.5008889833 1.003533

4271.5189551049 0.9837261

4271.5370212265 0.9823998

4271.55508734811 0.9960885

4271.57315346971 0.9272225

4271.59121959131 0.9508952

4271.60928571291 1.034235

4271.62735183452 0.9558624

4271.64541795612 0.9441744

4271.66348407772 0.9695661

4271.68155019932 0.9297091

4271.69961632092 0.8924768

4271.71768244252 0.8879712

4271.73574856413 0.9863949

4271.75381468573 0.9810293

4271.77188080733 0.9619832

4271.78994692893 0.9930713

4271.80801305054 0.9701817

4271.82607917214 0.9067667

4271.84414529374 0.8834559

4271.86221141534 0.9353241

4271.88027753694 0.9660102

4271.89834365855 0.9779099

4271.91640978015 0.9906067

4271.93447590175 0.9768183

4271.95254202335 0.9518595

4271.97060814496 0.893671

4271.98867426656 0.906181

4272.00674038816 0.9703034

4272.02480650976 0.9639286

4272.04287263137 0.9264721

4272.06093875297 0.9375116

4272.07900487457 0.948056

4272.09707099617 0.9066714

4272.11513711777 0.9405571

4272.13320323938 0.9227672

4272.15126936098 0.9274359

4272.16933548258 0.9371488

4272.18740160418 0.9229378

4272.20546772579 0.8866438

4272.22353384739 0.922874

4272.24159996899 0.9994798

4272.25966609059 1.020286

4272.27773221219 0.9838759

4272.29579833379 0.9545699

4272.3138644554 0.9117651

4272.331930577 0.9561203

4272.3499966986 0.9780244

4272.3680628202 0.9532442

4272.38612894181 0.9406539

4272.40419506341 0.9957379

4272.42226118501 1.008001

4272.44032730661 0.9234944

4272.45839342822 0.911496

4272.47645954982 0.9585949

4272.49452567142 0.9440017

4272.51259179302 0.9270253

4272.53065791463 1.00387

4272.54872403623 0.9849644

4272.56679015783 0.9188849

4272.58485627943 0.8462333

4272.60292240103 0.8835887

4272.62098852264 0.9853728

4272.63905464424 0.9658229

4272.65712076584 0.9281464

4272.67518688744 0.8934159

4272.69325300904 0.8927678

4272.71131913065 0.9382334

4272.72938525225 0.9749801

4272.74745137385 0.9572265

4272.76551749545 0.9034966

4272.78358361706 0.9334782

4272.80164973866 0.92352

4272.81971586026 0.8793133

4272.83778198186 0.9393554

4272.85584810346 0.9525462

4272.87391422507 0.945578

4272.89198034667 0.9752097

4272.91004646827 0.9553487

4272.92811258987 0.9902591

4272.94617871148 1.01572

4272.96424483308 0.960924

4272.98231095468 0.8367288

4273.00037707628 0.8587784

4273.01844319788 0.8721367

4273.03650931949 0.8863878

4273.05457544109 0.9599562

4273.07264156269 0.9757051

4273.09070768429 0.895274

4273.10877380589 0.933579

4273.1268399275 0.9578822

4273.1449060491 0.9129836

4273.1629721707 0.8825877

4273.1810382923 0.9208592

4273.19910441391 0.9833348

4273.21717053551 0.9696456

4273.23523665711 0.9254563

4273.25330277871 0.8795109

4273.27136890031 0.9270471

4273.28943502192 0.9851901

4273.30750114352 0.9579625

4273.32556726512 0.9441205

4273.34363338672 0.9529495

4273.36169950833 0.9691548

4273.37976562993 0.9635934

4273.39783175153 0.9870834

4273.41589787313 1.008819

4273.43396399473 0.9183112

4273.45203011634 0.8837655

4273.47009623794 0.9752578

4273.48816235954 0.9149432

4273.50622848114 0.8212904

4273.52429460275 0.8429217

4273.54236072435 0.8396968

4273.56042684595 0.9054702

4273.57849296755 0.8513618

4273.59655908915 0.7926189

4273.61462521076 0.7581055

4273.63269133236 0.7892804

4273.65075745396 0.8861673

4273.66882357556 0.9043961

4273.68688969716 0.8801046

4273.70495581877 0.9424148

4273.72302194037 0.912563

4273.74108806197 0.9239698

4273.75915418357 0.8865495

4273.77722030518 0.8711382

4273.79528642678 1.002809

4273.81335254838 0.919414

4273.83141866998 0.9154349

4273.84948479159 0.8851382

4273.86755091319 0.9185419

4273.88561703479 0.9960233

4273.90368315639 0.8978983

4273.92174927799 0.8601793

4273.9398153996 0.929687

4273.9578815212 0.9327807

4273.9759476428 0.9138581

4273.9940137644 0.9340787

4274.012079886 0.9174679

4274.03014600761 0.9750497

4274.04821212921 1.020433

4274.06627825081 0.986388

4274.08434437241 0.9697071

4274.10241049402 0.9867368

4274.12047661562 0.9913591

4274.13854273722 0.9597893

4274.15660885882 0.9685636

4274.17467498042 0.972429

4274.19274110203 0.9445691

4274.21080722363 0.9456811

4274.22887334523 0.9586506

4274.24693946683 0.9749171

4274.26500558843 0.9891531

4274.28307171004 1.00803

4274.30113783164 1.02895

4274.31920395324 0.9955822

4274.33727007484 0.9712342

4274.35533619645 1.005276

4274.37340231805 1.010963

4274.39146843965 0.9829512

4274.40953456125 0.9520851

4274.42760068285 0.9590621

4274.44566680446 0.9789376

4274.46373292606 0.9523669

4274.48179904766 0.9534275

4274.49986516926 0.9903019

4274.51793129087 0.9978209

4274.53599741247 0.9518036

4274.55406353407 1.000901

4274.57212965567 1.063985

4274.59019577727 1.03914

4274.60826189888 0.9845873

4274.62632802048 1.0273

4274.64439414208 1.036983

4274.66246026368 0.9755423

4274.68052638529 0.9881437

4274.69859250689 0.9867558

4274.71665862849 0.9953139

4274.73472475009 1.036507

4274.7527908717 1.001711

4274.7708569933 0.9883156

4274.7889231149 0.9979337

4274.8069892365 0.9755133

4274.8250553581 0.9753519

4274.84312147971 0.9947274

4274.86118760131 0.9790468

4274.87925372291 0.9631099

4274.89731984451 0.9854462

4274.91538596611 0.9948841

4274.93345208772 1.003464

4274.95151820932 1.021071

4274.96958433092 1.011351

4274.98765045252 0.9959906

4275.00571657413 0.9645829

4275.02378269573 0.9738188

4275.04184881733 0.9998003

4275.05991493893 0.9671422

4275.07798106053 0.9782859

4275.09604718214 0.9935093

4275.11411330374 1.007098

4275.13217942534 0.9996159

4275.15024554694 0.9545447

4275.16831166855 0.9275323

4275.18637779015 0.9750764

4275.20444391175 1.005675

4275.22251003335 0.9648945

4275.24057615496 0.9397035

4275.25864227656 0.9654387

4275.27670839816 0.9933176

4275.29477451976 0.9695661

4275.31284064136 0.9444132

4275.33090676296 0.9903665

4275.34897288457 1.008934

4275.36703900617 0.9594787

4275.38510512777 0.9404879

4275.40317124937 0.9468828

4275.42123737098 0.9425882

4275.43930349258 0.95571

4275.45736961418 0.9654465

4275.47543573578 0.9447354

4275.49350185738 0.9388295

4275.51156797899 0.9805985

4275.52963410059 1.000962

4275.54770022219 0.9498091

4275.56576634379 0.917762

4275.5838324654 0.9476791

4275.601898587 0.9730814

4275.6199647086 0.9319277

4275.6380308302 0.9287914

4275.6560969518 0.9581395

4275.67416307341 0.9537517

4275.69222919501 0.950577

4275.71029531661 0.9607127

4275.72836143821 0.9869051

4275.74642755982 0.9640647

4275.76449368142 0.9324846

4275.78255980302 0.9278123

4275.80062592462 0.9213356

4275.81869204622 0.9306144

4275.83675816783 0.9611626

4275.85482428943 0.9470335

4275.87289041103 0.9300863

4275.89095653263 0.9522679

4275.90902265423 0.9388678

4275.92708877584 0.9435765

4275.94515489744 0.9634603

4275.96322101904 0.9363047

4275.98128714064 0.9221597

4275.99935326225 0.9931797

4276.01741938385 1.000765

4276.03548550545 0.9863123

4276.05355162705 0.9500973

4276.07161774865 0.9374986

4276.08968387026 0.9487965

4276.10774999186 0.9802434

4276.12581611346 0.9848974

4276.14388223506 0.9489253

4276.16194835667 1.000878

4276.18001447827 1.052013

4276.19808059987 1.00588

4276.21614672147 0.9650455

4276.23421284307 0.9385945

4276.25227896468 0.9502931

4276.27034508628 0.9841745

4276.28841120788 0.9682466

4276.30647732948 0.930741

4276.32454345109 0.9509043

4276.34260957269 0.9781759

4276.36067569429 0.995795

4276.37874181589 1.032452

4276.3968079375 1.026342

4276.4148740591 0.983555

4276.4329401807 0.987138

4276.4510063023 0.9741341

4276.4690724239 0.9311755

4276.4871385455 0.9136552

4276.50520466711 0.9851484

4276.52327078871 1.008182

4276.54133691031 1.001489

4276.55940303191 0.9735516

4276.57746915352 0.9772542

4276.59553527512 0.9705498

4276.61360139672 0.9607812

4276.63166751832 0.9389067

4276.64973363993 0.9037148

4276.66779976153 0.9141823

4276.68586588313 0.9435315

4276.70393200473 0.9595916

4276.72199812634 0.9623588

4276.74006424794 0.941818

4276.75813036954 0.9543828

4276.77619649114 0.9963682

4276.79426261274 0.9678631

4276.81232873435 0.9242353

4276.83039485595 0.9380549

4276.84846097755 0.9468306

4276.86652709915 0.9976933

4276.88459322075 0.9841996

4276.90265934236 0.9606495

4276.92072546396 0.9349319

4276.93879158556 0.9463109

4276.95685770716 0.8963072

4276.97492382876 0.9095113

4276.99298995037 0.9555463

4277.01105607197 0.9124317

4277.02912219357 0.9145026

4277.04718831517 0.9191478

4277.06525443678 1.003701

4277.08332055838 0.9945347

4277.10138667998 0.9149191

4277.11945280158 0.8943043

4277.13751892319 0.8923203

4277.15558504479 0.8832529

4277.17365116639 0.914791

4277.19171728799 0.9575222

4277.20978340959 0.9543345

4277.2278495312 0.9442026

4277.2459156528 0.9851928

4277.2639817744 0.9810091

4277.282047896 0.9185686

4277.3001140176 0.9143212

4277.31818013921 0.9897218

4277.33624626081 0.9950148

4277.35431238241 0.9287976

4277.37237850401 0.9395827

4277.39044462562 0.925574

4277.40851074722 0.9356524

4277.42657686882 0.9357254

4277.44464299042 0.9415956

4277.46270911202 0.9338587

4277.48077523363 0.923329

4277.49884135523 0.9446906

4277.51690747683 0.9497225

4277.53497359843 0.9271964

4277.55303972004 0.9101548

4277.57110584164 0.9065833

4277.58917196324 0.9200535

4277.60723808484 0.9146433

4277.62530420644 0.8849782

4277.64337032805 0.9018879

4277.66143644965 0.9398996

4277.67950257125 0.9147145

4277.69756869285 0.9095696

4277.71563481446 0.9404728

4277.73370093606 0.9058548

4277.75176705766 0.8738778

4277.76983317926 0.9103982

4277.78789930086 0.9254028

4277.80596542247 0.8815756

4277.82403154407 0.8997205

4277.84209766567 0.9436916

4277.86016378727 0.9220288

4277.87822990888 0.950651

4277.89629603048 0.951534

4277.91436215208 0.9168667

4277.93242827368 0.8855973

4277.95049439528 0.8730422

4277.96856051689 0.9182789

4277.98662663849 0.9057403

4278.00469276009 0.8579649

4278.02275888169 0.8504597

4278.0408250033 0.8204781

4278.0588911249 0.8007563

4278.0769572465 0.8569064

4278.0950233681 0.9145762

4278.1130894897 0.9195557

4278.13115561131 0.8658522

4278.14922173291 0.8679757

4278.16728785451 0.904511

4278.18535397611 0.9007815

4278.20342009771 0.8866924

4278.22148621932 0.9033992

4278.23955234092 0.9369798

4278.25761846252 0.9271044

4278.27568458412 0.8962447

4278.29375070573 0.9293809

4278.31181682733 0.9279176

4278.32988294893 0.8940645

4278.34794907053 0.9327475

4278.36601519213 0.9102964

4278.38408131374 0.8974116

4278.40214743534 0.9612674

4278.42021355694 0.9600222

4278.43827967854 0.8975334

4278.45634580014 0.9008709

4278.47441192175 0.9214461

4278.49247804335 0.9093026

4278.51054416495 0.9168459

4278.52861028655 0.9220816

4278.54667640816 0.9492313

4278.56474252976 0.9416456

4278.58280865136 0.9197357

4278.60087477296 0.8872514

4278.61894089456 0.9311631

4278.63700701617 0.9844155

4278.65507313777 1.025154

4278.67313925937 0.9992814

4278.69120538097 0.9182372

4278.70927150258 0.922107

4278.72733762418 0.9518318

4278.74540374578 0.9162074

4278.76346986738 0.9288615

4278.78153598899 0.9435865

4278.79960211059 0.922847

4278.81766823219 0.9536085

4278.83573435379 0.9725719

4278.85380047539 0.9404541

4278.871866597 0.9354548

4278.8899327186 0.9517249

4278.9079988402 0.9543666

4278.9260649618 0.9630319

4278.9441310834 0.978794

4278.96219720501 0.97146

4278.98026332661 0.922768

4278.99832944821 0.9181283

4279.01639556981 0.92839

4279.03446169142 0.9714212

4279.05252781302 0.9673742

4279.07059393462 0.9293011

4279.08866005622 0.9504466

4279.10672617782 0.9836439

4279.12479229943 0.9684374

4279.14285842103 0.9477317

4279.16092454263 0.9472897

4279.17899066423 0.9631769

4279.19705678583 0.991567

4279.21512290744 0.9638262

4279.23318902904 0.9348771

4279.25125515064 0.9594653

4279.26932127224 0.9907531

4279.28738739385 0.9742997

4279.30545351545 0.935384

4279.32351963705 0.9231678

4279.34158575865 0.9333071

4279.35965188026 0.9378626

4279.37771800186 0.9368678

4279.39578412346 0.9379835

4279.41385024506 0.934565

4279.43191636666 0.9476613

4279.44998248827 0.9404175

4279.46804860987 0.9568048

4279.48611473147 0.9945322

4279.50418085307 0.985655

4279.52224697467 0.9701177

4279.54031309628 0.9593534

4279.55837921788 0.9197087

4279.57644533948 0.9391269

4279.59451146108 0.9648968

4279.61257758269 0.9498751

4279.63064370429 0.936147

4279.64870982589 0.964704

4279.66677594749 0.9879718

4279.68484206909 0.9637176

4279.7029081907 0.989182

4279.7209743123 0.9706839

4279.7390404339 0.915002

4279.7571065555 0.9525059

4279.77517267711 0.9685541

4279.79323879871 0.9068936

4279.81130492031 0.9011644

4279.82937104191 0.938875

4279.84743716352 0.9473649

4279.86550328512 0.9658918

4279.88356940672 0.9565268

4279.90163552832 0.9337467

4279.91970164992 0.9179766

4279.93776777153 0.9342583

4279.95583389313 0.9580479

4279.97390001473 0.9627752

4279.99196613633 0.9518456

4280.01003225794 0.9589921

4280.02809837954 0.9623653

4280.04616450114 0.9810406

4280.06423062274 0.9444602

4280.08229674434 0.9488798

4280.10036286594 0.9956568

4280.11842898755 0.9683807

4280.13649510915 0.9571236

4280.15456123075 0.9376698

4280.17262735236 0.8907205

4280.19069347396 0.9363612

4280.20875959556 0.9518778

4280.22682571716 0.8811433

4280.24489183876 0.8736529

4280.26295796037 0.9263754

4280.28102408197 0.9205422

4280.29909020357 0.9225639

4280.31715632517 0.9411699

4280.33522244677 0.9299047

4280.35328856838 0.9094526

4280.37135468998 0.9218932

4280.38942081158 0.9474475

4280.40748693318 0.9540348

4280.42555305479 0.9459475

4280.44361917639 0.9176654

4280.46168529799 0.9050245

4280.47975141959 0.9055477

4280.49781754119 0.9846066

4280.5158836628 1.009645

4280.5339497844 1.001617

4280.552015906 0.9951493

4280.5700820276 0.9771295

4280.58814814921 0.9896919

4280.60621427081 0.973827

4280.62428039241 0.9412744

4280.64234651401 0.9377539

4280.66041263561 0.9085723

4280.67847875722 0.9231528

4280.69654487882 0.9733673

4280.71461100042 0.9315093

4280.73267712202 0.910116

4280.75074324362 0.9465461

4280.76880936523 0.9543269

4280.78687548683 0.946538

4280.80494160843 0.928705

4280.82300773003 0.9344325

4280.84107385164 0.9194633

4280.85913997324 0.8957457

4280.87720609484 0.9561926

4280.89527221644 0.9327074

4280.91333833804 0.9212415

4280.93140445965 0.9158427

4280.94947058125 0.9195446

4280.96753670285 0.9119272

4280.98560282445 0.9209418

4281.00366894605 0.9294937

4281.02173506766 0.9386534

4281.03980118926 0.9369781

4281.05786731086 0.9321746

4281.07593343246 0.9202713

4281.09399955407 0.9128144

4281.11206567567 0.9260758

4281.13013179727 0.9313764

4281.14819791887 0.9244139

4281.16626404047 0.9545203

4281.18433016208 0.9710721

4281.20239628368 0.926241

4281.22046240528 0.9328622

4281.23852852688 0.9503634

4281.25659464849 0.9217455

4281.27466077009 0.9472067

4281.29272689169 0.9645772

4281.31079301329 0.9272716

4281.3288591349 0.9286946

4281.3469252565 0.935304

4281.3649913781 0.9691758

4281.3830574997 0.9573563

4281.4011236213 0.9203924

4281.41918974291 0.9113359

4281.43725586451 0.9348993

4281.45532198611 0.985051

4281.47338810771 0.9956281

4281.49145422931 0.9813622

4281.50952035092 0.9079795

4281.52758647252 0.9021729

4281.54565259412 0.9530246

4281.56371871572 0.9228742

4281.58178483733 0.9057988

4281.59985095893 0.9192005

4281.61791708053 0.8950496

4281.63598320213 0.9078291

4281.65404932373 0.9389048

4281.67211544534 0.9417115

4281.69018156694 0.9668018

4281.70824768854 0.9625595

4281.72631381014 0.9278044

4281.74437993175 0.9436646

4281.76244605335 0.9652311

4281.78051217495 0.9518707

4281.79857829655 0.941833

4281.81664441815 0.9163673

4281.83471053976 0.898196

4281.85277666136 0.9248576

4281.87084278296 0.9309152

4281.88890890456 0.9219921

4281.90697502617 0.9294364

4281.92504114777 0.9269826

4281.94310726937 0.9486821

4281.96117339097 0.9491422

4281.97923951257 0.9326658

4281.99730563418 0.9235128

4282.01537175578 0.9248484

4282.03343787738 0.9155689

4282.05150399898 0.9059341

4282.06957012059 0.9068228

4282.08763624219 0.9249251

4282.10570236379 0.9564308

4282.12376848539 0.9275318

4282.14183460699 0.9336327

4282.1599007286 0.9458245

4282.1779668502 0.9376816

4282.1960329718 0.9514242

4282.2140990934 0.970875

4282.23216521501 0.9545764

4282.25023133661 0.9326223

4282.26829745821 0.9544023

4282.28636357981 0.9468173

4282.30442970141 0.9282501

4282.32249582302 0.9496589

4282.34056194462 0.9162263

4282.35862806622 0.8878429

4282.37669418782 0.93101

4282.39476030942 0.951974

4282.41282643103 0.9438375

4282.43089255263 0.9286063

4282.44895867423 0.9222841

4282.46702479583 0.9407944

4282.48509091744 0.9606249

4282.50315703904 0.9382423

4282.52122316064 0.9026443

4282.53928928224 0.9090264

4282.55735540385 0.9371456

4282.57542152545 0.9339037

4282.59348764705 0.9296018

4282.61155376865 0.9056785

4282.62961989025 0.891638

4282.64768601185 0.9011859

4282.66575213346 0.8992869

4282.68381825506 0.8730078

4282.70188437666 0.8854015

4282.71995049826 0.9334409

4282.73801661987 0.9565271

4282.75608274147 0.9309762

4282.77414886307 0.9137714

4282.79221498467 0.9301233

4282.81028110627 0.9561925

4282.82834722788 0.9597139

4282.84641334948 0.916126

4282.86447947108 0.9057595

4282.88254559268 0.9602394

4282.90061171429 0.9333243

4282.91867783589 0.8998843

4282.93674395749 0.8925108

4282.95481007909 0.9363551

4282.97287620069 0.9707448

4282.9909423223 0.9705304

4283.0090084439 0.977939

4283.0270745655 0.9643801

4283.0451406871 0.9510367

4283.06320680871 0.9607223

4283.08127293031 0.9668607

4283.09933905191 0.9929696

4283.11740517351 1.012962

4283.13547129511 1.009217

4283.15353741672 0.9955472

4283.17160353832 1.007714

4283.18966965992 1.002138

4283.20773578152 0.9583207

4283.22580190313 0.9546882

4283.24386802473 0.9693608

4283.26193414633 0.9904486

4283.28000026793 0.9723919

4283.29806638953 0.9460228

4283.31613251114 0.9600183

4283.33419863274 0.9566951

4283.35226475434 0.9520192

4283.37033087594 0.9675133

4283.38839699754 0.9759292

4283.40646311915 0.9712185

4283.42452924075 0.9652591

4283.44259536235 0.9542457

4283.46066148395 0.9721041

4283.47872760556 0.9871995

4283.49679372716 0.9627469

4283.51485984876 0.941469

4283.53292597036 0.9451058

4283.55099209197 0.9503086

4283.56905821357 0.9672507

4283.58712433517 0.9788701

4283.60519045677 0.9344699

4283.62325657837 0.9273397

4283.64132269998 0.9650466

4283.65938882158 0.9717436

4283.67745494318 0.9427419

4283.69552106478 0.9175572

4283.71358718638 0.9175545

4283.73165330799 0.9361818

4283.74971942959 0.9597291

4283.76778555119 0.9539876

4283.78585167279 0.9775543

4283.8039177944 1.009165

4283.821983916 0.9916604

4283.8400500376 0.9707081

4283.8581161592 0.9227502

4283.8761822808 0.9022081

4283.89424840241 0.940429

4283.91231452401 0.9453269

4283.93038064561 0.9401416

4283.94844676721 0.9852753

4283.96651288882 0.9863356

4283.98457901042 0.9516522

4284.00264513202 0.9385978

4284.02071125362 0.9291519

4284.03877737522 0.9161206

4284.05684349683 0.907533

4284.07490961843 0.9520338

4284.09297574003 0.9743129

4284.11104186163 0.9459592

4284.12910798324 0.9461062

4284.14717410484 0.9224373

4284.16524022644 0.9413761

4284.18330634804 0.9465067

4284.20137246964 0.8923733

4284.21943859125 0.9051085

4284.23750471285 0.9265457

4284.25557083445 0.9307211

4284.27363695605 0.9650891

4284.29170307765 0.9814899

4284.30976919926 0.9617829

4284.32783532086 0.9166977

4284.34590144246 0.9057496

4284.36396756406 0.897657

4284.38203368567 0.8858032

4284.40009980727 0.9005942

4284.41816592887 0.8869629

4284.43623205047 0.8860029

4284.45429817207 0.8819731

4284.47236429368 0.8721573

4284.49043041528 0.8781879

4284.50849653688 0.8793579

4284.52656265848 0.8543079

4284.54462878009 0.8383193

4284.56269490169 0.8473617

4284.58076102329 0.845714

4284.59882714489 0.879854

4284.6168932665 0.9072164

4284.6349593881 0.8746818

4284.6530255097 0.8597616

4284.6710916313 0.8433986

4284.6891577529 0.8543755

4284.70722387451 0.8896443

4284.72528999611 0.9200729

4284.74335611771 0.8983853

4284.76142223931 0.9014072

4284.77948836092 0.8998706

4284.79755448252 0.87034

4284.81562060412 0.888465

4284.83368672572 0.9098616

4284.85175284732 0.9086052

4284.86981896893 0.9347565

4284.88788509053 0.9364016

4284.90595121213 0.908864

4284.92401733373 0.929521

4284.94208345534 0.9576306

4284.96014957694 0.9542059

4284.97821569854 0.9464585

4284.99628182014 0.9748362

4285.01434794174 0.9841771

4285.03241406335 0.9785236

4285.05048018495 0.98181

4285.06854630655 0.9629773

4285.08661242815 0.962151

4285.10467854975 0.9719176

4285.12274467136 0.9950175

4285.14081079296 1.028433

4285.15887691456 1.015757

4285.17694303616 0.9948382

4285.19500915777 1.006003

4285.21307527937 1.013577

4285.23114140097 1.013888

4285.24920752257 0.9733642

4285.26727364417 0.9453977

4285.28533976578 0.9551439

4285.30340588738 0.9894886

4285.32147200898 1.00433

4285.33953813058 0.9952856

4285.35760425218 0.986538

4285.37567037379 0.970295

4285.39373649539 0.9636474

4285.41180261699 0.9587808

4285.42986873859 0.9779646

4285.4479348602 0.9920895

4285.4660009818 0.9724346

4285.4840671034 0.990105

4285.502133225 0.9933998

4285.52019934661 0.9791658

4285.53826546821 0.9847623

4285.55633158981 0.9943448

4285.57439771141 0.9721981

4285.59246383301 0.9753022

4285.61052995462 0.9975777

4285.62859607622 0.9707369

4285.64666219782 0.9511257

4285.66472831942 0.9355705

4285.68279444102 0.9304128

4285.70086056263 0.9512818

4285.71892668423 0.9353766

4285.73699280583 0.935869

4285.75505892743 0.9553834

4285.77312504904 0.9421479

4285.79119117064 0.9536346

4285.80925729224 0.9857427

4285.82732341384 0.9451317

4285.84538953544 0.9128784

4285.86345565705 0.9444129

4285.88152177865 0.9540638

4285.89958790025 0.9640208

4285.91765402185 0.9661983

4285.93572014345 0.9492691

4285.95378626506 0.936258

4285.97185238666 0.9546698

4285.98991850826 0.9576415

4286.00798462986 0.9653986

4286.02605075147 0.9292918

4286.04411687307 0.9341988

4286.06218299467 0.9651061

4286.08024911627 0.9872028

4286.09831523788 0.99359

4286.11638135948 0.9630262

4286.13444748108 0.9413391

4286.15251360268 0.9576187

4286.17057972428 0.9561682

4286.18864584589 0.9507256

4286.20671196749 0.9494615

4286.22477808909 0.936192

4286.24284421069 0.9293204

4286.2609103323 0.9374688

4286.2789764539 0.9500051

4286.2970425755 0.9394332

4286.3151086971 0.942585

4286.3331748187 0.9620626

4286.35124094031 0.9490579

4286.36930706191 0.9606428

4286.38737318351 0.948787

4286.40543930511 0.929009

4286.42350542672 0.9142421

4286.44157154832 0.9022154

4286.45963766992 0.9217484

4286.47770379152 0.9046921

4286.49576991312 0.9060616

4286.51383603473 0.8864657

4286.53190215633 0.90825

4286.54996827793 0.9450405

4286.56803439953 0.9283327

4286.58610052113 0.9185752

4286.60416664274 0.9554377

4286.62223276434 0.9807577

4286.64029888594 0.9545201

4286.65836500754 0.9500591

4286.67643112915 0.9622668

4286.69449725075 0.9547299

4286.71256337235 0.9324257

4286.73062949395 0.9645816

4286.74869561556 0.9619654

4286.76676173716 0.9586375

4286.78482785876 0.9874227

4286.80289398036 0.9798477

4286.82096010196 0.987613

4286.83902622356 1.002096

4286.85709234517 0.9981676

4286.87515846677 0.998578

4286.89322458837 0.9789208

4286.91129070997 0.9807693

4286.92935683158 1.025641

4286.94742295318 1.037943

4286.96548907478 1.028345

4286.98355519638 0.9953907

4287.00162131799 0.9718363

4287.01968743959 0.9712037

4287.03775356119 0.9756579

4287.05581968279 0.9714187

4287.07388580439 0.9938171

4287.091951926 1.013577

4287.1100180476 0.9919227

4287.1280841692 0.9894492

4287.1461502908 1.030872

4287.16421641241 1.047207

4287.18228253401 1.004192

4287.20034865561 0.9920665

4287.21841477721 1.009608

4287.23648089881 0.9785779

4287.25454702042 0.9706496

4287.27261314202 1.004104

4287.29067926362 1.003929

4287.30874538522 0.9798201

4287.32681150682 0.9903885

4287.34487762843 1.00097

4287.36294375003 1.012563

4287.38100987163 1.009407

4287.39907599323 0.9698321

4287.41714211484 0.9549177

4287.43520823644 0.9995669

4287.45327435804 1.04227

4287.47134047964 1.021398

4287.48940660124 0.9803104

4287.50747272285 1.011428

4287.52553884445 1.024646

4287.54360496605 0.9718702

4287.56167108765 0.9839896

4287.57973720926 1.004841

4287.59780333086 1.031023

4287.61586945246 1.019723

4287.63393557406 0.9964679

4287.65200169566 0.9971291

4287.67006781727 1.007039

4287.68813393887 1.010153

4287.70620006047 0.9654979

4287.72426618207 0.9417045

4287.74233230368 0.9762306

4287.76039842528 0.9738374

4287.77846454688 0.955188

4287.79653066848 0.9440861

4287.81459679008 0.9599259

4287.83266291169 0.9510399

4287.85072903329 0.9574187

4287.86879515489 0.9505763

4287.88686127649 0.9072593

4287.90492739809 0.9112011

4287.9229935197 0.9305238

4287.9410596413 0.9317721

4287.9591257629 0.9185848

4287.9771918845 0.8969669

4287.99525800611 0.8684342

4288.01332412771 0.8967943

4288.03139024931 0.9204195

4288.04945637091 0.9021922

4288.06752249251 0.8803784

4288.08558861412 0.8769098

4288.10365473572 0.8801661

4288.12172085732 0.8735247

4288.13978697892 0.8632408

4288.15785310053 0.8597625

4288.17591922213 0.8995875

4288.19398534373 0.8920698

4288.21205146533 0.8561541

4288.23011758693 0.8738543

4288.24818370854 0.8860862

4288.26624983014 0.8979801

4288.28431595174 0.8724831

4288.30238207334 0.8834053

4288.32044819495 0.8954852

4288.33851431655 0.8835895

4288.35658043815 0.8704276

4288.37464655975 0.9092869

4288.39271268135 0.9242437

4288.41077880296 0.8986268

4288.42884492456 0.8884337

4288.44691104616 0.9054127

4288.46497716776 0.9204202

4288.48304328936 0.9136645

4288.50110941097 0.9072472

4288.51917553257 0.9207512

4288.53724165417 0.940261

4288.55530777577 0.9123988

4288.57337389738 0.9061353

4288.59144001898 0.9106251

4288.60950614058 0.927969

4288.62757226218 0.9208942

4288.64563838379 0.8997462

4288.66370450539 0.9120259

4288.68177062699 0.9205056

4288.69983674859 0.9118137

4288.71790287019 0.9179247

4288.7359689918 0.945516

4288.7540351134 0.9481475

4288.772101235 0.9193914

4288.7901673566 0.9553288

4288.80823347821 0.9738318

4288.82629959981 0.9736369

4288.84436572141 1.006415

4288.86243184301 0.99994

4288.88049796461 0.9706722

4288.89856408622 0.9587245

4288.91663020782 0.9654005

4288.93469632942 0.9788946

4288.95276245102 0.9971286

4288.97082857263 0.9892975

4288.98889469423 0.9459461

4289.00696081583 0.938012

4289.02502693743 0.9697176

4289.04309305903 0.9765625

4289.06115918063 0.9728295

4289.07922530224 0.9767373

4289.09729142384 0.9597489

4289.11535754544 0.9957076

4289.13342366704 0.9808507

4289.15148978865 0.9667025

4289.16955591025 0.9908167

4289.18762203185 0.9967333

4289.20568815345 0.9897944

4289.22375427505 0.9906664

4289.24182039666 0.9816698

4289.25988651826 0.9677413

4289.27795263986 0.9783587

4289.29601876146 0.9848919

4289.31408488307 0.9671202

4289.33215100467 0.9631408

4289.35021712627 0.9715813

4289.36828324787 0.9891236

4289.38634936948 0.9834639

4289.40441549108 0.9516546

4289.42248161268 0.945205

4289.44054773428 0.9496863

4289.45861385588 0.9750719

4289.47667997749 0.963174

4289.49474609909 0.9384657

4289.51281222069 0.9637424

4289.53087834229 0.9905207

4289.5489444639 1.00668

4289.5670105855 0.991591

4289.5850767071 0.9706131

4289.6031428287 0.9824735

4289.6212089503 0.9815212

4289.63927507191 0.9544159

4289.65734119351 0.9324025

4289.67540731511 0.9369886

4289.69347343671 0.9770294

4289.71153955832 0.9845071

4289.72960567992 0.9794068

4289.74767180152 0.9739877

4289.76573792312 0.9837508

4289.78380404472 0.9896892

4289.80187016633 0.9635901

4289.81993628793 0.9479787

4289.83800240953 0.936462

4289.85606853113 0.9333243

4289.87413465273 0.9136504

4289.89220077434 0.94522

4289.91026689594 0.917291

4289.92833301754 0.9114487

4289.94639913914 0.9113711

4289.96446526075 0.8635795

4289.98253138235 0.8524448

4290.00059750395 0.8624869

4290.01866362555 0.8742163

4290.03672974715 0.8411256

4290.05479586876 0.8024684

4290.07286199036 0.7982051

4290.09092811196 0.8156272

4290.10899423356 0.8053256

4290.12706035517 0.7657361

4290.14512647677 0.7587726

4290.16319259837 0.7687657

4290.18125871997 0.7621329

4290.19932484157 0.7477698

4290.21739096318 0.7425491

4290.23545708478 0.7547954

4290.25352320638 0.7397233

4290.27158932798 0.7093168

4290.28965544959 0.7371353

4290.30772157119 0.764827

4290.32578769279 0.7339409

4290.34385381439 0.7244489

4290.36191993599 0.7322495

4290.3799860576 0.7109141

4290.3980521792 0.7295372

4290.4161183008 0.7679054

4290.4341844224 0.7595416

4290.45225054401 0.727062

4290.47031666561 0.7410679

4290.48838278721 0.7887561

4290.50644890881 0.7675289

4290.52451503041 0.7705349

4290.54258115202 0.8060985

4290.56064727362 0.8003169

4290.57871339522 0.8301022

4290.59677951682 0.8439717

4290.61484563843 0.7948291

4290.63291176003 0.7659833

4290.65097788163 0.8073858

4290.66904400323 0.844254

4290.68711012483 0.8479546

4290.70517624644 0.8748891

4290.72324236804 0.8835789

4290.74130848964 0.8734536

4290.75937461124 0.8772584

4290.77744073284 0.9023054

4290.79550685445 0.9420952

4290.81357297605 0.9591491

4290.83163909765 0.9296041

4290.84970521925 0.8939297

4290.86777134086 0.9050071

4290.88583746246 0.9354621

4290.90390358406 0.9311948

4290.92196970566 0.9149834

4290.94003582726 0.9315919

4290.95810194887 0.9226564

4290.97616807047 0.8816934

4290.99423419207 0.8955498

4291.01230031367 0.9366407

4291.03036643527 0.9210258

4291.04843255688 0.9147463

4291.06649867848 0.901062

4291.08456480008 0.9086882

4291.10263092168 0.9080638

4291.12069704329 0.9350926

4291.13876316489 0.9142345

4291.15682928649 0.9222038

4291.17489540809 0.9221977

4291.19296152969 0.9079844

4291.2110276513 0.8935516

4291.2290937729 0.8916497

4291.2471598945 0.921762

4291.2652260161 0.9290426

4291.28329213771 0.9364732

4291.30135825931 0.92495

4291.31942438091 0.8976828

4291.33749050251 0.9117704

4291.35555662412 0.951902

4291.37362274572 0.9502954

4291.39168886732 0.9255378

4291.40975498892 0.9630381

4291.42782111052 0.9845605

4291.44588723213 0.9732562

4291.46395335373 0.99606

4291.48201947533 0.978458

4291.50008559693 0.9294884

4291.51815171853 0.9306817

4291.53621784014 0.9155689

4291.55428396174 0.9119982

4291.57235008334 0.932189

4291.59041620494 0.958536

4291.60848232655 0.9397601

4291.62654844815 0.9042329

4291.64461456975 0.9302988

4291.66268069135 0.9641367

4291.68074681295 0.951244

4291.69881293456 0.9473115

4291.71687905616 0.9154946

4291.73494517776 0.9308199

4291.75301129936 0.9366579

4291.77107742097 0.9272566

4291.78914354257 0.927909

4291.80720966417 0.910938

4291.82527578577 0.9361871

4291.84334190737 0.9790629

4291.86140802898 0.9741243

4291.87947415058 0.966952

4291.89754027218 0.930104

4291.91560639378 0.9097496

4291.93367251539 0.8928907

4291.95173863699 0.8956047

4291.96980475859 0.9378991

4291.98787088019 0.924723

4292.00593700179 0.9162469

4292.0240031234 0.9151944

4292.042069245 0.8920182

4292.0601353666 0.8769081

4292.0782014882 0.9103872

4292.0962676098 0.9218476

4292.11433373141 0.9347569

4292.13239985301 0.9270024

4292.15046597461 0.8948503

4292.16853209621 0.9080896

4292.18659821782 0.9308015

4292.20466433942 0.8932633

4292.22273046102 0.880253

4292.24079658262 0.8981079

4292.25886270422 0.9107538

4292.27692882583 0.8999546

4292.29499494743 0.896858

4292.31306106903 0.928316

4292.33112719063 0.9562242

4292.34919331224 0.9722437

4292.36725943384 0.9287096

4292.38532555544 0.9313328

4292.40339167704 0.9658247

4292.42145779864 0.9307616

4292.43952392025 0.9006263

4292.45759004185 0.9245807

4292.47565616345 0.9438242

4292.49372228505 0.9390424

4292.51178840666 0.9175729

4292.52985452826 0.8958158

4292.54792064986 0.8925391

4292.56598677146 0.9169912

4292.58405289306 0.9413723

4292.60211901467 0.9288528

4292.62018513627 0.9259232

4292.63825125787 0.9176181

4292.65631737947 0.913648

4292.67438350107 0.9184491

4292.69244962268 0.9559128

4292.71051574428 0.9976358

4292.72858186588 0.9734954

4292.74664798748 0.9522929

4292.76471410909 0.9456623

4292.78278023069 0.9568496

4292.80084635229 0.9663214

4292.81891247389 0.9605666

4292.83697859549 0.9412489

4292.8550447171 0.9216318

4292.8731108387 0.9297314

4292.8911769603 0.9445576

4292.9092430819 0.9258724

4292.92730920351 0.9216489

4292.94537532511 0.9210084

4292.96344144671 0.9193726

4292.98150756831 0.9190497

4292.99957368991 0.9233332

4293.01763981152 0.9549617

4293.03570593312 0.9811385

4293.05377205472 0.9441828

4293.07183817632 0.9035214

4293.08990429793 0.9019355

4293.10797041953 0.9279485

4293.12603654113 0.9447984

4293.14410266273 0.9253687

4293.16216878433 0.9392284

4293.18023490594 0.9502701

4293.19830102754 0.9434439

4293.21636714914 0.9497057

4293.23443327074 0.9476286

4293.25249939234 0.931379

4293.27056551395 0.9461771

4293.28863163555 0.9605835

4293.30669775715 0.9662384

4293.32476387875 0.9646903

4293.34283000036 0.9282432

4293.36089612196 0.92057

4293.37896224356 0.9336494

4293.39702836516 0.93339

4293.41509448677 0.9404501

4293.43316060837 0.9669078

4293.45122672997 0.9411439

4293.46929285157 0.9336659

4293.48735897317 0.9596029

4293.50542509478 0.9903812

4293.52349121638 0.9787697

4293.54155733798 0.9733036

4293.55962345958 0.9609691

4293.57768958119 0.9491059

4293.59575570279 0.9625087

4293.61382182439 0.9882248

4293.63188794599 1.008425

4293.64995406759 1.016647

4293.6680201892 1.01044

4293.6860863108 1.001549

4293.7041524324 0.9850619

4293.722218554 0.9586043

4293.74028467561 0.9526496

4293.75835079721 0.95789

4293.77641691881 0.9798189

4293.79448304041 1.004995

4293.81254916201 1.012919

4293.83061528362 0.9889885

4293.84868140522 1.006442

4293.86674752682 1.011767

4293.88481364842 0.9666501

4293.90287977003 0.9650297

4293.92094589163 0.9552702

4293.93901201323 0.9116753

4293.95707813483 0.9100746

4293.97514425643 0.9464152

4293.99321037804 0.9175267

4294.01127649964 0.9028525

4294.02934262124 0.8910434

4294.04740874284 0.8924158

4294.06547486444 0.9088731

4294.08354098605 0.9232121

4294.10160710765 0.9207062

4294.11967322925 0.9288445

4294.13773935085 0.898543

4294.15580547246 0.8789937

4294.17387159406 0.8952434

4294.19193771566 0.8968009

4294.21000383726 0.872618

4294.22806995886 0.8621004

4294.24613608047 0.8711848

4294.26420220207 0.876954

4294.28226832367 0.8909787

4294.30033444527 0.8883003

4294.31840056688 0.869705

4294.33646668848 0.8700881

4294.35453281008 0.873863

4294.37259893168 0.8862813

4294.39066505328 0.8587779

4294.40873117489 0.8715897

4294.42679729649 0.859751

4294.44486341809 0.8450484

4294.46292953969 0.8990932

4294.4809956613 0.9071159

4294.4990617829 0.8606522

4294.5171279045 0.8693625

4294.5351940261 0.8867426

4294.5532601477 0.9268869

4294.57132626931 0.9493979

4294.58939239091 0.9208601

4294.60745851251 0.904356

4294.62552463411 0.9094503

4294.64359075571 0.9076554

4294.66165687732 0.9129984

4294.67972299892 0.9386634

4294.69778912052 0.9804878

4294.71585524212 0.9675803

4294.73392136373 0.9446205

4294.75198748533 0.9654408

4294.77005360693 0.9720418

4294.78811972853 0.9794707

4294.80618585013 0.9875011

4294.82425197174 0.9652836

4294.84231809334 0.9443523

4294.86038421494 0.9416596

4294.87845033654 0.9727839

4294.89651645815 0.9593623

4294.91458257975 0.9184314

4294.93264870135 0.9539064

4294.95071482295 0.9758481

4294.96878094455 0.9730247

4294.98684706616 0.9583236

4295.00491318776 0.9583937

4295.02297930936 0.9661127

4295.04104543096 0.9895551

4295.05911155257 0.9603382

4295.07717767417 0.980784

4295.09524379577 1.010566

4295.11330991737 0.9400868

4295.13137603897 0.9472359

4295.14944216058 0.996442

4295.16750828218 0.98899

4295.18557440378 0.9501945

4295.20364052538 0.9352578

4295.22170664698 0.9761234

4295.23977276859 0.988753

4295.25783889019 0.9824781

4295.27590501179 0.940338

4295.29397113339 0.9628377

4295.312037255 1.004449

4295.3301033766 0.9767198

4295.3481694982 0.9422992

4295.3662356198 0.9563624

4295.3843017414 0.9717811

4295.40236786301 0.9658669

4295.42043398461 0.9648643

4295.43850010621 0.9528246

4295.45656622781 0.9887475

4295.47463234942 1.001593

4295.49269847102 0.9775139

4295.51076459262 0.9857519

4295.52883071422 0.9756103

4295.54689683583 0.9790019

4295.56496295743 0.9811898

4295.58302907903 0.9683522

4295.60109520063 0.9796423

4295.61916132223 0.9860126

4295.63722744384 0.9698715

4295.65529356544 0.9588336

4295.67335968704 0.9479566

4295.69142580864 0.9282557

4295.70949193024 0.9587847

4295.72755805185 0.980404

4295.74562417345 0.9559106

4295.76369029505 0.945322

4295.78175641665 0.9734235

4295.79982253826 0.9939996

4295.81788865986 0.9896456

4295.83595478146 0.9994923

4295.85402090306 0.9787363

4295.87208702466 0.9718659

4295.89015314627 1.009752

4295.90821926787 1.002653

4295.92628538947 0.9658866

4295.94435151107 0.982915

4295.96241763268 0.9703659

4295.98048375428 0.937604

4295.99854987588 0.941165

4296.01661599748 0.9657569

4296.03468211908 0.971631

4296.05274824069 0.9534423

4296.07081436229 0.9423866

4296.08888048389 0.9191785

4296.10694660549 0.8981261

4296.1250127271 0.9071488

4296.1430788487 0.9264446

4296.1611449703 0.9534582

4296.1792110919 0.961334

4296.1972772135 0.9355332

4296.2153433351 0.9248497

4296.23340945671 0.9298785

4296.25147557831 0.9477503

4296.26954169991 0.9346907

4296.28760782151 0.9013249

4296.30567394312 0.9375038

4296.32374006472 0.9352645

4296.34180618632 0.9301063

4296.35987230792 0.9367458

4296.37793842953 0.9124135

4296.39600455113 0.9218167

4296.41407067273 0.9139922

4296.43213679433 0.8645965

4296.45020291593 0.8119878

4296.46826903754 0.8522491

4296.48633515914 0.8823545

4296.50440128074 0.8506362

4296.52246740234 0.8495367

4296.54053352395 0.8691557

4296.55859964555 0.8687454

4296.57666576715 0.9006203

4296.59473188875 0.8703533

4296.61279801035 0.85074

4296.63086413196 0.8345586

4296.64893025356 0.8150131

4296.66699637516 0.8093967

4296.68506249676 0.800073

4296.70312861837 0.8254449

4296.72119473997 0.793314

4296.73926086157 0.7774932

4296.75732698317 0.7848011

4296.77539310477 0.7827678

4296.79345922638 0.8051547

4296.81152534798 0.7922178

4296.82959146958 0.8089651

4296.84765759118 0.8426248

4296.86572371279 0.8258513

4296.88378983439 0.8194169

4296.90185595599 0.8440118

4296.91992207759 0.8463569

4296.93798819919 0.8217604

4296.9560543208 0.8340608

4296.9741204424 0.8365619

4296.992186564 0.8244125

4297.0102526856 0.8125452

4297.0283188072 0.8211299

4297.04638492881 0.8132061

4297.06445105041 0.8166221

4297.08251717201 0.8700379

4297.10058329361 0.8895331

4297.11864941522 0.8866596

4297.13671553682 0.8811677

4297.15478165842 0.856987

4297.17284778002 0.8902615

4297.19091390162 0.9480932

4297.20898002323 0.9456345

4297.22704614483 0.9272643

4297.24511226643 0.9409032

4297.26317838803 0.9635311

4297.28124450963 0.946021

4297.29931063124 0.9157523

4297.31737675284 0.9179379

4297.33544287444 0.9463485

4297.35350899604 0.9339228

4297.37157511765 0.942947

4297.38964123925 0.9659255

4297.40770736085 0.9653105

4297.42577348245 0.9283741

4297.44383960405 0.9121323

4297.46190572566 0.9279761

4297.47997184726 0.9680865

4297.49803796886 0.9571676

4297.51610409046 0.9233987

4297.53417021207 0.9122842

4297.55223633367 0.9495817

4297.57030245527 0.9585905

4297.58836857687 0.9598948

4297.60643469847 0.9560883

4297.62450082008 0.969348

4297.64256694168 1.000696

4297.66063306328 0.9826113

4297.67869918488 0.9620301

4297.69676530649 1.003285

4297.71483142809 0.9750124

4297.73289754969 0.9552733

4297.75096367129 0.991331

4297.7690297929 1.001607

4297.7870959145 0.9565289

4297.8051620361 0.9386498

4297.8232281577 0.9446803

4297.8412942793 0.9327796

4297.8593604009 0.9516826

4297.87742652251 0.9893497

4297.89549264411 0.9772079

4297.91355876571 0.9380528

4297.93162488731 0.9253372

4297.94969100892 0.9357386

4297.96775713052 0.9553639

4297.98582325212 0.9735101

4298.00388937372 0.9883991

4298.02195549532 0.9882748

4298.04002161693 0.9385055

4298.05808773853 0.9327972

4298.07615386013 0.9612494

4298.09421998173 0.9771385

4298.11228610334 0.9574894

4298.13035222494 0.9614329

4298.14841834654 0.9693593

4298.16648446814 0.9752533

4298.18455058975 1.004079

4298.20261671135 0.9852127

4298.22068283295 0.9603907

4298.23874895455 0.960398

4298.25681507615 0.9635385

4298.27488119776 0.9834685

4298.29294731936 0.9400647

4298.31101344096 0.9317983

4298.32907956256 0.9839014

4298.34714568417 0.9813129

4298.36521180577 0.9789519

4298.38327792737 0.9899346

4298.40134404897 0.9503596

4298.41941017057 0.9401619

4298.43747629218 0.9539206

4298.45554241378 0.941161

4298.47360853538 0.9426647

4298.49167465698 0.9259548

4298.50974077859 0.9088674

4298.52780690019 0.9147117

4298.54587302179 0.9281184

4298.56393914339 0.9222962

4298.58200526499 0.9112619

4298.6000713866 0.920768

4298.6181375082 0.9577951

4298.6362036298 0.936022

4298.6542697514 0.9278109

4298.67233587301 0.9562254

4298.69040199461 0.9547254

4298.70846811621 0.9192262

4298.72653423781 0.9035573

4298.74460035942 0.8990772

4298.76266648102 0.9296356

4298.78073260262 0.9613823

4298.79879872422 0.9722721

4298.81686484582 0.9603908

4298.83493096743 0.9435445

4298.85299708903 0.9329545

4298.87106321063 0.9373515

4298.88912933223 0.9791958

4298.90719545383 0.9856688

4298.92526157544 0.9876133

4298.94332769704 0.9777129

4298.96139381864 0.9617923

4298.97945994024 0.9774508

4298.99752606185 0.9592786

4299.01559218345 0.9652369

4299.03365830505 0.9928997

4299.05172442665 0.9892355

4299.06979054825 0.9614047

4299.08785666986 0.9473022

4299.10592279146 0.9400709

4299.12398891306 0.9703593

4299.14205503466 0.9471021

4299.16012115626 0.9579431

4299.17818727787 0.9401182

4299.19625339947 0.9559076

4299.21431952107 0.9473754

4299.23238564267 0.9278927

4299.25045176428 0.9174722

4299.26851788588 0.9339823

4299.28658400748 0.9669031

4299.30465012908 0.9868342

4299.32271625068 0.9615016

4299.34078237229 0.9426033

4299.35884849389 0.9671112

4299.37691461549 0.9564798

4299.39498073709 0.9544809

4299.4130468587 0.9632962

4299.4311129803 0.9100913

4299.4491791019 0.9054807

4299.4672452235 0.9291362

4299.4853113451 0.9291147

4299.50337746671 0.9606959

4299.52144358831 0.9582603

4299.53950970991 0.9548973

4299.55757583151 0.9604424

4299.57564195311 0.9361181

4299.59370807472 0.9407347

4299.61177419632 0.9442531

4299.62984031792 1.002869

4299.64790643952 1.035674

4299.66597256113 1.022989

4299.68403868273 0.9947252

4299.70210480433 0.9660439

4299.72017092593 0.9432787

4299.73823704753 0.9887069

4299.75630316914 0.986172

4299.77436929074 0.9759184

4299.79243541234 0.9390053

4299.81050153394 0.9388369

4299.82856765555 0.9650412

4299.84663377715 0.9889085

4299.86469989875 0.9530063

4299.88276602035 0.923722

4299.90083214195 0.9269984

4299.91889826356 0.9256324

4299.93696438516 0.8991458

4299.95503050676 0.881507

4299.97309662836 0.9319155

4299.99116274997 0.9278476

4300.00922887157 0.9212093

4300.02729499317 0.9208027

4300.04536111477 0.8860039

4300.06342723637 0.8828351

4300.08149335798 0.8617504

4300.09955947958 0.8251712

4300.11762560118 0.8296971

4300.13569172278 0.8347235

4300.15375784439 0.852129

4300.17182396599 0.8372561

4300.18989008759 0.7967172

4300.20795620919 0.7865317

4300.22602233079 0.7969178

4300.2440884524 0.7987295

4300.262154574 0.7950346

4300.2802206956 0.7983695

4300.2982868172 0.7769653

4300.31635293881 0.8049002

4300.33441906041 0.8312185

4300.35248518201 0.8826234

4300.37055130361 0.8749257

4300.38861742521 0.8488275

4300.40668354681 0.8528033

4300.42474966842 0.8617714

4300.44281579002 0.8616734

4300.46088191162 0.8703459

4300.47894803322 0.8820341

4300.49701415483 0.8482405

4300.51508027643 0.852183

4300.53314639803 0.910357

4300.55121251963 0.8963428

4300.56927864124 0.8854192

4300.58734476284 0.9095487

4300.60541088444 0.9382985

4300.62347700604 0.9379169

4300.64154312764 0.9342021

4300.65960924925 0.9485826

4300.67767537085 0.9216385

4300.69574149245 0.9250411

4300.71380761405 0.9414756

4300.73187373566 0.9716915

4300.74993985726 0.980614

4300.76800597886 0.9373259

4300.78607210046 0.8984525

4300.80413822206 0.9254514

4300.82220434367 0.9675767

4300.84027046527 0.9974597

4300.85833658687 1.038353

4300.87640270847 1.028036

4300.89446883008 0.9976789

4300.91253495168 0.9988205

4300.93060107328 1.007449

4300.94866719488 0.9813241

4300.96673331648 1.010342

4300.98479943809 1.067663

4301.00286555969 0.9810781

4301.02093168129 0.9176797

4301.03899780289 0.9583541

4301.05706392449 0.9646543

4301.0751300461 0.971247

4301.0931961677 0.9744505

4301.1112622893 1.007141

4301.1293284109 1.013433

4301.14739453251 0.983348

4301.16546065411 1.001095

4301.18352677571 1.050152

4301.20159289731 1.032482

4301.21965901891 0.9736069

4301.23772514052 0.9874002

4301.25579126212 1.013474

4301.27385738372 1.009601

4301.29192350532 1.016661

4301.30998962693 1.017642

4301.32805574853 1.014188

4301.34612187013 1.013568

4301.36418799173 0.9932565

4301.38225411334 0.9581351

4301.40032023494 0.9423176

4301.41838635654 0.9618703

4301.43645247814 0.9527757

4301.45451859974 0.9271317

4301.47258472134 0.9823948

4301.49065084295 0.9705623

4301.50871696455 0.9470637

4301.52678308615 0.9686186

4301.54484920775 0.9892085

4301.56291532936 1.039017

4301.58098145096 1.019424

4301.59904757256 0.9645131

4301.61711369416 0.9784011

4301.63517981577 0.9763632

4301.65324593737 0.9592862

4301.67131205897 0.9648871

4301.68937818057 0.9811464

4301.70744430217 1.001435

4301.72551042378 0.9903495

4301.74357654538 0.9376661

4301.76164266698 0.8783327

4301.77970878858 0.8912118

4301.79777491018 0.9554546

4301.81584103179 0.9707752

4301.83390715339 0.950829

4301.85197327499 0.9637104

4301.87003939659 0.9684339

4301.8881055182 0.9452341

4301.9061716398 0.9174838

4301.9242377614 0.9247226

4301.942303883 0.9508961

4301.96037000461 0.9278966

4301.97843612621 0.8969349

4301.99650224781 0.9111505

4302.01456836941 0.9131014

4302.03263449101 0.9235076

4302.05070061262 0.8886293

4302.06876673422 0.8440155

4302.08683285582 0.856611

4302.10489897742 0.9027011

4302.12296509902 0.9139607

4302.14103122063 0.9039575

4302.15909734223 0.8911231

4302.17716346383 0.8731788

4302.19522958543 0.9044358

4302.21329570704 0.9158731

4302.23136182864 0.917639

4302.24942795024 0.9789174

4302.26749407184 0.9906604

4302.28556019344 0.9707571

4302.30362631505 0.9922571

4302.32169243665 0.9759389

4302.33975855825 0.9580905

4302.35782467985 0.9663193

4302.37589080146 1.009184

4302.39395692306 0.9999843

4302.41202304466 0.9605837

4302.43008916626 0.9737238

4302.44815528786 0.9899832

4302.46622140947 1.037791

4302.48428753107 1.029211

4302.50235365267 1.041256

4302.52041977427 1.023211

4302.53848589588 1.007359

4302.55655201748 1.01878

4302.57461813908 1.005199

4302.59268426068 1.059091

4302.61075038228 1.074264

4302.62881650388 1.044136

4302.64688262549 1.06648

4302.66494874709 1.028279

4302.68301486869 1.056259

4302.70108099029 1.038311

4302.7191471119 1.013709

4302.7372132335 1.047161

4302.7552793551 1.02004

4302.7733454767 0.9752167

4302.79141159831 0.9786911

4302.80947771991 0.9897546

4302.82754384151 0.9850101

4302.84560996311 1.003185

4302.86367608472 1.022083

4302.88174220632 0.9927804

4302.89980832792 0.9886933

4302.91787444952 0.987385

4302.93594057112 0.9816772

4302.95400669273 0.9582243

4302.97207281433 0.9676883

4302.99013893593 1.004005

4303.00820505753 0.9434766

4303.02627117913 0.9157696

4303.04433730074 0.9377812

4303.06240342234 0.9174284

4303.08046954394 0.9539692

4303.09853566554 0.9634758

4303.11660178715 0.9385279

4303.13466790875 0.9095044

4303.15273403035 0.8731843

4303.17080015195 0.8683773

4303.18886627356 0.8994825

4303.20693239516 0.9001731

4303.22499851676 0.8720851

4303.24306463836 0.9038219

4303.26113075996 0.9141663

4303.27919688157 0.9033121

4303.29726300317 0.9088733

4303.31532912477 0.8845611

4303.33339524637 0.8617899

4303.35146136797 0.8749503

4303.36952748958 0.8699718

4303.38759361118 0.856847

4303.40565973278 0.8868903

4303.42372585438 0.8873103

4303.44179197598 0.8325295

4303.45985809759 0.8149058

4303.47792421919 0.8243291

4303.49599034079 0.8358358

4303.51405646239 0.8876321

4303.532122584 0.8631313

4303.5501887056 0.8428788

4303.5682548272 0.8998542

4303.5863209488 0.925909

4303.60438707041 0.8839486

4303.62245319201 0.8770106

4303.64051931361 0.9142247

4303.65858543521 0.9069419

4303.67665155681 0.9378585

4303.69471767842 0.8778687

4303.71278380002 0.8561387

4303.73084992162 0.8298222

4303.74891604322 0.871531

4303.76698216483 0.9148773

4303.78504828643 0.9684528

4303.80311440803 0.9555963

4303.82118052963 0.9074471

4303.83924665123 0.9056195

4303.85731277284 0.9657174

4303.87537889444 0.9881175

4303.89344501604 0.9713603

4303.91151113764 1.001705

4303.92957725924 1.029534

4303.94764338085 1.002858

4303.96570950245 0.9297464

4303.98377562405 0.8748027

4304.00184174565 0.8810397

4304.01990786726 0.9426336

4304.03797398886 1.001461

4304.05604011046 0.9731667

4304.07410623206 0.9775891

4304.09217235366 0.9625533

4304.11023847527 0.922356

4304.12830459687 0.9043386

4304.14637071847 0.9508483

4304.16443684007 0.9703707

4304.18250296168 1.011868

4304.20056908328 1.091167

4304.21863520488 1.03891

4304.23670132648 0.9511681

4304.25476744808 1.027358

4304.27283356969 1.067713

4304.29089969129 1.03519

4304.30896581289 1.029858

4304.32703193449 0.9741673

4304.34509805609 0.951297

4304.3631641777 1.002642

4304.3812302993 0.9650674

4304.3992964209 0.9405063

4304.4173625425 0.9659692

4304.43542866411 0.9480776

4304.45349478571 0.953308

4304.47156090731 0.9775199

4304.48962702891 1.010227

4304.50769315051 0.9953375

4304.52575927212 0.9133888

4304.54382539372 0.8971043

4304.56189151532 0.9597909

4304.57995763692 0.9970601

4304.59802375852 0.9472896

4304.61608988013 0.9486809

4304.63415600173 0.9470941

4304.65222212333 0.8881179

4304.67028824493 0.8575116

4304.68835436654 0.9103267

4304.70642048814 0.9345223

4304.72448660974 0.972665

4304.74255273134 1.00107

4304.76061885295 0.9837321

4304.77868497455 0.955501

4304.79675109615 0.9513031

4304.81481721775 0.9695007

4304.83288333935 0.9392085

4304.85094946096 0.9345083

4304.86901558256 0.9345206

4304.88708170416 0.8749585

4304.90514782576 0.8982731

4304.92321394737 0.9455146

4304.94128006897 0.9750777

4304.95934619057 0.9909582

4304.97741231217 0.9978185

4304.99547843377 0.9495349

4305.01354455538 0.8698219

4305.03161067698 0.8923808

4305.04967679858 0.9259571

4305.06774292018 0.9282795

4305.08580904179 0.92273

4305.10387516339 0.9302067

4305.12194128499 0.9818283

4305.14000740659 0.9275186

4305.1580735282 0.9243447

4305.1761396498 1.00256

4305.1942057714 1.012649

4305.212271893 1.000129

4305.2303380146 0.9652398

4305.2484041362 0.9365058

4305.26647025781 0.8633291

4305.28453637941 0.872387

4305.30260250101 0.8441901

4305.32066862261 0.9244121

4305.33873474422 0.9600402

4305.35680086582 0.9839098

4305.37486698742 0.9483374

4305.39293310902 0.9115772

4305.41099923062 0.8783761

4305.42906535223 0.88902

4305.44713147383 0.9136247

4305.46519759543 0.9337615

4305.48326371703 0.8908806

4305.50132983864 0.9392399

4305.51939596024 0.8986552

4305.53746208184 0.7891846

4305.55552820344 0.8523933

4305.57359432505 0.9228144

4305.59166044665 0.9464233

4305.60972656825 0.9692683

4305.62779268985 0.9371191

4305.64585881145 0.9317394

4305.66392493305 0.9575717

4305.68199105466 0.9241989

4305.70005717626 0.9413106

4305.71812329786 1.052513

4305.73618941946 1.064027

4305.75425554107 0.9777806

4305.77232166267 0.9004188

4305.79038778427 0.8909578

4305.80845390587 0.8282448

4305.82652002747 0.8252012

4305.84458614908 0.8411012

4305.86265227068 0.8942057

4305.88071839228 0.94079

4305.89878451388 0.955116

4305.91685063549 0.8983179

4305.93491675709 0.8909266

4305.95298287869 1.04312

4305.97104900029 1.017462

4305.9891151219 0.9735421

4306.0071812435 1.026523

4306.0252473651 0.9854726

4306.0433134867 0.9883801

4306.0613796083 0.9269283

4306.07944572991 0.8787963

4306.09751185151 0.9463714

4306.11557797311 1.00828

4306.13364409471 0.9927143

4306.15171021632 0.9474952

4306.16977633792 0.9236978

4306.18784245952 0.9623023

4306.20590858112 0.9744664

4306.22397470272 0.9526666

4306.24204082432 0.9820933

4306.26010694593 1.037794

4306.27817306753 1.014137

4306.29623918913 0.9762889

4306.31430531073 1.023467

4306.33237143234 1.083393

4306.35043755394 0.9813524

4306.36850367554 0.9903598

4306.38656979714 0.9455315

4306.40463591875 0.9586029

4306.42270204035 0.9782796

4306.44076816195 0.9310211

4306.45883428355 0.8823089

4306.47690040515 0.9055671

4306.49496652676 0.9793212

4306.51303264836 1.0056

4306.53109876996 1.005512

4306.54916489156 0.9577934

4306.56723101317 0.9174004

4306.58529713477 0.9267604

4306.60336325637 0.9288862

4306.62142937797 0.898968

4306.63949549957 0.9990954

4306.65756162118 0.9994301

4306.67562774278 1.016237

4306.69369386438 1.050606

4306.71175998598 1.003951

4306.72982610759 1.077555

4306.74789222919 0.9887337

4306.76595835079 0.8335063

4306.78402447239 0.9553491

4306.80209059399 0.9969472

4306.8201567156 0.8983732

4306.8382228372 0.8652385

4306.8562889588 0.8818823

4306.8743550804 0.9040231

4306.892421202 0.9571153

4306.91048732361 0.8879347

4306.92855344521 0.8503076

4306.94661956681 0.8811454

4306.96468568841 0.9652047

4306.98275181002 0.9251256

4307.00081793162 0.895663

4307.01888405322 0.915264

4307.03695017482 0.9587223

4307.05501629643 0.9793802

4307.07308241803 0.9420177

4307.09114853963 0.8952248

4307.10921466123 0.9241551

4307.12728078283 0.9536039

4307.14534690444 0.9213805

4307.16341302604 0.9314176

4307.18147914764 0.9308574

4307.19954526924 0.951775

4307.21761139084 0.9327166

4307.23567751245 0.9201864

4307.25374363405 0.9260622

4307.27180975565 0.9487049

4307.28987587725 0.9360403

4307.30794199886 0.9453

4307.32600812046 0.9602212

4307.34407424206 0.9251947

4307.36214036366 0.9022655

4307.38020648526 0.9295639

4307.39827260687 0.969299

4307.41633872847 0.9727178

4307.43440485007 0.9628689

4307.45247097167 0.9769531

4307.47053709328 0.980892

4307.48860321488 0.9499856

4307.50666933648 0.9402618

4307.52473545808 0.9789342

4307.54280157968 0.9871195

4307.56086770129 0.9850138

4307.57893382289 0.9574659

4307.59699994449 0.9378356

4307.61506606609 0.9633078

4307.63313218769 0.979686

4307.6511983093 0.9762374

4307.6692644309 0.9595025

4307.6873305525 0.9287617

4307.7053966741 0.9586825

4307.72346279571 0.9551223

4307.74152891731 0.9239173

4307.75959503891 0.915095

4307.77766116051 0.9133832

4307.79572728211 0.9338275

4307.81379340372 0.9256148

4307.83185952532 0.8864968

4307.84992564692 0.8920265

4307.86799176852 0.9173442

4307.88605789013 0.9054394

4307.90412401173 0.8905209

4307.92219013333 0.8962452

4307.94025625493 0.8876147

4307.95832237653 0.87474

4307.97638849814 0.8751438

4307.99445461974 0.8978621

4308.01252074134 0.9140148

4308.03058686294 0.9039522

4308.04865298455 0.9038238

4308.06671910615 0.9254159

4308.08478522775 0.8971517

4308.10285134935 0.8764399

4308.12091747095 0.9008417

4308.13898359256 0.8653138

4308.15704971416 0.8510134

4308.17511583576 0.8681113

4308.19318195736 0.8885169

4308.21124807897 0.8673849

4308.22931420057 0.8670721

4308.24738032217 0.9179436

4308.26544644377 0.9214531

4308.28351256537 0.9109072

4308.30157868698 0.9358355

4308.31964480858 0.9308869

4308.33771093018 0.8407889

4308.35577705178 0.868054

4308.37384317339 0.9044063

4308.39190929499 0.9419873

4308.40997541659 0.9446819

4308.42804153819 0.9579695

4308.44610765979 0.9868708

4308.4641737814 0.9675834

4308.482239903 0.9453604

4308.5003060246 0.9606107

4308.5183721462 0.9435816

4308.53643826781 0.9468482

4308.55450438941 0.9804868

4308.57257051101 0.9730364

4308.59063663261 0.9610306

4308.60870275421 0.9489757

4308.62676887582 0.9223709

4308.64483499742 0.9576371

4308.66290111902 0.9494576

4308.68096724062 0.9377486

4308.69903336223 0.9593515

4308.71709948383 0.9385132

4308.73516560543 1.003555

4308.75323172703 0.9520013

4308.77129784863 0.9010099

4308.78936397024 0.8945184

4308.80743009184 0.9001114

4308.82549621344 0.9226081

4308.84356233504 0.9462382

4308.86162845664 0.9514948

4308.87969457825 0.9422339

4308.89776069985 0.9541311

4308.91582682145 0.9552932

4308.93389294305 0.9489519

4308.95195906466 0.9579121

4308.97002518626 0.915302

4308.98809130786 0.897543

4309.00615742946 0.9525819

4309.02422355106 0.9575936

4309.04228967267 0.9253944

4309.06035579427 0.9119477

4309.07842191587 0.9279209

4309.09648803747 0.9566522

4309.11455415908 0.9423229

4309.13262028068 0.9300487

4309.15068640228 0.9201205

4309.16875252388 0.977129

4309.18681864548 1.019959

4309.20488476709 1.044231

4309.22295088869 0.9451205

4309.24101701029 0.9514427

4309.25908313189 1.019426

4309.2771492535 0.9612271

4309.2952153751 0.9530376

4309.3132814967 0.9709949

4309.3313476183 0.9308804

4309.3494137399 0.9627945

4309.36747986151 1.002437

4309.38554598311 1.007149

4309.40361210471 1.035639

4309.42167822631 1.04367

4309.43974434791 1.031285

4309.45781046952 1.056722

4309.47587659112 1.003021

4309.49394271272 0.9360437

4309.51200883432 0.9699028

4309.53007495593 1.005144

4309.54814107753 1.001748

4309.56620719913 0.962867

4309.58427332073 0.9605907

4309.60233944233 0.9936669

4309.62040556394 0.97058

4309.63847168554 0.9547418

4309.65653780714 0.9604214

4309.67460392874 0.986366

4309.69267005035 1.00176

4309.71073617195 0.9582095

4309.72880229355 0.913772

4309.74686841515 0.9344913

4309.76493453675 0.9756588

4309.78300065836 0.9754877

4309.80106677996 0.9578151

4309.81913290156 0.9809479

4309.83719902316 0.9726416

4309.85526514476 0.9594692

4309.87333126637 0.9620125

4309.89139738797 0.9666054

4309.90946350957 0.9407167

4309.92752963117 0.9244928

4309.94559575278 0.9692174

4309.96366187438 1.010627

4309.98172799598 1.039841

4309.99979411758 0.9993216

4310.01786023918 0.9290197

4310.03592636079 1.001168

4310.05399248239 1.03761

4310.07205860399 1.006606

4310.0901247256 0.9492443

4310.1081908472 0.9559346

4310.1262569688 1.042863

4310.1443230904 1.028347

4310.162389212 0.9731618

4310.18045533361 1.009878

4310.19852145521 1.053374

4310.21658757681 1.053334

4310.23465369841 1.00195

4310.25271982001 0.9803292

4310.27078594162 0.9931444

4310.28885206322 0.9987507

4310.30691818482 0.9890802

4310.32498430642 0.952131

4310.34305042803 0.9441385

4310.36111654963 0.9975946

4310.37918267123 1.02905

4310.39724879283 0.9968926

4310.41531491443 0.9735861

4310.43338103604 0.9884954

4310.45144715764 0.9660469

4310.46951327924 0.9580923

4310.48757940084 0.9908996

4310.50564552245 1.007001

4310.52371164405 0.9788846

4310.54177776565 0.9816177

4310.55984388725 1.006019

4310.57791000885 0.9896634

4310.59597613046 0.9562938

4310.61404225206 0.9495335

4310.63210837366 0.9868385

4310.65017449526 0.9721944

4310.66824061687 0.9533298

4310.68630673847 1.004197

4310.70437286007 1.015976

4310.72243898167 0.992959

4310.74050510327 0.9712809

4310.75857122488 0.9627131

4310.77663734648 1.001171

4310.79470346808 1.020857

4310.81276958968 1.008352

4310.83083571128 0.9928674

4310.84890183289 0.9828956

4310.86696795449 1.026286

4310.88503407609 1.02749

4310.90310019769 0.9887996

4310.9211663193 0.9657922

4310.9392324409 0.9787189

4310.9572985625 1.002489

4310.9753646841 0.9819275

4310.9934308057 0.9858848

4311.01149692731 0.9968422

4311.02956304891 1.0081

4311.04762917051 1.005283

4311.06569529211 0.9833739

4311.08376141371 0.9756386

4311.10182753532 0.9783853

4311.11989365692 0.9724065

4311.13795977852 0.9822071

4311.15602590012 1.009768

4311.17409202173 1.025463

4311.19215814333 1.039822

4311.21022426493 1.068768

4311.22829038653 1.062201

4311.24635650813 0.9998481

4311.26442262974 0.9857158

4311.28248875134 1.017748

4311.30055487294 0.9901249

4311.31862099454 0.9525317

4311.33668711615 0.9555975

4311.35475323775 0.9711589

4311.37281935935 1.009593

4311.39088548095 0.9919527

4311.40895160255 0.9387467

4311.42701772416 0.9759713

4311.44508384576 0.9774721

4311.46314996736 1.016548

4311.48121608896 1.031676

4311.49928221056 0.9648411

4311.51734833217 1.010847

4311.53541445377 0.970831

4311.55348057537 0.926676

4311.57154669697 0.9339837

4311.58961281858 0.9636116

4311.60767894018 0.9551286

4311.62574506178 0.9626086

4311.64381118338 0.9728113

4311.66187730499 1.018971

4311.67994342659 1.039233

4311.69800954819 0.9877548

4311.71607566979 0.9597002

4311.73414179139 0.9837668

4311.752207913 0.9820123

4311.7702740346 0.9910141

4311.7883401562 1.024207

4311.8064062778 1.028569

4311.8244723994 1.038454

4311.84253852101 1.070266

4311.86060464261 0.9910338

4311.87867076421 0.9420403

4311.89673688581 0.9746606

4311.91480300742 0.9404445

4311.93286912902 0.9641935

4311.95093525062 0.9966608

4311.96900137222 0.9648463

4311.98706749382 0.9231831

4312.00513361543 0.9739776

4312.02319973703 0.9803744

4312.04126585863 0.9440776

4312.05933198023 0.9276094

4312.07739810184 0.9554088

4312.09546422344 0.9924701

4312.11353034504 1.002027

4312.13159646664 0.977765

4312.14966258824 0.9642987

4312.16772870985 0.9978576

4312.18579483145 1.022154

4312.20386095305 1.004632

4312.22192707465 0.9779959

4312.23999319626 0.9562845

4312.25805931786 0.9808511

4312.27612543946 0.9957342

4312.29419156106 1.034857

4312.31225768267 1.009951

4312.33032380427 1.020084

4312.34838992587 0.9867188

4312.36645604747 0.9927771

4312.38452216907 1.021908

4312.40258829068 0.9857545

4312.42065441228 0.9956814

4312.43872053388 0.9743088

4312.45678665548 0.9719172

4312.47485277708 0.9894715

4312.49291889869 0.959347

4312.51098502029 0.9671574

4312.52905114189 0.9828329

4312.54711726349 0.9788076

4312.5651833851 0.963547

4312.5832495067 0.9092278

4312.6013156283 0.9185637

4312.6193817499 0.9894369

4312.6374478715 0.9803489

4312.65551399311 0.9396945

4312.67358011471 0.9088297

4312.69164623631 0.933783

4312.70971235791 0.9641829

4312.72777847951 0.9361756

4312.74584460112 0.8997747

4312.76391072272 0.8879925

4312.78197684432 0.9019732

4312.80004296592 0.9308717

4312.81810908753 0.9150263

4312.83617520913 0.8773821

4312.85424133073 0.8944876

4312.87230745233 0.8957705

4312.89037357394 0.8791926

4312.90843969554 0.8870339

4312.92650581714 0.8908682

4312.94457193874 0.8724989

4312.96263806034 0.8491551

4312.98070418195 0.8697937

4312.99877030355 0.8897069

4313.01683642515 0.8351413

4313.03490254675 0.8175756

4313.05296866835 0.8364608

4313.07103478996 0.827846

4313.08910091156 0.8399573

4313.10716703316 0.8733498

4313.12523315476 0.9097097

4313.14329927637 0.895198

4313.16136539797 0.877843

4313.17943151957 0.8794855

4313.19749764117 0.8741455

4313.21556376277 0.8695787

4313.23362988438 0.8846143

4313.25169600598 0.867583

4313.26976212758 0.8522662

4313.28782824918 0.8718551

4313.30589437079 0.8866974

4313.32396049239 0.906508

4313.34202661399 0.9198617

4313.36009273559 0.9213638

4313.37815885719 0.9400683

4313.3962249788 0.9313225

4313.4142911004 0.9229317

4313.432357222 0.9140842

4313.4504233436 0.9343104

4313.4684894652 0.9808915

4313.48655558681 1.008311

4313.50462170841 1.014087

4313.52268783001 0.992604

4313.54075395161 0.9755803

4313.55882007322 0.986532

4313.57688619482 0.9809124

4313.59495231642 0.9995728

4313.61301843802 0.9926426

4313.63108455962 0.9626861

4313.64915068123 0.9654196

4313.66721680283 1.017259

4313.68528292443 1.016196

4313.70334904603 1.001684

4313.72141516764 0.9740693

4313.73948128924 0.9293634

4313.75754741084 0.9689132

4313.77561353244 1.008325

4313.79367965404 0.9962014

4313.81174577565 0.997163

4313.82981189725 1.008251

4313.84787801885 0.9920163

4313.86594414045 0.9780744

4313.88401026206 0.9711542

4313.90207638366 0.9661853

4313.92014250526 0.9939359

4313.93820862686 0.9859517

4313.95627474847 0.9762898

4313.97434087007 0.9739154

4313.99240699167 0.9660076

4314.01047311327 1.001749

4314.02853923487 1.037218

4314.04660535647 1.023618

4314.06467147808 1.014041

4314.08273759968 1.0044

4314.10080372128 0.9686497

4314.11886984288 0.9843934

4314.13693596449 0.9977742

4314.15500208609 0.9473312

4314.17306820769 0.933701

4314.19113432929 0.953146

4314.20920045089 0.9394487

4314.2272665725 0.926855

4314.2453326941 0.9098005

4314.2633988157 0.9329873

4314.2814649373 0.9441676

4314.29953105891 0.9563695

4314.31759718051 0.9494359

4314.33566330211 0.918519

4314.35372942371 0.9203107

4314.37179554531 0.9267118

4314.38986166692 0.8897905

4314.40792778852 0.8840652

4314.42599391012 0.8928494

4314.44406003172 0.897347

4314.46212615333 0.9009534

4314.48019227493 0.8775426

4314.49825839653 0.8943965

4314.51632451813 0.9409955

4314.53439063973 0.9339087

4314.55245676134 0.9079111

4314.57052288294 0.894675

4314.58858900454 0.8935686

4314.60665512614 0.9153576

4314.62472124774 0.9149673

4314.64278736935 0.9066551

4314.66085349095 0.9284486

4314.67891961255 0.9041072

4314.69698573415 0.8803848

4314.71505185576 0.9059139

4314.73311797736 0.8699055

4314.75118409896 0.8882031

4314.76925022056 0.9296666

4314.78731634217 0.9430306

4314.80538246377 0.9134409

4314.82344858537 0.9003404

4314.84151470697 0.9036873

4314.85958082858 0.8937496

4314.87764695018 0.9282572

4314.89571307178 0.9644824

4314.91377919338 0.9433371

4314.93184531498 0.900388

4314.94991143659 0.8838496

4314.96797755819 0.9085455

4314.98604367979 0.9342721

4315.00410980139 0.9127048

4315.02217592299 0.9022197

4315.0402420446 0.9207149

4315.0583081662 0.8888195

4315.0763742878 0.8735214

4315.0944404094 0.8917563

4315.11250653101 0.8741573

4315.13057265261 0.8481399

4315.14863877421 0.8869442

4315.16670489581 0.890439

4315.18477101741 0.8866448

4315.20283713902 0.8682081

4315.22090326062 0.8597207

4315.23896938222 0.8477368

4315.25703550382 0.8603352

4315.27510162543 0.8644018

4315.29316774703 0.8931636

4315.31123386863 0.9161701

4315.32929999023 0.9032931

4315.34736611183 0.9146096

4315.36543223344 0.9225278

4315.38349835504 0.901854

4315.40156447664 0.9283508

4315.41963059824 0.9777173

4315.43769671985 0.9839382

4315.45576284145 0.9571493

4315.47382896305 0.9270292

4315.49189508465 0.947297

4315.50996120625 0.965004

4315.52802732786 0.9807659

4315.54609344946 0.9991055

4315.56415957106 0.9706982

4315.58222569266 0.9755025

4315.60029181426 0.9865508

4315.61835793587 0.9587662

4315.63642405747 0.9315685

4315.65449017907 0.9347243

4315.67255630067 0.966454

4315.69062242228 0.9873116

4315.70868854388 0.9783208

4315.72675466548 0.9956949

4315.74482078708 1.023589

4315.76288690868 1.011482

4315.78095303029 0.9876981

4315.79901915189 0.9915057

4315.81708527349 1.000904

4315.83515139509 0.9840374

4315.8532175167 0.9689455

4315.8712836383 0.9568727

4315.8893497599 0.9592482

4315.9074158815 0.9483558

4315.9254820031 0.9785912

4315.94354812471 0.9967785

4315.96161424631 0.9659595

4315.97968036791 0.9430583

4315.99774648951 0.9756082

4316.01581261111 0.9774299

4316.03387873272 0.9574869

4316.05194485432 0.9707547

4316.07001097592 0.9658895

4316.08807709752 0.9664804

4316.10614321913 0.9730473

4316.12420934073 0.9629474

4316.14227546233 0.9504583

4316.16034158393 0.9604135

4316.17840770553 0.9734768

4316.19647382714 0.976253

4316.21453994874 0.9925729

4316.23260607034 0.9795456

4316.25067219194 0.9406641

4316.26873831355 0.9702915

4316.28680443515 0.9642869

4316.30487055675 0.9291768

4316.32293667835 0.9500289

4316.34100279995 0.9432763

4316.35906892156 0.9477408

4316.37713504316 0.9814878

4316.39520116476 0.9674789

4316.41326728636 0.9566319

4316.43133340797 0.9736563

4316.44939952957 0.9650564

4316.46746565117 0.9630728

4316.48553177277 0.9734788

4316.50359789437 0.9685535

4316.52166401598 0.9716663

4316.53973013758 0.9801872

4316.55779625918 0.976401

4316.57586238078 0.9503169

4316.59392850239 0.9631838

4316.61199462399 1.001014

4316.63006074559 0.9946198

4316.64812686719 0.9615027

4316.66619298879 0.9663607

4316.6842591104 0.9704386

4316.702325232 0.9422969

4316.7203913536 0.9396349

4316.7384574752 0.9569284

4316.75652359681 0.9329082

4316.77458971841 0.943868

4316.79265584001 0.9485458

4316.81072196161 0.9546776

4316.82878808321 0.9463738

4316.84685420482 0.9100055

4316.86492032642 0.9236399

4316.88298644802 0.9350494

4316.90105256962 0.9417286

4316.91911869122 0.9457557

4316.93718481283 0.9258695

4316.95525093443 0.9190952

4316.97331705603 0.9530552

4316.99138317763 0.9699012

4317.00944929924 0.935012

4317.02751542084 0.9029288

4317.04558154244 0.9162397

4317.06364766404 0.9414874

4317.08171378565 0.931437

4317.09977990725 0.9116469

4317.11784602885 0.9168817

4317.13591215045 0.9267813

4317.15397827205 0.9291415

4317.17204439366 0.9004455

4317.19011051526 0.9039513

4317.20817663686 0.9373301

4317.22624275846 0.9468081

4317.24430888006 0.901805

4317.26237500167 0.8702291

4317.28044112327 0.8564737

4317.29850724487 0.8848574

4317.31657336647 0.9247389

4317.33463948808 0.9151766

4317.35270560968 0.9077525

4317.37077173128 0.9433671

4317.38883785288 0.9443958

4317.40690397448 0.9384191

4317.42497009609 0.9503759

4317.44303621769 0.9286119

4317.46110233929 0.9277223

4317.47916846089 0.9382572

4317.4972345825 0.9524066

4317.5153007041 0.9590068

4317.5333668257 0.9377024

4317.5514329473 0.9235083

4317.5694990689 0.9481893

4317.58756519051 0.9507962

4317.60563131211 0.931399

4317.62369743371 0.9378379

4317.64176355531 0.9154201

4317.65982967691 0.89748

4317.67789579852 0.9460335

4317.69596192012 0.9777684

4317.71402804172 0.9542678

4317.73209416332 0.9521836

4317.75016028493 0.9695306

4317.76822640653 0.9882305

4317.78629252813 0.9766428

4317.80435864973 0.9627604

4317.82242477133 0.9676811

4317.84049089294 0.984113

4317.85855701454 0.9779152

4317.87662313614 0.9439716

4317.89468925774 0.9538473

4317.91275537935 0.9905366

4317.93082150095 0.9982129

4317.94888762255 0.9398651

4317.96695374415 0.960413

4317.98501986575 0.9719321

4318.00308598736 0.9836886

4318.02115210896 1.005261

4318.03921823056 1.003326

4318.05728435216 0.9748734

4318.07535047377 0.9553815

4318.09341659537 0.9911525

4318.11148271697 0.987736

4318.12954883857 0.9686589

4318.14761496017 0.951713

4318.16568108178 0.9430999

4318.18374720338 0.9936279

4318.20181332498 1.00825

4318.21987944658 1.004209

4318.23794556819 0.9749531

4318.25601168979 0.9485109

4318.27407781139 0.9576247

4318.29214393299 0.9613551

4318.31021005459 0.9490228

4318.3282761762 0.9569615

4318.3463422978 0.9641

4318.3644084194 0.9495865

4318.382474541 0.9546629

4318.4005406626 0.9741963

4318.41860678421 0.9961867

4318.43667290581 1.013403

4318.45473902741 1.017918

4318.47280514901 0.9730569

4318.49087127062 0.9355494

4318.50893739222 0.9357698

4318.52700351382 0.9533232

4318.54506963542 0.9896621

4318.56313575702 0.9831114

4318.58120187863 0.9968438

4318.59926800023 0.9927187

4318.61733412183 0.952635

4318.63540024343 0.9419657

4318.65346636504 0.9518544

4318.67153248664 0.9692497

4318.68959860824 0.9684297

4318.70766472984 0.9675813

4318.72573085145 0.9912492

4318.74379697305 1.014423

4318.76186309465 1.012619

4318.77992921625 1.00055

4318.79799533785 0.9891686

4318.81606145945 0.9825443

4318.83412758106 0.9847307

4318.85219370266 0.967365

4318.87025982426 0.9546724

4318.88832594587 0.9837532

4318.90639206747 0.9964095

4318.92445818907 0.9672818

4318.94252431067 0.968985

4318.96059043227 0.9972137

4318.97865655388 1.010865

4318.99672267548 1.007619

4319.01478879708 1.006464

4319.03285491868 1.00838

4319.05092104029 1.021567

4319.06898716189 1.002778

4319.08705328349 0.9725392

4319.10511940509 0.9542566

4319.12318552669 0.9462417

4319.1412516483 0.9745854

4319.1593177699 0.9893832

4319.1773838915 0.9675652

4319.1954500131 0.9525143

4319.2135161347 0.92102

4319.23158225631 0.9477868

4319.24964837791 0.9675064

4319.26771449951 0.95468

4319.28578062111 0.9669539

4319.30384674272 0.9755931

4319.32191286432 0.9723423

4319.33997898592 0.9792529

4319.35804510752 0.9709931

4319.37611122912 0.9412828

4319.39417735073 0.9376577

4319.41224347233 0.9592429

4319.43030959393 0.9624331

4319.44837571553 0.9566215

4319.46644183713 0.9548267

4319.48450795874 0.9432795

4319.50257408034 0.924823

4319.52064020194 0.921068

4319.53870632354 0.9349599

4319.55677244515 0.931985

4319.57483856675 0.9485993

4319.59290468835 0.9384151

4319.61097080995 0.9412736

4319.62903693155 0.9729786

4319.64710305316 0.9777732

4319.66516917476 0.9484903

4319.68323529636 0.9343758

4319.70130141796 0.9291142

4319.71936753957 0.9228209

4319.73743366117 0.9284599

4319.75549978277 0.940289

4319.77356590437 0.9360729

4319.79163202598 0.9179931

4319.80969814758 0.9131905

4319.82776426918 0.9479835

4319.84583039078 0.9625611

4319.86389651238 0.9302033

4319.88196263399 0.9297692

4319.90002875559 0.9447286

4319.91809487719 0.9337422

4319.93616099879 0.9367899

4319.95422712039 0.9623858

4319.972293242 0.968789

4319.9903593636 0.9422277

4320.0084254852 0.9560867

4320.0264916068 0.9752051

4320.04455772841 0.9394487

4320.06262385001 0.9467487

4320.08068997161 0.9672205

4320.09875609321 0.9622804

4320.11682221481 0.9422532

4320.13488833642 0.9509918

4320.15295445802 0.9912788

4320.17102057962 0.9938836

4320.18908670122 0.9730657

4320.20715282282 0.9781798

4320.22521894443 0.9978073

4320.24328506603 0.9847273

4320.26135118763 0.9408105

4320.27941730923 0.9543216

4320.29748343084 0.9842637

4320.31554955244 0.9929154

4320.33361567404 1.008689

4320.35168179564 1.018805

4320.36974791724 0.9768537

4320.38781403885 0.9952193

4320.40588016045 1.007225

4320.42394628205 0.978891

4320.44201240365 0.9722891

4320.46007852526 0.9875422

4320.47814464686 0.9797722

4320.49621076846 0.9447192

4320.51427689006 0.9449024

4320.53234301166 0.9616448

4320.55040913327 0.9376683

4320.56847525487 0.9313772

4320.58654137647 0.9622252

4320.60460749807 0.9679779

4320.62267361968 0.9386113

4320.64073974128 0.952171

4320.65880586288 0.9454632

4320.67687198448 0.9520991

4320.69493810608 0.9668062

4320.71300422769 0.9780687

4320.73107034929 0.9680038

4320.74913647089 0.9804634

4320.76720259249 0.9757486

4320.7852687141 0.9288045

4320.8033348357 0.92661

4320.8214009573 0.9265908

4320.8394670789 0.9178447

4320.8575332005 0.9167465

4320.87559932211 0.9239612

4320.89366544371 0.8984947

4320.91173156531 0.9043333

4320.92979768691 0.9281336

4320.94786380852 0.9496899

4320.96592993012 0.9449717

4320.98399605172 0.9260859

4321.00206217332 0.9067935

4321.02012829492 0.8922554

4321.03819441653 0.9047035

4321.05626053813 0.8865042

4321.07432665973 0.8912824

4321.09239278133 0.901787

4321.11045890293 0.8836429

4321.12852502454 0.8722266

4321.14659114614 0.882547

4321.16465726774 0.8908576

4321.18272338934 0.9043579

4321.20078951095 0.8800207

4321.21885563255 0.8741968

4321.23692175415 0.9015088

4321.25498787575 0.9081711

4321.27305399736 0.9258585

4321.29112011896 0.939234

4321.30918624056 0.9123117

4321.32725236216 0.8817614

4321.34531848376 0.870923

4321.36338460536 0.8898258

4321.38145072697 0.9268516

4321.39951684857 0.9489083

4321.41758297017 0.9186707

4321.43564909177 0.892158

4321.45371521338 0.9237406

4321.47178133498 0.9283053

4321.48984745658 0.907631

4321.50791357818 0.9247772

4321.52597969978 0.9370862

4321.54404582139 0.9429356

4321.56211194299 0.9557504

4321.58017806459 0.9562352

4321.59824418619 0.978038

4321.6163103078 0.9746954

4321.6343764294 0.9654616

4321.652442551 0.9510079

4321.6705086726 0.965661

4321.68857479421 0.9657647

4321.70664091581 0.945798

4321.72470703741 0.9756322

4321.74277315901 0.9657927

4321.76083928061 0.9714032

4321.77890540222 0.9721907

4321.79697152382 0.9776942

4321.81503764542 0.9633766

4321.83310376702 0.9643302

4321.85116988862 0.9877176

4321.86923601023 0.9771373

4321.88730213183 0.9639328

4321.90536825343 0.9679568

4321.92343437503 0.9775376

4321.94150049664 0.987273

4321.95956661824 0.9859333

4321.97763273984 0.9802237

4321.99569886144 1.005637

4322.01376498304 0.9892452

4322.03183110465 0.9584537

4322.04989722625 0.9784341

4322.06796334785 0.9570298

4322.08602946945 0.9853721

4322.10409559106 1.035344

4322.12216171266 0.9969911

4322.14022783426 0.9561018

4322.15829395586 0.9526687

4322.17636007747 0.9742461

4322.19442619907 0.9636911

4322.21249232067 0.9579078

4322.23055844227 0.9724543

4322.24862456387 0.9802313

4322.26669068548 0.9775385

4322.28475680708 0.9557441

4322.30282292868 0.9479891

4322.32088905028 0.9814304

4322.33895517189 1.01525

4322.35702129349 1.016561

4322.37508741509 0.9900277

4322.39315353669 0.9712607

4322.41121965829 0.9650654

4322.42928577989 0.979032

4322.4473519015 0.9959806

4322.4654180231 1.001536

4322.4834841447 1.005616

4322.5015502663 0.9826291

4322.51961638791 0.9703302

4322.53768250951 0.9631727

4322.55574863111 0.9464915

4322.57381475271 0.9591291

4322.59188087431 0.9740313

4322.60994699592 1.000436

4322.62801311752 1.022504

4322.64607923912 0.9864728

4322.66414536072 0.9636158

4322.68221148233 0.9520473

4322.70027760393 0.9414027

4322.71834372553 0.954187

4322.73640984713 0.9301175

4322.75447596873 0.9195623

4322.77254209034 0.9560518

4322.79060821194 0.9784566

4322.80867433354 0.967067

4322.82674045514 0.9660944

4322.84480657675 0.9604613

4322.86287269835 0.9749492

4322.88093881995 0.9829071

4322.89900494155 0.968343

4322.91707106315 0.9514015

4322.93513718476 0.9544373

4322.95320330636 0.9655252

4322.97126942796 0.9612707

4322.98933554956 0.9757829

4323.00740167117 0.9728956

4323.02546779277 0.9543469

4323.04353391437 0.9642791

4323.06160003597 0.9491838

4323.07966615757 0.9458796

4323.09773227918 0.9702423

4323.11579840078 0.9629126

4323.13386452238 0.958637

4323.15193064398 0.9349298

4323.16999676558 0.9187928

4323.18806288719 0.9414816

4323.20612900879 0.9324844

4323.22419513039 0.9378741

4323.24226125199 0.9388527

4323.2603273736 0.9629089

4323.2783934952 0.9657704

4323.2964596168 0.9478695

4323.3145257384 0.9394698

4323.33259186 0.9571253

4323.35065798161 0.969457

4323.36872410321 0.9676875

4323.38679022481 0.9545122

4323.40485634641 0.9386379

4323.42292246802 0.9520597

4323.44098858962 0.9639166

4323.45905471122 0.96464

4323.47712083282 0.9617736

4323.49518695443 0.955453

4323.51325307603 0.9735918

4323.53131919763 0.9560573

4323.54938531923 0.9373889

4323.56745144083 0.9523566

4323.58551756244 0.9652517

4323.60358368404 0.9500231

4323.62164980564 0.9365975

4323.63971592724 0.9641851

4323.65778204885 0.9855003

4323.67584817045 0.9755585

4323.69391429205 0.9690092

4323.71198041365 0.9566383

4323.73004653525 0.9623467

4323.74811265686 0.9818711

4323.76617877846 0.9565493

4323.78424490006 0.9216528

4323.80231102166 0.9271983

4323.82037714326 0.9450763

4323.83844326487 0.9810775

4323.85650938647 0.9870614

4323.87457550807 0.96031

4323.89264162967 0.9679214

4323.91070775128 0.9804608

4323.92877387288 0.963352

4323.94683999448 0.9534709

4323.96490611608 0.9606341

4323.98297223769 0.9499117

4324.00103835929 0.934463

4324.01910448089 0.9478308

4324.03717060249 0.9686227

4324.05523672409 0.977639

4324.0733028457 0.9436224

4324.0913689673 0.9472873

4324.1094350889 0.9629779

4324.1275012105 0.9470372

4324.1455673321 0.9313083

4324.16363345371 0.9388375

4324.18169957531 0.9541326

4324.19976569691 0.9766436

4324.21783181851 0.9531808

4324.23589794012 0.9116719

4324.25396406172 0.9104531

4324.27203018332 0.9292996

4324.29009630492 0.9653682

4324.30816242652 0.9633635

4324.32622854813 0.9267379

4324.34429466973 0.919614

4324.36236079133 0.9293242

4324.38042691293 0.9476084

4324.39849303453 0.9467518

4324.41655915614 0.9230367

4324.43462527774 0.9206678

4324.45269139934 0.9338053

4324.47075752094 0.9412154

4324.48882364255 0.9455653

4324.50688976415 0.9269172

4324.52495588575 0.9141281

4324.54302200735 0.9233855

4324.56108812895 0.9248517

4324.57915425056 0.932773

4324.59722037216 0.9683851

4324.61528649376 0.9778665

4324.63335261536 0.9571856

4324.65141873697 0.9544376

4324.66948485857 0.9405605

4324.68755098017 0.9520056

4324.70561710177 0.9473011

4324.72368322338 0.9421871

4324.74174934498 0.9506931

4324.75981546658 0.9472703

4324.77788158818 0.9243675

4324.79594770978 0.9248735

4324.81401383139 0.9333153

4324.83207995299 0.9400165

4324.85014607459 0.9363777

4324.86821219619 0.9326406

4324.88627831779 0.9287218

4324.9043444394 0.9163087

4324.922410561 0.9162263

4324.9404766826 0.9367958

4324.9585428042 0.9286795

4324.97660892581 0.9322156

4324.99467504741 0.9149158

4325.01274116901 0.9099437

4325.03080729061 0.9305183

4325.04887341221 0.9119084

4325.06693953382 0.9116514

4325.08500565542 0.9126899

4325.10307177702 0.8947655

4325.12113789862 0.9000964

4325.13920402023 0.9197282

4325.15727014183 0.9183643

4325.17533626343 0.9125627

4325.19340238503 0.9297616

4325.21146850663 0.9256148

4325.22953462824 0.932543

4325.24760074984 0.9365547

4325.26566687144 0.8977636

4325.28373299304 0.8798244

4325.30179911464 0.9043124

4325.31986523625 0.9143546

4325.33793135785 0.8970219

4325.35599747945 0.8942847

4325.37406360105 0.8972036

4325.39212972266 0.8629717

4325.41019584426 0.8406877

4325.42826196586 0.8588189

4325.44632808746 0.9004139

4325.46439420907 0.8952895

4325.48246033067 0.8444424

4325.50052645227 0.8420272

4325.51859257387 0.8532081

4325.53665869547 0.8464257

4325.55472481707 0.845893

4325.57279093868 0.8071454

4325.59085706028 0.831943

4325.60892318188 0.8802624

4325.62698930348 0.8726847

4325.64505542509 0.8614974

4325.66312154669 0.8244861

4325.68118766829 0.8439943

4325.69925378989 0.8311504

4325.71731991149 0.8215443

4325.7353860331 0.8114044

4325.7534521547 0.8086827

4325.7715182763 0.7965387

4325.7895843979 0.8094869

4325.80765051951 0.8387319

4325.82571664111 0.8149638

4325.84378276271 0.8010573

4325.86184888431 0.8162511

4325.87991500592 0.8170133

4325.89798112752 0.8091152

4325.91604724912 0.8059156

4325.93411337072 0.787378

4325.95217949232 0.7726941

4325.97024561393 0.8083663

4325.98831173553 0.839259

4326.00637785713 0.8231899

4326.02444397873 0.8011253

4326.04251010033 0.8072578

4326.06057622194 0.8111968

4326.07864234354 0.8185032

4326.09670846514 0.8388495

4326.11477458674 0.8538903

4326.13284070835 0.8552701

4326.15090682995 0.8504399

4326.16897295155 0.8635204

4326.18703907315 0.871263

4326.20510519475 0.8239017

4326.22317131636 0.8354628

4326.24123743796 0.8817047

4326.25930355956 0.8939852

4326.27736968116 0.8420483

4326.29543580277 0.8229486

4326.31350192437 0.8408641

4326.33156804597 0.8756493

4326.34963416757 0.8963671

4326.36770028917 0.9000851

4326.38576641078 0.8913532

4326.40383253238 0.9107152

4326.42189865398 0.9203591

4326.43996477558 0.9062811

4326.45803089719 0.8730431

4326.47609701879 0.8998134

4326.49416314039 0.9087688

4326.51222926199 0.8838371

4326.53029538359 0.8638856

4326.5483615052 0.866136

4326.5664276268 0.8700304

4326.5844937484 0.8502312

4326.60255987 0.8495702

4326.6206259916 0.8589793

4326.63869211321 0.8254254

4326.65675823481 0.8271754

4326.67482435641 0.8304481

4326.69289047801 0.8435083

4326.71095659962 0.8300662

4326.72902272122 0.7858108

4326.74708884282 0.8440552

4326.76515496442 0.8875983

4326.78322108602 0.8581495

4326.80128720763 0.8676981

4326.81935332923 0.8944589

4326.83741945083 0.8953195

4326.85548557243 0.8823857

4326.87355169404 0.8734143

4326.89161781564 0.8664715

4326.90968393724 0.8576885

4326.92775005884 0.8660588

4326.94581618045 0.8807952

4326.96388230205 0.8614601

4326.98194842365 0.8375769

4327.00001454525 0.8515089

4327.01808066685 0.8563581

4327.03614678846 0.8480958

4327.05421291006 0.8526074

4327.07227903166 0.8521144

4327.09034515326 0.8678288

4327.10841127487 0.8754652

4327.12647739647 0.8789953

4327.14454351807 0.8804984

4327.16260963967 0.8660407

4327.18067576127 0.8782929

4327.19874188288 0.874724

4327.21680800448 0.8992127

4327.23487412608 0.9063119

4327.25294024768 0.8916305

4327.27100636929 0.8861309

4327.28907249089 0.8978424

4327.30713861249 0.8892311

4327.32520473409 0.8773844

4327.34327085569 0.9100505

4327.3613369773 0.9329237

4327.3794030989 0.896162

4327.3974692205 0.87905

4327.4155353421 0.8909786

4327.4336014637 0.9099684

4327.45166758531 0.9153932

4327.46973370691 0.9106501

4327.48779982851 0.9198117

4327.50586595011 0.92105

4327.52393207172 0.9076803

4327.54199819332 0.8826082

4327.56006431492 0.8735735

4327.57813043652 0.900026

4327.59619655812 0.9201258

4327.61426267973 0.8814341

4327.63232880133 0.8858324

4327.65039492293 0.9083859

4327.66846104453 0.9134426

4327.68652716613 0.9139485

4327.70459328774 0.9267045

4327.72265940934 0.9340371

4327.74072553094 0.9130181

4327.75879165254 0.8933728

4327.77685777415 0.8829136

4327.79492389575 0.8891563

4327.81299001735 0.8887321

4327.83105613895 0.884853

4327.84912226055 0.910768

4327.86718838216 0.9431485

4327.88525450376 0.9620163

4327.90332062536 0.9241604

4327.92138674696 0.9064412

4327.93945286857 0.9113443

4327.95751899017 0.881977

4327.97558511177 0.8787374

4327.99365123337 0.8698437

4328.01171735497 0.872746

4328.02978347658 0.8881822

4328.04784959818 0.8888588

4328.06591571978 0.8893477

4328.08398184138 0.8750302

4328.10204796299 0.8799202

4328.12011408459 0.9002285

4328.13818020619 0.8898802

4328.15624632779 0.8868678

4328.17431244939 0.8539076

4328.192378571 0.8790044

4328.2104446926 0.8940831

4328.2285108142 0.8758091

4328.2465769358 0.8774928

4328.2646430574 0.8621654

4328.28270917901 0.8504076

4328.30077530061 0.8804709

4328.31884142221 0.8826492

4328.33690754381 0.8695734

4328.35497366542 0.8770299

4328.37303978702 0.8495414

4328.39110590862 0.8676876

4328.40917203022 0.9085399

4328.42723815183 0.8940657

4328.44530427343 0.8510436

4328.46337039503 0.8493683

4328.48143651663 0.860611

4328.49950263823 0.8706016

4328.51756875984 0.8938463

4328.53563488144 0.8721069

4328.55370100304 0.8624802

4328.57176712464 0.8917528

4328.58983324624 0.8857778

4328.60789936785 0.8611413

4328.62596548945 0.8748235

4328.64403161105 0.8946617

4328.66209773265 0.8897254

4328.68016385426 0.8982512

4328.69822997586 0.9235663

4328.71629609746 0.9052939

4328.73436221906 0.9228841

4328.75242834066 0.928111

4328.77049446227 0.8912514

4328.78856058387 0.8747846

4328.80662670547 0.9114882

4328.82469282707 0.9372854

4328.84275894868 0.9148002

4328.86082507028 0.8818605

4328.87889119188 0.8767157

4328.89695731348 0.8780028

4328.91502343509 0.8737869

4328.93308955669 0.8925467

4328.95115567829 0.9148009

4328.96922179989 0.9190906

4328.98728792149 0.9089596

4329.0053540431 0.8974768

4329.0234201647 0.8737731

4329.0414862863 0.8759383

4329.0595524079 0.8827935

4329.07761852951 0.885023

4329.09568465111 0.8857898

4329.11375077271 0.8841727

4329.13181689431 0.8641616

4329.14988301591 0.8701094

4329.16794913752 0.8896347

4329.18601525912 0.8725301

4329.20408138072 0.8675858

4329.22214750232 0.8685513

4329.24021362392 0.8747759

4329.25827974553 0.8833464

4329.27634586713 0.8944724

4329.29441198873 0.8709624

4329.31247811033 0.8418748

4329.33054423194 0.8468415

4329.34861035354 0.8527341

4329.36667647514 0.8308775

4329.38474259674 0.8460204

4329.40280871834 0.877227

4329.42087483995 0.8502803

4329.43894096155 0.8404164

4329.45700708315 0.8753

4329.47507320475 0.8944889

4329.49313932636 0.8831396

4329.51120544796 0.85747

4329.52927156956 0.8566601

4329.54733769116 0.8879242

4329.56540381276 0.8760451

4329.58346993437 0.8716501

4329.60153605597 0.890017

4329.61960217757 0.8742626

4329.63766829917 0.8503207

4329.65573442077 0.8355621

4329.67380054238 0.8432778

4329.69186666398 0.8363066

4329.70993278558 0.8537246

4329.72799890718 0.8625869

4329.74606502879 0.874041

4329.76413115039 0.8626029

4329.78219727199 0.8289558

4329.80026339359 0.8081908

4329.81832951519 0.8209684

4329.8363956368 0.8462469

4329.8544617584 0.8754997

4329.87252788 0.8669561

4329.8905940016 0.8485316

4329.90866012321 0.837364

4329.92672624481 0.8235565

4329.94479236641 0.8203261

4329.96285848801 0.824141

4329.98092460961 0.820127

4329.99899073122 0.8156655

4330.01705685282 0.8247107

4330.03512297442 0.7951161

4330.05318909602 0.7967857

4330.07125521763 0.8280616

4330.08932133923 0.8512096

4330.10738746083 0.8220052

4330.12545358243 0.8060419

4330.14351970403 0.8106661

4330.16158582564 0.8051225

4330.17965194724 0.7754326

4330.19771806884 0.8287662

4330.21578419044 0.8694293

4330.23385031204 0.8237554

4330.25191643365 0.7849287

4330.26998255525 0.7664806

4330.28804867685 0.7374594

4330.30611479845 0.7590943

4330.32418092006 0.7707831

4330.34224704166 0.7720964

4330.36031316326 0.7702392

4330.37837928486 0.7773787

4330.39644540646 0.7651669

4330.41451152807 0.7308174

4330.43257764967 0.742616

4330.45064377127 0.7738335

4330.46870989287 0.8093047

4330.48677601448 0.8370295

4330.50484213608 0.8111005

4330.52290825768 0.7925395

4330.54097437928 0.8084921

4330.55904050088 0.7675295

4330.57710662249 0.7855229

4330.59517274409 0.7701997

4330.61323886569 0.7567601

4330.63130498729 0.7725074

4330.6493711089 0.7982184

4330.6674372305 0.8054461

4330.6855033521 0.7715112

4330.7035694737 0.8047665

4330.7216355953 0.7724944

4330.73970171691 0.7618128

4330.75776783851 0.7926608

4330.77583396011 0.8085351

4330.79390008171 0.8086846

4330.81196620331 0.7882175

4330.83003232492 0.7841624

4330.84809844652 0.802042

4330.86616456812 0.7824442

4330.88423068972 0.7455696

4330.90229681133 0.7836903

4330.92036293293 0.8323902

4330.93842905453 0.8235772

4330.95649517613 0.8161026

4330.97456129773 0.7976217

4330.99262741934 0.7754438

4331.01069354094 0.7829537

4331.02875966254 0.806916

4331.04682578414 0.7909181

4331.06489190575 0.8124847

4331.08295802735 0.8197197

4331.10102414895 0.8125823

4331.11909027055 0.7802303

4331.13715639215 0.7871156

4331.15522251376 0.799552

4331.17328863536 0.8273014

4331.19135475696 0.832373

4331.20942087856 0.8218409

4331.22748700017 0.8346349

4331.24555312177 0.8218714

4331.26361924337 0.834699

4331.28168536497 0.8461273

4331.29975148658 0.8413873

4331.31781760818 0.8460709

4331.33588372978 0.8420262

4331.35394985138 0.8006281

4331.37201597298 0.8108077

4331.39008209458 0.8417823

4331.40814821619 0.8286985

4331.42621433779 0.811988

4331.44428045939 0.8191587

4331.46234658099 0.8080707

4331.4804127026 0.8015632

4331.4984788242 0.7823944

4331.5165449458 0.7782305

4331.5346110674 0.7960845

4331.552677189 0.7899337

4331.57074331061 0.8118122

4331.58880943221 0.793361

4331.60687555381 0.8026688

4331.62494167542 0.8178585

4331.64300779702 0.8124202

4331.66107391862 0.8440892

4331.67914004022 0.8434768

4331.69720616182 0.825285

4331.71527228343 0.8260511

4331.73333840503 0.8399261

4331.75140452663 0.8155516

4331.76947064823 0.7775009

4331.78753676983 0.7861744

4331.80560289144 0.8220325

4331.82366901304 0.8328286

4331.84173513464 0.7958927

4331.85980125624 0.7932572

4331.87786737785 0.8351467

4331.89593349945 0.8478017

4331.91399962105 0.8175629

4331.93206574265 0.8038335

4331.95013186425 0.8158464

4331.96819798586 0.809548

4331.98626410746 0.7961

4332.00433022906 0.8191807

4332.02239635066 0.8291753

4332.04046247227 0.7877387

4332.05852859387 0.7775587

4332.07659471547 0.8133076

4332.09466083707 0.8561658

4332.11272695867 0.8438854

4332.13079308028 0.8559006

4332.14885920188 0.8270235

4332.16692532348 0.8284365

4332.18499144508 0.8282975

4332.20305756668 0.8321844

4332.22112368829 0.8609825

4332.23918980989 0.8388433

4332.25725593149 0.8269238

4332.27532205309 0.8017365

4332.2933881747 0.8373172

4332.3114542963 0.8868936

4332.3295204179 0.8222851

4332.3475865395 0.7918069

4332.3656526611 0.8608816

4332.38371878271 0.8636144

4332.40178490431 0.8315591

4332.41985102591 0.8389671

4332.43791714751 0.8802536

4332.45598326912 0.8665135

4332.47404939072 0.8113049

4332.49211551232 0.8242584

4332.51018163392 0.82862

4332.52824775552 0.8088287

4332.54631387713 0.8173604

4332.56437999873 0.8249436

4332.58244612033 0.8444672

4332.60051224193 0.837118

4332.61857836354 0.8305732

4332.63664448514 0.7773222

4332.65471060674 0.7952942

4332.67277672834 0.8440768

4332.69084284994 0.8171141

4332.70890897155 0.7877967

4332.72697509315 0.7824211

4332.74504121475 0.7853556

4332.76310733635 0.7620799

4332.78117345795 0.7776043

4332.79923957956 0.8155346

4332.81730570116 0.7928766

4332.83537182276 0.806558

4332.85343794436 0.8011438

4332.87150406597 0.8223082

4332.88957018757 0.840201

4332.90763630917 0.8178989

4332.92570243077 0.7878058

4332.94376855237 0.8185019

4332.96183467398 0.8248836

4332.97990079558 0.8007731

4332.99796691718 0.8070791

4333.01603303878 0.8352115

4333.03409916038 0.8272661

4333.05216528199 0.7824696

4333.07023140359 0.8019372

4333.08829752519 0.8001325

4333.10636364679 0.7775526

4333.1244297684 0.784278

4333.14249589 0.8236359

4333.1605620116 0.8056567

4333.1786281332 0.797361

4333.19669425481 0.8141656

4333.21476037641 0.8028059

4333.23282649801 0.8008838

4333.25089261961 0.7919816

4333.26895874121 0.7782385

4333.28702486282 0.7800912

4333.30509098442 0.781821

4333.32315710602 0.7840099

4333.34122322762 0.7929895

4333.35928934923 0.8088505

4333.37735547083 0.8079119

4333.39542159243 0.8347023

4333.41348771403 0.81058

4333.43155383563 0.7947595

4333.44961995724 0.8272256

4333.46768607884 0.8150303

4333.48575220044 0.7968544

4333.50381832204 0.7884053

4333.52188444365 0.8071747

4333.53995056525 0.8152723

4333.55801668685 0.7764369

4333.57608280845 0.7553252

4333.59414893005 0.7764647

4333.61221505166 0.8061769

4333.63028117326 0.8136792

4333.64834729486 0.8076541

4333.66641341646 0.8005038

4333.68447953807 0.8044177

4333.70254565967 0.7774652

4333.72061178127 0.7802725

4333.73867790287 0.8089821

4333.75674402447 0.8518066

4333.77481014608 0.8363442

4333.79287626768 0.8046325

4333.81094238928 0.7858732

4333.82900851088 0.8562864

4333.84707463248 0.8523432

4333.86514075409 0.8183398

4333.88320687569 0.8120563

4333.90127299729 0.8165288

4333.91933911889 0.8148607

4333.9374052405 0.7933131

4333.9554713621 0.7877629

4333.9735374837 0.8173337

4333.9916036053 0.8533437

4334.0096697269 0.8495548

4334.02773584851 0.8212301

4334.04580197011 0.7783146

4334.06386809171 0.7664587

4334.08193421331 0.811934

4334.10000033492 0.8212348

4334.11806645652 0.7926804

4334.13613257812 0.7857362

4334.15419869972 0.7754163

4334.17226482132 0.7764626

4334.19033094293 0.808915

4334.20839706453 0.799816

4334.22646318613 0.8432777

4334.24452930773 0.8336426

4334.26259542934 0.793372

4334.28066155094 0.7733452

4334.29872767254 0.8479998

4334.31679379414 0.85884

4334.33485991574 0.798591

4334.35292603735 0.7944503

4334.37099215895 0.8153659

4334.38905828055 0.8403827

4334.40712440215 0.8438699

4334.42519052375 0.7722103

4334.44325664536 0.7574972

4334.46132276696 0.8074281

4334.47938888856 0.8180587

4334.49745501016 0.7999175

4334.51552113177 0.8006558

4334.53358725337 0.790326

4334.55165337497 0.7482244

4334.56971949657 0.7397792

4334.58778561817 0.7479063

4334.60585173978 0.7692845

4334.62391786138 0.7477096

4334.64198398298 0.7232248

4334.66005010458 0.7528473

4334.67811622619 0.7833563

4334.69618234779 0.7794983

4334.71424846939 0.7987248

4334.73231459099 0.7392954

4334.75038071259 0.721212

4334.7684468342 0.7454083

4334.7865129558 0.7723302

4334.8045790774 0.7878984

4334.822645199 0.8130346

4334.84071132061 0.8081523

4334.85877744221 0.7920482

4334.87684356381 0.7709928

4334.89490968541 0.7373483

4334.91297580701 0.7485914

4334.93104192862 0.7545058

4334.94910805022 0.7353404

4334.96717417182 0.7269042

4334.98524029342 0.7773511

4335.00330641502 0.7677744

4335.02137253663 0.7281698

4335.03943865823 0.713937

4335.05750477983 0.7714252

4335.07557090143 0.7772405

4335.09363702304 0.7505419

4335.11170314464 0.7905583

4335.12976926624 0.7761272

4335.14783538784 0.7279267

4335.16590150944 0.7193595

4335.18396763105 0.7314771

4335.20203375265 0.7407169

4335.22009987425 0.724836

4335.23816599585 0.7277856

4335.25623211746 0.7081496

4335.27429823906 0.7267005

4335.29236436066 0.7560801

4335.31043048226 0.7528299

4335.32849660386 0.7247635

4335.34656272547 0.7301333

4335.36462884707 0.7296697

4335.38269496867 0.7513868

4335.40076109027 0.8044622

4335.41882721188 0.7177219

4335.43689333348 0.7117966

4335.45495945508 0.7001812

4335.47302557668 0.7083343

4335.49109169829 0.7208834

4335.50915781989 0.7622656

4335.52722394149 0.8564786

4335.54529006309 0.6972253

4335.56335618469 0.697623

4335.58142230629 0.7099953

4335.5994884279 0.6820672

4335.6175545495 0.6923943

4335.6356206711 0.6781942

4335.6536867927 0.680664

4335.67175291431 0.7351975

4335.68981903591 0.7315379

4335.70788515751 0.6396883

4335.72595127911 0.6577184

4335.74401740072 0.7258629

4335.76208352232 0.7756481

4335.78014964392 0.6973746

4335.79821576552 0.694184

4335.81628188712 0.6728494

4335.83434800873 0.7128753

4335.85241413033 0.6923472

4335.87048025193 0.6895027

4335.88854637353 0.7016209

4335.90661249514 0.727879

4335.92467861674 0.7091311

4335.94274473834 0.674815

4335.96081085994 0.6861796

4335.97887698154 0.681645

4335.99694310314 0.7701523

4336.01500922475 0.855031

4336.03307534635 0.7203925

4336.05114146795 0.6319952

4336.06920758955 0.664237

4336.08727371116 0.6713131

4336.10533983276 0.6643648

4336.12340595436 0.6795248

4336.14147207596 0.6674654

4336.15953819756 0.6703809

4336.17760431917 0.6681235

4336.19567044077 0.7427233

4336.21373656237 0.671283

4336.23180268398 0.6614581

4336.24986880558 0.7105198

4336.26793492718 0.672325

4336.28600104878 0.6368731

4336.30406717038 0.601073

4336.32213329199 0.6436638

4336.34019941359 0.619693

4336.35826553519 0.6468289

4336.37633165679 0.7055144

4336.39439777839 0.5985621

4336.4124639 0.5949473

4336.4305300216 0.6271386

4336.4485961432 0.6294111

4336.4666622648 0.6348134

4336.48472838641 0.6273401

4336.50279450801 0.5963264

4336.52086062961 0.6005983

4336.53892675121 0.5586129

4336.55699287281 0.5979512

4336.57505899442 0.6757742

4336.59312511602 0.6306226

4336.61119123762 0.6352285

4336.62925735922 0.6602153

4336.64732348083 0.6141424

4336.66538960243 0.6323509

4336.68345572403 0.6557795

4336.70152184563 0.653217

4336.71958796723 0.6319199

4336.73765408884 0.6452707

4336.75572021044 0.6573315

4336.77378633204 0.6241072

4336.79185245364 0.6168233

4336.80991857525 0.5995731

4336.82798469685 0.5905247

4336.84605081845 0.650658

4336.86411694005 0.6851318

4336.88218306165 0.6689758

4336.90024918326 0.7202812

4336.91831530486 0.7575123

4336.93638142646 0.6826181

4336.95444754806 0.6231073

4336.97251366966 0.6252438

4336.99057979127 0.5899616

4337.00864591287 0.6669646

4337.02671203447 0.5942379

4337.04477815607 0.6156864

4337.06284427768 0.6125695

4337.08091039928 0.6266978

4337.09897652088 0.6548865

4337.11704264248 0.6127777

4337.13510876408 0.6027866

4337.15317488569 0.6316716

4337.17124100729 0.6092302

4337.18930712889 0.6148407

4337.20737325049 0.597519

4337.2254393721 0.60135

4337.2435054937 0.597308

4337.2615716153 0.5530519

4337.2796377369 0.5853706

4337.2977038585 0.5760173

4337.31576998011 0.5923182

4337.33383610171 0.6003534

4337.35190222331 0.6071347

4337.36996834491 0.5762326

4337.38803446652 0.5355548

4337.40610058812 0.5891221

4337.42416670972 0.6086447

4337.44223283132 0.5718027

4337.46029895292 0.5931575

4337.47836507453 0.5879356

4337.49643119613 0.5908519

4337.51449731773 0.5712648

4337.53256343933 0.5489428

4337.55062956094 0.588833

4337.56869568254 0.5730415

4337.58676180414 0.5461847

4337.60482792574 0.5553088

4337.62289404734 0.5551607

4337.64096016895 0.5372097

4337.65902629055 0.5274248

4337.67709241215 0.5401464

4337.69515853375 0.5859359

4337.71322465536 0.5755648

4337.73129077696 0.6041905

4337.74935689856 0.5334775

4337.76742302016 0.5222536

4337.78548914176 0.5101452

4337.80355526337 0.5090303

4337.82162138497 0.4476826

4337.83968750657 0.4585146

4337.85775362817 0.5323786

4337.87581974977 0.5354912

4337.89388587138 0.5149449

4337.91195199298 0.3988509

4337.93001811458 0.5045862

4337.94808423618 0.5237883

4337.96615035778 0.6121851

4337.98421647939 0.584312

4338.00228260099 0.5354372

4338.02034872259 0.5409045

4338.03841484419 0.5019595

4338.0564809658 0.4732335

4338.0745470874 0.493236

4338.092613209 0.4966974

4338.1106793306 0.5281438

4338.1287454522 0.4880323

4338.14681157381 0.4740022

4338.16487769541 0.5024833

4338.18294381701 0.543403

4338.20100993861 0.4638971

4338.21907606022 0.4672232

4338.23714218182 0.4309372

4338.25520830342 0.4629989

4338.27327442502 0.4880749

4338.29134054663 0.4977238

4338.30940666823 0.572211

4338.32747278983 0.4646617

4338.34553891143 0.4079446

4338.36360503303 0.4490636

4338.38167115464 0.4657652

4338.39973727624 0.4337709

4338.41780339784 0.3932201

4338.43586951944 0.4835508

4338.45393564105 0.4767106

4338.47200176265 0.4316551

4338.49006788425 0.4578728

4338.50813400585 0.523558

4338.52620012745 0.4989701

4338.54426624906 0.4354531

4338.56233237066 0.4884567

4338.58039849226 0.4738427

4338.59846461386 0.5066377

4338.61653073546 0.4725079

4338.63459685707 0.4304207

4338.65266297867 0.4648143

4338.67072910027 0.5101775

4338.68879522187 0.53063

4338.70686134348 0.5420254

4338.72492746508 0.4697772

4338.74299358668 0.4818552

4338.76105970828 0.4821738

4338.77912582988 0.5155247

4338.79719195149 0.4973124

4338.81525807309 0.5371031

4338.83332419469 0.5219093

4338.85139031629 0.5152744

4338.8694564379 0.4775554

4338.8875225595 0.5322741

4338.9055886811 0.4669921

4338.9236548027 0.464517

4338.9417209243 0.478393

4338.95978704591 0.4973961

4338.97785316751 0.484927

4338.99591928911 0.4599716

4339.01398541071 0.4488724

4339.03205153232 0.4712974

4339.05011765392 0.4679184

4339.06818377552 0.4544005

4339.08624989712 0.4171093

4339.10431601872 0.4526714

4339.12238214033 0.4660249

4339.14044826193 0.4758942

4339.15851438353 0.4655655

4339.17658050513 0.4336347

4339.19464662673 0.4316234

4339.21271274834 0.454947

4339.23077886994 0.4440263

4339.24884499154 0.4428505

4339.26691111314 0.4591747

4339.28497723475 0.4459457

4339.30304335635 0.4552878

4339.32110947795 0.4303914

4339.33917559955 0.4393165

4339.35724172115 0.4491156

4339.37530784276 0.4455001

4339.39337396436 0.4328395

4339.41144008596 0.4483978

4339.42950620756 0.4440354

4339.44757232917 0.4237174

4339.46563845077 0.444639

4339.48370457237 0.456616

4339.50177069397 0.4340836

4339.51983681557 0.414488

4339.53790293718 0.4014761

4339.55596905878 0.4619096

4339.57403518038 0.4089893

4339.59210130198 0.4026492

4339.61016742359 0.410158

4339.62823354519 0.4412116

4339.64629966679 0.4226146

4339.66436578839 0.4223261

4339.68243190999 0.4087041

4339.7004980316 0.3964501

4339.7185641532 0.4058384

4339.7366302748 0.4306881

4339.7546963964 0.4163415

4339.772762518 0.3441317

4339.79082863961 0.3321089

4339.80889476121 0.3868009

4339.82696088281 0.3471938

4339.84502700441 0.3677765

4339.86309312602 0.378553

4339.88115924762 0.4059383

4339.89922536922 0.3984975

4339.91729149082 0.3891309

4339.93535761242 0.337326

4339.95342373403 0.2946891

4339.97148985563 0.3277674

4339.98955597723 0.3756987

4340.00762209883 0.3989043

4340.02568822044 0.3791897

4340.04375434204 0.34752

4340.06182046364 0.3900726

4340.07988658524 0.4259662

4340.09795270685 0.3568

4340.11601882845 0.314002

4340.13408495005 0.3443884

4340.15215107165 0.3404008

4340.17021719325 0.3514181

4340.18828331485 0.3851694

4340.20634943646 0.3639878

4340.22441555806 0.3234043

4340.24248167966 0.2914179

4340.26054780126 0.2501783

4340.27861392287 0.251937

4340.29668004447 0.2921023

4340.31474616607 0.280048

4340.33281228767 0.2626883

4340.35087840927 0.254761

4340.36894453088 0.2409591

4340.38701065248 0.2687397

4340.40507677408 0.251738

4340.42314289569 0.2423094

4340.44120901729 0.2631318

4340.45927513889 0.2027158

4340.47734126049 0.2279256

4340.49540738209 0.2718882

4340.5134735037 0.2561062

4340.5315396253 0.2438344

4340.5496057469 0.2624328

4340.5676718685 0.2803687

4340.5857379901 0.2601122

4340.60380411171 0.2135982

4340.62187023331 0.1697666

4340.63993635491 0.1607128

4340.65800247651 0.2056101

4340.67606859812 0.2552333

4340.69413471972 0.2679958

4340.71220084132 0.2671457

4340.73026696292 0.2370984

4340.74833308452 0.2002921

4340.76639920613 0.239994

4340.78446532773 0.2800114

4340.80253144933 0.2458848

4340.82059757093 0.226704

4340.83866369254 0.2682966

4340.85672981414 0.2794606

4340.87479593574 0.2785457

4340.89286205734 0.2542388

4340.91092817894 0.2233959

4340.92899430055 0.2394128

4340.94706042215 0.2555531

4340.96512654375 0.2667029

4340.98319266535 0.2665818

4341.00125878696 0.2514672

4341.01932490856 0.2816197

4341.03739103016 0.3131492

4341.05545715176 0.3068253

4341.07352327336 0.3038718

4341.09158939497 0.3032014

4341.10965551657 0.3239356

4341.12772163817 0.3294708

4341.14578775977 0.3142316

4341.16385388137 0.2822783

4341.18192000298 0.2676347

4341.19998612458 0.3514022

4341.21805224618 0.385704

4341.23611836778 0.3099531

4341.25418448939 0.2959245

4341.27225061099 0.3656794

4341.29031673259 0.3702039

4341.30838285419 0.3433253

4341.32644897579 0.3692361

4341.3445150974 0.3709147

4341.362581219 0.3763471

4341.3806473406 0.4320615

4341.3987134622 0.407221

4341.41677958381 0.4073149

4341.43484570541 0.3950454

4341.45291182701 0.4116159

4341.47097794861 0.3902885

4341.48904407021 0.3652582

4341.50711019182 0.4032641

4341.52517631342 0.3800364

4341.54324243502 0.3472518

4341.56130855662 0.3779497

4341.57937467823 0.3697661

4341.59744079983 0.3179136

4341.61550692143 0.3467109

4341.63357304303 0.4373739

4341.65163916463 0.4525129

4341.66970528624 0.4140293

4341.68777140784 0.4281907

4341.70583752944 0.4316369

4341.72390365104 0.4105518

4341.74196977264 0.4092783

4341.76003589425 0.4471444

4341.77810201585 0.4681737

4341.79616813745 0.4415865

4341.81423425905 0.4420964

4341.83230038066 0.4764367

4341.85036650226 0.4696785

4341.86843262386 0.4168736

4341.88649874546 0.3924019

4341.90456486706 0.4411311

4341.92263098867 0.4771096

4341.94069711027 0.4333405

4341.95876323187 0.4003121

4341.97682935347 0.4173197

4341.99489547508 0.4378903

4342.01296159668 0.4509346

4342.03102771828 0.4687889

4342.04909383988 0.4465697

4342.06715996148 0.4255937

4342.08522608309 0.4143799

4342.10329220469 0.3800864

4342.12135832629 0.3852474

4342.13942444789 0.4337795

4342.15749056949 0.4430881

4342.1755566911 0.4454864

4342.1936228127 0.4513828

4342.2116889343 0.4666566

4342.2297550559 0.4762096

4342.24782117751 0.4691758

4342.26588729911 0.4794889

4342.28395342071 0.4804673

4342.30201954231 0.4445474

4342.32008566392 0.4278982

4342.33815178552 0.4323859

4342.35621790712 0.4841257

4342.37428402872 0.4819815

4342.39235015032 0.441438

4342.41041627193 0.4553198

4342.42848239353 0.4417259

4342.44654851513 0.4042815

4342.46461463673 0.4607043

4342.48268075834 0.4942758

4342.50074687994 0.4698751

4342.51881300154 0.4705828

4342.53687912314 0.4775702

4342.55494524474 0.474535

4342.57301136635 0.5026291

4342.59107748795 0.5232224

4342.60914360955 0.4911437

4342.62720973115 0.4562554

4342.64527585276 0.442997

4342.66334197436 0.4759186

4342.68140809596 0.5542932

4342.69947421756 0.5599814

4342.71754033916 0.5234861

4342.73560646077 0.4682123

4342.75367258237 0.4537795

4342.77173870397 0.4798328

4342.78980482557 0.5072622

4342.80787094717 0.4687819

4342.82593706878 0.4599575

4342.84400319038 0.5109959

4342.86206931198 0.5333231

4342.88013543358 0.5146965

4342.89820155519 0.4894496

4342.91626767679 0.4912621

4342.93433379839 0.5190186

4342.95239991999 0.5473753

4342.97046604159 0.5562649

4342.9885321632 0.5664702

4343.0065982848 0.5555089

4343.0246644064 0.5560688

4343.042730528 0.5713795

4343.06079664961 0.526884

4343.07886277121 0.4735637

4343.09692889281 0.4867711

4343.11499501441 0.5127084

4343.13306113601 0.4996335

4343.15112725762 0.4928372

4343.16919337922 0.5251814

4343.18725950082 0.5308816

4343.20532562242 0.527661

4343.22339174403 0.5747703

4343.24145786563 0.6032597

4343.25952398723 0.5655264

4343.27759010883 0.5275292

4343.29565623043 0.5304613

4343.31372235204 0.5743678

4343.33178847364 0.5887767

4343.34985459524 0.5228958

4343.36792071684 0.4860984

4343.38598683844 0.496437

4343.40405296005 0.5322039

4343.42211908165 0.5296021

4343.44018520325 0.5492752

4343.45825132485 0.5593502

4343.47631744646 0.5752795

4343.49438356806 0.5866058

4343.51244968966 0.57729

4343.53051581126 0.5841388

4343.54858193287 0.5809201

4343.56664805447 0.559873

4343.58471417607 0.5771945

4343.60278029767 0.568417

4343.62084641927 0.510403

4343.63891254088 0.5291314

4343.65697866248 0.5915436

4343.67504478408 0.5814049

4343.69311090568 0.5471368

4343.71117702729 0.5370488

4343.72924314889 0.5544181

4343.74730927049 0.5247738

4343.76537539209 0.5234402

4343.78344151369 0.5704433

4343.8015076353 0.5948731

4343.8195737569 0.612398

4343.8376398785 0.5951105

4343.8557060001 0.5679193

4343.8737721217 0.5758804

4343.89183824331 0.5785276

4343.90990436491 0.5760263

4343.92797048651 0.5618696

4343.94603660811 0.5584309

4343.96410272972 0.5407448

4343.98216885132 0.5323356

4344.00023497292 0.5820559

4344.01830109452 0.5922828

4344.03636721612 0.5367715

4344.05443333773 0.5129471

4344.07249945933 0.5508969

4344.09056558093 0.5760587

4344.10863170253 0.5566722

4344.12669782413 0.5639739

4344.14476394574 0.5756455

4344.16283006734 0.5850127

4344.18089618894 0.6087307

4344.19896231054 0.6263431

4344.21702843215 0.5961618

4344.23509455375 0.5756977

4344.25316067535 0.5716164

4344.27122679695 0.5788653

4344.28929291856 0.5873812

4344.30735904016 0.5576109

4344.32542516176 0.5956134

4344.34349128336 0.6047027

4344.36155740496 0.6299784

4344.37962352656 0.5786902

4344.39768964817 0.5512559

4344.41575576977 0.5571164

4344.43382189137 0.5674151

4344.45188801297 0.5912184

4344.46995413458 0.5975744

4344.48802025618 0.6104143

4344.50608637778 0.6372982

4344.52415249938 0.6267673

4344.54221862098 0.585752

4344.56028474259 0.5534077

4344.57835086419 0.5682516

4344.59641698579 0.5904813

4344.61448310739 0.5850306

4344.632549229 0.5677843

4344.6506153506 0.5748983

4344.6686814722 0.6092515

4344.6867475938 0.6175805

4344.70481371541 0.5856376

4344.72287983701 0.6176654

4344.74094595861 0.6487208

4344.75901208021 0.6422305

4344.77707820181 0.6359649

4344.79514432342 0.6337568

4344.81321044502 0.6256254

4344.83127656662 0.6274887

4344.84934268822 0.6412376

4344.86740880983 0.6292351

4344.88547493143 0.6274048

4344.90354105303 0.65365

4344.92160717463 0.6614866

4344.93967329623 0.653108

4344.95773941783 0.6714731

4344.97580553944 0.6969373

4344.99387166104 0.7288108

4345.01193778264 0.7110088
[truncated: 4,826,382 more chars]
